# Synthesis and Application of Chiral 2-Amido and 1-Phenyl-2-amido Dienes in Diels–Alder Reactions to Access Chiral Cyclic Ketones

**DOI:** 10.1021/acs.joc.4c00689

**Published:** 2024-11-05

**Authors:** Aoibheann O’ Connor, Calvin Q. O’Broin, Julia Bruno-Colmenarez, Patrick J. Guiry

**Affiliations:** Centre for Synthesis and Chemical Biology, School of Chemistry, University College Dublin, Belfield, Dublin 4, Ireland

## Abstract

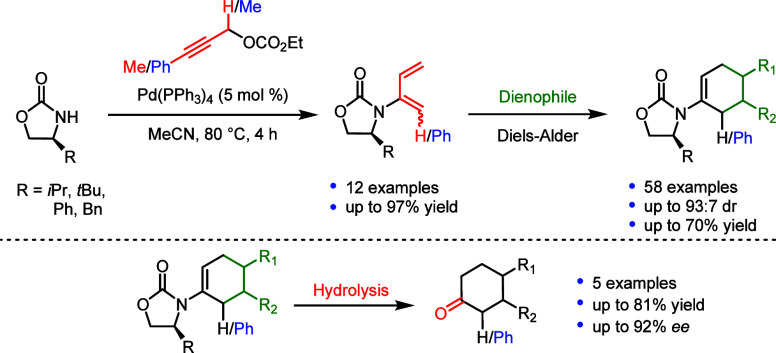

The preparation of a focused library of chiral 2-amido and 2-amido-1-phenyl-1,3-dienes from a range of chiral oxazolidinones using palladium-catalysis is reported. This palladium-catalyzed carbon–nitrogen bond-forming reaction provides the corresponding chiral amido-dienes in moderate to excellent yields (12 examples, up to 97%). The resulting chiral amido-dienes are employed as novel dienes in Diels–Alder (DA) reactions (58 examples, up to 93:7 dr, up to 70% yield). A range of chiral cyclic ketones were accessed upon hydrolysis, affording products with high levels of enantioselectivity (up to 92% ee, up to 81% yield).

## Introduction

**Scheme 1:**
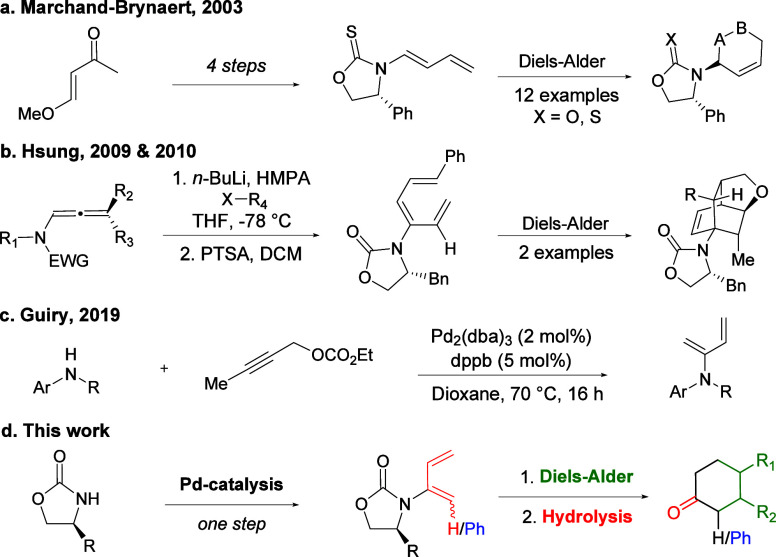
Previous Work

Few methods for C–C bond formation are as powerful as the Diels–Alder (DA) reaction, which has seen strategic evolution over the last century.^1,2^ Interest in the asymmetric variant of this reaction is an active area of research, with many natural products and biologically relevant molecules accessed through this transformation.^3−5^ Extensive research has been carried out to investigate the stereoselectivity of employing a chiral dienophile, beautifully demonstrated by the work of Evans on the use of chiral oxazolidinones as chiral auxiliaries.^6^ Further development of chiral catalysts by Koga et al. and Corey et al. has also shown significant success.^7,8^ However, studies in the DA reaction employing chiral dienes are much less common and the slow development of this specific topic may be ascribed to the difficulty of preparing the requisite chiral dienes as well as previous suggestions that the chiral information would be too distant from the reaction center to exert sufficient control.^5^ Conjugated dienes are important synthetic precursors to a variety of functionalized intermediates due to the reactive dienyl group, which can be transformed by a range of cycloaddition and functionalization reactions to afford products in a stepwise manner with up to four stereocenters.^9−11^ It is well documented that C–N bonds are present in pharmaceuticals and functionalized materials and can be readily further transformed.^12−14^ Enders and Meyer have comprehensively reviewed the varied modern syntheses toward 2-amino-1,3-dienes.^15^ Chiral variants of these amino 1,3-dienes containing a pyrrolidine have been reported by Rawal and Kozmin; however, limited examples of chiral amido 1,3-dienes have been reported to date.^16^ Notable contributions from Marchand-Brynaert et al. and Hsung et al., among others, exploited the use of chiral oxazolidinones (Scheme 1a,b); however, they are prepared in multistep syntheses.^17−22^ In these studies, the amido-dienes are used in a range of elegant DA reactions, although the auxiliary is not removed. Previously, our group has successfully developed a Pd-catalyzed dienylation, whereby the use of propargyl electrophiles, known for their diverse modes of reactivity, provided access to this valuable 1,3-diene motif derived from a range of secondary and tertiary amines (Scheme 1c).^23^

Utilizing chiral oxazolidinones, such as Evans’ auxiliaries in the asymmetric DA reaction, provides the basis for the current work, which applies palladium catalysis to prepare a range of novel and known chiral 2-amido- and 2-amido-1-phenyl-1,3-dienes in a single step followed by hydrolysis of the DA products to recycle the chiral auxiliary and access a range of chiral cyclic ketones (Scheme 1d).^6,18,24−29^

## Results and Discussion

**Scheme 2:**
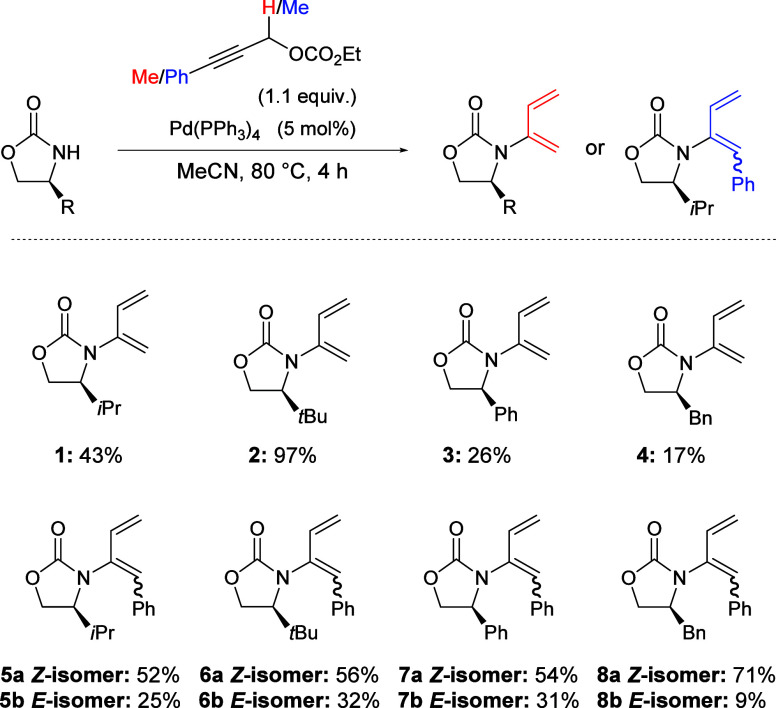
Pd-Catalyzed Dienylation of Chiral Oxazolidinones Affording 2-Amido-1,3-dienes **1**–**4** and 2-Amido-1-phenyl-1,3-dienes **5a**–**8b**

A range of chiral oxazolidinones were prepared from the corresponding chiral amino alcohols following literature procedures.^30,31^ The *i*-Pr-, *t*-Bu-, Ph-, and Bn-substituted oxazolidinones were tested in a Pd-catalyzed decarboxylative dienylation, optimized for amides. This optimization included using Pd(PPh_3_)_4_ instead of the first studied Pd_2_dba_3_ catalytic system and decreasing the reaction times from 16 to 4 h to afford the resulting known and novel dienylated oxazolidinones **1**–**4** in yields of up to 97% (Scheme 2).^18^

Efforts to further explore the scope of this novel reaction included the use of a Ph-substituted propargyl carbonate, which could act as an alternative source of π-propargyl species in the Pd-catalyzed dienylation. The Ph-substituted propargyl carbonate was tested in the Pd-catalyzed dienylation with the *i*-Pr-, *t*-Bu-, Ph-, and Bn-substituted oxazolidinones to afford the chiral 2-amido-1-phenyl-1,3-dienes **5a**–**8b** in moderate to good yields as mixtures of *E*- and *Z*-isomers (Scheme 2). It was observed that there was an overall preference for the formation of the *Z*-isomer, irrespective of the chiral auxiliary used. However, the Bn oxazolidinone showcased the largest proportion of Z-isomer, which was isolated in a 71% yield as opposed to the *E-*isomer which was only isolated in a 9% yield.

**Figure 1:**
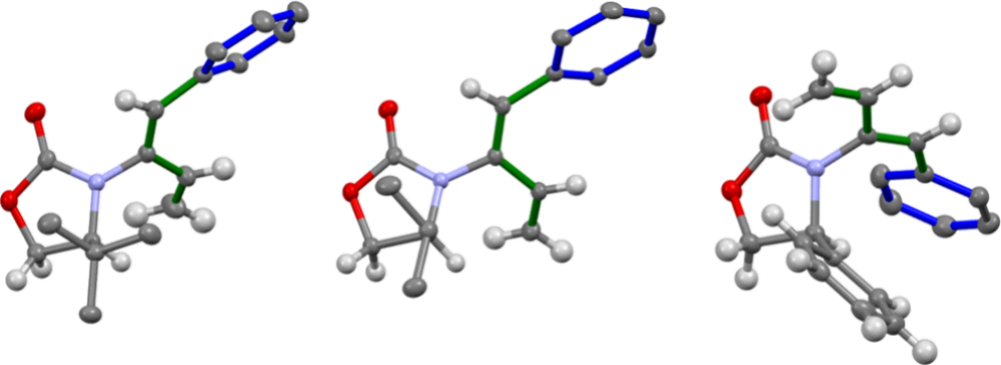
XRD Structures of **6b**, **5b**, and **7a**, respectively.

Pleasingly, the isomers were separable by column chromatography. XRD analysis confirmed the structures of novel compounds **5b, 6b**, and **7a** (Figure 1). Products **3**, **4**, **7b**, and **8b** have previously been reported in the literature.^19^ Overall, the 2-amido-1-phenyl-1,3-dienes **5a** – **8b** were formed in high yields.

**Figure 2:**
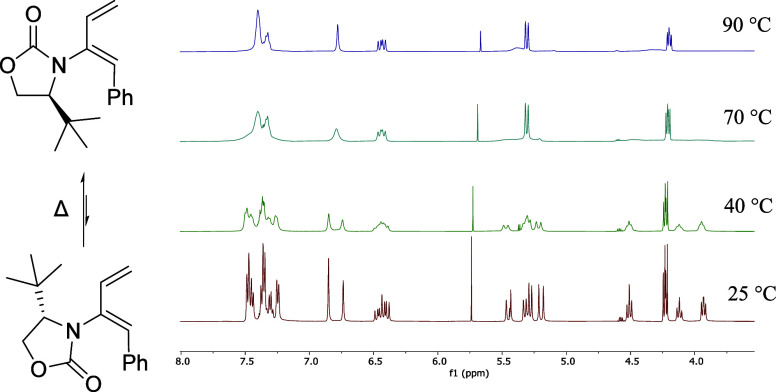
Variable temperature (VT) ^1^H NMR spectra of Z-**6a**, illustrating the presence of rotamers in solution.

It was observed that there were additional peaks in the ^1^H NMR spectrum of the *Z*-isomer **6a** and further analysis by variable temperature (VT) ^1^H NMR spectroscopy indicated that the *Z*-isomer exists as rotamers. Only at temperatures above 70 °C the barrier to rotation was overcome and peaks began to coalesce for long enough for the detection of a single conformer (Figure 2). The unfavorable steric clash between the phenyl ring of the 1,3-diene moiety and the *t*-Bu group of the oxazolidinone leads to restricted rotation about the C–N bond.

**Scheme 3:**
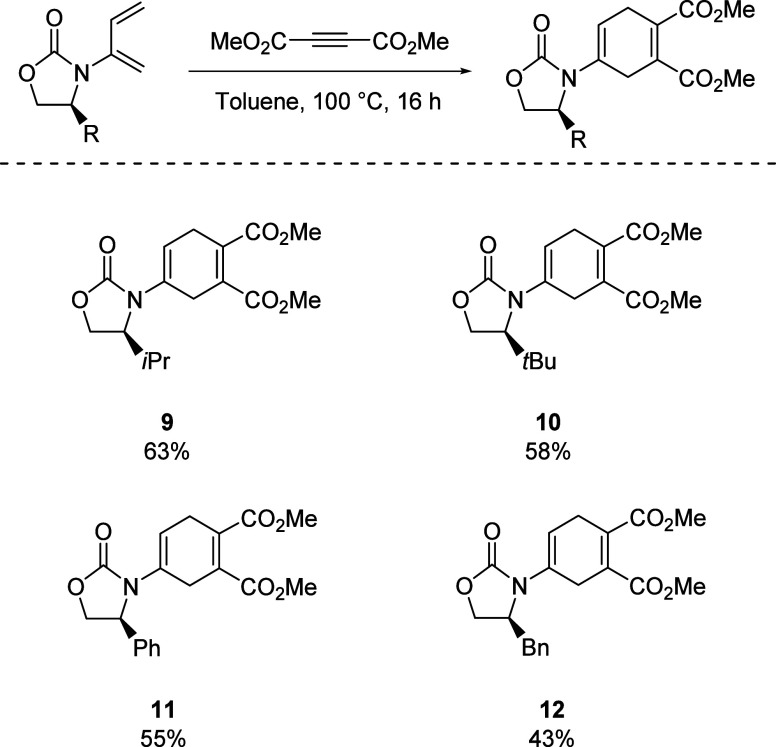
Probing Reactivity of Dienes with Symmetric Dienophile

Investigations into the reactivity of the chiral 2-amido-1,3-dienes **1–4** in the DA reaction were carried out using dimethylacetylene dicarboxylate as the dienophile. The desired products **9–12** were isolated in yields of 43% to 63% (Scheme 3). While this was only representative of a symmetric DA reaction rather than the asymmetric variant, it would probe the reactivity of the dienes in this transformation. Despite the moderate yields, we were pleased that the dienes **1–4** showed the requisite reactivity to undergo the cycloaddition.

**Scheme 4:**
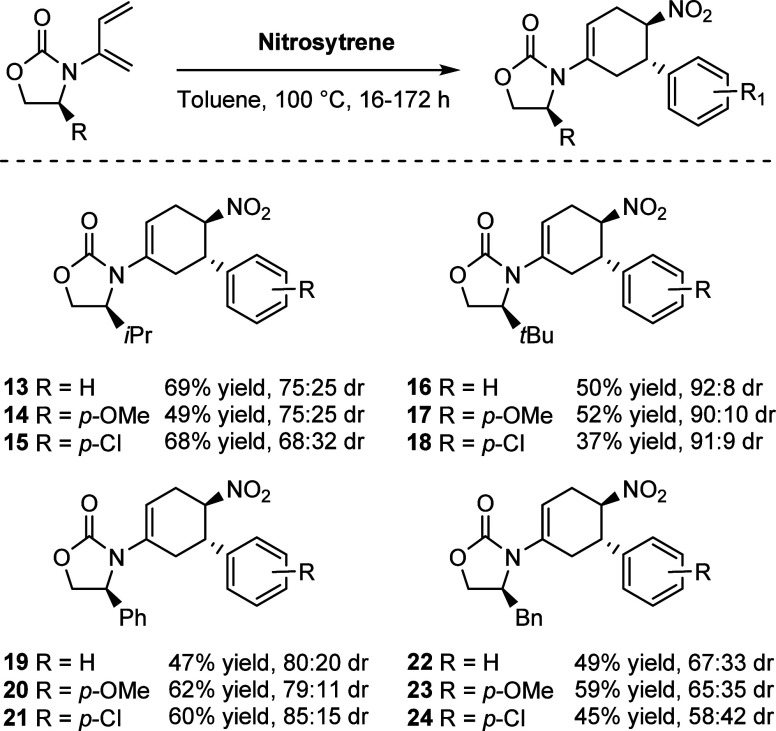
Scope of Diels–Alder with Dienes **1–4**

**Scheme 5:**
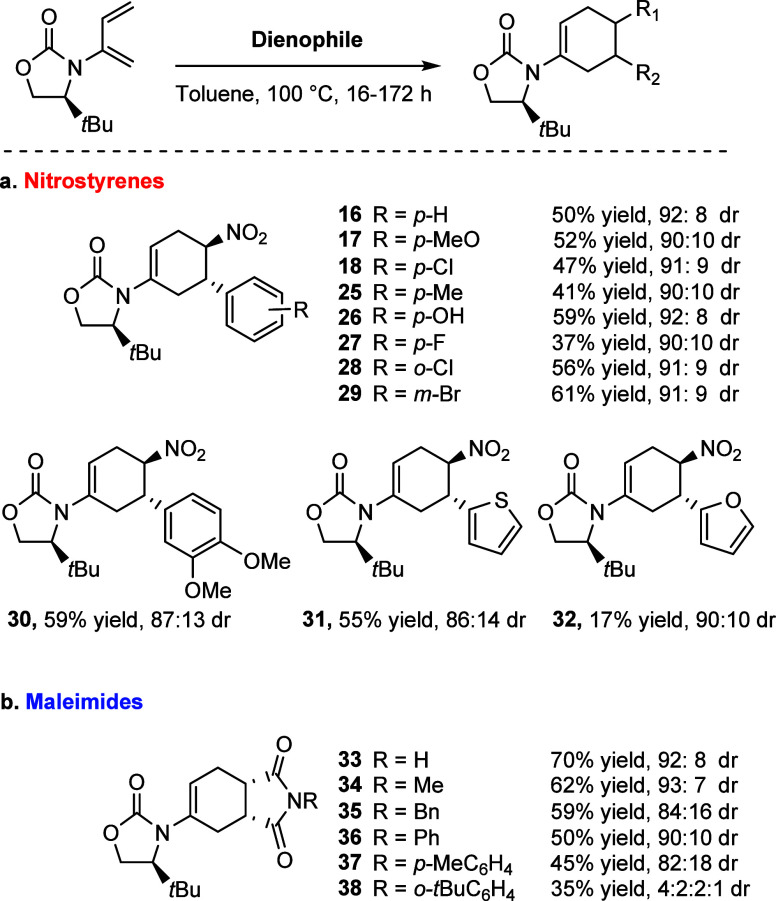
Scope of Diels–Alder with Model 2-Amido-diene

With these results in hand, further work could be undertaken to assess the potential of the chiral auxiliaries to induce asymmetry in the DA reaction. The dienes **1–4** were employed in an asymmetric DA reaction with a range of substituted nitrostyrenes. The desired compounds were obtained in moderate to good yields, where a clear trend of diastereoselectivity was observed (Scheme 4). *Trans-*β-nitrostyrene, 4-methoxy-*trans*-β-nitrostyrene, and 4-chloro-*trans*-β-nitrostyrene were employed as dienophiles. The *i-*Pr diene **1** gave yields of 49–68% of the desired products and displayed moderate diastereoselectivity of up to 75:25 dr. The diastereomers present were not separable by column chromatography. This would remain a trend for most of the nitrostyrene products formed. The *t*-Bu diene **2** gave yields of 37–52% of the desired products. Although the *para-*Cl-substituted nitrostyrene **18** was only isolated in a low yield compared to **15**, the levels of diastereoselectivity were far superior, up to 92:8 dr. The Ph diene **3** gave yields of 47–62% of the desired products and displayed good diastereoselectivity of up to 85:15 dr. The Bn diene **4** was also employed and gave only moderate yields and poor diastereoselectivity of up to 67:33, with drs as low as 57:43. It was in these cases where the regiomeric 1,3-product was most prevalent and in the case of **22**, was observable by ^1^H NMR spectroscopy where the regioisomeric ratio was 70:30. It was possible, however, to isolate the major diastereomer of **22**. In all cases the 1,4-product of the DA reaction was obtained as the major regioisomer. Generally, taking into account steric considerations, the trend observed in the general diastereoselectivity of the dienes **1–4** is not surprising. The dienes bearing the substituents with greater steric bulk (*t*-Bu > Ph > *i-*Pr > Bn) had greater potential to induce asymmetry in the formation of the corresponding DA products, despite their distance from the reaction center. Overall, the *t*-Bu diene **2** led to the greatest levels of asymmetric induction, delivering the desired products in moderate to good yields and diastereoselectivities of up to 92:8 dr. Therefore, diene **2** was employed as the model substrate for exploring the substrate scope (Scheme 5).

The reaction of diene **2** with a range of substituted nitrostyrenes afforded products **16**–**32** with high levels of diastereoselectivity and moderate to good yields (17–61%) (Scheme 5a). Pleasingly, substitution at the *para-*position was well tolerated, including incorporation of electron-donating groups (**17, 25**, and **26**) giving good yields and excellent diastereoselectivity of up to 92:8. Additionally, electron-withdrawing groups (**18** and **27**) gave consistent diastereoselectivities of 90:10 or higher. Furthermore, *meta*- and *ortho*-substitution also saw no change in diastereoselectivity (**28** and **29**). Disubstitution on the aromatic ring, while maintaining a good yield, saw a slight fall in diastereoselectivity to 87:13 dr as compared with the aforementioned products. Furthermore, it was possible to incorporate more complex nitrostyrenes such as those possessing heteroaromatic systems. Their employment in the asymmetric DA reaction with the model substrate **2** also revealed impressive diastereoselectivities of up to 90:10 (**31** and **32**), although the furan product was formed in a low yield of 17%.

**Figure 3:**
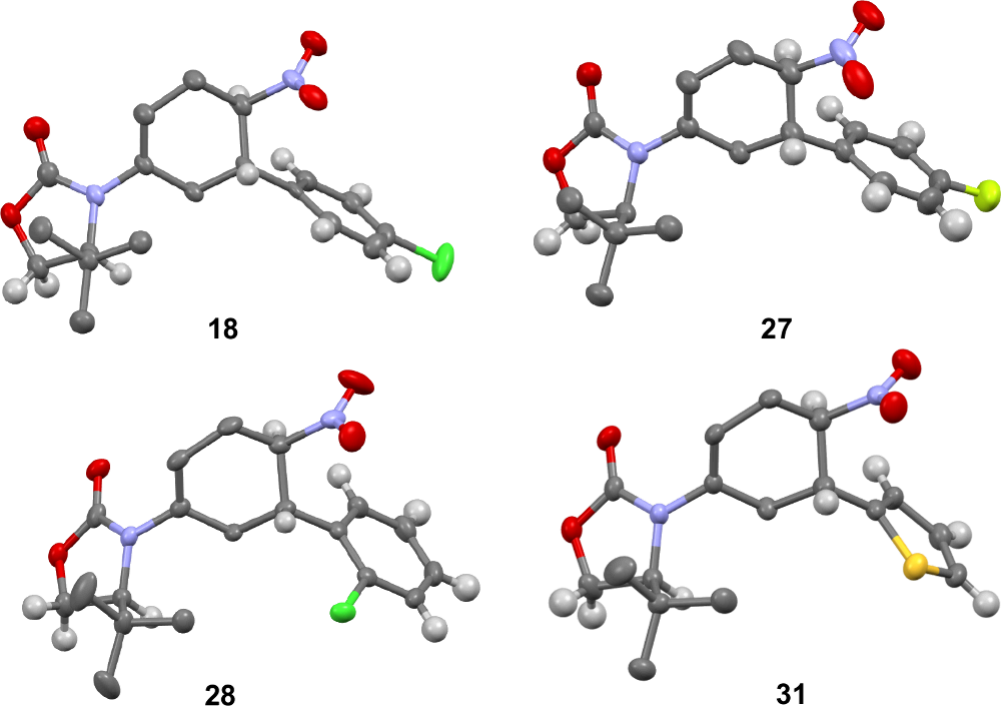
XRD Structures of **18**, **27**, **28**, and **31**.

We utilized XRD analysis to ascertain the structure of four of the aforementioned compounds; **18**, **27**, **28**, and **31.** This allowed us to confirm their structure and stereochemistry. In doing so, we can demonstrate the consistency of this system to deliver the (6*R*, 1*S*), configuration in the newly formed cyclohexene products, irrespective of the substitution pattern on the nitrostyrene (Figure 3).

**Scheme 6:**
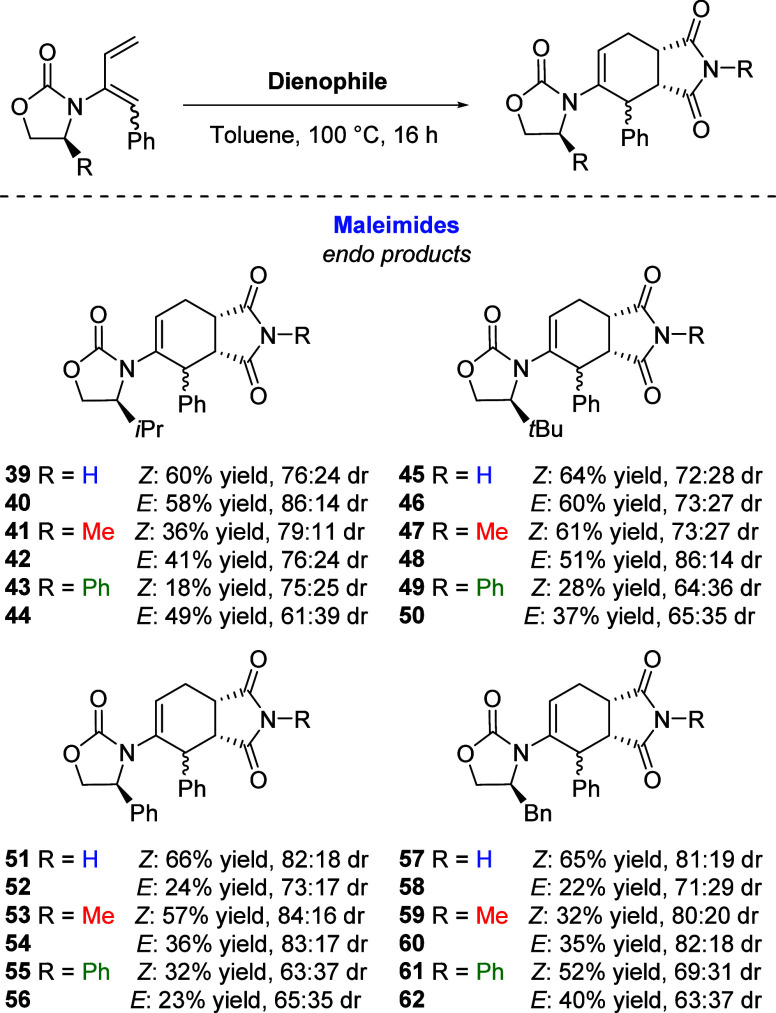
Diels–Alder with 1-Phenyl-2-amido Dienes

It was also of interest to explore other dienophiles that would provide asymmetric products, so that we could further investigate the potential of the *t-*Bu diene **2** to induce asymmetry. We chose to use substituted maleimides, known as a common choice of dienophile in DA chemistry. Pleasingly, the substituted maleimides also provided high levels of diastereoselectivity and moderate to good yields. The major (*endo*) and minor (*exo*) diastereomers were separated by column chromatography and isolated. Alkyl (**34** and **35**) and aromatic (**36**–**38**) substitutions were well tolerated (Scheme 6).

**Figure 4:**
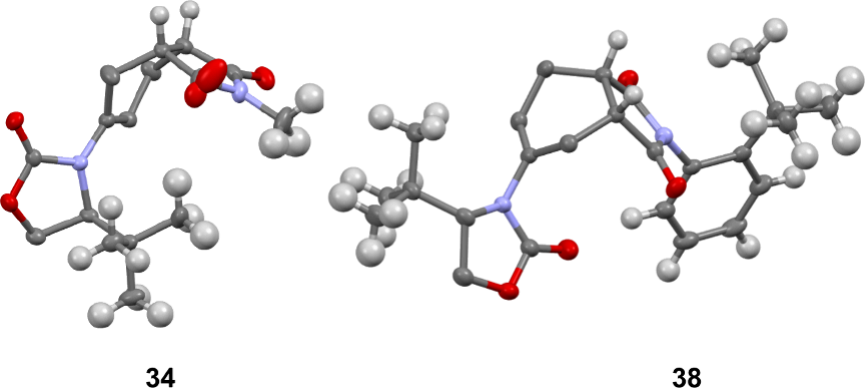
XRD Structures of **34** and **38**.

The yields reported are for the *endo*-products. In the interesting example of **38**, both central and axial chirality are present and **38** was formed with a diastereoselectivity of 4:2:2:1, the major *endo* product of which, along with cycloadduct **34**, was further characterized by XRD analysis (Figure 4).

The 2-amido-1-phenyl-1,3-dienes (**5a–8b**) were also employed in DA reactions with a range of substituted maleimides (Scheme 6). The major product in each case was the *endo*-product, and yields are reported as such. Although in these examples we do not see the high levels of diastereoselectivity observed with diene **2**, they are still significant and showcase good diastereoselectivity (up to 84:16) and moderate to good yields. It was observed that generally the use of the *Z*-isomer results in a slight improvement in diastereoselectivity for the *i*-Pr systems **41**–**44**. This trend is not observed for the *t*-Bu (**45–50**), Ph (**51–56**), and Bn (**57–62**) systems.

**Scheme 7:**
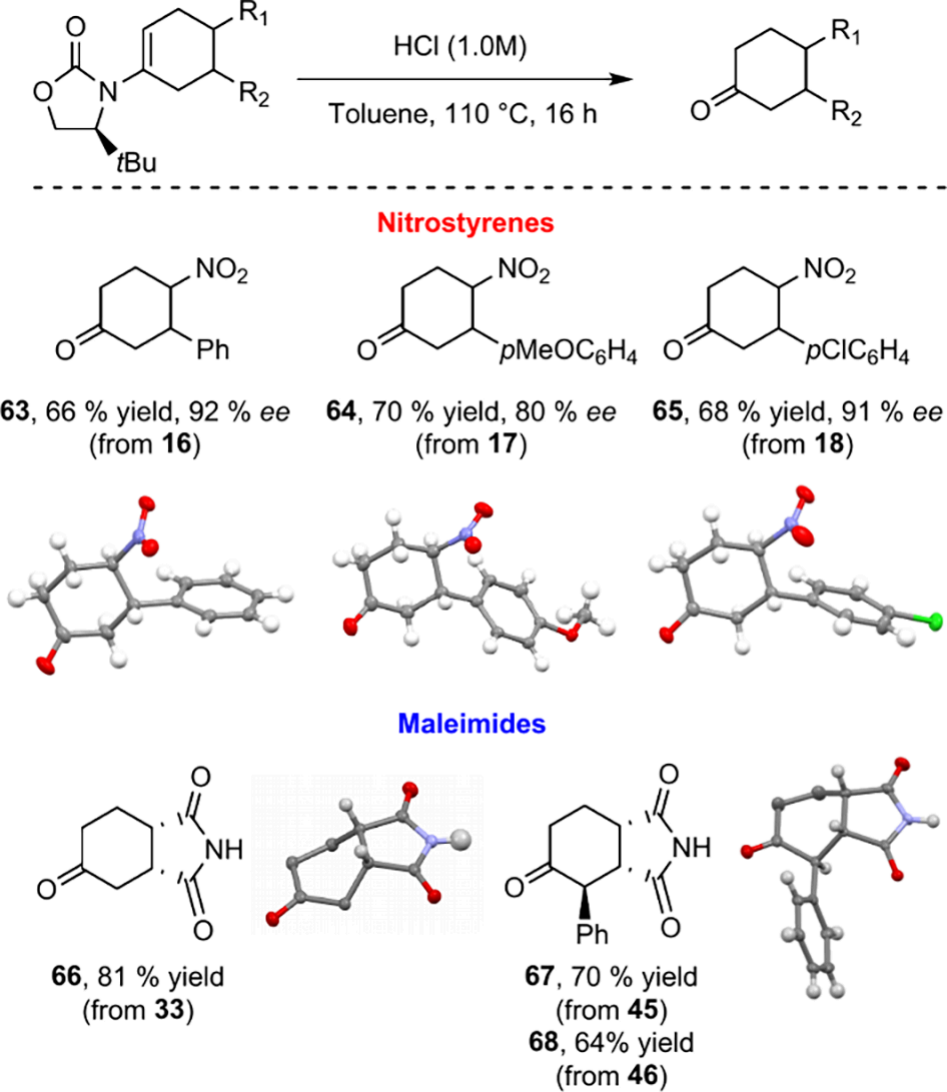
Hydrolysis of Diels–Alder Products

Following the preparation of the DA products (**9–62**), it was of interest to cleave the chiral auxiliary *via* hydrolysis, thus revealing the chiral cyclic ketone. Employing the DA products in the hydrolysis reaction would allow for the investigation of the enantioselectivity of the isolated ketone products as well as the potential of recycling the chiral auxiliary for further use. Initial attempts of hydrolysis were unsuccessful, despite large equivalents of HCl (up to 100 equiv). It was later revealed that performing the reaction at reflux was imperative in order to obtain full consumption of the starting material. As a result, the chiral auxiliaries could then be successfully removed *via* hydrolysis with HCl (1.0 M) at reflux (Scheme 7). Compounds **63–65** were isolated in high levels of enantioselectivity (80–92% *ee*) and good yields of 66–70%.^21^ No degradation of the chiral auxiliary nor desired product was observed under these conditions. Using this approach, novel compounds **65**–**67** were prepared and also saw no degradation or undesired reactivity. XRD analysis was used to confirm the structures of **63**–**67** and revealed the retention of stereochemistry from the DA products to the corresponding chiral ketones.

**Scheme 8:**
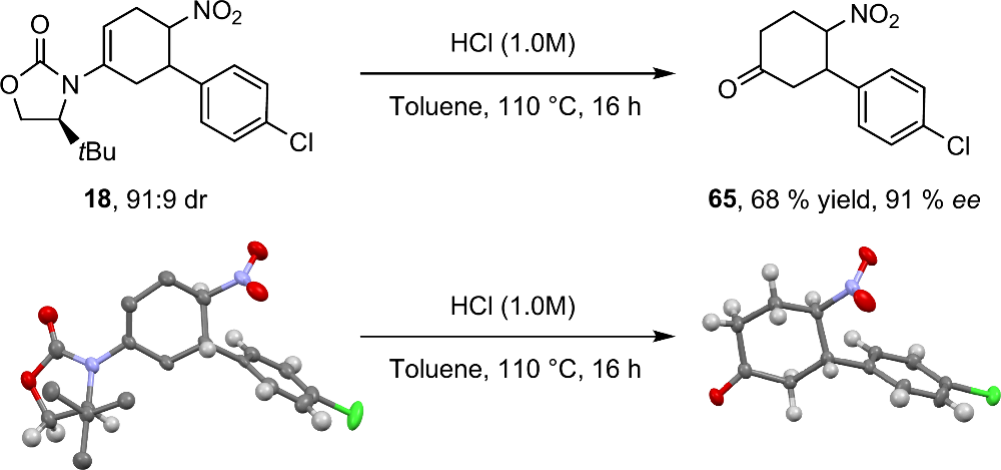
XRD Analysis Reveals Retention of Stereochemistry from **18** to **65**

Following the hydrolysis of the chiral auxiliary from the DA product **18**, the enantioselectivity of the product could be determined using SFC. The desired compound was isolated in a 68% yield and 91% ee. The structure of both the DA product and the hydrolysis product could be confirmed using XRD analysis (Scheme 8). The use of XRD in this context could confirm that there was no change in stereochemistry during the harsh conditions of the hydrolysis reaction (high temperature, stoichiometric acid). This further emphasizes the consistency of our strategy to access chiral cyclic ketones in high enantioselectivity with predictable stereochemistry through the use of the chiral diene **2** and nitrostyrenes.

**Scheme 9:**
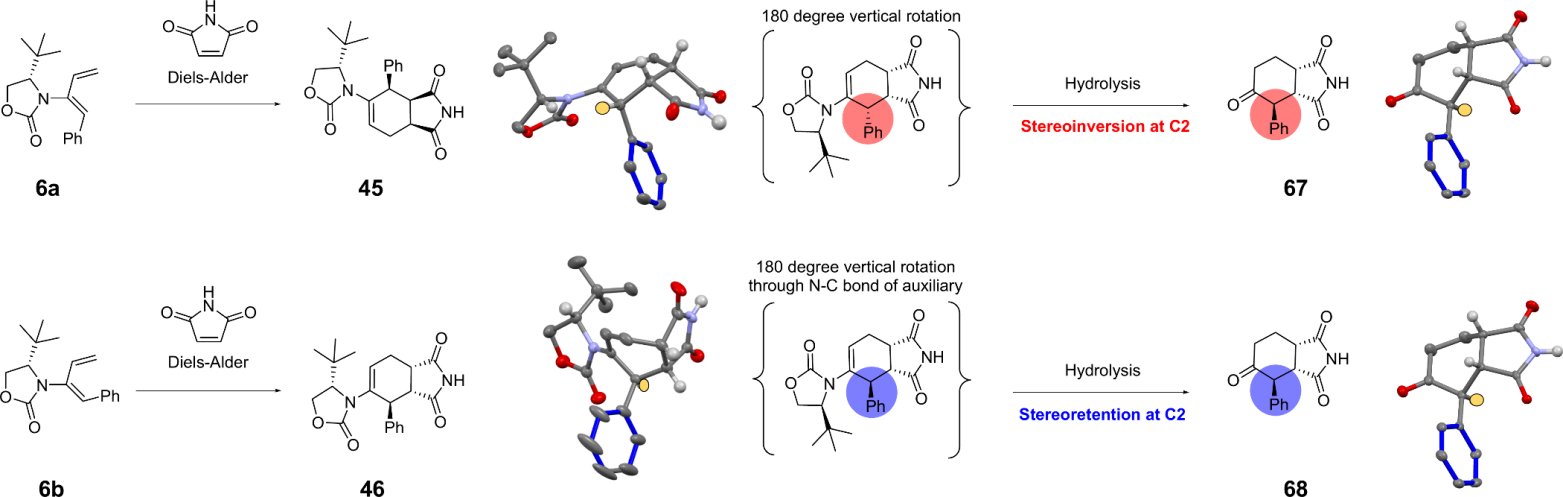
Stereochemical Investigation of Diels–Alder

An investigation using XRD analysis was carried out (Scheme 9) to confirm the stereochemistry of the DA products **45** and **46** as curiously the corresponding hydrolyzed products **67** and **68** had identical stereochemistry. When employing the *Z*-isomer and *E*-isomer of dienes **5a–8b** in the DA reaction with maleimides, it was observed that both isomers gave products with similar spectral features. It was therefore difficult in some cases to ascertain the structure of the corresponding DA product. We anticipated that using different geometric isomers would deliver different diastereomers of the DA products (**38**–**61**) as well as different diastereomers of the corresponding hydrolyzed products. Utilizing XRD analysis, we would be able to obtain crucial stereochemical information, not possible from 2D NMR experimentation alone.

This study revealed that during the hydrolysis process, compound **45** undergoes stereoinversion at C2, leading to the product **67**. This is likely due to the epimerization of the stereocenter on the relatively acidic chiral center under acidic conditions to the more stable conformation. We believe this conformation has less steric strain and is therefore lower in energy, and is the favorable conformation for the product to adopt. When analyzing the XRD structure of **46**, it was clear the Ph group was orientated differently and was indeed a diastereomer of **45**, as anticipated. It was therefore of interest to explore whether the hydrolyzed product would also undergo stereoinversion at C2. However, stereoinversion was not apparent and instead, we observed the identical hydrolyzed product, **68**. This was corroborated using XRD analysis. In this example, XRD analysis provided crucial insight, not possible from ^1^H NMR spectroscopic studies alone.

**Scheme 10:**
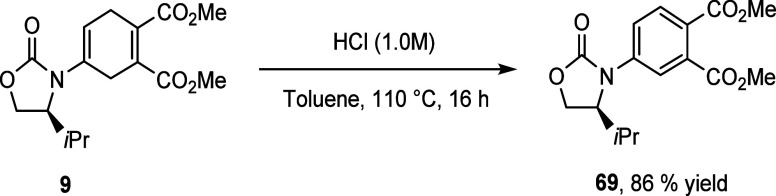
Aromatization of the DA Product

Another hydrolysis study performed to cleave the chiral auxiliary and reveal a chiral cyclic ketone gave rise to an unexpected product (Scheme 10). The DA product **9** obtained from our symmetric DA was employed in the hydrolysis reaction, utilizing the same conditions as previously described (Scheme 8). The desired chiral auxiliary and ketone products were not observed but rather under the acidic conditions provided the aromatized product **69** in a good yield of 86%. Crude ^1^H NMR spectroscopy revealed that neither the organic or aqueous layer revealed the presence of the chiral auxiliary nor the desired ketone product, revealing that the reaction proceeds in good levels of conversion. The aromatized product is observed in less than 10% in the crude ^1^H NMR spectrum of the DA product **9**, suggesting the importance of oxygen as the oxidant in the reaction, as well as the necessity of the acidic environment. There exists some literature precedent of this transformation.^32^

## Conclusions

A range of novel and known 2-amido- and 2-amido-1-phenyl-1,3-dienes have been synthesized in a single step with yields up to 97% using palladium catalysis. The 2-amido-1-phenyl-1,3-dienes **5a–8b** were formed as separable mixtures of *E*- and *Z*-isomers, three of which were characterized by X-ray crystallography and one, *Z*-isomer **6a**, exhibited restricted bond rotation about the C–N bond. The dienes **1**–**8b** were tested in a variety of Diels–Alder reactions to afford novel products with high diastereoselectivity (up to 93:7) in moderate to good yields (up to 70%). Subjecting these products to hydrolysis allowed for the isolation of novel and known chiral cyclic ketones in high levels of enantioselectivity (up to 92% ee) and good yields (up to 81%). XRD analysis provided crucial structural information for a range of products in each step of this study.

## Experimental Section

### General Procedure 1 for the Preparation of Chiral Dienes

A 10 mL Schlenk flask equipped with a stir bar was evacuated and backfilled with N_2_ three times. Chiral oxazolidinone (0.35 mmol, 1.0 equiv) and Pd(PPh_3_)_4_ (0.0175 mmol, 0.05 equiv) were charged simultaneously followed by anhydrous acetonitrile (0.1 M) and propargyl carbonate (0.038 mmol, 1.1 equiv) *via* a syringe. The reaction mixture was stirred at 80 °C over 4 h. The solution was concentrated *in vacuo* and purified by flash column chromatography (3:1 cyclohexane:EtOAc) to afford the product.

### Procedure for Gram-Scale Synthesis of (**2**)

A 25 mL Schlenk flask equipped with a stir bar was evacuated and backfilled with N_2_ three times. (*S*)-4-*tert*-Butyloxazolidin-2-one (0.1430 g, 1.00 mmol, 1.0 equiv) and Pd(PPh_3_)_4_ (0.0578 g, 0.05 mmol, 0.05 equiv) were charged simultaneously followed by anhydrous acetonitrile (0.1 M) and but-2-yn-1-yl ethyl carbonate (0.10 mL, 1.1 mmol, 1.1 equiv., *p* = 1.36 g/cm^3^) *via* a syringe. The reaction mixture was stirred at 80 °C over 4 h. The solution was concentrated *in vacuo* and purified by flash column chromatography (3:1 cyclohexane:EtOAc) to afford the product **2** as a yellow oil (0.1794 g, 92%).

### Procedure for Gram-Scale Synthesis of (**6a** and **6b**)

A 25 mL Schlenk flask equipped with a stir bar was evacuated and backfilled with N_2_ three times. (*S*)-4-*tert*-Butyloxazolidin-2-one (0.1430 g, 1.00 mmol, 1.0 equiv) and Pd(PPh_3_)_4_ (0.0578 g, 0.05 mmol, 0.05 equiv) were charged simultaneously, followed by anhydrous acetonitrile (0.1 M) and ethyl (4-phenylbut-3-yn-2-yl) carbonate (0.21 mL, 1.1 mmol, 1.1 equiv., *p* = 1.12 g/cm^3^) *via* a syringe. The reaction mixture was stirred at 80 °C over 4 h. The solution was concentrated *in vacuo* and purified by flash column chromatography (3:1 cyclohexane:EtOAc) to afford the product **6a** as a yellow oil (0.1411 g, 52%) and **6b** as a yellow solid (0.0759 g, 28%).

### General Procedure 2 for the Diels–Alder Reaction

A 10 mL Schlenk flask equipped with a stir bar was evacuated and backfilled with N_2_ three times. Chiral diene (0.1 mmol, 1.0 equiv) was diluted in toluene (3.2 mL) and charged to the vessel *via* a syringe followed by dienophile (0.3 mmol, 3.0 equiv). The reaction mixture was stirred at 100 °C over 16–172 h. The solution was concentrated *in vacuo* and purified by flash column chromatography (3:1 cyclohexane:EtOAc) to afford the product. For dienophiles such as dimethylacetylene dicarboxylate and maleimide, 16 h was sufficient for the reaction to take place. For dienophiles such as nitrostyrene, reaction times of 172 h were necessary.

### Procedure for Gram-Scale Synthesis of (**17**)

A 25 mL Schlenk flask equipped with a stir bar was evacuated and backfilled with N_2_ three times. Chiral diene **2** (0.1950g, 1.0 mmol, 1.0 equiv) was diluted in toluene (0.1 M) and charged to the vessel *via* a syringe followed by dienophile (0.5375 g, 3.0 mmol, 3.0 equiv). The reaction mixture was stirred at 100 °C over 172 h. The solution was concentrated *in vacuo* and purified by flash column chromatography (3:1 Cyclohexane: EtOAc) to afford the product **17** as a yellow solid (0.1872 g, 50%).

### General Procedure 3 for the Hydrolysis of the Nitrostyrene Diels–Alder Product

Diels–Alder product (0.05 mmol, 1.0 equiv) was dissolved in 2 mL toluene and charged into an oven-dried 15 mL boiling tube equipped with a stir bar, followed by HCl (1.0 M, 14 equiv). The biphasic reaction mixture was stirred at 110 °C for 48 h. The aqueous phase was extracted with toluene (3 × 5 mL) and the organic layers were combined, dried over Na_2_SO_4_, and concentrated *in vacuo*. The crude hydrolyzed product was purified by flash column chromatography (3:1 cyclohexane:EtOAc) to afford the desired compound.

### Procedure for Hydrolysis of the Maleimide Diels–Alder Product (**66**)

Diels–Alder product (0.05 mmol, 1.0 equiv) was dissolved in 2 mL toluene and charged into an oven-dried 15 mL boiling tube equipped with a stir bar followed by HCl (1.0 M, 14 equiv). The biphasic reaction mixture was stirred at 110 °C for 48 h. The organic phase was extracted with H_2_O (3 × 5 mL) and the aqueous layers were combined and concentrated *in vacuo* to afford the desired compound without the need for further purification as an off-white solid (0.0067 g, 81%).

### Procedure for the Hydrolysis of the Ph-Substituted Maleimide Diels–Alder Products (**67** and **68**)

The Diels–Alder product (0.05 mmol, 1.0 equiv) was dissolved in 2 mL of toluene and charged into an oven-dried 15 mL boiling tube equipped with a stir bar followed by HCl (1.0 M, 14 equiv). The biphasic reaction mixture was stirred at 110 °C for 48 h. The aqueous phase was extracted with toluene (3 × 5 mL) and the organic layers were combined, dried over Na_2_SO_4_, and concentrated *in vacuo*. The crude hydrolyzed product was purified by trituration of the oxazolidinone byproduct with CDCl_3_ to afford the desired compound as a white solid without the need for further purification as a white solid (0.0085 g, 70%).

#### (*S*)-3-(Buta-1,3-dien-2-yl)-4-isopropyloxazolidin-2-one (**1**)

Pale yellow oil. Synthesized by GP1 (0.0272 g, 43%). R_f_ = 0.22 (cyclohexane:EtOAc, 3:1). ^1^H NMR (400 MHz, CDCl_3_): δ 6.29 (dd, *J* = 17.3, 10.8 Hz, 1H), 5.38 (d, *J* = 17.3 Hz, 2H), 5.24 (d, *J* = 10.9 Hz, 2H), 4.34 (t, *J* = 8.9 Hz, 1H), 4.16 (dd, *J* = 8.9, 5.4 Hz, 1H), 4.03 (ddd, *J* = 9.0, 5.3, 3.7 Hz, 1H), 2.01 (ddh, *J* = 10.6, 6.9, 3.4 Hz, 1H), 0.89 (d, *J* = 6.1 Hz, 3H), 0.87 (d, *J* = 7.2 Hz, 3H). ^13^C {^1^H} NMR (126 MHz, CDCl_3_): δ 156.7, 140.3, 132.8, 116.8, 113.4, 63.2, 60.6, 28.5, 17.9, 14.6. IR ν(cm^–1^): 2976, 2930, 1748, 1406, 1220. HRMS (ESI) *m*/*z*: [M + H]^+^ calculated for C_10_H_15_NO_2_, 182.1176; found, 182.1175.

#### (*S*)-3-(Buta-1,3-dien-2-yl)-4-(*tert*-butyl)oxazolidin-2-one (**2**)

Pale yellow oil. Synthesized by GP1 (0.0662 g, 97%). R_f_ = 0.33 (cyclohexane:EtOAc, 3:1). ^1^H NMR (400 MHz, CDCl_3_): δ 6.26 (dd, *J* = 17.4, 10.8 Hz, 1H), 5.42–5.33 (m, 3H), 5.30 (d, *J* = 10.8 Hz, 1H), 4.35 (t, *J* = 9.0 Hz, 1H), 4.24 (dd, *J* = 8.9, 4.8 Hz, 1H), 3.91 (dd, *J* = 8.9, 4.8 Hz, 1H), 0.94 (d, *J* = 3.2 Hz, 9H). ^13^C {^1^H} NMR (101 MHz, CDCl_3_): δ 157.4, 142.7, 133.0, 117.2, 115.2, 64.7, 64.6, 35.2, 25.5. IR ν(cm^–1^): 2960, 2873, 1744, 1399. HRMS (ESI) *m*/*z*: [M + H]^+^ calculated for C_11_H_17_NO_2_, 196.1332; found, 196.1330.

#### (*S*)-3-(Buta-1,3-dien-2-yl)-4-phenyloxazolidin-2-one (**3**)

Synthesized by GP1 (0.0196g, 26%). Described previously.^18,19^^1^H NMR (500 MHz, CDCl_3_): δ 7.40–7.34 (m, 3H), 7.30–7.27 (m, 2H), 6.29 (dd, *J* = 17.3, 10.8 Hz, 1H), 5.42 (d, *J* = 17.3 Hz, 1H), 5.22 (d, *J* = 10.8 Hz, 1H), 5.13–5.06 (m, 2H), 4.82 (s, 1H), 4.73 (t, *J* = 8.7 Hz, 1H), 4.24 (dd, *J* = 8.7, 6.8 Hz, 1H). ^13^C {^1^H} NMR (101 MHz, CDCl_3_): δ 156.2, 140.4, 137.9, 132.7, 129.3, 129.1, 126.9, 116.6, 111.6, 70.1, 61.3.

#### (*S*)-3-(Buta-1,3-dien-2-yl)-4-benzyloxazolidin-2-one (**4**)

Synthesized by GP1 (0.0136 g, 17%). Described previously.^18,19^^1^H NMR (400 MHz, CDCl_3_): δ 7.37–7.22 (m, 3H), 7.13 (d, *J* = 7.0 Hz, 2H), 6.40 (dd, *J* = 17.2, 10.7 Hz, 1H), 5.44 (d, *J* = 17.3 Hz, 1H), 5.40 (s, 1H), 5.30 (d, *J* = 10.8 Hz, 1H), 5.27 (s, 1H), 4.34–4.22 (m, 2H), 4.13 (d, *J* = 3.0 Hz, 1H), 3.17–3.08 (m, 1H), 2.65 (dd, *J* = 13.5, 9.6 Hz, 1H). ^13^C {^1^H} NMR (101 MHz, CDCl_3_): δ 156.2, 140.3, 135.5, 132.9, 129.2, 129.0, 127.3, 117.0, 113.5, 66.9, 57.9, 38.5.

#### (*S*,*Z*)-4-Isopropyl-3-(1-phenylbuta-1,3-dien-2-yl)oxazolidin-2-one (**5a**)

Yellow oil. Synthesized by GP1 (0.0481 g, 52%). R_f_ = 0.51 (cyclohexane:EtOAc, 3:1). Present as rotamers in solution, see SI Figures S1 & S2 for more detail. ^1^H NMR (400 MHz, CDCl_3_): δ 7.35 (m, 4H), 7.31–7.27 (m, 1H), 6.69 (s, 1H), 6.42 (dd, *J* = 17.3, 10.7 Hz, 1H), 5.43 (d, *J* = 17.3 Hz, 1H), 5.31 (d, *J* = 10.7 Hz, 1H), 4.24 (t, *J* = 8.9 Hz, 1H), 4.10 (dd, *J* = 8.9, 7.0 Hz, 1H), 3.61 (ddd, *J* = 8.9, 7.0, 4.8 Hz, 1H), 1.70 (dd, *J* = 6.8, 2.1 Hz, 1H), 0.73 (d, *J* = 6.8 Hz, 3H), 0.71 (d, *J* = 6.8 Hz, 3H). ^13^C {^1^H} NMR (101 MHz, CDCl_3_): δ 157.7, 134.9, 134.4, 128.8, 128.7, 128.5, 116.0, 65.1, 61.2, 29.9, 27.0, 18.8, 16.2. IR ν(cm^–1^): 2962, 2922, 1740, 1406, 1224, 698. HRMS (ESI) *m*/*z*: [M + H]^+^ calculated for C_16_H_19_NO_2_, 258.1489; found, 258.1488.

#### (*S*,*E*)-4-Isopropyl-3-(1-phenylbuta-1,3-dien-2-yl)oxazolidin-2-one (**5b**)

Yellow solid. Synthesized by GP1 (0.0231 g, 25%). R_f_ = 0.48 (cyclohexane:EtOAc, 3:1). Melting point: 119–123 °C. XRD sample prepared by slow evaporation of compound in CH_2_Cl_2_ with pentane antisolvent. ^1^H NMR (400 MHz, CDCl_3_): δ 7.34 (d, *J* = 2.7 Hz, 4H), 7.31–7.26 (m, 1H), 6.72 (s, 1H), 6.64 (dd, *J* = 17.4, 10.9 Hz, 1H), 5.42 (d, *J* = 17.4 Hz, 1H), 5.38 (d, *J* = 10.9 Hz, 1H), 4.40 (t, *J* = 8.9 Hz, 1H), 4.21 (dd, *J* = 8.9, 5.4 Hz, 1H), 4.10 (ddd, *J* = 9.0, 5.4, 3.8 Hz, 1H), 1.98 (ddp, *J* = 10.6, 6.9, 3.6 Hz, 1H), 0.99 (d, *J* = 6.9 Hz, 3H), 0.91 (d, *J* = 6.9 Hz, 3H). ^13^C {^1^H} NMR (126 MHz, CDCl_3_): δ 157.3, 135.1, 133.1, 131.5, 129.6, 129.0, 128.4, 127.9, 117.9, 63.5, 60.6, 29.1, 18.1, 14.9. IR ν(cm^–1^): 2959, 2921, 1741, 1213, 702. HRMS (ESI) *m*/*z*: [M + H]^+^ calculated for C_16_H_19_NO_2_, 258.1489; found, 258.1488.

#### (*S*,*Z*)-4-(*tert*-butyl)-3-(1-phenylbuta-1,3-dien-2-yl)oxazolidin-2-one (**6a**)

Orange oil. Synthesized by GP1 (0.0531 g, 56%). R_f_ = 0.54 (Cyclohexane:EtOAc, 3:1). Restricted rotation observable in ^1^H NMR at rt. See Supporting Information (S2) for ^1^H VT NMR studies. ^1^H NMR (400 MHz, CDCl_3_): δ 7.39–7.27 (m, 5H), 6.58 (s, 1H), 6.45 (dd, *J* = 17.2, 10.8 Hz, 1H), 5.62 (d, *J* = 17.2 Hz, 1H), 5.40 (d, *J* = 10.8 Hz, 1H), 4.12 (dd, *J* = 9.1, 5.1 Hz, 1H), 4.05 (t, *J* = 9.1 Hz, 1H), 3.24 (dd, *J* = 9.2, 5.1 Hz, 1H), 0.82 (s, 9H).^13^C {^1^H} NMR (126 MHz, *d-*DMSO): δ 158.1, 136.4, 135.5, 135.2, 134.87, 134.5, 133.9, 132.9, 131.2, 129.3, 129.0, 128.4, 128.3, 127.8, 116.4, 114.9, 64.7, 63.4, 34.1, 24.9. IR ν(cm^–1^): 2958, 2937, 1747, 1397, 703. HRMS (ESI) *m*/*z*: [M + H]^+^ calculated for C_17_H_21_NO_2_, 272.1645; found, 272.1644.

#### (*S*,*E*)-4-(*tert*-butyl)-3-(1-phenylbuta-1,3-dien-2-yl)oxazolidin-2-one (**6b**)

Orange solid. Synthesized by GP1 (0.0304 g, 32%). R_f_ = 0.45 (Cyclohexane:EtOAc, 3:1). Melting point: 115–119 °C. XRD sample prepared by slow evaporation of compound in CH_2_Cl_2_ with pentane antisolvent. ^1^H NMR (400 MHz, CDCl_3_): δ 7.37–7.27 (m, 5H), 6.78 (s, 1H), 6.62 (ddd, *J* = 17.6, 11.1, 0.6 Hz, 1H), 5.41 (t, *J* = 11.1, 1H), 5.37 (d, *J* = 17.6 Hz, 1H), 4.38 (t, *J* = 9.1 Hz, 1H), 4.28 (dd, *J* = 9.1, 5.0 Hz, 1H), 4.01 (dd, *J* = 9.1, 5.0 Hz, 1H), 0.98 (s, 9H). ^13^C {^1^H} NMR (101 MHz, CDCl_3_): δ 158.0, 137.1, 135.4, 135.0, 129.0, 128.5, 128.1, 128.1, 117.4, 114.9, 65.3, 64.3, 35.4, 25.5. IR ν(cm^–1^): 2958, 2937, 1747, 1397, 703. HRMS (ESI) *m*/*z*: [M + H]^+^ calculated for C_17_H_21_NO_2_, 272.1645; found, 272.1644.

#### (*S*,*Z*)-4-Phenyl-3-(1-phenylbuta-1,3-dien-2-yl)oxazolidin-2-one (**7a**)

Yellow solid. Synthesized by GP1 (0.0546 g, 54%). R_f_ = 0.33 (cyclohexane:EtOAc, 3:1). Melting point: 116–120 °C. XRD sample prepared by slow evaporation of compound in CH_2_Cl_2_ with pentane antisolvent. ^1^H NMR (400 MHz, CDCl_3_): δ 7.35 (ddd, *J* = 12.0, 7.7, 6.0 Hz, 3H), 7.25–7.21 (m, 2H), 7.21–7.12 (m, 2H), 7.00–6.93 (m, 3H), 6.53 (s, 1H), 6.23 (dd, *J* = 17.3, 10.7 Hz, 1H), 5.36 (d, *J* = 17.3 Hz, 1H), 5.20 (d, *J* = 10.7 Hz, 1H), 4.74 (t, *J* = 8.6 Hz, 1H), 4.59 (t, *J* = 8.6 Hz, 1H), 4.34 (t, *J* = 8.6 Hz, 1H). ^13^C {^1^H} NMR (101 MHz, CDCl_3_): δ 157.0, 136.1, 135.0, 133.9, 133.2, 129.1, 128.7, 128.7, 128.6, 128.4, 127.9, 115.8, 70.1, 60.4. IR ν(cm^–1^): 2999, 2914, 1748, 1052, 698. HRMS (ESI) *m*/*z*: [M + H]^+^ calculated for C_19_H_17_NO_2_, 292.1332; found, 292.1331.

#### (*S*,*E*)-4-Phenyl-3-(1-phenylbuta-1,3-dien-2-yl)oxazolidin-2-one (**7b**)

Synthesized by GP1 (0.0314 g, 31%). Described previously.^18,19^^1^H NMR (400 MHz, CDCl_3_): δ 7.44–7.34 (m, 5H), 7.31–7.16 (m, 3H), 7.15–7.06 (m, 2H), 6.50 (ddd, *J* = 17.4, 10.9, 0.7 Hz, 1H), 6.40 (s, 1H), 5.48 (d, *J* = 17.4 Hz, 1H), 5.36 (dt, *J* = 10.9, 1.3 Hz, 1H), 5.15 (dd, *J* = 8.8, 6.7 Hz, 1H), 4.79 (t, *J* = 8.8 Hz, 1H), 4.41 (dd, *J* = 8.8, 6.7 Hz, 1H). ^13^C {^1^H} NMR (101 MHz, CDCl_3_): δ 156.8, 137.9, 134.9, 132.8, 132.3, 129.4, 129.2, 129.0, 128.3, 127.9, 127.6, 117.6, 69.8, 61.3.

#### (*S*,*Z*)-4-Benzyl-3-(1-phenylbuta-1,3-dien-2-yl)oxazolidin-2-one (**8a**)

Orange oil. Synthesized by GP1 (0.0758 g, 71%). R_f_ = 0.23 (Cyclohexane:EtOAc, 4:1). ^1^H NMR (400 MHz, CDCl_3_): δ 7.43–7.35 (m, 4H), 7.34–7.28 (m, 1H), 7.24–7.17 (m, 3H), 6.89 (dd, *J* = 7.4, 1.7 Hz, 2H), 6.74 (s, 1H), 6.48 (dd, *J* = 17.2, 10.6 Hz, 1H), 5.42 (d, *J* = 17.2 Hz, 1H), 5.34 (d, *J* = 10.6 Hz, 1H), 4.20 (t, *J* = 8.6 Hz, 1H), 4.10 (dd, *J* = 8.6, 7.5 Hz, 1H), 3.97 (dtd, *J* = 11.5, 8.0, 3.9 Hz, 1H), 2.91 (dd, *J* = 13.4, 3.9 Hz, 1H), 2.42 (dd, *J* = 13.4, 10.5 Hz, 1H). ^13^C {^1^H} NMR (101 MHz, CDCl_3_): δ 156.9, 135.6, 134.7, 134.5, 133.2, 128.9, 128.9, 128.7, 128.7, 127.2, 115.8, 68.6, 57.5, 39.2. IR ν(cm^–1^): 3026, 2921, 1749, 1399, 699. HRMS (ESI) *m*/*z*: [M + H]^+^ calculated for C_20_H_19_NO_2_, 306.1489; found, 306.1486.

#### (*S*,*E*)-4-Benzyl-3-(1-phenylbuta-1,3-dien-2-yl)oxazolidin-2-one (**8b**)

Synthesized by GP1 (0.0096 g, 9%). Described previously.^18,19^^1^H NMR (500 MHz, CDCl_3_): δ 7.37 (d, *J* = 6.6 Hz, 4H), 7.28 (s, 3H), 7.28–7.22 (m, 1H), 7.14 (d, *J* = 7.2 Hz, 2H), 6.76 (s, 1H), 6.72 (dd, *J* = 17.6, 11.0 Hz, 1H), 5.47 (d, *J* = 17.6 Hz, 1H), 5.41 (d, *J* = 11.0 Hz, 1H), 4.40–4.35 (m, 1H), 4.35–4.31 (m, 1H), 4.18 (h, *J* = 5.3 Hz, 1H), 3.15 (dd, *J* = 13.5, 3.7 Hz, 1H), 2.73–2.67 (m, 1H). ^13^C {^1^H} NMR (126 MHz, CDCl_3_): δ 156.7, 135.5, 134.8, 132.7, 131.9, 129.6, 129.3, 129.0, 128.9, 128.4, 128.0, 127.2, 117.9, 67.2, 57.8, 39.1.

#### Dimethyl (*S*)-4-(4-(*iso*-Propyl)-2-oxooxazolidin-3-yl)cyclohexa-1,4-diene-1,2-dicarboxylate (**9**)

Colorless oil. Synthesized by GP2 (0.0712 g, 63%). R_f_ = 0.11 (cyclohexane:EtOAc, 2:1). ^1^H NMR (400 MHz, CDCl_3_): δ 5.64–5.57 (m, 1H), 4.27 (t, J = 8.9 Hz, 1H), 4.11 (dd, J = 8.9, 5.0 Hz, 1H), 4.03 (ddd, J = 8.9, 4.9, 3.6 Hz, 1H), 3.76 (s, 3H), 3.75 (s, 3H), 3.63–3.47 (m, 1H), 3.22–3.12 (m, 2H), 3.12–2.96 (m, 1H), 2.04 (dddt, J = 13.8, 10.4, 6.7, 3.6 Hz, 1H), 0.87 (d, J = 6.7 Hz, 3H), 0.85 (d, J = 6.7 Hz, 3H). ^13^C {^1^H} NMR (101 MHz, CDCl_3_): δ 168.1, 167.4, 155.9, 132.4, 131.0, 129.2, 114.3, 62.8, 59.7, 52.4, 28.4, 28.0, 17.8, 14.5. IR ν(cm^–1^): 2956, 2927, 1722, 1113, 1054. HRMS (ESI) *m*/*z*: [M + H]^+^ calculated for C_16_H_21_NO_6_, 324.1442; found, 324.1442.

#### Dimethyl (*S*)-4-(4-(*tert*-Butyl)-2-oxooxazolidin-3-yl)cyclohexa-1,4-diene-1,2-dicarboxylate (**10**)

Colorless oil. Synthesized from GP2 (0.0290 g, 58%). R_f_ = 0.15 (cyclohexane:EtOAc, 2:1). ^1^H NMR (400 MHz, CDCl_3_): δ 5.87–5.79 (m, 1H), 4.28 (t, *J* = 9.1 Hz, 1H), 4.19 (dd, *J* = 9.1, 4.2 Hz, 1H), 3.82 (dd, *J* = 9.1, 4.2 Hz, 1H), 3.78 (s, 3H), 3.77 (s, 3H), 3.57–3.44 (m, 1H), 3.20 (qd, *J* = 8.7, 3.7 Hz, 2H), 2.99–2.85 (m, 1H), 0.93 (s, 9H). ^13^C {^1^H} NMR (101 MHz, CDCl_3_): δ 168.3, 167.3, 156.9, 133.3, 131.1, 130.4, 117.7, 64.5, 63.4, 52.5, 35.7, 28.7, 28.5, 25.5. IR ν(cm^–1^): 2957, 1720, 1605, 1397, 1192, 1124. HRMS (ESI) *m*/*z*: [M + H]^+^ calculated for C_17_H_23_NO_6_, 338.1598; found, 338.1599.

#### Dimethyl (*S*)-4-(4-Phenyl-2-oxooxazolidin-3-yl)cyclohexa-1,4-diene-1,2-dicarboxylate (**11**)

Colorless oil. Synthesized by GP2 (0.0472 g, 55%). R_f_ = 0.18 (cyclohexane:EtOAc, 2:1). ^1^H NMR (400 MHz, CDCl_3_): δ 7.37 (m, 3H), 7.26 (dd, *J* = 7.9, 1.4 Hz, 2H), 5.31 (dt, *J* = 3.7, 2.5 Hz, 1H), 5.09 (dd, *J* = 8.7, 6.4 Hz, 1H), 4.68 (t, *J* = 8.7 Hz, 1H), 4.15–4.08 (m, 1H), 3.76 (s, 3H), 3.74 (s, 3H), 3.60–3.47 (m, 1H), 3.29–3.18 (m, 1H), 3.01 (td, *J* = 8.4, 3.7 Hz, 2H). ^13^C {^1^H} NMR (126 MHz, CDCl_3_): δ 168.1, 167.5, 155.8, 138.1, 132.1, 131.1, 129.6, 129.2, 126.3, 112.0, 70.1, 60.6, 52.4, 28.4, 28.1. IR ν(cm^–1^): 2959, 2922, 1742, 1548, 1400, 700. HRMS (ESI) *m*/*z*: [M + NH_4_]^+^ calculated for C_19_H_19_NO_6_, 375.1551; found, 375.1548.

#### Dimethyl (*S*)-4-(4-Benzyl-2-oxooxazolidin-3-yl)cyclohexa-1,4-diene-1,2-dicarboxylate (**12**)

Colorless oil. Synthesized by GP2 (0.0240 g, 43%). R_f_ = 0.15 (cyclohexane:EtOAc, 2:1). ^1^H NMR (400 MHz, CDCl_3_): δ 7.37–7.27 (m, 3H), 7.14 (d, *J* = 6.9 Hz, 2H), 5.65 (s, 1H), 4.31 (dt, *J* = 8.6, 4.2 Hz, 1H), 4.28–4.21 (m, 1H), 4.11 (dd, *J* = 8.6, 4.2 Hz, 1H), 3.84 (s, 3H), 3.80 (s, 3H), 3.70–3.50 (m, 1H), 3.23 (dq, *J* = 13.7, 4.0 Hz, 2H), 3.13 (dd, *J* = 14.1, 4.0 Hz, 2H), 2.72 (dd, *J* = 13.7, 8.9 Hz, 1H). ^13^C {^1^H} NMR (126 MHz, CDCl_3_): δ 168.0, 167.4, 155.4, 135.1, 132.0, 131.2, 129.1, 129.0, 127.4, 113.3, 66.5, 56.8, 52.4, 38.2, 28.6, 28.3. IR ν(cm^–1^): 2949, 2923, 1718, 1395, 1191, 701. HRMS (ESI) *m*/*z*: [M + H]^+^ calculated for C_20_H_21_NO_6_, 372.1442; found, 372.1440.

#### (*S*)-4-*iso*-Propyl-3-((1*S*,6*R*)-6-nitro-1,2,5,6-tetrahydro-[1,1′-biphenyl]-3-yl)oxazolidin-2-one (**13**)

Yellow oil. Synthesized by GP2 (0.0228 g, 69%). R_f_ = 0.09 (cyclohexane:EtOAc, 3:1). Inseparable diastereomers, dr 75:25. *Major diastereomer*:^1^H NMR (400 MHz, CDCl_3_): δ 7.33–7.27 (m, 2H), 7.23–7.16 (m, 3H), 5.73–5.68 (m, 1H), 4.99–4.86 (m, 1H), 4.31–4.25 (m, 1H), 4.16–4.10 (m, 1H), 3.99 (ddd, *J* = 8.8, 5.0, 3.6 Hz, 1H), 3.54 (qd, *J* = 10.6, 5.8 Hz, 1H), 3.04–2.90 (m, 3H), 2.46–2.32 (m, 1H), 2.06 (dq, *J* = 10.3, 3.3 Hz, 1H), 0.92 (d, *J* = 3.3 Hz, 3H), 0.90 (d, *J* = 1.7 Hz, 3H). ^13^C {^1^H} NMR (126 MHz, CDCl_3_): δ 156.1, 137.4, 133.9, 132.2, 129.3, 129.2 128.9, 115.9, 113.1, 86.3, 62.9, 60.1, 43.5, 32.6, 29.9, 28.6, 27.0, 17.8, 14.7. IR ν(cm^–1^): 2964, 1749, 1545, 1409, 704. HRMS (ESI) *m*/*z*: [M + H]^+^ calculated for C_18_H_22_N_2_O_4_, 331.1652; found, 331.1652.

#### (*S*)-4-*iso*-Propyl-3-((1*S*,6*R*)-4′-methoxy-6-nitro-1,2,5,6-tetrahydro-[1,1′-biphenyl]-3-yl)oxazolidin-2-one (**14**)

Yellow oil. Synthesized by GP2 (0.0177 g, 49%). R_f_ = 0.06 (cyclohexane:EtOAc, 3:1). Inseparable diastereomers, dr 75:25. *Major diastereomer*:^1^H NMR (400 MHz, CDCl_3_): δ 7.18–7.14 (m, 2H), 6.89–6.80 (m, 2H), 5.76–5.70 (m, 1H), 4.94–4.86 (m, 1H), 4.30–4.23 (m, 1H), 4.14–4.08 (m, 1H), 3.98 (ddd, *J* = 8.8, 4.9, 3.7 Hz, 1H), 3.77 (s, 3H), 3.50 (qd, *J* = 10.0, 5.6 Hz, 1H), 2.98–2.87 (m, 3H), 2.40 (ddd, *J* = 16.5, 10.6, 2.2 Hz, 1H), 2.05 (ddt, *J* = 10.6, 6.9, 3.5 Hz, 1H), 0.92 (d, *J* = 3.5 Hz, 3H), 0.90 (d, *J* = 3.5 Hz, 3H). ^13^C {^1^H} NMR (101 MHz, CDCl_3_): δ 159.3, 156.1, 132.2, 130.7, 128.5, 116.2, 114.5, 86.7, 62.9, 60.0, 55.4, 43.3, 32.7, 29.9, 28.7, 17.8, 14.7. IR ν(cm^–1^): 2960, 2919, 2850, 1740, 1546, 1245. HRMS (ESI) *m*/*z*: [M + H]^+^ calculated for C_19_H_24_N_2_O_5_, 361.1758; found, 361.1761.

#### (*S*)-3-((1*S*,6*R*)-4′-Chloro-6-nitro-1,2,5,6-tetrahydro-[1,1′-biphenyl]-3-yl)-4-iso-propyloxazolidin-2-one (**15**)

Yellow oil. Synthesized by GP2 (0.0248 g, 68%). R_f_ = 0.09 (cyclohexane:EtOAc, 3:1). Inseparable diastereomers, dr 68:32. *Major diastereomer*:^1^H NMR (400 MHz, CDCl_3_): δ 7.30 (d, *J* = 8.4 Hz, 2H), 7.19 (d, *J* = 8.4 Hz, 2H), 5.71 (s, 1H), 4.92 (ddd, *J* = 10.8, 8.4, 6.9 Hz, 1H), 4.33–4.24 (m, 1H), 4.13 (ddd, *J* = 8.9, 5.1, 3.1 Hz, 1H), 4.03–3.95 (m, 1H), 3.56 (td, *J* = 10.8, 5.7 Hz, 1H), 3.05–2.88 (m, 3H), 2.40 (ddd, *J* = 17.6, 10.4, 2.6 Hz, 1H), 2.10–1.99 (m, 1H), 0.93 (d, *J* = 3.4 Hz, 3H), 0.91 (d, *J* = 3.4 Hz, 3H). ^13^C {^1^H} NMR (101 MHz, CDCl_3_): δ 156.1, 137.4, 132.2, 129.3, 128.9, 115.8, 86.3, 62.9, 60.1, 43.6, 32.6, 29.8, 28.6, 27.0, 17.8, 14.7. IR ν(cm^–1^): 2960, 2919, 2851, 1742, 1546, 1091. HRMS (ESI) *m*/*z*: [M + H]^+^ calculated for C_18_H_21_ClN_2_O_4_, 365.1263; found, 365.1265.

#### (*S*)-4-(*tert*-Butyl)-3-((1*S*,6*R*)-6-nitro-1,2,5,6-tetrahydro-[1,1′-biphenyl]-3-yl)oxazolidin-2-one (**16**)

Yellow oil. Synthesized by GP2 (0.0172 g, 50%). R_f_ = 0.13 (cyclohexane:EtOAc, 3:1). Inseparable diastereomers, dr 92:8. *Major diastereomer*:^1^H NMR (500 MHz, CDCl_3_): δ 7.33 (dd, *J* = 7.9, 6.4 Hz, 2H), 7.30–7.22 (m, 3H), 5.92 (s, 1H), 5.00 (dt, *J* = 10.8, 7.6 Hz, 1H), 4.25 (t, *J* = 9.0 Hz, 1H), 4.18 (dd, *J* = 9.0, 4.7 Hz, 1H), 3.78 (dd, *J* = 9.0, 4.7 Hz, 1H), 3.53 (td, *J* = 10.8, 5.6 Hz, 1H), 2.94 (dd, *J* = 6.0, 2.9 Hz, 2H), 2.92–2.82 (m, 1H), 2.35 (ddd, *J* = 17.5, 10.8, 2.4 Hz, 1H), 0.97 (s, 9H). ^13^C {^1^H} NMR (101 MHz, CDCl_3_): δ 157.0, 138.9, 133.8, 129.2, 128.2, 127.5, 119.3, 86.4, 64.3, 63.3, 44.1, 35.5, 32.9, 30.2, 25.3. IR ν(cm^–1^): 2960, 2922, 2853, 1743, 1548, 1402, 700. HRMS (ESI) *m*/*z*: [M + H]^+^ calculated for C_19_H_24_N_2_O_4_, 345.1809; found, 345.1812.

#### (*S*)-4-(*tert*-Butyl)-3-((1*S*,6*R*)-4′-methoxy-6-nitro-1,2,5,6-tetrahydro-[1,1′-biphenyl]-3-yl)oxazolidin-2-one (**17**)

Yellow oil. Synthesized by GP2 (0.0195 g, 52%). R_f_ = 0.06 (cyclohexane:EtOAc, 3:1). Inseparable diastereomers, dr 90:10. *Major diastereomer*:^1^H NMR (400 MHz, CDCl_3_): δ 7.16 (dt, J = 7.3, 2.5 Hz, 2H), 6.90–6.82 (m, 2H), 5.91 (s, 1H), 4.93 (dt, J = 10.8, 7.6 Hz, 1H), 4.25 (t, J = 9.0 Hz, 1H), 4.18 (dd, J = 9.1, 4.7 Hz, 1H), 3.79 (d, J = 4.5 Hz, 1H), 3.78 (s, 3H), 3.48 (td, J = 10.7, 5.6 Hz, 1H), 2.97–2.90 (m, 2H), 2.84 (dd, J = 17.7, 4.8 Hz, 1H), 2.39–2.25 (m, 1H), 0.97 (s, 9H). ^13^C {^1^H} NMR (101 MHz, CDCl_3_): δ 159.3, 157.0, 133.8, 130.7, 128.5, 119.3, 114.5, 86.7, 64.3, 63.2, 55.4, 43.3, 35.5, 32.9, 30.1, 25.3. IR ν(cm^–1^): 2961, 2915, 2872, 1744, 1548, 1247, 731. HRMS (ESI) *m*/*z*: [M + H]^+^ calculated for C_20_H_26_N_2_O_5_, 375.1914; found, 375.1916.

#### (*S*)-4-(*tert*-Butyl)-3-((1S,6R)-4′-chloro-6-nitro-1,2,5,6-tetrahydro-[1,1′-biphenyl]-3-yl)oxazolidin-2-one (**18**)

Yellow solid. Synthesized by GP2 (0.0140 g, 37%). R_f_ = 0.06 (cyclohexane:EtOAc, 3:1). Inseparable diastereomers, dr 92:8. The XRD sample prepared by slow evaporation of compound in CH_2_Cl_2_ with pentane antisolvent. *Major diastereomer*:^1^H NMR (400 MHz, CDCl_3_): δ 7.35–7.28 (m, 2H), 7.23–7.14 (m, 2H), 5.90 (s, 1H), 4.94 (dt, *J* = 10.8, 7.6 Hz, 1H), 4.26 (t, *J* = 9.0 Hz, 1H), 4.18 (dd, *J* = 9.0, 4.7 Hz, 1H), 3.79 (dt, *J* = 9.0, 4.7 Hz, 1H), 3.52 (td, *J* = 10.7, 5.5 Hz, 1H), 2.93 (dd, *J* = 7.4, 4.4 Hz, 2H), 2.91–2.82 (m, 1H), 2.30 (ddd, *J* = 17.6, 10.8, 2.2 Hz, 1H), 0.97 (s, 9H). ^13^C {^1^H} NMR (101 MHz, CDCl_3_): δ 157.0, 137.3, 134.1, 133.7, 129.4, 128.9, 119.4, 86.2, 64.4, 63.4, 43.5, 35.5, 32.8, 30.1, 29.8, 27.1, 25.4. IR ν(cm^–1^): 2958, 2920, 2852, 1750, 1551, 1197. HRMS (ESI) *m*/*z*: [M + H]^+^ calculated for C_19_H_23_ClN_2_O_4_, 379.1419; found, 379.1421.

#### (*S*)-3-((1*S*,6*R*)-6-Nitro-1,2,5,6-tetrahydro-[1,1′-biphenyl]-3-yl)-4-phenyloxazolidin-2-one (**19**)

Yellow oil. Synthesized by GP2 (0.0171 g, 47%). R_f_ = 0.12 (cyclohexane:EtOAc, 3:1). Regiomeric isomers inseparable, rr = 85:15. Major regioisomer dr = 80:20, minor regioisomer dr = 50:50. *Major diastereomer*: ^1^H NMR (400 MHz, CDCl_3_): δ 7.43 (ddd, J = 10.6, 4.0, 2.0 Hz, 3H), 7.28 (dt, J = 5.4, 1.7 Hz, 4H), 7.17–7.12 (m, 2H), 7.02–6.97 (m, 1H), 5.39–5.32 (m, 1H), 5.14–5.02 (m, 1H), 4.84–4.74 (m, 1H), 4.68 (dt, J = 12.5, 8.7 Hz, 1H), 4.14 (td, J = 8.9, 6.3 Hz, 1H), 3.42 (td, J = 10.6, 5.8 Hz, 1H), 3.08–2.97 (m, 1H), 2.88–2.61 (m, 2H), 2.61–2.47 (m, 1H). ^13^C {^1^H} NMR (126 MHz, CDCl_3_): δ 155.7, 138.9, 132.9, 129.7, 129.3, 129.1, 127.9, 127.5, 126.3, 112.8, 86.2, 70.1, 70.0, 43.9, 33.1, 29.7. IR ν(cm^–1^): 2917, 1747, 1545, 1395, 699. HRMS (ESI) *m*/*z*: [M + H]^+^ calculated for C_21_H_20_N_2_O_4_, 365.1496; found, 365.1499.

#### (*S*)-3-((1*S*,6*R*)-4′-Methoxy-6-nitro-1,2,5,6-tetrahydro-[1,1′-biphenyl]-3-yl)-4-phenyloxazolidin-2-one (**20**)

Yellow oil. Synthesized by GP2 (0.0245 g, 62%). R_f_ = 0.12 (cyclohexane:EtOAc, 3:1). Inseparable diastereomers, 87:13 dr. *Major diastereomer*:^1^H NMR (400 MHz, CDCl_3_): δ 7.43 (m, 4H), 7.35–7.26 (m, 1H), 7.09–7.03 (m, 2H), 6.83–6.77 (m, 2H), 5.36 (d, *J* = 2.8 Hz, 1H), 5.04 (dd, *J* = 8.7, 6.0 Hz, 1H), 4.79–4.69 (m, 1H), 4.66 (t, *J* = 8.7 Hz, 1H), 4.13 (dd, *J* = 8.7, 5.9 Hz, 1H), 3.76 (s, 3H), 3.35 (td, *J* = 10.7, 5.8 Hz, 1H), 3.04–2.94 (m, 1H), 2.74 (d, *J* = 9.7 Hz, 1H), 2.70–2.60 (m, 1H), 2.57–2.44 (m, 1H). ^13^C {^1^H} NMR (101 MHz, CDCl_3_): δ 159.2, 155.7, 138.2, 132.9, 130.8, 129.5, 128.5, 126.3, 114.4, 86.5, 70.0, 60.9, 55.3, 43.2, 33.0, 29.6. IR ν(cm^–1^): 2963, 2922, 1740, 1546, 1407. HRMS (ESI) *m*/*z*: [M + H]^+^ calculated for C_22_H_22_N_2_O_5_, 395.1601; found, 395.1601.

#### (*S*)-3-((1*S*,6*R*)-4′-Chloro-6-nitro-1,2,5,6-tetrahydro-[1,1′-biphenyl]-3-yl)-4-phenyloxazolidin-2-one (**21**)

Yellow oil. Synthesized by GP2 (0.0239 g, 60%). R_f_ = 0.11 (cyclohexane:EtOAc, 3:1). Isolated as a single diastereomer. *Major diastereomer*:^1^H NMR (500 MHz, CDCl_3_): δ 7.49–7.46 (m, 3H), 7.29 (dd, *J* = 7.3, 2.1 Hz, 2H), 7.08–7.01 (m, 2H), 6.47–6.40 (m, 2H), 5.20–5.13 (m, 1H), 4.96 (s, 1H), 4.69 (t, *J* = 8.8 Hz, 1H), 4.27–4.19 (m, 1H), 4.14 (dd, *J* = 8.7, 7.4 Hz, 1H), 4.04 (dq, *J* = 8.2, 2.6 Hz, 1H), 3.44–3.34 (m, 1H), 2.42 (dddd, *J* = 15.6, 7.7, 5.8, 3.6 Hz, 1H), 2.33 (dtd, *J* = 13.1, 10.5, 5.5 Hz, 1H), 2.24 (ddt, *J* = 13.2, 6.0, 3.6 Hz, 1H). ^13^C {^1^H} NMR (126 MHz, CDCl_3_): δ 155.6, 138.6, 137.8, 134.3, 133.6, 129.8, 129.3, 129.1, 129.0, 126.5, 115.0, 88.6, 69.9, 61.2, 44.4, 26.9, 24.7. IR ν(cm^–1^): 2987, 2924, 2908, 1747, 1546, 1394, 1050. HRMS (ESI) *m*/*z*: [M + H]^+^ calculated for C_21_H_19_ClN_2_O_4_, 399.1106; found, 399.1106.

#### (*S*)-4-Benzyl-3-((1*S*,6*R*)-6-nitro-1,2,5,6-tetrahydro-[1,1′-biphenyl]-3-yl)oxazolidin-2-one (**22**)

Yellow oil. Synthesized by GP2 (0.0185 g, 49%). R_f_ = 0.09 (cyclohexane:EtOAc, 3:1). Regiomeric isomers, rr = 70:30. Major regioisomer dr = 70:30, minor regioisomer dr = 70:30. Isolated as a single diastereomer. ^1^H NMR (400 MHz, CDCl_3_): δ 7.43–7.27 (m, 5H), 7.15 (dd, *J* = 8.1, 6.3 Hz, 4H), 5.43 (s, 1H), 4.57 (td, *J* = 8.1, 5.4 Hz, 1H), 4.36 (dq, *J* = 8.1, 3.9 Hz, 1H), 4.29 (d, *J* = 8.1 Hz, 1H), 4.26–4.23 (m, 1H), 4.13 (dd, *J* = 8.7, 4.9 Hz, 1H), 3.17 (dd, *J* = 13.8, 4.1 Hz, 1H), 3.12–2.98 (m, 1H), 2.79 (dd, *J* = 13.8, 8.6 Hz, 1H), 2.50 (dd, *J* = 17.8, 4.7 Hz, 1H), 2.37 (dt, *J* = 7.9, 4.7 Hz, 2H). ^13^C {^1^H} NMR (101 MHz, CDCl_3_): δ 155.3, 138.4, 135.3, 134.6, 134.1, 129.5, 129.4, 129.23, 129.19, 127.6, 115.3, 87.9, 66.7, 56.9, 44.7, 38.2, 26.9, 25.2. IR ν(cm^–1^): 2928, 1753, 1537, 1399, 696. HRMS (ESI) *m*/*z*: [M + H]^+^ calculated for C_22_H_22_N_2_O_4_, 379.1652; found, 379.1650.

#### (*S*)-4-Benzyl-3-((1*S*,6*R*)-4′-methoxy-6-nitro-1,2,5,6-tetrahydro-[1,1′-biphenyl]-3-yl)oxazolidin-2-one (**23**)

Yellow solid. Synthesized by GP2 (0.0241g, 59%). R_f_ = 0.12 (cyclohexane:EtOAc, 3:1). Regiomeric isomers inseparable, rr = 91:9. Major regioisomer dr = 65:35, minor regioisomer dr = 63:17. *Major diastereomer:*^1^H NMR (500 MHz, CDCl_3_): δ 7.40–7.31 (m, 3H), 7.21–7.16 (m, 2H), 7.12 (d, *J* = 8.6 Hz, 2H), 6.85 (d, *J* = 8.6 Hz, 2H), 5.75–5.70 (m, 1H), 4.82 (ddd, *J* = 11.0, 8.8, 6.5 Hz, 1H), 4.35–4.27 (m, 2H), 4.13–4.05 (m, 1H), 3.78 (s, 3H), 3.26 (td, *J* = 10.8, 5.7 Hz, 1H), 3.14–3.07 (m, 1H), 2.87–2.77 (m, 4H), 2.36 (tdd, *J* = 13.3, 10.4, 4.5 Hz, 1H). ^13^C {^1^H} NMR (126 MHz, CDCl_3_): δ 159.3, 155.7, 135.6, 132.5, 129.2, 129.1, 128.6, 127.6, 114.4, 86.7, 66.9, 56.9, 55.4, 43.4, 39.6, 33.0, 29.9. IR ν(cm^–1^): 2926, 1754, 1737, 1398, 1247, 1179, 1028, 742, 700. HRMS (ESI) *m*/*z*: [M + H]^+^ calculated for C_23_H_24_N_2_O_5_, 409.1758; found, 409.1756.

#### (*S*)-4-Benzyl-3-((1*S*,6*R*)-4′-chloro-6-nitro-1,2,5,6-tetrahydro-[1,1′-biphenyl]-3-yl)oxazolidin-2-one (**24**)

Yellow oil. Synthesized by GP2 (0.0183 g, 45%). R_f_ = 0.10 (cyclohexane:EtOAc, 3:1). *Major diastereomer:*^1^H NMR (400 MHz, CDCl_3_): δ 7.39–7.27 (m, 5H), 7.22 (dd, J = 8.1, 1.4 Hz, 2H), 7.16–7.12 (m, 2H), 5.49 (d, J = 1.2 Hz, 1H), 4.69–4.58 (m, 1H), 4.42–4.31 (m, 1H), 4.32–4.24 (m, 2H), 4.17–4.09 (m, 1H), 3.18 (dt, J = 13.9, 4.6 Hz, 1H), 3.12–3.02 (m, 1H), 2.78 (dd, J = 13.8, 8.7 Hz, 1H), 2.57–2.44 (m, 1H), 2.38 (m, 2H). ^13^C {^1^H} NMR (101 MHz, CDCl_3_): δ 155.4, 139.9, 135.4, 134.3, 129.3, 129.23, 129.17, 128.2, 127.6, 115.9, 88.0, 66.7, 57.1, 45.3, 38.2, 26.8, 25.1. IR ν(cm^–1^): 2920, 2853, 1738, 1397, 740, 699. HRMS (ESI) *m*/*z*: [M + H]^+^ calculated for C_22_H_21_ClN_2_O_4_, 413.1263; found, 413.1260.

#### (*S*)-4-(*tert*-Butyl)-3-((1*S*,6*R*)-4′-methyl-6-nitro-1,2,5,6-tetrahydro-[1,1′-biphenyl]-3-yl)oxazolidin-2-one (**25**)

Pale yellow solid. Synthesized by GP2 (0.0147 g, 41%). R_f_ = 0.06 (cyclohexane:EtOAc, 3:1). Melting point: 111–116 °C. Inseparable diastereomers, dr 90:10. *Major diastereomer:*^1^H NMR (500 MHz, CDCl_3_): δ 7.14 (s, 4H), 5.91 (s, 1H), 4.97 (dt, *J* = 10.9, 7.8 Hz, 1H), 4.24 (t, *J* = 9.1 Hz, 1H), 4.18 (dd, *J* = 9.1, 4.6 Hz, 1H), 3.78 (dd, *J* = 9.1, 4.6 Hz, 1H), 3.49 (td, *J* = 10.8, 5.6 Hz, 1H), 2.97–2.90 (m, 2H), 2.85 (dd, *J* = 17.7, 5.6 Hz, 1H), 2.35 (dd, *J* = 10.8, 2.6 Hz, 1H), 2.31 (s, 3H), 0.97 (s, 9H). ^13^C {^1^H} NMR (126 MHz, CDCl_3_): δ 156.9, 137.9, 135.8, 133.8, 129.8, 127.3, 119.2, 86.5, 64.3, 63.2, 43.8, 35.5, 32.9, 30.2, 25.3, 21.2. IR ν(cm^–1^): 2959, 2919, 1749, 1548, 1402. HRMS (ESI) *m*/*z*: [M + H]^+^ calculated for C_20_H_26_N_2_O_4_, 359.1965; found, 359.1966.

#### (*S*)-4-(*tert*-Butyl)-3-((1*S*,6*R*)-4′-hydroxy-6-nitro-1,2,5,6-tetrahydro-[1,1′-biphenyl]-3-yl)oxazolidin-2-one (**26**)

Yellow oil. Synthesized by GP2 (0.0213 g, 59%). R_f_ = 0.38 (cyclohexane:EtOAc, 1:1). Inseparable diastereomers, dr 92:8. *Major diastereomer*:^1^H NMR (400 MHz, CDCl_3_): δ 7.04 (d, *J* = 8.5 Hz, 2H), 6.78 (d, *J* = 8.4 Hz, 2H), 5.88 (s, 1H), 4.87 (dt, *J* = 10.7, 7.6 Hz, 1H), 4.25 (t, *J* = 9.1 Hz, 1H), 4.21–4.15 (m, 1H), 3.78 (dd, *J* = 8.9, 4.8 Hz, 1H), 3.41 (td, *J* = 10.6, 5.6 Hz, 1H), 2.88 (m, 2H), 2.79 (dd, *J* = 17.6, 5.5 Hz, 1H), 2.43–2.23 (m, 1H), 0.95 (s, 9H). ^13^C {^1^H} NMR (126 MHz, CDCl_3_): δ 157.4, 156.4, 133.7, 129.9, 129.2, 128.6, 119.8, 116.1, 86.7, 64.5, 63.2, 43.3, 35.4, 32.8, 30.1, 29.8, 25.3. IR ν(cm^–1^): 3666, 3157, 2967, 1740, 1547, 1024. HRMS (ESI) *m*/*z*: [M + H]^+^ calculated for C_19_H_24_N_2_O_5_, 361.1758; found, 361.1757.

#### (*S*)-4-(*tert*-Butyl)-3-((1*S*,6*R*)-4′-fluoro-6-nitro-1,2,5,6-tetrahydro-[1,1′-biphenyl]-3-yl)oxazolidin-2-one (**27**)

Yellow oil. Synthesized by GP2 (0.0134 g, 37%). R_f_ = 0.10 (cyclohexane:EtOAc, 3:1). Inseparable diastereomers, dr 90:10. The XRD sample prepared by slow evaporation of compound in CH_2_Cl_2_ with pentane antisolvent. *Major diastereomer:*^1^H NMR (500 MHz, CDCl_3_): δ 7.22 (dd, *J* = 8.6, 5.3 Hz, 2H), 7.01 (t, *J* = 8.6 Hz, 2H), 5.90 (s, 1H), 4.93 (dt, *J* = 10.8, 7.6 Hz, 1H), 4.25 (t, *J* = 9.2 Hz, 1H), 4.17 (dd, *J* = 9.2, 4.7 Hz, 1H), 3.79 (dd, *J* = 9.2, 4.7 Hz, 1H), 3.52 (td, *J* = 10.8, 5.6 Hz, 1H), 2.95–2.91 (m, 2H), 2.88 (dd, *J* = 17.8, 5.6 Hz, 1H), 2.35–2.28 (m, 1H), 0.96 (s, 9H). ^13^C {^1^H} NMR (101 MHz, CDCl_3_): δ 163.7, 161.2, 157.1, 134.6, 133.8, 119.4, 116.3, 116.0, 86.4, 64.4, 63.4, 43.4, 35.5, 32.9, 30.1, 25.3. IR ν(cm^–1^): 2960, 2918, 1749, 1549, 1406, 838. HRMS (ESI) *m*/*z*: [M + H]^+^ calculated for C_19_H_23_FN_2_O_4_, 363.1715; found, 363.1715.

#### (*S*)-4-(*tert*-Butyl)-3-((1*S*,6*R*)-2′-chloro-6-nitro-1,2,5,6-tetrahydro-[1,1′-biphenyl]-3-yl)oxazolidin-2-one (**28**)

Yellow solid. Synthesized by GP2 (0.0212 g, 56%). R_f_ = 0.11 (cyclohexane:EtOAc, 3:1). Melting point: 170–176 °C. Inseparable diastereomers, dr 91:9. XRD sample prepared by slow evaporation of compound in CH_2_Cl_2_ with pentane antisolvent. *Major diastereomer:*^1^H NMR (500 MHz, CDCl_3_): δ 7.38 (dd, *J* = 7.9, 1.2 Hz, 1H), 7.34 (dd, *J* = 7.7, 1.5 Hz, 1H), 7.27 (td, *J* = 7.3, 1.1 Hz, 1H), 7.21 (td, *J* = 7.6, 1.6 Hz, 1H), 5.94 (s, 1H), 5.18 (q, *J* = 7.3 Hz, 1H), 4.23 (t, *J* = 9.0 Hz, 2H), 4.18 (dd, *J* = 9.1, 4.8 Hz, 1H), 3.84–3.73 (m, 1H), 2.95 (dq, *J* = 6.9, 2.9 Hz, 2H), 2.89 (ddd, *J* = 11.3, 4.9, 0.9 Hz, 1H), 2.17 (s, 1H), 0.98 (s, 9H). ^13^C {^1^H} NMR (126 MHz, CDCl_3_): δ 157.1, 136.6, 133.8, 133.3, 130.4, 129.1, 127.9, 119.8, 83.7, 64.4, 62.9, 35.5, 31.2, 29.8, 25.3. IR ν(cm^–1^): 2959, 2922, 2871, 1752, 1549, 1397, 765. HRMS (ESI) *m*/*z*: [M + H]^+^ calculated for C_19_H_23_ClN_2_O_4_, 379.1419; found, 379.1419.

#### (*S*)-3-((1*S*,6*R*)-3′-Bromo-6-nitro-1,2,5,6-tetrahydro-[1,1′-biphenyl]-3-yl)-4-(*tert*-butyl)oxazolidin-2-one (**29**)

Yellow oil. Synthesized by GP2 (0.0258 g, 61%). R_f_ = 0.10 (cyclohexane:EtOAc, 3:1). Inseparable diastereomers, dr 91:9. *Major diastereomer*:^1^H NMR (400 MHz, CDCl_3_): δ 7.41 (dt, *J* = 8.0, 1.7 Hz, 3H), 7.23–7.14 (m, 1H), 5.91 (s, 1H), 4.96 (ddd, *J* = 10.8, 8.8, 6.5 Hz, 1H), 4.26 (t, *J* = 9.0 Hz, 1H), 4.18 (dd, *J* = 9.2, 4.7 Hz, 1H), 3.79 (dd, *J* = 8.9, 4.7 Hz, 1H), 3.51 (td, *J* = 10.8, 5.5 Hz, 1H), 2.99–2.91 (m, 2H), 2.87 (d, *J* = 5.4 Hz, 1H), 2.31 (dd, *J* = 14.1, 11.1 Hz, 1H), 0.97 (s, 9H). ^13^C {^1^H} NMR (101 MHz, CDCl_3_): δ 156.9, 141.2, 133.6, 131.3, 130.8, 126.0, 123.1, 119.2, 85.9, 64.4, 63.3, 43.7, 35.5, 32.8, 30.2, 25.4. IR ν(cm^–1^): 2960, 2922, 1742, 1547, 1400. HRMS (ESI) *m*/*z*: [M + H]^+^ calculated for C_19_H_23_BrN_2_O_4_, 423.0914; found, 423.0914.

#### (*S*)-4-(*tert*-Butyl)-3-((1*S*,6*R*)-3′,4′-dimethoxy-6-nitro-1,2,5,6-tetrahydro-[1,1′-biphenyl]-3-yl)oxazolidin-2-one (**30**)

Yellow oil. Synthesized by GP2 (0.0239 g, 59%). R_f_ = 0.03 (cyclohexane:EtOAc, 3:1). Inseparable diastereomers, dr 87:13. *Major diastereomer*:^1^H NMR (400 MHz, CDCl_3_): δ 6.81 (d, *J* = 2.6 Hz, 2H), 6.75 (s, 1H), 5.91 (s, 1H), 4.93 (ddd, *J* = 10.5, 8.2, 6.7 Hz, 1H), 4.25 (t, *J* = 9.1 Hz, 1H), 4.18 (dd, *J* = 9.1, 4.8 Hz, 1H), 3.87 (s, 3H), 3.85 (s, 3H), 3.79 (dt, *J* = 9.1, 4.3 Hz, 1H), 3.50 (td, *J* = 10.3, 5.6 Hz, 1H), 2.93 (d, *J* = 6.2 Hz, 2H), 2.85 (dd, *J* = 18.0, 4.8 Hz, 1H), 2.45–2.27 (m, 1H), 0.98 (s, 9H). ^13^C {^1^H} NMR (101 MHz, CDCl_3_): δ 157.1, 149.4, 148.8, 133.7, 131.3, 119.7, 119.6, 111.6, 110.6, 86.5, 64.4, 63.2, 56.2, 56.0, 43.6, 35.5, 32.6, 29.9, 25.3. IR ν(cm^–1^): 2959, 2921, 1743, 1548, 1240, 1025. HRMS (ESI) *m*/*z*: [M + H]^+^ calculated for C_21_H_28_N_2_O_6_, 405.2020; found, 405.2018.

#### (*S*)-4-(*tert*-Butyl)-3-((4*R*,5*R*)-4-nitro-5-(thiophen-2-yl)cyclohex-1-en-1-yl)oxazolidin-2-one (**31**)

Yellow oil. Synthesized by GP2 (0.0193 g, 55%). R_f_ = 0.16 (cyclohexane:EtOAc, 3:1). Melting point: 172–178 °C. Inseparable diastereomers, dr 86:14. XRD sample prepared by slow evaporation of compound in CH_2_Cl_2_ with pentane antisolvent. *Major diastereomer:*^1^H NMR (400 MHz, CDCl_3_): δ 7.22 (dd, *J* = 4.7, 1.6 Hz, 1H), 6.94 (d, *J* = 4.7 Hz, 2H), 5.91 (s, 1H), 4.87 (app dd, *J* = 6.9, 2.3 Hz, 1H), 4.27 (t, *J* = 9.1 Hz, 1H), 4.19 (dd, *J* = 9.2, 4.6 Hz, 1H), 3.89 (td, *J* = 10.2, 5.5 Hz, 1H), 3.80 (dd, *J* = 8.9, 4.6 Hz, 1H), 3.00 (dd, *J* = 17.1, 4.2 Hz, 1H), 2.96–2.90 (m, 2H), 2.49–2.35 (m, 1H), 0.97 (s, 9H). ^13^C {^1^H} NMR (101 MHz, CDCl_3_): δ 156.9, 141.4, 133.2, 127.3, 125.9, 125.0, 119.2, 87.4, 64.4, 63.2, 39.3, 35.5, 33.3, 29.8, 25.3. IR ν(cm^–1^): 2962, 2921, 2871, 1746, 1404, 1368, 710. HRMS (ESI) *m*/*z*: [M + H]^+^ calculated for C_17_H_22_N_2_O_4_S, 351.1373; found, 351.1375.

#### (*S*)-4-(*tert*-Butyl)-3-((4*R*,5*R*)-5-(furan-2-yl)-4-nitrocyclohex-1-en-1-yl)oxazolidin-2-one (**32**)

Yellow oil. Synthesized by GP2 (0.0056 g, 17%). R_f_ = 0.13 (cyclohexane:EtOAc, 3:1). Inseparable diastereomers., dr 90:10. *Major diastereomer:*^1^H NMR (400 MHz, CDCl_3_): δ 7.34 (d, *J* = 1.9 Hz, 1H), 6.29 (dd, *J* = 3.2, 1.9 Hz, 1H), 6.23 (d, *J* = 3.2 Hz, 1H), 5.90–5.83 (m, 1H), 5.05–4.89 (m, 1H), 4.31–4.23 (m, 1H), 4.19 (dd, *J* = 9.1, 4.6 Hz, 1H), 3.80 (dd, *J* = 8.9, 4.6 Hz, 1H), 3.75 (dt, *J* = 9.9, 4.9 Hz, 1H), 2.98–2.75 (m, 3H), 2.54–2.41 (m, 1H), 0.96 (s, 9H). ^13^C {^1^H} NMR (101 MHz, CDCl_3_): δ 157.0, 151.7, 142.6, 133.2, 118.9, 110.7, 107.6, 84.3, 64.4, 63.4, 37.4, 35.5, 29.4, 29.2, 25.4. IR ν(cm^–1^): 2963, 2922, 2873, 1743, 1550, 1402, 1369, 1197, 733. HRMS (ESI) *m*/*z*: [M + H]^+^ calculated for C_17_H_22_N_2_O_5_, 335.1601; found, 335.1601.

#### (3*aR*,7*aS*)-5-((*S*)-4-(*tert*-Butyl)-2-oxooxazolidin-3-yl)-3*a*,4,7,7*a*-tetrahydro-1*H*-isoindole-1,3(2*H*)-dione (**33**)

White solid. Synthesized by GP2 (0.0205 g, 70%). R_f_ = 0.42 (cyclohexane:EtOAc, 1:2). Melting point: 181–186 °C. Diastereomers separable by column chromatography, dr 92:8. *Major diastereomer:*^1^H NMR (500 MHz, CDCl_3_): δ 8.83 (s, 1H), 5.92 (ddd, J = 6.2, 4.3, 1.5 Hz, 1H), 4.27 (td, J = 9.1, 2.3 Hz, 1H), 4.15 (dd, J = 9.1, 4.2 Hz, 1H), 3.82 (dd, J = 9.1, 4.4 Hz, 1H), 3.25 (ddd, J = 9.0, 7.8, 3.7 Hz, 1H), 3.12 (ddd, J = 9.0, 7.8, 3.7 Hz, 1H), 2.70–2.66 (m, 1H), 2.66–2.62 (m, 1H), 2.58 (dd, J = 15.4, 7.6 Hz, 1H), 2.34 (dddd, J = 15.6, 7.6, 4.4, 1.5 Hz, 1H), 0.89 (s, 9H). ^13^C {^1^H} NMR (126 MHz, CDCl_3_): δ 179.7, 156.7, 135.7, 119.3, 64.4, 63.6, 41.3, 40.2, 35.4, 26.5, 25.6, 23.9. IR ν(cm^–1^): 3198, 2962, 2922, 1707, 1411, 1056, 758. HRMS (ESI) *m*/*z*: [M + H]^+^ calculated for C_15_H_20_N_2_O_4_, 293.1496; found, 293.1496. [α]_D_^20^: −51.19 (c 0.1 CHCl_3_).

#### (3*aS*,7*aR*)-5-((*S*)-4-(*tert*-Butyl)-2-oxooxazolidin-3-yl)-3*a*,4,7,7*a*-tetrahydro-1*H*-isoindole-1,3(2*H*)-dione (**33’**)

Clear oil. Synthesized by GP2 (0.0015 g, 5%). R_f_ = 0.15 (cyclohexane:EtOAc, 1:2). *Minor diastereomer*:^1^H NMR (500 MHz, CDCl_3_): δ 8.51 (s, 1H), 6.01–5.95 (m, 1H), 4.27 (t, *J* = 9.2 Hz, 1H), 4.19 (dd, *J* = 9.2, 4.1 Hz, 1H), 3.76 (dt, *J* = 9.2, 5.1 Hz, 1H), 3.24 (td, *J* = 8.9, 3.3 Hz, 1H), 3.16 (ddd, *J* = 8.9, 7.6, 3.3 Hz, 1H), 2.74 (dd, *J* = 16.2, 3.3 Hz, 1H), 2.67 (ddd, *J* = 16.2, 6.2, 3.3 Hz, 1H), 2.54–2.41 (m, 2H), 0.88 (s, 9H). ^13^C {^1^H} NMR (126 MHz, CDCl_3_): δ 179.6, 179.4, 157.2, 135.6, 122.5, 64.7, 63.5, 40.6, 39.7, 35.4, 25.6, 25.2, 23.4. IR ν(cm^–1^): 3200, 2959, 2923, 1707, 1414, 1199, 757. HRMS (ESI) *m*/*z*: [M + H]^+^ calculated for C_15_H_20_N_2_O_4_, 293.1496; found, 293.1496. [α]_D_^20^: −35.75 (c 0.1 CHCl_3_).

#### 5-((*S*)-4-(*tert*-Butyl)-2-oxooxazolidin-3-yl)-2-methyl-3*a*,4,7,7*a*-tetrahydro-1*H*-isoindole-1,3(2*H*)-dione (**34**)

Clear oil. Synthesized by GP2 (0.0189 g, 62%). R_f_ = 0.34 (cyclohexane:EtOAc, 1:2). Diastereomers separable by column chromatography, dr 93:7. Minor diastereomer not isolated. XRD sample prepared by slow evaporation of compound in CH_2_Cl_2_ with pentane antisolvent. *Major diastereomer*:^1^H NMR (400 MHz, CDCl_3_): δ 5.82 (ddd, *J* = 6.5, 4.1, 2.0 Hz, 1H), 4.32–4.22 (m, 1H), 4.14 (dd, *J* = 9.2, 4.4 Hz, 1H), 3.81 (dd, *J* = 8.9, 4.4 Hz, 1H), 3.26–3.15 (m, 1H), 3.14–3.06 (m, 1H), 2.93 (s, 3H), 2.78–2.67 (m, 2H), 2.60 (ddt, *J* = 15.5, 7.5, 1.8 Hz, 1H), 2.32 (dddd, *J* = 15.6, 7.3, 4.1, 1.8 Hz, 1H), 0.87 (s, 9H). ^13^C {^1^H} NMR (101 MHz, CDCl_3_): δ 179.6, 179.4, 156.6, 135.9, 118.6, 64.4, 63.5, 40.1, 39.1, 35.4, 26.9, 25.5, 25.5, 25.3, 24.1. IR ν(cm^–1^): 2961, 2940, 1730, 1687, 1410, 1252, 767. HRMS (ESI) *m*/*z*: [M + H]^+^ calculated for C_16_H_22_N_2_O_4_, 307.1652; found, 307.1654. [α]_D_^20^: −45.27 (c 0.1 CHCl_3_).

#### (3*aR*,7*aS*)-2-Benzyl-5-((*S*)-4-(*tert*-butyl)-2-oxooxazolidin-3-yl)-3*a*,4,7,7*a*-tetrahydro-1*H*-isoindole-1,3(2*H*)-dione (**35**)

Off-white solid. Synthesized by GP2 (0.0226 g, 59%). R_f_ = 0.33 (cyclohexane:EtOAc, 1:2). Melting point: 174–178 °C. Diastereomers separable by column chromatography, dr 84:16. *Major diastereomer:*^1^H NMR (500 MHz, CDCl_3_): δ 7.37–7.27 (m, 5H), 5.93–5.87 (m, 1H), 4.60 (s, 2H), 4.26 (t, *J* = 8.9 Hz, 1H), 4.16 (dd, *J* = 9.1, 4.2 Hz, 1H), 3.82 (dd, *J* = 8.8, 4.2 Hz, 1H), 3.20 (ddd, *J* = 9.1, 7.4, 4.2 Hz, 1H), 3.08 (td, *J* = 8.3, 3.7 Hz, 1H), 2.73–2.59 (m, 3H), 2.42–2.33 (m, 1H), 0.88 (s, 9H). ^13^C {^1^H} NMR (126 MHz, CDCl_3_): δ 179.1, 178.9, 156.5, 135.8, 128.8, 128.0, 118.5, 64.4, 63.6, 42.8, 40.0, 38.9, 35.5, 26.7, 25.6, 24.0. IR ν(cm-^1^): 2966, 2912, 1733, 1691, 1402, 1057. HRMS (ESI) *m*/*z*: [M + H]^+^ calculated for C_22_H_26_N_2_O_4_, 383.1965; found, 383.1967. [α]_D_^20^: −46.61 (c 0.1 CHCl_3_).

#### (3*aS*,7*aR*)-2-Benzyl-5-((*S*)-4-(*tert*-butyl)-2-oxooxazolidin-3-yl)-3*a*,4,7,7*a*-tetrahydro-1*H*-isoindole-1,3(2*H*)-dione (**35’**)

White solid. Synthesized by GP2 (0.0027 g, 7%). R_f_ = 0.22 (cyclohexane:EtOAc, 1:2). Melting point: 149–152 °C. *Minor diastereomer:*^1^H NMR (500 MHz, CDCl_3_): δ 7.33 (dd, *J* = 7.9, 1.5 Hz, 2H), 7.31–7.25 (m, 3H), 5.99–5.92 (m, 1H), 4.62 (s, 2H), 4.25 (t, *J* = 9.0 Hz, 1H), 4.17 (dd, *J* = 9.1, 3.9 Hz, 1H), 3.77 (dd, *J* = 8.7, 3.8 Hz, 1H), 3.67–3.58 (m, 1H), 3.06 (td, *J* = 7.2, 4.5 Hz, 1H), 2.45–2.34 (m, 1H), 2.15–2.02 (m, 2H), 2.02–1.95 (m, 1H), 0.82 (s, 9H). ^13^C {^1^H} NMR (126 MHz, CDCl_3_): δ 178.3, 176.3, 156.5, 138.5, 135.8, 128.7, 128.1, 115.3, 64.5, 63.5, 42.5, 41.2, 38.7, 35.7, 25.4, 24.2, 22.5. IR ν(cm^–1^): 2968, 2901, 1733, 1694, 1400, 1055. HRMS (ESI) *m*/*z*: [M + H]^+^ calculated for C_22_H_26_N_2_O_4_, 383.1965; found, 383.1965. [α]_D_^20^: −42.78 (c 0.1 CHCl_3_).

#### (3*aR*,7*aS*)-5-((*S*)-4-(*tert*-Butyl)-2-oxooxazolidin-3-yl)-2-phenyl-3*a*,4,7,7*a*-tetrahydro-1*H*-isoindole-1,3(2*H*)-dione **(36**)

Off-white solid. Synthesized by GP2 (0.0184 g, 50%). R_f_ = 0.45 (cyclohexane:EtOAc, 1:2). Melting point: 211–216 °C. Diastereomers separable by column chromatography, dr 90:10. *Major diastereomer*:^1^H NMR (500 MHz, CDCl_3_): δ 7.44 (t, *J* = 7.7 Hz, 2H), 7.39–7.34 (m, 1H), 7.30–7.26 (m, 2H), 6.00 (ddd, *J* = 6.4, 3.9, 1.9 Hz, 1H), 4.26 (t, *J* = 9.0 Hz, 1H), 4.16 (dd, *J* = 9.1, 4.2 Hz, 1H), 3.83 (dd, *J* = 8.9, 4.1 Hz, 1H), 3.43–3.35 (m, 1H), 3.27 (ddd, *J* = 9.3, 7.7, 3.0 Hz, 1H), 2.86 (dd, *J* = 7.0, 3.0 Hz, 1H), 2.81 (dd, *J* = 15.1, 3.1 Hz, 1H), 2.67–2.58 (m, 1H), 2.41 (dddd, *J* = 15.7, 7.5, 4.0, 1.7 Hz, 1H), 0.91 (s, 9H). ^13^C {^1^H} NMR (126 MHz, CDCl_3_): δ 178.3, 156.4, 135.6, 132.1, 129.3, 128.8, 126.9, 119.3, 64.3, 63.5, 40.3, 39.2, 35.5, 26.8, 24.5. IR ν(cm^–1^): 2972, 2947, 2921, 1735, 1701, 1390, 1177. HRMS (ESI) *m*/*z*: [M + H]^+^ calculated for C_21_H_24_N_2_O_4_, 369.1809; found, 369.1807. [α]_D_^20^: −8.78 (c 0.1, CHCl_3_).

#### (3*aS*,7*aR*)-5-((*S*)-4-(*tert*-butyl)-2-oxooxazolidin-3-yl)-2-phenyl-3*a*,4,7,7*a*-tetrahydro-1*H*-isoindole-1,3(2*H*)-dione (**36’**)

Clear oil. Synthesized by GP2 (0.0033 g, 9%). R_f_ = 0.26 (cyclohexane:EtOAc, 1:2). *Minor diastereomer:*^1^H NMR (500 MHz, CDCl_3_): δ 7.47–7.42 (m, 2H), 7.39–7.35 (m, 1H), 7.31–7.27 (m, 2H), 6.05–5.99 (m, 1H), 4.27 (t, *J* = 9.0 Hz, 1H), 4.19 (dd, *J* = 9.2, 4.1 Hz, 1H), 3.77 (dd, *J* = 8.8, 4.1 Hz, 1H), 3.43–3.34 (m, 1H), 3.33–3.26 (m, 1H), 2.88 (dd, *J* = 16.2, 2.9 Hz, 1H), 2.83 (ddd, *J* = 16.2, 6.5, 3.3 Hz, 1H), 2.61–2.51 (m, 2H), 0.87 (s, 9H). ^13^C {^1^H} NMR (126 MHz, CDCl_3_): δ 178.5, 178.2, 157.1, 135.7, 132.0, 129.2, 128.7, 126.4, 122.3, 64.6, 63.6, 39.4, 38.6, 35.5, 25.6, 23.6. IR ν(cm-1): 2969, 2902, 1740, 1704, 1384, 1054, 763. HRMS (ESI) *m*/*z*: [M + H]^+^ calculated for C_21_H_24_N_2_O_4_, 369.1809; found, 369.1808. [α]_D_^20^: −52.43 (c 0.1, CHCl_3_).

#### (3*aR*,7*aS*)-5-((*S*)-4-(*tert*-Butyl)-2-oxooxazolidin-3-yl)-2-(*p*-tolyl)-3a,4,7,7a-tetrahydro-1*H*-isoindole-1,3(2*H*)-dione (**37**)

Off-white solid. Synthesized by GP2 (0.0172 g, 45%). R_f_ = 0.42 (cyclohexane:EtOAc, 1:2). Melting point: 208–213 °C. Diastereomers separable by column chromatography, dr 82:18. *Major diastereomer:*^1^H NMR (500 MHz, CDCl_3_): δ 7.24 (d, *J* = 8.1 Hz, 2H), 7.15 (d, *J* = 8.3 Hz, 2H), 5.99 (dt, *J* = 4.5, 2.1 Hz, 1H), 4.26 (t, *J* = 9.0 Hz, 1H), 4.16 (dd, *J* = 9.2, 4.0 Hz, 1H), 3.83 (dd, *J* = 8.8, 4.0 Hz, 1H), 3.38 (ddd, *J* = 10.4, 7.4, 3.3 Hz, 1H), 3.26 (td, *J* = 8.5, 3.0 Hz, 1H), 2.83–2.76 (m, 2H), 2.67–2.58 (m, 1H), 2.46–2.38 (m, 1H), 2.35 (s, 3H), 0.91 (s, 9H). ^13^C {^1^H} NMR (126 MHz, CDCl_3_): δ 178.7, 178.5, 156.4, 138.8, 135.6, 129.6, 129.4, 126.7, 126.2, 119.1, 64.3, 63.5, 40.2, 39.2, 35.6, 26.8, 25.6, 24.4, 21.4. IR ν(cm^–1^): 2963, 2925, 1732, 1700, 1394, 1197. HRMS (ESI) *m*/*z*: [M + H]^+^ calculated for C_22_H_26_N_2_O_4_, 383.1965; found, 383.1964. [α]_D_^20^: 3.23 (c 0.1, CHCl_3_).

#### (3*aS*,7*aR*)-5-((*S*)-4-(*tert*-Butyl)-2-oxooxazolidin-3-yl)-2-(*p*-tolyl)-3*a*,4,7,7*a*-tetrahydro-1*H*-isoindole-1,3(2*H*)-dione (**37’**)

Clear oil. Synthesized by GP2 (0.0177g, 15%). R_f_ = 0.15 (cyclohexane:EtOAc, 1:2). *Minor diastereomer*:^1^H NMR (500 MHz, CDCl_3_): δ 7.26 (d, *J* = 7.5 Hz, 2H), 7.12–7.09 (m, 2H), 6.07 (d, *J* = 4.2 Hz, 1H), 4.29 (t, *J* = 9.0 Hz, 1H), 4.24–4.19 (m, 1H), 3.84 (dd, *J* = 8.7, 3.9 Hz, 1H), 3.82–3.74 (m, 1H), 3.24 (dt, *J* = 8.3, 5.7 Hz, 1H), 2.58–2.49 (m, 1H), 2.38 (s, 3H), 2.30–2.23 (m, 1H), 2.16–2.03 (m, 2H), 0.93 (s, 9H). ^13^C {^1^H} NMR (126 MHz, CDCl_3_): δ 177.7, 175.8, 156.5, 138.9, 138.6, 129.9, 129.1, 126.3, 115.7, 64.6, 63.5, 41.5, 38.8, 35.9, 35.5, 25.5, 24.2, 22.4, 21.3. IR ν(cm-1): 2970, 2902, 1746, 1704, 1393, 1049, 786. HRMS (ESI) *m*/*z*: [M + H]^+^ calculated for C_22_H_26_N_2_O_4_, 383.1965; found, 383.1964. [α]_D_^20^: −68.04 (c 0.1, CHCl_3_).

#### 5-((*S*)-4-(*tert*-Butyl)-2-oxooxazolidin-3-yl)-2-(2-(*tert*-butyl)phenyl)-3*a*,4,7,7*a*-tetrahydro-1*H*-isoindole-1,3(2*H*)-dione (**38**)

Off-white solid. Synthesized by GP2 (0.0149 g, 35%). R_f_ = 0.44 (cyclohexane:EtOAc, 1:2). Melting point: 209–214 °C. Diastereomers separable by column chromatography, dr 4:2:2:1. XRD sample prepared by slow evaporation of compound in CH_2_Cl_2_ with pentane antisolvent. *Major diastereomer:*^1^H NMR (500 MHz, CDCl_3_): δ 7.51 (dd, *J* = 8.1, 1.4 Hz, 1H), 7.35 (td, *J* = 7.8, 1.5 Hz, 1H), 7.29 (td, *J* = 7.7, 1.6 Hz, 1H), 7.05 (dd, *J* = 7.7, 1.5 Hz, 1H), 6.11 (ddd, *J* = 6.4, 3.7, 2.0 Hz, 1H), 4.25 (t, *J* = 8.9 Hz, 1H), 4.17 (dd, *J* = 9.1, 3.7 Hz, 1H), 3.82 (dd, *J* = 8.8, 3.6 Hz, 1H), 3.39 (ddd, *J* = 9.8, 6.9, 3.1 Hz, 1H), 3.32–3.24 (m, 1H), 2.89 (ddd, *J* = 15.7, 7.1, 2.8 Hz, 1H), 2.80 (dd, *J* = 15.1, 3.1 Hz, 1H), 2.53 (ddt, *J* = 15.1, 6.9, 1.7 Hz, 1H), 2.39 (m, 1H), 1.28 (s, 9H), 0.93 (s, 9H). ^13^C {^1^H} NMR (126 MHz, CDCl_3_): δ 179.6, 179.4, 156.2, 147.3, 135.4, 131.7, 130.6, 129.8, 128.3, 127.9, 119.4, 64.2, 63.5, 40.7, 39.5, 35.6, 35.6, 31.7, 26.3, 25.7, 25.5, 24.4. IR ν(cm^–1^): 2959, 2915, 1741, 1704, 1123, 759. HRMS (ESI) *m*/*z*: [M + H]^+^ calculated for C_25_H_32_N_2_O_4_, 425.2435; found, 425.2433. [α]_D_^20^: −13.63 (c 0.1, CHCl_3_).

#### (3*aR*,4*R*,7*aS*)-5-((*S*)-4-Isopropyl-2-oxooxazolidin-3-yl)-4-phenyl-3*a*,4,7,7*a*-tetrahydro-1*H*-isoindole-1,3(2*H*)-dione (**39**)

Off-white solid. Synthesized by GP2 (0.0213 g, 60%). R_f_ = 0.53 (cyclohexane:EtOAc, 1:2). Melting point range: 107–111 °C. Diastereomers separable by column chromatography, dr 76:24. ^1^H NMR (400 MHz, CDCl_3_ δ 7.51 (s, 1H), 7.24–7.18 (m, 3H), 7.12 (dd, *J* = 6.7, 2.9 Hz, 2H), 6.04 (dd, *J* = 6.0, 2.7 Hz, 1H), 4.52 (d, *J* = 7.4 Hz, 1H), 4.13 (q, *J* = 7.0 Hz, 1H), 4.07–3.95 (m, 2H), 3.47 (dd, *J* = 9.2, 7.4 Hz, 1H), 3.21 (ddd, *J* = 11.5, 9.3, 4.6 Hz, 1H), 3.11–3.00 (m, 1H), 2.81–2.68 (m, 1H), 1.84 (ddq, *J* = 9.7, 6.9, 3.4 Hz, 1H), 0.83 (d, *J* = 7.1 Hz, 3H), 0.46 (d, *J* = 6.8 Hz, 3H). ^13^C {^1^H} NMR (101 MHz, CDCl_3_): δ 179.2, 176.9, 156.1, 135.7, 134.9, 129.3, 128.8, 128.3, 118.8, 62.3, 59.3, 46.5, 40.3, 37.4, 28.2, 20.9, 17.9, 13.7. IR ν(cm^–1^): 2962, 2927, 1703, 727, 700. HRMS (ESI) *m*/*z*: [M + H]^+^ calculated for C_20_H_22_N_2_O_4_, 355.1652; found, 355.1651. [α]_D_^20^: −180.81 (c 0.1, CHCl_3_).

#### (3*aR*,4*S*,7*aS*)-5-((*S*)-4-Isopropyl-2-oxooxazolidin-3-yl)-4-phenyl-3*a*,4,7,7*a*-tetrahydro-1*H*-isoindole-1,3(2*H*)-dione (**40**)

Yellow oil. Synthesized by GP2 (0.0206 g, 58%). R_f_ = 0.31 (cyclohexane:EtOAc, 1:2). Diastereomers separable by column chromatography, dr 86:14. ^1^H NMR (400 MHz, CDCl_3_): δ 7.31 (s, 1H), 7.25–7.20 (m, 3H), 7.14 (dd, *J* = 6.8, 2.9 Hz, 2H), 6.05 (dd, *J* = 5.9, 2.7 Hz, 1H), 4.53 (d, *J* = 7.4 Hz, 1H), 4.14 (d, *J* = 7.4 Hz, 1H), 4.07–3.96 (m, 2H), 3.49 (dd, *J* = 9.2, 7.4 Hz, 1H), 3.28–3.17 (m, 1H), 3.06 (d, *J* = 3.3 Hz, 1H), 2.78 (d, *J* = 11.8 Hz, 1H), 1.85 (d, *J* = 2.8 Hz, 1H), 0.83 (d, *J* = 7.1 Hz, 3H), 0.46 (d, *J* = 6.8 Hz, 3H). ^13^C {^1^H} NMR (101 MHz, CDCl_3_): δ 179.0, 176.8, 156.2, 135.7, 135.0, 129.3, 128.9, 128.4, 118.8, 62.3, 59.3, 46.6, 40.4, 37.4, 29.8, 28.2, 20.9, 18.0, 13.7. IR ν(cm^–1^): 2918, 2850, 1704, 1408, 1171, 700. HRMS (ESI) *m*/*z*: [M + H]^+^ calculated for C_20_H_22_N_2_O_4_, 355.1652; found, 355.1654. [α]_D_^20^: −161.90 (c 0.1, CHCl_3_).

#### (3*aR*,4*R*,7*aS*)-5-((*S*)-4-Isopropyl-2-oxooxazolidin-3-yl)-2-methyl-4-phenyl-3*a*,4,7,7*a*-tetrahydro-1*H*-isoindole-1,3(2*H*)-dione (**41**)

Off-white solid. Synthesized by GP2 (0.0133 g, 36%). R_f_ = 0.33 (cyclohexane:EtOAc, 1:2). Melting point: 63–67 °C. Diastereomers separable by column chromatography, dr 79:11. ^1^H NMR (400 MHz, CDCl_3_): δ 7.21 (d, *J* = 6.9 Hz, 3H), 7.08 (d, *J* = 7.7 Hz, 2H), 6.08 (dd, *J* = 5.7, 2.5 Hz, 1H), 4.46 (d, *J* = 7.2 Hz, 1H), 4.19–4.07 (m, 1H), 4.01 (h, *J* = 5.2 Hz, 2H), 3.47 (t, *J* = 7.8 Hz, 1H), 3.11 (td, *J* = 10.4, 4.0 Hz, 2H), 2.84–2.70 (m, 1H), 2.32 (s, 3H), 1.92–1.80 (m, 1H), 0.84 (d, *J* = 7.1 Hz, 3H), 0.50 (d, *J* = 6.8 Hz, 3H). ^13^C {^1^H} NMR (126 MHz, CDCl_3_): δ 179.2, 177.2, 156.1, 135.7, 134.3, 129.1, 128.6, 128.2, 119.4, 62.3, 59.2, 45.8, 40.5, 36.1, 28.3, 23.9, 20.9, 17.9, 13.8. IR ν(cm^–1^): 2961, 2926, 1740, 1697, 1057, 702. HRMS (ESI) *m*/*z*: [M + H]^+^ calculated for C_21_H_24_N_2_O_4_, 369.1809; found, 369.1807. [α]_D_^20^: −156.3 (c 0.1, CHCl_3_).

#### (3*aR*,4*S*,7*aS*)-5-((*S*)-4-Isopropyl-2-oxooxazolidin-3-yl)-2-methyl-4-phenyl-3*a*,4,7,7*a*-tetrahydro-1*H*-isoindole-1,3(2*H*)-dione (**42**)

Pale yellow oil. Synthesized by GP2 (0.0151 g, 41%). R_f_ = 0.41 (cyclohexane:EtOAc, 1:2). Diastereomers separable by column chromatography, dr 76:24. ^1^H NMR (400 MHz, CDCl_3_): δ 7.24–7.17 (m, 3H), 7.08 (dd, *J* = 7.5, 1.9 Hz, 2H), 6.08 (dd, *J* = 5.8, 2.6 Hz, 1H), 4.45 (d, *J* = 7.2 Hz, 1H), 4.18–4.09 (m, 1H), 4.06–3.95 (m, 2H), 3.51–3.43 (m, 1H), 3.18–3.09 (m, 2H), 2.84–2.70 (m, 1H), 2.32 (s, 3H), 1.92–1.80 (m, 1H), 0.84 (d, *J* = 7.0 Hz, 3H), 0.50 (d, *J* = 7.0 Hz, 3H). ^13^C {^1^H} NMR (101 MHz, CDCl_3_): δ 179.3, 177.3, 156.1, 135.7, 134.3, 129.2, 128.7, 128.2, 119.4, 62.3, 59.2, 45.8, 40.5, 36.1, 28.3, 23.9, 20.9, 17.9, 13.8. IR ν(cm^–1^): 2923, 1741, 1696, 1407, 702. HRMS (ESI) *m*/*z*: [M + H]^+^ calculated for C_21_H_24_N_2_O_4_, 369.1809; found, 369.1809. [α]_D_^20^: −176.90 (c 0.1, CHCl_3_).

#### (3*aR*,4*R*,7*aS*)-5-((*S*)-4-Isopropyl-2-oxooxazolidin-3-yl)-2,4-diphenyl-3*a*,4,7,7*a*-tetrahydro-1*H*-isoindole-1,3(2*H*)-dione (**43**)

Off-white solid. Synthesized by GP2 (0.0077 g, 18%). R_f_ = 0.41 (cyclohexane:EtOAc, 1:2). Melting point: 86–90 °C. Diastereomers separable by column chromatography, dr 75:25. ^1^H NMR (400 MHz, CDCl_3_): δ 7.30–7.16 (m, 8H), 6.43–6.33 (m, 2H), 6.11 (dd, *J* = 5.8, 2.7 Hz, 1H), 4.61 (d, *J* = 7.3 Hz, 1H), 4.15 (q, *J* = 7.8 Hz, 1H), 4.10–3.99 (m, 2H), 3.64 (dd, *J* = 9.1, 7.3 Hz, 1H), 3.37 (ddd, *J* = 11.6, 9.1, 4.0 Hz, 1H), 3.31–3.20 (m, 1H), 2.94–2.80 (m, 1H), 1.88 (ddq, *J* = 9.9, 6.9, 3.5 Hz, 1H), 0.85 (d, *J* = 6.9 Hz, 3H), 0.49 (d, *J* = 6.9 Hz, 3H). ^13^C {^1^H} NMR (126 MHz, CDCl_3_): δ 178.4, 176.1, 156.2, 136.0, 134.7, 129.7, 129.1, 128.9, 128.6, 128.5, 126.4, 119.1, 62.3, 59.3, 45.5, 40.6, 36.4, 31.1, 28.3, 21.1, 18.0, 13.7. IR ν(cm^–1^): 2960, 2924, 1740, 1708, 1381, 1175, 693. HRMS (ESI) *m*/*z*: [M + H]^+^ calculated for C_26_H_26_N_2_O_4_, 431.1965; found, 431.1964. [α]_D_^20^: −139.05 (c 0.1, CHCl_3_).

#### (3*aR*,4*S*,7*aS*)-5-((*S*)-4-Isopropyl-2-oxooxazolidin-3-yl)-2,4-diphenyl-3*a*,4,7,7*a*-tetrahydro-1*H*-isoindole-1,3(2*H*)-dione (**44**)

Pale yellow oil. Synthesized by GP2 (0.0211 g, 49%). R_f_ = 0.53 (cyclohexane:EtOAc, 1:2). Diastereomers separable by column chromatography, dr 61:39. ^1^H NMR (400 MHz, CDCl_3_): δ 7.31–7.16 (m, 8H), 6.43–6.33 (m, 2H), 6.11 (dd, *J* = 5.8, 2.7 Hz, 1H), 4.60 (d, *J* = 7.3 Hz, 1H), 4.16 (t, *J* = 7.8 Hz, 1H), 4.10–3.98 (m, 2H), 3.64 (dd, *J* = 9.1, 7.3 Hz, 1H), 3.37 (ddd, *J* = 11.7, 9.1, 4.0 Hz, 1H), 3.31–3.20 (m, 1H), 2.94–2.80 (m, 1H), 1.89 (ddd, *J* = 13.8, 6.9, 3.0 Hz, 1H), 0.85 (d, *J* = 6.9 Hz, 3H), 0.49 (d, *J* = 6.9 Hz, 3H). ^13^C {^1^H} NMR (101 MHz, CDCl_3_): δ 178.4, 176.2, 156.2, 136.0, 134.7, 131.4, 129.7, 129.1, 129.0, 128.6, 128.5, 126.4, 119.2, 62.3, 59.3, 45.5, 40.6, 36.4, 28.3, 21.1, 18.0, 13.7. IR ν(cm^–1^): 2960, 2924, 1740, 1708, 1381, 1175, 693. HRMS (ESI) *m*/*z*: [M + H]^+^ calculated for C_26_H_26_N_2_O_4_, 431.1965; found, 431.1963. [α]_D_^20^: −112.9 (c 0.1, CHCl_3_).

#### (3*aR*,4*R*,7*aS*)-5-((*S*)-4-(*tert*-Butyl)-2-oxooxazolidin-3-yl)-4-phenyl-3*a*,4,7,7*a*-tetrahydro-1*H*-isoindole-1,3(2*H*)-dione (**45**)

Off-white solid. Synthesized by GP2 (0.0236 g, 64%). R_f_ = 0.72 (cyclohexane:EtOAc, 1:2). Melting point: 237–240 °C. Diastereomers separable by column chromatography, dr 72:28. XRD sample prepared by slow evaporation of compound in CH_2_Cl_2_ with pentane antisolvent. *Major diastereomer:*^1^H NMR (500 MHz, CDCl_3_): δ 7.33 (s, 1H), 7.23 (dd, *J* = 5.2, 1.8 Hz, 3H), 7.15 (dd, *J* = 7.1, 2.5 Hz, 2H), 6.34 (dd, *J* = 5.7, 2.8 Hz, 1H), 4.11–4.08 (m, 1H), 4.07 (d, *J* = 4.9 Hz, 1H), 3.94 (t, *J* = 9.0 Hz, 1H), 3.74 (dd, *J* = 8.9, 3.4 Hz, 1H), 3.36 (dd, *J* = 9.2, 7.4 Hz, 1H), 3.20 (ddd, *J* = 11.3, 9.2, 4.3 Hz, 1H), 3.15–3.06 (m, 1H), 2.82–2.71 (m, 1H), 0.97 (s, 9H). ^13^C {^1^H} NMR (126 MHz, CDCl_3_): δ 178.7, 176.8, 156.8, 134.7, 134.5, 129.3, 129.0, 128.6, 122.2, 63.9, 61.7, 46.5, 40.4, 37.2, 35.7, 25.3, 20.8. IR ν(cm^–1^): 2958, 2922, 1710, 1673, 1411, 1162, 1122, 762, 705. HRMS (ESI) *m*/*z*: [M + H]^+^ calculated for C_21_H_24_N_2_O_4_, 369.1809; found, 369.1812. [α]_D_^20^: −247.16 (c 0.1, CHCl_3_).

#### (3*aS*,4*R*,7*aR*)-5-((*S*)-4-(*tert*-Butyl)-2-oxooxazolidin-3-yl)-4-phenyl-3*a*,4,7,7*a*-tetrahydro-1*H*-isoindole-1,3(2*H*)-dione (**45’**)

Pale yellow oil. Synthesized by GP2 (0.0037 g, 10%). R_f_ = 0.61 (cyclohexane:EtOAc, 1:2). *Minor diastereomer:*^1^H NMR (500 MHz, CDCl_3_): δ 8.14 (s, 1H), 7.36–7.26 (m, 5H), 6.31 (t, *J* = 4.7 Hz, 1H), 4.17 (s, 1H), 4.08 (dd, *J* = 9.1, 2.3 Hz, 1H), 3.82 (t, *J* = 8.8 Hz, 1H), 3.60 (dd, *J* = 8.5, 2.3 Hz, 1H), 3.35 (dd, *J* = 9.4, 1.8 Hz, 1H), 3.16 (dt, *J* = 9.4, 5.9 Hz, 1H), 2.75 (t, *J* = 5.0 Hz, 2H), 0.88 (s, 9H). ^13^C {^1^H} NMR (101 MHz, CDCl_3_): δ 179.3, 178.5, 157.2, 139.7, 134.9, 129.6, 127.8, 127.6, 123.7, 64.4, 61.8, 49.1, 39.8, 38.1, 35.9, 25.4, 23.7. IR ν(cm^–1^): 2968, 2918, 1705, 1407, 1122, 702. HRMS (ESI) *m*/*z*: [M + H]^+^ calculated for C_21_H_24_N_2_O_4_, 369.1809; found, 369.1812. [α]_D_^20^: −101.29 (c 0.1, CHCl_3_).

#### (3*aR*,4*S*,7*aS*)-5-((*S*)-4-(*tert*-Butyl)-2-oxooxazolidin-3-yl)-4-phenyl-3*a*,4,7,7*a-*tetrahydro-1*H*-isoindole-1,3(2*H*)-dione (**46**)

Off-white solid. Synthesized by GP2 (0.0221 g, 60%). R_f_ = 0.52 (cyclohexane:EtOAc, 1:2). Melting point: 82–85 °C. Diastereomers separable by column chromatography, dr 73:27. XRD sample prepared by slow evaporation of compound in CH_2_Cl_2_ with pentane antisolvent. ^1^H NMR (500 MHz, DMSO): δ 11.36 (s, 1H), 7.31 (d, J = 7.2 Hz, 2H), 7.28 (d, J = 7.2 Hz, 2H), 7.25–7.17 (m, 1H), 5.82 (dd, J = 8.0, 2.6 Hz, 1H), 4.27–4.19 (m, 2H), 4.09–4.03 (m, 1H), 3.52 (dd, J = 9.0, 1.7 Hz, 1H), 3.03 (t, J = 8.3 Hz, 1H), 2.51–2.44 (m, 2H), 2.21 (ddd, J = 16.0, 8.7, 2.9 Hz, 1H), 0.77 (s, 9H). ^13^C {^1^H} NMR (126 MHz, DMSO): δ 181.3, 180.2, 155.2, 140.4, 137.8, 128.5, 127.3, 126.2, 113.1, 64.2, 61.4, 49.4, 41.9, 35.7, 25.5, 23.3. IR ν(cm^–1^): 2968, 2918, 2854, 1743, 1707, 1382, 1176, 727, 695. HRMS (ESI) *m*/*z*: [M + H]^+^ calculated for C_21_H_24_N_2_O_4_, 369.1809; found, 368.1812. [α]_D_^20^: 85.19 (c 0.1, CHCl_3_).

#### (3*aR*,4*R*,7*aS*)-5-((*S*)-4-(*tert*-Butyl)-2-oxooxazolidin-3-yl)-2-methyl-4-phenyl-3*a*,4,7,7*a*-tetrahydro-1*H*-isoindole-1,3(2*H*)-dione (**47**)

Off-white solid. Synthesized by GP2 (0.0233 g, 61%). R_f_ = 0.12 (cyclohexane:EtOAc, 1:1). Melting point: 175–179 °C. Diastereomers separable by column chromatography, dr 73:27. ^1^H NMR (400 MHz, CDCl_3_): δ 7.27–7.15 (m, 3H), 7.12–7.06 (m, 2H), 6.34 (dd, *J* = 5.5, 2.9 Hz, 1H), 4.08 (dd, *J* = 9.1, 3.4 Hz, 1H), 4.03 (d, *J* = 7.2 Hz, 1H), 3.92 (t, *J* = 9.0 Hz, 1H), 3.74 (dd, *J* = 9.0, 3.4 Hz, 1H), 3.36–3.30 (m, 1H), 3.20–3.13 (m, 1H), 3.09 (ddd, *J* = 12.1, 8.6, 3.8 Hz, 1H), 2.81–2.72 (m, 1H), 2.31 (s, 3H), 0.97 (s, 9H). ^13^C {^1^H} NMR (126 MHz, CDCl_3_): δ 178.9, 177.3, 156.8, 134.8, 134.0, 129.1, 128.8, 128.4, 122.6, 122.5, 63.9, 61.6, 45.7, 40.5, 35.9, 35.7, 25.3, 24.9, 23.9, 20.8. IR ν(cm^–1^): 2953, 2916, 2873, 1733, 1670, 1404, 1121, 702. HRMS (ESI) *m*/*z*: [M + H]^+^ calculated for C_22_H_26_N_2_O_4_, 383.1965; found, 383.1964. [α]_D_^20^: −277.16 (c 0.1, CHCl_3_).

#### (3*aR*,4*S*,7*aS*)-5-((*S*)-4-(*tert*-Butyl)-2-oxooxazolidin-3-yl)-2-methyl-4-phenyl-3*a*,4,7,7*a*-tetrahydro-1*H*-isoindole-1,3(2*H*)-dione (**48**)

Orange oil. Synthesized by GP2 (0.0195 g, 51%). R_f_ = 0.46 (cyclohexane:EtOAc, 1:2). Diastereomers separable by column chromatography, dr 86:14. ^1^H NMR (500 MHz, CDCl_3_): δ 7.37–7.26 (m, 5H), 5.94 (dd, *J* = 7.9, 3.2 Hz, 1H), 4.69 (s, 1H), 4.19 (t, *J* = 8.4 Hz, 1H), 4.16 (dd, *J* = 9.1, 2.9 Hz, 1H), 3.63 (dd, *J* = 8.0, 2.9 Hz, 1H), 3.40 (dd, *J* = 8.8, 1.6 Hz, 1H), 3.07 (t, *J* = 8.1 Hz, 1H), 2.98 (s, 3H), 2.80 (ddd, *J* = 16.3, 8.0, 1.3 Hz, 1H), 2.54 (ddd, *J* = 16.3, 8.4, 3.2 Hz, 1H), 0.81 (s, 9H). ^13^C {^1^H} NMR (126 MHz, CDCl_3_): δ 179.9, 178.5, 156.6, 139.3, 138.1, 129.1, 127.7, 127.2, 119.4, 64.9, 62.9, 49.9, 44.7, 38.6, 36.1, 27.1, 25.9, 25.5, 24.6. IR ν(cm^–1^): 2959, 2940, 2923, 1738, 1692, 1401, 1247, 1102, 702. HRMS (ESI) *m*/*z*: [M + H]^+^ calculated for C_22_H_26_N_2_O_4_, 383.1965; found, 383.1965. [α]_D_^20^: −77.03 (c 0.1, CHCl_3_).

#### (3*aR*,4*R*,7*aS*)-5-((*S*)-4-(*tert*-Butyl)-2-oxooxazolidin-3-yl)-2,4-diphenyl-3*a*,4,7,7*a*-tetrahydro-1*H*-isoindole-1,3(2*H*)-dione (**49**)

Off-white solid. Synthesized by GP2 (0.0124 g, 28%). R_f_ = 0.30 (cyclohexane:EtOAc, 1:2). Melting point: 261–262 °C. Diastereomers separable by column chromatography, dr 64:36. ^1^H NMR (400 MHz, CDCl_3_): δ 7.30–7.17 (m, 8H), 6.41–6.34 (m, 3H), 4.15 (d, *J* = 7.3 Hz, 1H), 4.09 (dd, *J* = 9.2, 3.4 Hz, 1H), 3.92 (t, *J* = 9.0 Hz, 1H), 3.76 (dd, *J* = 8.9, 3.4 Hz, 1H), 3.51 (dd, *J* = 8.9, 7.4 Hz, 1H), 3.34 (ddd, *J* = 11.4, 9.0, 3.7 Hz, 1H), 3.31–3.24 (m, 1H), 2.92–2.81 (m, 1H), 0.99 (s, 9H). ^13^C {^1^H} NMR (126 MHz, CDCl_3_): δ 178.0, 176.1, 156.8, 135.1, 134.2, 131.3, 129.6, 129.2, 128.9, 128.6, 128.5, 126.3, 122.4, 122.3, 63.9, 61.6, 45.4, 40.6, 36.2, 35.7, 25.3, 20.9. IR ν(cm^–1^): 2967, 2919, 1746, 1707, 1387, 1181, 694. HRMS (ESI) *m*/*z*: [M + H]^+^ calculated for C_27_H_28_N_2_O_4_, 445.2122; found, 445.2120. [α]_D_^20^: −186.41 (c 0.1, CHCl_3_).

#### (3*aR*,4*S*,7*aS*)-5-((*S*)-4-(*tert*-butyl)-2-oxooxazolidin-3-yl)-2,4-diphenyl-3*a*,4,7,7*a*-tetrahydro-1*H*-isoindole-1,3(2*H*)-dione (**50**)

Off-white solid. Synthesized by GP2 (0.0164 g, 37%). R_f_ = 0.30 (cyclohexane:EtOAc, 1:2). Melting point: 261–262 °C. Diastereomers separable by column chromatography, dr 65:35. ^1^H NMR (400 MHz, CDCl_3_): δ 7.29 (dd, J = 4.1, 2.5 Hz, 3H), 7.24 (dd, J = 6.6, 2.8 Hz, 3H), 7.18 (dd, J = 6.6, 2.9 Hz, 2H), 6.42–6.37 (m, 2H), 6.28 (dd, J = 6.6, 2.3 Hz, 1H), 4.22–4.09 (m, 2H), 4.04 (d, J = 7.1 Hz, 1H), 3.65 (dd, J = 9.4, 7.1 Hz, 1H), 3.43 (ddd, J = 11.4, 9.4, 4.4 Hz, 1H), 3.32 (dd, J = 8.2, 3.8 Hz, 1H), 3.26–3.14 (m, 1H), 3.04–2.91 (m, 1H), 0.70 (s, 9H). ^13^C {^1^H} NMR (101 MHz, CDCl_3_): δ 178.6, 175.6, 158.2, 137.1, 131.4, 129.7, 129.1, 128.9, 128.6, 128.4, 126.4, 126.1, 65.4, 63.7, 47.2, 46.0, 37.0, 35.7, 25.7. IR ν(cm^–1^): 2955, 2923, 1742, 1714, 1232, 1175, 746, 694. HRMS (ESI) *m*/*z*: [M + H]^+^ calculated for C_27_H_28_N_2_O_4_, 445.2122; found, 445.2122. [α]_D_^20^: 97.79 (c 0.1, CHCl_3_).

#### (3*aR*,4*R*,7*aS*)-5-((*S*)-2-Oxo-4-phenyloxazolidin-3-yl)-4-phenyl-3*a*,4,7,7*a*-tetrahydro-1*H*-isoindole-1,3(2*H*)-dione (**51**)

Off-white solid. Synthesized by GP2 (0.0257 g, 66%). R_f_ = 0.39 (cyclohexane:EtOAc, 1:2). Melting point: 101–104 °C. Diastereomers separable by column chromatography, dr 82:18. ^1^H NMR (400 MHz, CDCl_3_): δ 7.46 (s, 1H), 7.31–7.24 (m, 1H), 7.21–7.11 (m, 3H), 7.07 (t, *J* = 7.4 Hz, 2H), 7.00–6.91 (m, 2H), 6.88–6.80 (m, 2H), 5.79 (dd, *J* = 6.0, 2.7 Hz, 1H), 5.04 (t, *J* = 8.5 Hz, 1H), 4.63 (d, *J* = 7.4 Hz, 1H), 4.51 (t, *J* = 8.8 Hz, 1H), 3.93 (t, *J* = 8.5 Hz, 1H), 3.23 (dd, *J* = 9.3, 7.4 Hz, 1H), 3.05 (ddd, *J* = 11.3, 9.4, 4.8 Hz, 1H), 2.81–2.70 (m, 1H), 2.65–2.51 (m, 1H). ^13^C {^1^H} NMR (101 MHz, CDCl_3_): δ 179.1, 176.7, 155.8, 136.6, 135.0, 134.9, 129.2, 129.1, 128.9, 128.5, 127.9, 126.8, 116.7, 69.6, 60.2, 46.1, 40.1, 37.2, 20.5. IR ν(cm^–1^): 2921, 2855, 1703, 1358, 1171, 699. HRMS (ESI) *m*/*z*: [M + H]^+^ calculated for C_23_H_20_N_2_O_4_, 389.1496; found, 389.1493. [α]_D_^20^: −127.6 (c 0.1, CHCl_3_).

#### (3*aR*,4*S*,7*aS*)-5-((*S*)-4-(*tert*-Butyl)-2-oxooxazolidin-3-yl)-4-phenyl-3*a*,4,7,7*a*-tetrahydro-1*H*-isoindole-1,3(2*H*)-dione (**52**)

Off-white solid. Synthesized by GP2 (0.0093 g, 24%). R_f_ = 0.35 (cyclohexane:EtOAc, 1:2). Melting point: 159–162 °C. Diastereomers separable by column chromatography, dr 73:17. ^1^H NMR (400 MHz, CDCl_3_): δ 7.32–7.26 (m, 1H), 7.25–7.15 (m, 3H), 7.11 (t, *J* = 7.4 Hz, 2H), 7.01–6.94 (m, 2H), 6.92–6.84 (m, 2H), 5.82 (dd, *J* = 6.0, 2.7 Hz, 1H), 5.07 (t, *J* = 8.5 Hz, 1H), 4.67 (d, *J* = 7.4 Hz, 1H), 4.54 (t, *J* = 8.8 Hz, 1H), 3.97 (t, *J* = 8.5 Hz, 1H), 3.28 (dd, *J* = 9.3, 7.4 Hz, 1H), 3.09 (ddd, *J* = 11.3, 9.3, 4.8 Hz, 1H), 2.87–2.75 (m, 1H), 2.69–2.55 (m, 1H). ^13^C {^1^H} NMR (101 MHz, CDCl_3_): δ 179.0, 176.6, 155.9, 136.8, 135.2, 135.1, 129.4, 129.2, 129.1, 128.7, 128.1, 127.0, 116.8, 69.8, 60.4, 46.3, 40.3, 37.4, 20.7. IR ν(cm^–1^): 2954, 2917, 2854, 1714, 1677, 1401, 1157, 761, 704. HRMS (ESI) *m*/*z*: [M + H]^+^ calculated for C_23_H_20_N_2_O_4_, 389.1496; found, 389.1500. [α]_D_^20^: −55.34 (c 0.1, CHCl_3_).

#### (3*aR*,4*R*,7*aS*)-2-Methyl-5-((*S*)-2-oxo-4-phenyloxazolidin-3-yl)-4-phenyl-3*a*,4,7,7*a*-tetrahydro-1*H*-isoindole-1,3(2*H*)-dione (**53**)

Off-white solid. Synthesized by GP2 (0.0229 g, 57%). R_f_ = 0.36 (cyclohexane:EtOAc, 1:2). Melting point: 52–56 °C. Diastereomers separable by column chromatography, dr 84:16. ^1^H NMR (400 MHz, CDCl_3_): δ 7.33–7.26 (m, 1H), 7.25–7.13 (m, 3H), 7.13–7.07 (m, 2H), 7.02–6.95 (m, 2H), 6.90–6.78 (m, 2H), 5.83 (dd, *J* = 5.8, 2.8 Hz, 1H), 5.06 (t, *J* = 8.5 Hz, 1H), 4.62 (d, *J* = 7.2 Hz, 1H), 4.53 (t, *J* = 8.8 Hz, 1H), 3.96 (t, *J* = 8.5 Hz, 1H), 3.26 (dd, *J* = 8.6, 7.4 Hz, 1H), 2.98 (ddd, *J* = 12.4, 8.7, 4.1 Hz, 1H), 2.93–2.82 (m, 1H), 2.70–2.56 (m, 1H), 2.26 (s, 3H). ^13^C {^1^H} NMR (126 MHz, CDCl_3_): δ 179.3, 177.1, 155.9, 136.9, 135.2, 134.5, 129.3, 129.2, 129.1, 128.5, 127.9, 126.9, 117.2, 69.7, 60.3, 45.5, 40.4, 36.1, 23.8, 20.7. IR ν(cm^–1^): 2921, 2854, 1744, 1696, 1382, 1036, 700. HRMS (ESI) *m*/*z*: [M + H]^+^ calculated for C_24_H_22_N_2_O_4_, 403.1652; found, 403.1651. [α]_D_^20^: −103.01 (c 0.1, CHCl_3_).

#### (3*aR*,4*S*,7*aS*)-2-Methyl-5-((*S*)-2-oxo-4-phenyloxazolidin-3-yl)-4-phenyl-3*a*,4,7,7*a*-tetrahydro-1*H*-isoindole-1,3(2*H*)-dione (**54**)

Yellow oil. Synthesized by GP2 (0.0145 g, 36%). R_f_ = 0.50 (cyclohexane:EtOAc, 1:2). Diastereomers separable by column chromatography, dr 83:17. ^1^H NMR (400 MHz, CDCl_3_): δ 7.33–7.24 (m, 1H), 7.24–7.14 (m, 3H), 7.13–7.07 (m, 2H), 7.02–6.96 (m, 2H), 6.84–6.75 (m, 2H), 5.84 (dd, *J* = 5.8, 2.8 Hz, 1H), 5.06 (t, *J* = 8.5 Hz, 1H), 4.61 (d, *J* = 7.3 Hz, 1H), 4.54 (t, *J* = 8.8 Hz, 1H), 4.01–3.92 (m, 1H), 3.26 (dd, *J* = 8.6, 7.4 Hz, 1H), 2.99 (ddd, *J* = 12.5, 8.8, 4.2 Hz, 1H), 2.92–2.82 (m, 1H), 2.70–2.56 (m, 1H), 2.26 (s, 3H). ^13^C {^1^H} NMR (101 MHz, CDCl_3_): δ 179.3, 177.1, 155.9, 136.9, 135.3, 134.5, 129.3, 129.2, 129.1, 128.9, 128.5, 127.9, 127.0, 126.7, 117.3, 69.7, 60.3, 45.5, 40.4, 36.1, 23.9, 20.7. IR ν(cm^–1^): 2928, 1744, 1695, 1383, 1272, 699. HRMS (ESI) *m*/*z*: [M + H]^+^ calculated for C_24_H_22_N_2_O_4_, 403.1652; found, 403.1651. [α]_D_^20^: −112.07 (c 0.1, CHCl_3_).

#### (3*aR*,4*R*,7*aS*)-5-((*S*)-2-Oxo-4-phenyloxazolidin-3-yl)-2,4-diphenyl-3*a*,4,7,7*a*-tetrahydro-1*H*-isoindole-1,3(2*H*)-dione (**55**)

Off-white solid. Synthesized by GP2 (0.0149 g, 32%). R_f_ = 0.44 (cyclohexane:EtOAc, 1:2). Melting point: 65–69 °C. Inseparable mixture of diastereomers, dr 63:37. Peaks reported for major. ^1^H NMR (400 MHz, CDCl_3_): δ 7.29 (tdd, *J* = 5.5, 4.7, 2.0 Hz, 1H), 7.25–7.18 (m, 6H), 7.18–7.11 (m, 2H), 6.99 (dd, *J* = 7.2, 1.4 Hz, 2H), 6.97–6.89 (m, 2H), 6.38–6.28 (m, 2H), 5.87 (dd, *J* = 5.8, 2.7 Hz, 1H), 5.09 (t, *J* = 8.5 Hz, 1H), 4.76 (d, *J* = 7.3 Hz, 1H), 4.55 (t, *J* = 8.8 Hz, 1H), 4.03–3.91 (m, 1H), 3.44 (dd, *J* = 9.2, 7.3 Hz, 1H), 3.24 (ddd, *J* = 11.6, 9.2, 4.0 Hz, 1H), 3.05–2.94 (m, 1H), 2.80–2.65 (m, 1H). ^13^C {^1^H} NMR (101 MHz, CDCl_3_): δ 178.4, 175.9, 155.9, 136.8, 135.5, 134.8, 129.7, 129.2, 128.9, 128.8, 127.0, 126.3, 117.1, 69.8, 60.4, 45.2, 40.6, 36.4, 20.9. IR ν(cm^–1^): 2921, 2854, 1744, 1696, 1382, 1035, 699. HRMS (ESI) *m*/*z*: [M + H]^+^ calculated for C_29_H_24_N_2_O_4_, 465.1809; found, 465.1808. [α]_D_^20^: −163.64 (c 0.1, CHCl_3_).

#### (3*aR*,4*S*,7*aS*)-5-((*S*)-2-Oxo-4-phenyloxazolidin-3-yl)-2,4-diphenyl-3*a*,4,7,7*a*-tetrahydro-1*H*-isoindole-1,3(2*H*)-dione (**56**)

Off-white solid. Synthesized by GP2 (0.0107 g, 23%). R_f_ = 0.45 (cyclohexane:EtOAc, 1:2). Melting point: 65–69 °C. Inseparable mixture of diastereomers, dr 65:35. Peaks reported for major. ^1^H NMR (400 MHz, CDCl_3_): δ 7.32–7.18 (m, 7H), 7.18–7.11 (m, 2H), 7.02–6.97 (m, 2H), 6.96–6.91 (m, 2H), 6.42–6.28 (m, 2H), 5.87 (dd, *J* = 5.8, 2.7 Hz, 1H), 5.09 (t, *J* = 8.5 Hz, 1H), 4.76 (d, *J* = 7.3 Hz, 1H), 4.56 (t, *J* = 8.8 Hz, 1H), 4.03–3.93 (m, 1H), 3.44 (dd, *J* = 9.2, 7.3 Hz, 1H), 3.24 (ddd, *J* = 11.6, 9.2, 4.1 Hz, 1H), 3.05–2.94 (m, 1H), 2.80–2.66 (m, 1H). ^13^C {^1^H} NMR (101 MHz, CDCl_3_): δ 178.4, 175.9, 155.9, 136.9, 135.5, 134.8, 131.3, 129.7, 129.2, 128.9, 128.9, 128.5, 128.1, 126.9, 126.3, 117.1, 69.8, 60.3, 45.2, 40.6, 36.4, 20.9. IR ν(cm^–1^): 2964, 2918, 2851, 1743, 1707, 1381, 1176, 728, 695. HRMS (ESI) *m*/*z*: [M + H]^+^ calculated for C_29_H_24_N_2_O_4_, 465.1809; found, 465.1809. [α]_D_^20^: −163.64 (c 0.1, CHCl_3_).

#### (3*aR*,4*R*,7*aS*)-5-((*S*)-4-Benzyl-2-oxooxazolidin-3-yl)-4-phenyl-3a,4,7,7a-tetrahydro-1*H*-isoindole-1,3(2*H*)-dione (**57**)

Off-white solid. Synthesized by GP2 (0.0262 g, 65%). R_f_ = 0.42 (cyclohexane:EtOAc, 1:2). Melting point: 99–103 °C. Diastereomers separable by column chromatography, dr 81:19. ^1^H NMR (400 MHz, CDCl_3_): δ 7.33–7.27 (m, 3H), 7.24 (dd, *J* = 5.1, 2.0 Hz, 3H), 7.19–7.10 (m, 2H), 7.07–6.99 (m, 2H), 6.05 (dd, *J* = 5.9, 2.7 Hz, 1H), 4.52 (d, *J* = 7.5 Hz, 1H), 4.35–4.24 (m, 1H), 4.11 (t, *J* = 8.5 Hz, 1H), 3.90 (dd, *J* = 8.8, 6.3 Hz, 1H), 3.34 (dd, *J* = 9.3, 7.5 Hz, 1H), 3.26–3.15 (m, 1H), 3.13–3.02 (m, 1H), 2.93 (dd, *J* = 13.8, 4.7 Hz, 1H), 2.85–2.71 (m, 1H), 2.26–2.15 (m, 1H). ^13^C {^1^H} NMR (101 MHz, CDCl_3_ δ 179.1, 176.7, 155.8, 135.8, 135.4, 135.4, 129.3, 129.2, 129.1, 128.9, 128.4, 127.4, 118.4, 66.9, 56.5, 46.1, 40.7, 38.8, 37.4, 31.1, 21.0. IR ν(cm^–1^): 2955, 1754, 1737, 1399, 1028, 742, 701. HRMS (ESI) *m*/*z*: [M + H]^+^ calculated for C_24_H_22_N_2_O_4_, 403.1652; found, 403.1649. [α]_D_^20^: −147.47 (c 0.1, CHCl_3_).

#### (3*aR*,4*S*,7*aS*)-5-((*S*)-4-Benzyl-2-oxooxazolidin-3-yl)-4-phenyl-3*a*,4,7,7*a*-tetrahydro-1*H*-isoindole-1,3(2*H*)-dione (**58**)

Clear oil. Synthesized by GP2 (0.0088 g, 22%). R_f_ = 0.44 (cyclohexane:EtOAc, 1:2). Diastereomers separable by column chromatography, dr 71:29. ^1^H NMR (500 MHz, CDCl_3_): δ 7.30 (t, *J* = 7.2 Hz, 2H), 7.26–7.23 (m, 4H), 7.18 (s, 1H), 7.15 (dd, *J* = 7.6, 1.9 Hz, 2H), 7.04 (d, *J* = 7.0 Hz, 2H), 6.06 (dd, *J* = 6.0, 2.7 Hz, 1H), 4.53 (d, *J* = 7.5 Hz, 1H), 4.34–4.25 (m, 1H), 4.16–4.08 (m, 1H), 3.91 (dd, *J* = 8.8, 6.3 Hz, 1H), 3.34 (dd, *J* = 9.3, 7.6 Hz, 1H), 3.25–3.17 (m, 1H), 3.13–3.04 (m, 1H), 2.93 (dd, *J* = 13.8, 4.7 Hz, 1H), 2.84–2.73 (m, 1H), 2.25–2.16 (m, 1H). ^13^C {^1^H} NMR (101 MHz, CDCl_3_): δ 178.9, 176.4, 155.8, 135.8, 135.4, 135.4, 129.4, 129.2, 129.1, 128.9, 128.4, 127.4, 118.4, 66.9, 56.5, 46.2, 40.7, 38.9, 37.4, 21.0. IR ν(cm^–1^): 2923, 2853, 1704, 1454, 1171, 726, 699. HRMS (ESI) *m*/*z*: [M + H]^+^ calculated for C_24_H_22_N_2_O_4_, 403.1652; found, 403.1653. [α]_D_^20^: −108.61 (c 0.1, CHCl_3_).

#### (3*aR*,4*R*,7*aS*)-5-((*S*)-4-Benzyl-2-oxooxazolidin-3-yl)-2-methyl-4-phenyl-3*a*,4,7,7*a*-tetrahydro-1*H*-isoindole-1,3(2*H*)-dione (**59**)

Off-white solid. Synthesized by GP2 (0.0133 g, 32%). R_f_ = 0.42 (cyclohexane:EtOAc, 1:2). Melting point: 56–60 °C. Diastereomers separable by column chromatography, dr 80:20. ^1^H NMR (400 MHz, CDCl_3_): δ 7.34–7.20 (m, 6H), 7.07 (dd, *J* = 7.7, 1.6 Hz, 2H), 7.03 (d, *J* = 6.9 Hz, 2H), 6.05 (dd, *J* = 5.7, 2.5 Hz, 1H), 4.47 (d, *J* = 7.3 Hz, 1H), 4.33–4.22 (m, 1H), 4.08 (t, *J* = 8.5 Hz, 1H), 3.89 (dd, *J* = 8.8, 6.2 Hz, 1H), 3.36–3.27 (m, 1H), 3.16–3.03 (m, 2H), 2.97–2.88 (m, 1H), 2.84–2.71 (m, 1H), 2.33 (s, 3H), 2.22 (dd, *J* = 13.8, 9.2 Hz, 1H). ^13^C {^1^H} NMR (101 MHz, CDCl_3_): δ 179.3, 177.1, 155.8, 135.8, 135.4, 134.7, 129.22, 129.16, 129.1, 128.7, 128.2, 127.4, 118.8, 66.8, 56.4, 45.4, 40.8, 38.9, 36.2, 23.9, 21.0. IR ν(cm^–1^): 2971, 2922, 1743, 1696, 1065, 700. HRMS (ESI) *m*/*z*: [M + H]^+^ calculated for C_25_H_24_N_2_O_4_, 417.1809; found, 417.1807. [α]_D_^20^: −64.54 (c 0.1, CHCl_3_).

#### (3*aR*,4*S*,7*aS*)-5-((*S*)-4-Benzyl-2-oxooxazolidin-3-yl)-2-methyl-4-phenyl-3*a*,4,7,7*a*-tetrahydro-1*H*-isoindole-1,3(2*H*)-dione (**60**)

Pale yellow oil. Synthesized by GP2 (0.0146 g, 35%). R_f_ = 0.38 (cyclohexane:EtOAc, 1:2). Diastereomers separable by column chromatography, dr 82:18. ^1^H NMR (500 MHz, CDCl_3_): δ 7.37–7.27 (m, 3H), 7.25–7.16 (m, 3H), 7.11–7.07 (m, 2H), 7.07–7.01 (m, 2H), 6.07 (dd, *J* = 5.7, 2.5 Hz, 1H), 4.53–4.44 (m, 1H), 4.35–4.23 (m, 1H), 4.10 (t, *J* = 8.5 Hz, 1H), 3.91 (dd, *J* = 8.8, 6.2 Hz, 1H), 3.33 (s, 1H), 3.19–3.05 (m, 2H), 2.95 (dd, *J* = 13.8, 4.6 Hz, 1H), 2.84–2.73 (m, 1H), 2.35 (s, 3H), 2.24 (dd, *J* = 13.8, 9.1 Hz, 1H). ^13^C {^1^H} NMR (101 MHz, CDCl_3_): δ 179.3, 177.1, 155.8, 135.8, 135.4, 134.7, 129.2, 129.16, 129.1, 128.7, 128.2, 127.4, 118.8, 66.8, 56.4, 45.4, 40.8, 38.9, 36.2, 23.9, 21.0. IR ν(cm^–1^): 2967, 2920, 1746, 1704, 1385, 1181, 728, 695. HRMS (ESI) *m*/*z*: [M + H]^+^ calculated for C_25_H_24_N_2_O_4_, 417.1809; found, 417.1807. [α]_D_^20^: −81.09 (c 0.1, CHCl_3_).

#### (3*aR*,4*R*,7*aS*)-5-((*S*)-4-Benzyl-2-oxooxazolidin-3-yl)-2,4-diphenyl-3*a*,4,7,7*a*-tetrahydro-1*H*-isoindole-1,3(2*H*)-dione (**61**)

Off-white solid. Synthesized by GP2 (0.0217 g, 52%). R_f_ = 0.55 (cyclohexane:EtOAc, 1:2). Melting point: 85–90 °C. Diastereomers separable by column chromatography, dr 69:31. ^1^H NMR (400 MHz, CDCl_3_): δ 7.35–7.26 (m, 6H), 7.26–7.19 (m, 5H), 7.09–7.01 (m, 2H), 6.45–6.35 (m, 2H), 6.11 (dd, J = 5.7, 2.7 Hz, 1H), 4.62 (d, J = 7.4 Hz, 1H), 4.38–4.27 (m, 1H), 4.11 (t, J = 8.5 Hz, 1H), 3.92 (dd, J = 8.8, 6.2 Hz, 1H), 3.50 (dd, J = 9.2, 7.4 Hz, 1H), 3.35 (ddd, J = 11.5, 9.2, 3.6 Hz, 1H), 3.31–3.21 (m, 1H), 2.97 (dd, J = 13.8, 4.6 Hz, 1H), 2.94–2.83 (m, 1H), 2.25 (dd, J = 13.8, 9.1 Hz, 1H). ^13^C {^1^H} NMR (101 MHz, CDCl_3_): δ 178.4, 175.9, 155.8, 136.0, 135.3, 134.9, 131.4, 129.7, 129.1, 129.1, 129.1, 128.9, 128.6, 128.4, 127.4, 126.3, 118.6, 66.8, 56.4, 45.1, 40.9, 38.8, 36.4, 21.1. IR ν(cm^–1^): 2921, 2853, 1743, 1708, 1380, 1175, 751, 694. HRMS (ESI) *m*/*z*: [M + H]^+^ calculated for C_30_H_26_N_2_O_4_, 479.1965; found, 479.1962. [α]_D_^20^: −159.15 (c 0.1, CHCl_3_).

#### (3*aR*,4*S*,7*aS*)-5-((*S*)-4-Benzyl-2-oxooxazolidin-3-yl)-2,4-diphenyl-3*a*,4,7,7*a*-tetrahydro-1*H*-isoindole-1,3(2*H*)-dione (**62**)

Pale yellow oil. Synthesized by GP2 (0.0167 g, 40%). R_f_ = 0.33 (cyclohexane:EtOAc, 1:2). Diastereomers separable by column chromatography, dr 63:37. ^1^H NMR (400 MHz, CDCl_3_): δ 7.36–7.27 (m, 6H), 7.26–7.19 (m, 5H), 7.09–7.01 (m, 2H), 6.45–6.35 (m, 2H), 6.11 (dd, *J* = 5.7, 2.7 Hz, 1H), 4.62 (d, *J* = 7.4 Hz, 1H), 4.38–4.27 (m, 1H), 4.11 (t, *J* = 8.5 Hz, 1H), 3.92 (dd, *J* = 8.8, 6.2 Hz, 1H), 3.50 (dd, *J* = 9.2, 7.4 Hz, 1H), 3.35 (ddd, *J* = 11.5, 9.2, 3.6 Hz, 1H), 3.31–3.22 (m, 1H), 2.97 (dd, *J* = 13.8, 4.6 Hz, 1H), 2.94–2.83 (m, 1H), 2.25 (dd, *J* = 13.8, 9.1 Hz, 1H). ^13^C {^1^H} NMR (101 MHz, CDCl_3_): δ 178.4, 175.9, 155.8, 136.0, 135.3, 134.9, 129.7, 129.2, 129.1, 129.1, 128.9, 128.6, 128.5, 127.5, 126.3, 118.6, 66.8, 56.4, 45.1, 40.9, 38.9, 36.4, 21.2. IR ν(cm^–1^): 2957, 2854, 1742, 1707, 1190, 727, 693. HRMS (ESI) *m*/*z*: [M + H]^+^ calculated for C_30_H_26_N_2_O_4_, 479.1965; found, 479.1966. [α]_D_^20^: −98.96 (c 0.1, CHCl_3_).

#### (3*S*,4*R*)-4-Nitro-3-phenylcyclohexan-1-one (**63**)

Synthesized by GP3. (0.0072 g, 66%). Previously described.^24^ ee = 92% (determined by chiral SFC, ID Column, 99_1 to 70_30 over 5 min, 3 mL/min flow rate [Heptane/MeOH]). R_t_ = 2.22 min (major), R_t_ = 2.347 min (minor). XRD sample prepared by slow evaporation of the compound in CH_2_Cl_2_ with pentane antisolvent. ^1^H NMR (400 MHz, CDCl_3_): δ 7.38–7.27 (m, 3H), 7.24–7.20 (m, 2H), 5.06 (td, *J* = 10.5, 3.7 Hz, 1H), 3.71 (td, *J* = 10.9, 5.1 Hz, 1H), 2.74–2.66 (m, 3H), 2.64–2.57 (m, 2H), 2.47 (dd, *J* = 10.5, 5.1 Hz, 1H). ^13^C {^1^H} NMR (101 MHz, CDCl_3_): δ 205.7, 138.3, 129.4, 128.4, 127.1, 88.2, 46.9, 45.6, 38.1, 29.7.

#### (3*S*,4*R*)-3-(4-Methoxyphenyl)-4-nitrocyclohexan-1-one (**64**)

Synthesized by GP3. (0.0087, 70%). Previously described.^24^ ee = 80% (determined by chiral SFC, ID Column, 99_1 to 70_30 over 5 min, 3 mL/min flow rate [Heptane/MeCN]). R_t_ = 3.692 min (minor), R_t_ = 4.283 min (major). XRD sample prepared by slow evaporation of the compound in CH_2_Cl_2_ with pentane antisolvent. ^1^H NMR (400 MHz, CDCl_3_): δ 7.13 (d, *J* = 8.7 Hz, 1H), 6.87 (d, *J* = 8.7 Hz, 1H), 4.99 (td, *J* = 10.4, 3.6 Hz, 1H), 3.78 (s, 2H), 3.64 (td, *J* = 10.9, 6.1 Hz, 1H), 2.71–2.63 (m, 2H), 2.63–2.53 (m, 2H), 2.48–2.37 (m, 1H). ^13^C {^1^H} NMR (126 MHz, CDCl_3_): δ 205.2, 134.3, 129.6, 128.5, 88.0, 46.4, 45.5, 38.1, 29.7.

#### (3*S*,4*R*)-3-(4-Chlorophenyl)-4-nitrocyclohexan-1-one (**65**)

Synthesized by GP3 (0.0086 g, 68%). ee = 91% (determined by chiral SFC, ID Column, 99_1 to 70_30 over 5 min, 3 mL/min flow rate [Heptane/MeCN]) R_t_ = 2.96 min (minor), R_t_ = 3.369 min (major). XRD sample prepared by slow evaporation of the compound in CH_2_Cl_2_ with pentane antisolvent. ^1^H NMR (400 MHz, CDCl_3_): δ 7.37–7.29 (m, 2H), 7.21–7.12 (m, 2H), 5.01 (td, *J* = 10.5, 3.7 Hz, 1H), 3.68 (td, *J* = 11.3, 5.4 Hz, 1H), 2.71–2.63 (m, 4H), 2.63–2.53 (m, 3H), 2.46 (dd, *J* = 10.5, 5.4 Hz, 1H). ^13^C {^1^H} NMR (101 MHz, CDCl_3_): δ 205.1, 136.7, 134.4, 129.6, 128.5, 88.0, 46.4, 45.5, 38.1, 29.7. IR ν(cm^–1^): 3674, 2960, 2919, 1705, 1544, 830. HRMS (ESI) *m*/*z*: [M + H]^+^ calculated for C_12_H_12_ClNO_3_, 254.0578; found, 254.0576. [α]_D_^20^: −23.11 (c 0.1, CHCl_3_).

#### (3*aR*,7*aS*)-Tetrahydro-1*H*-isoindole-1,3,5(2*H*,4*H*)-trione (**66**)

Off-white solid (0.0068 g, 81%). Melting point: 175–180 °C. XRD sample prepared by slow evaporation of the compound in CH_2_Cl_2_ with pentane antisolvent. ^1^H NMR (400 MHz, d-DMSO): δ 11.30 (s, 1H), 3.26 (dt, *J* = 9.5, 7.1 Hz, 1H), 3.08 (dt, *J* = 9.5, 6.2 Hz, 1H), 2.64–2.46 (m, 2H), 2.23 (dd, *J* = 10.6, 3.8 Hz, 1H), 2.06 (dd, *J* = 7.6, 2.2 Hz, 1H), 2.04–1.92 (m, 2H). ^13^C {^1^H} NMR (101 MHz, d-DMSO): δ δ 209.1, 180.6, 180.4, 54.9, 38.3, 37.3, 36.3, 21.0. IR ν(cm^–1^): 3184, 3077, 2971, 2745, 1782, 1714, 1693, 1174, 966, 822, 793, 590. HRMS (ESI) *m*/*z*: [M + H]^+^ calculated for C_8_H_9_NO_3_, 168.0655; found, 168.0655. [α]_D_^20^: 78.04 (c 0.1, DMSO).

#### (3*aR*,4*S*,7*aS*)-4-Phenyltetrahydro-1*H*-isoindole-1,3,5(2*H*,4*H*)-trione (**67** and **68**)

White solid (0.0085 g, 70%). Purified by trituration with CDCl_3_. Melting point: 196–197 °C. XRD sample prepared by slow evaporation of the compound in CH_2_Cl_2_ with pentane antisolvent. ^1^H NMR (500 MHz, *d-*DMSO): δ 7.29 (t, *J* = 7.4 Hz, 2H), 7.22 (t, *J* = 7.3 Hz, 1H), 7.15 (d, *J* = 7.2 Hz, 2H), 4.07 (d, *J* = 9.5 Hz, 1H), 4.02 (s, 1H), 3.58 (t, *J* = 9.5 Hz, 1H), 3.21 (td, *J* = 9.5, 6.5 Hz, 1H), 2.43–2.27 (m, 2H), 2.17–2.00 (m, 2H). ^13^C {^1^H} NMR (101 MHz, CDCl_3_): δ 208.4, 179.9, 178.8, 137.4, 129.1, 127.9, 126.6, 52.6, 43.7, 36.7, 21.2. IR ν(cm^–1^): 3193, 3069, 2350, 1762, 1693, 1339, 1187, 696. HRMS (ESI) *m*/*z*: [M + H]^+^ calculated for C_14_H_13_NO_3_, 244.0968; found, 224.0968. [α]_D_^20^: −76.28 (c 0.1, *d-*DMSO).

#### Dimethyl (*S*)-4-(4-Isopropyl-2-oxooxazolidin-3-yl)phthalate (**69**)

Orange oil. Synthesized by GP3 (0.0138 g, 86%). ^1^H NMR (400 MHz, CDCl_3_): δ 7.86–7.79 (m, 1H), 7.79–7.72 (m, 2H), 4.51–4.45 (m, 1H), 4.42 (t, J = 8.7 Hz, 1H), 4.28 (dd, J = 8.6, 3.9 Hz, 1H), 3.92 (s, 3H), 3.90 (s, 3H), 2.20 (ddt, J = 10.4, 6.9, 3.5 Hz, 1H), 0.94 (d, J = 7.0 Hz, 3H), 0.83 (d, J = 6.8 Hz, 3H). ^13^C {^1^H} NMR (101 MHz, CDCl_3_): δ 168.1, 167.0, 155.3, 140.0, 134.3, 130.7, 129.2, 128.4, 126.7, 125.4, 122.6, 120.2, 62.6, 59.9, 52.9, 52.8, 27.5, 17.9, 14.2. IR ν(cm^–1^): 2956, 2920, 2850, 1721, 1401, 1255, 1196, 1118, 1070, 773. HRMS (ESI) *m*/*z*: [M + H]^+^ calculated for C_16_H_19_NO_6_, 322.1285; found, 322.1286.

## Data Availability

The data underlying this study are available in the published article and its Supporting Information.

## Supporting Information Available

Detailed procedures for the preparation of 2-amido-1,3-dienes **1**–**4** and 2-amido-1-phenyl-1,3-dienes **5a**–**8b** and their application in Diels–Alder reactions; full spectral (^1^H and ^13^C NMR spectroscopy) characterization data for all novel compounds (PDF)
