# Supplementary material for: Synthesis and Application of Chiral 2-Amido and 1-Phenyl-2-amido Dienes in Diels–Alder Reactions to Access Chiral Cyclic Ketones
Source: J Org Chem. 2024 Nov 5;89(22):16325–39. doi: 10.1021/acs.joc.4c00689 (PMC11574853; doi:10.1021/acs.joc.4c00689)
Supplement: Supplementary file 1 — jo4c00689_si_001.pdf [file jo4c00689_si_001.pdf]

Synthesis and Application of Chiral 2-Amido and 1-Phenyl-2-Amido-Dienes in Diels-Alder Reactions to Access Chiral Cyclic Ketones

Aoibheann O' Connor, Calvin Q. O'Broin, Julia Bruno-Colmenarez and

Patrick J. Guiry

Centre for Synthesis and Chemical Biology, School of Chemistry, University College Dublin, Belfield, Dublin 4, Ireland.

Supporting Information

Table of Contents

Variable Temperature (VT) NMR Study	**S2**
Materials and Instrumentation	**S3**
Procedures	**S5**
References	**S8**
Experimental Section	**S9**
NMR Spectra for Novel Compounds	**S11**
X-Ray Crystallography Data	**S88**
SFC Traces of 63, 64 and 65	**S118**

Variable Temperature (VT) ^{1}H NMR Study (600 MHz, $CDCl_3$)

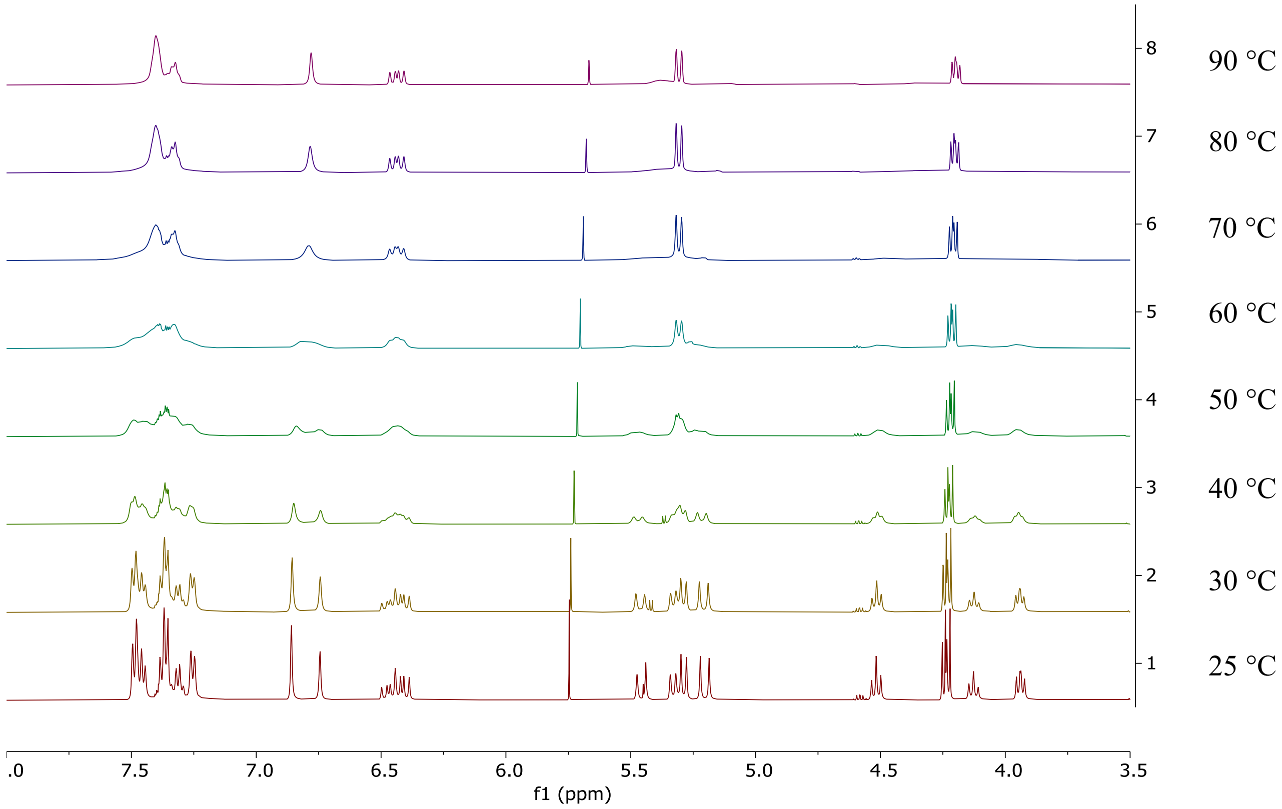

Figure S1: Alkenyl region

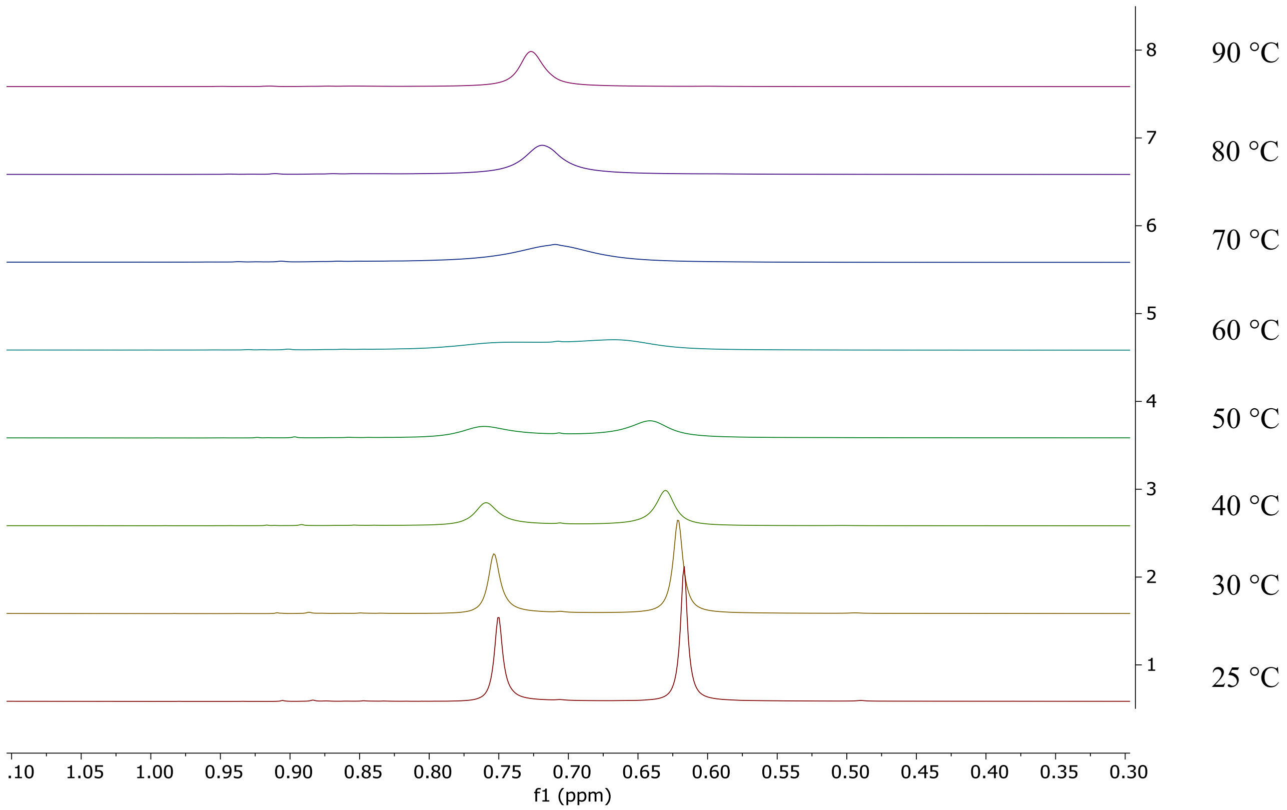

Figure S2: Alkyl region

Materials and Methods

Reagents were obtained from Sigma Aldrich and were used without further purification unless otherwise stated. Reactions were carried out with rigorous exclusion of air and moisture under an inert atmosphere of nitrogen in flame-dried glassware with magnetic stirring unless otherwise stated. N2-flushed plastic syringes were used to transfer air and moisture sensitive reagents. Oxygen free nitrogen was obtained from BOC gases. All anhydrous solvents were obtained from commercial sources and used as received. In vacuo refers to the evaporation of solvent under reduced pressure on a rotary evaporator. Thin-layer chromatography was performed on silica coated aluminium sheets (60 F254) supplied by Merck. Compounds were visualised with UV light (254 nm) fluorescence quenching, or by charring with an acidic vanillin solution (vanillin, H_2SO_4 in ethanol). Flash column chromatography was performed using 40-63 µm, 230-400 mesh silica gel.

Instrumentation

Instrumentation ^{1}H NMR spectra were recorded on Varian Unity 500, 400 and 300 MHz system spectrometers. ^{13}C NMR spectra were recorded on 400 and 500 MHz spectrometers at 101 and 126 MHz. Chemical shifts (δ) are quoted in parts per million (ppm) downfield from tetramethylsilane (TMS) and referenced to residual solvent peaks in the NMR solvent (CDCl3 = δ 7.26 ppm; δ 77.2 ppm). ^{1}H and ^{13}C NMR chemical shift assignments are based on two-dimensional NMR experiments including ^{1}H-gCOSY, DEPT, HSQC and HMBC. All ^{13}C spectra are ^{1}H decoupled. NMR data are represented as follows: chemical shift (δ ppm), multiplicity (s = singlet, d = doublet, t = triplet, q = quartet, dd = double doublet, m = multiplet, app. dd, = apparent doublet of doublets, app. t = apparent triplet), coupling constant (J) in Hertz (Hz), integration. Infrared spectra were recorded on a Bruker Platinum ATR spectrometer and are reported in terms of wavenumbers with units of reciprocal centimetres (cm^{-1}). Melting points were determined in open capillary tubes using a Barnstead electrothermal melting point apparatus. High resolution mass spectra [electrospray ionization (ESI-TOF)] (HRMS) were measured on a micromass LCT orthogonal time-of-flight mass spectrometer with leucine encephalin (Tyr-Gly-Phe-Leu) as an internal lock mass. Racemic reactions were performed using (+/-) 4-isopropyloxazolidin-2-one as the chiral auxiliary. Crystallographic data were obtained utilizing a Rigaku Oxford Diffraction Supernova four-circle diffractometer, formerly manufactured by Agilent Technologies and Oxford Diffraction. This instrument features a micro-focus sealed X-ray tube with a mirror acting as a monochromator, coupled with an Atlas detector. Cu Kα radiation (λ = 1.54184 Å) was employed for all samples, with data integration performed using Crysalispro. Subsequent absorption corrections were carried out using the SCALE3 ABSPACK method, employing either Multi-scan or Gaussian corrections. Structural determination utilised dual methods, initially employing SHELXT for solution followed by refinement via full-matrix least-squares methods against F2 using SHELXL within the Olex2 software suite. Anisotropic displacement parameters were applied to all

non-hydrogen atoms, while hydrogen atoms were refined using isotropic displacement parameters. Some hydrogen atoms were either refined freely while others were placed at calculated positions, adopting a riding model approach with their Uiso values constrained to 1.5 times the Ueq of their pivot atoms for terminal sp3 carbon atoms and 1.2 times for all other carbon atoms.

General Procedure for the preparation of the chiral oxazolidinones

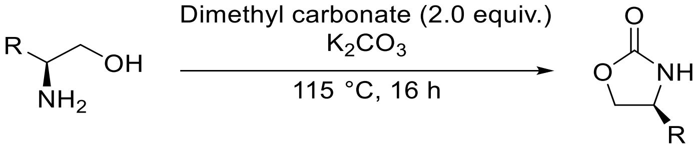

A multi-necked round bottom flask equipped with a stir bar and a Dean-Stark apparatus, and was evacuated and backfilled with N_2 three times. Chiral amino alcohol (10 mmol, 1.0 equiv.) and K_2CO_3 (1 mmol, 0.1 equiv.) were charged to the vessel. Dimethyl carbonate (20 mmol, 2 equiv.) was charged *via* syringe. The reaction mixture was stirred at 115 °C for 18 h under N_2. The solution was diluted with CH_2Cl_2 (10 mL) and washed with equal parts HCl (1.0 M), H_2O and brine. The organic layers were collected, combined, and dried over $MgSO_4$. The filtrate was concentrated *in vacuo* to afford a solid. The solid was recrystallised from EtOAc : pentane.

General Procedure for the preparation of chiral dienes

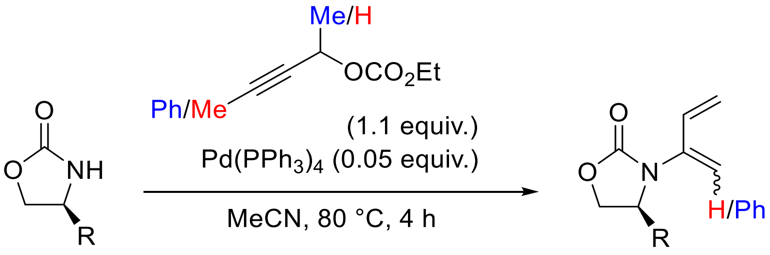

A 10 mL Schlenk flask equipped with a stir bar was evacuated and backfilled with N_2 three times. Chiral oxazolidinone (0.35 mmol, 1.0 equiv.) and $Pd(PPh_3)_4$ (0.0175 mmol, 0.05 equiv.) were charged simultaneously, followed by anhydrous acetonitrile (0.1 M) and propargyl carbonate (0.038 mmol, 1.1 equiv.) *via* syringe. The reaction mixture was stirred at 80 °C over 4 h. The solution was concentrated *in vacuo* and purified by flash column chromatography (3:1 Cyclohexane: EtOAc) to afford the product.

General Procedure for the Diels-Alder reaction

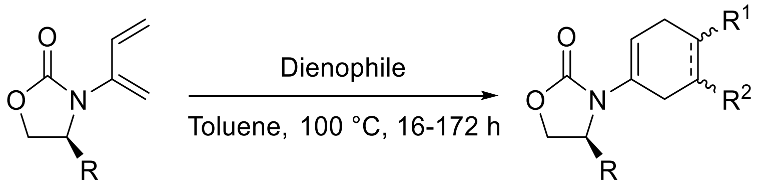

A 10 mL Schlenk flask equipped with a stir bar was evacuated and backfilled with N_2 three times. Chiral diene (0.1 mmol, 1.0 equiv.) was diluted in toluene (3.2 mL) and charged to the vessel *via* syringe followed by dienophile (0.3 mmol, 3.0 equiv.). The reaction mixture was stirred at 100 °C over 16-172 h. The solution was concentrated *in vacuo* and purified by flash column chromatography (3:1 Cyclohexane: EtOAc) to afford the product. For dienophiles such as dimethylacetylene dicarboxylate and maleimide, 16 h was sufficient for the reaction to take place. For dienophiles such as nitrostyrene, reaction times of 172 h were necessary.

General Procedure for the Hydrolysis of the Nitrostyrene Diels-Alder product

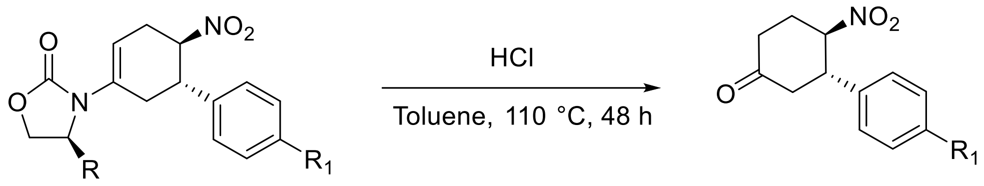

Diels-Alder product (0.05 mmol, 1.0 equiv.) was dissolved in 2 mL Toluene and charged into an oven-dried 15 mL boiling tube equipped with a stir-bar, followed by HCl (1.0 M, 14 equiv.). The biphasic reaction mixture was stirred at 110 °C for 48 h. The aqueous phase was extracted with Toluene (3 x 5 mL) and the organic layers were combined, dried over Na_2SO_4 and concentrated *in vacuo*. The crude hydrolysed product was purified by flash column chromatography (3:1 Cyclohexane : EtOAc) to afford the desired compound.

Procedure for hydrolysis of the Maleimide Diels-Alder product

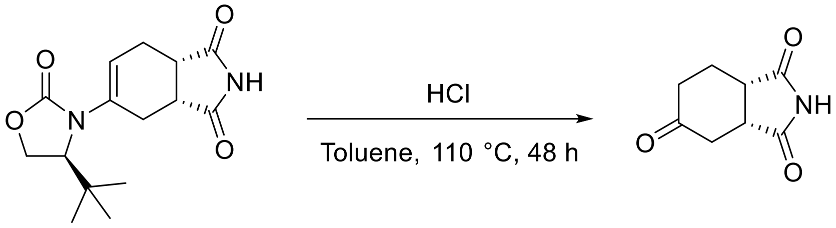

Diels-Alder product (0.05 mmol, 1.0 equiv.) was dissolved in 2 mL Toluene and charged into an oven-dried 15 mL boiling tube equipped with a stir-bar, followed by HCl (1.0 M, 14 equiv.). The biphasic reaction mixture was stirred at 110 °C for 48 h. The organic phase was extracted with H_2O (3 x 5 mL) and the aqueous layers were combined and concentrated *in vacuo* to afford the desired compound without the need for further purification.

Procedure for the hydrolysis of the Ph-substituted Maleimide Diels-Alder product

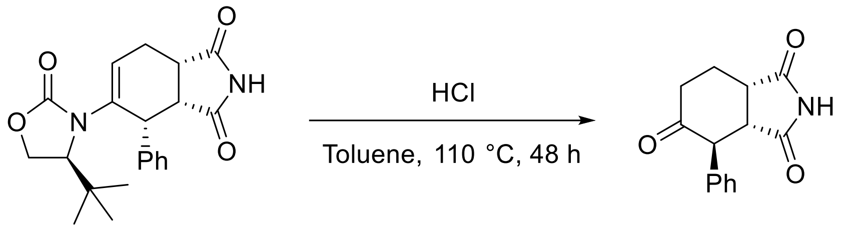

Diels-Alder product (0.05 mmol, 1.0 equiv.) was dissolved in 2 mL Toluene and charged into an oven-dried 15 mL boiling tube equipped with a stir-bar, followed by HCl (1.0 M, 14 equiv.). The biphasic reaction mixture was stirred at 110 °C for 48 h. The aqueous phase was extracted with Toluene (3 x 5 mL) and the organic layers were combined, dried over Na_2SO_4 and concentrated *in vacuo*. The crude hydrolysed product was purified by trituration of the oxazolidinone by-product with $CDCl_3$ to afford the desired compound as a white solid without the need for further purification.

References

[1] Chen R, Li Y, Zhao J, Zheng J, Zhu G, inventors; Gilead Pharmasset LLC, assignee. Process for the preparation of a fluorolacton derivative. United States patent US 9,624,183. 2017 Apr 18.

[2] Evans D. A., Chapman K. T., Bisaha J.;. *J. Am. Chem. Soc*., **1988**, *4*, 1238-56.

[3] O'Broin C.Q., Guiry P.J.; *Org. Lett.*, **2020**, *3,* 879-883.

[4] Hayashi R., Hsung R. P., Feltenberger J. B., Lohse A. G.; *Org. Lett.,* **2009,** *11*, 2125-2128

[5] Crysalispro, 1.171.42.51, 2022, Rigaku OD.

[6] G. M. Sheldrick, *Acta Cryst*. **2015**, A71, 3–8.

[7] G. M. Sheldrick, *Acta Cryst*. **2015**, C71, 3–8.

[8] Dolomanov, O.V.; Bourhis, L.J.; Gildea, R.J.; Howard, J.A.K.; Puschmann, H., OLEX2: A complete structure solution, refinement and analysis program, *J. Appl. Cryst*., **2009**, *42*, 339-341.

[9] Jensen K. L., Weise C. F., Dickmeiss G., Morana F., Davis R. L., Jørgensen K. A,; *Chem. Eur. J.,* **2012**, *38*, 11913-8.

<u>Characterisation data for synthesized substrates:</u>

(4*S*)-4-Isopropyl-1,3-oxazolidin-2-one

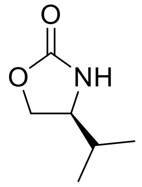

(0.83 g, 64%). Described previously.[1][2]

^{1}H NMR (400 MHz, CDCl$_3$): δ 6.03 (s, 1H), 4.44 (t, *J* = 8.7 Hz, 1H), 4.10 (dd, *J* = 8.7, 6.3 Hz, 1H), 3.65 – 3.55 (m, 1H), 1.73 (dq, *J* = 13.5, 6.8 Hz, 1H), 0.96 (d, *J* = 6.8 Hz, 3H), 0.90 (d, *J* = 6.8 Hz, 3H).

^{13}C {^{1}H} NMR (101 MHz, CDCl$_3$): δ 160.7, 68.6, 58.4, 32.6, 17.9, 17.6

(*S*)-4-*Tert*-butyloxazolidin-2-one

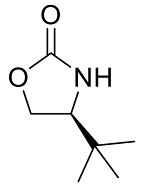

(0.82 g, 57%). Described previously.[1][2]

^{1}H NMR (400 MHz, CDCl$_3$): δ 5.78 (s, 1H), 4.37 (t, *J* = 8.9 Hz, 1H), 4.20 (dd, *J* = 8.9, 5.7 Hz, 1H), 3.59 (ddd, *J* = 8.9, 5.7, 0.8 Hz, 1H), 0.91 (s, 9H).

^{13}C {^{1}H} NMR (101 MHz, CDCl$_3$): δ 160.5, 66.5, 61.5, 33.3, 24.8.

(*S*)-4-Phenyl-2-oxazolidinone

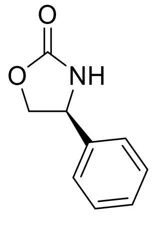

(1.19 g, 73%). Described previously.[1][2]

^{1}H NMR (300 MHz, CDCl3): δ 7.38 (q, J = 7.7 Hz, 5H), 5.20 (bs, 1H), 5.01 – 4.90 (m, 1H), 4.75 (t, J = 8.6 Hz, 1H), 4.21 (dd, J = 8.6, 7.7 Hz, 1H).

^{13}C {^{1}H} NMR (101 MHz, CDCl$_3$): δ 159.7, 139.4, 129.2, 128.8, 126.0, 72.5, 56.4.

(*S*)-4-Benzyl-2-oxazolidinone

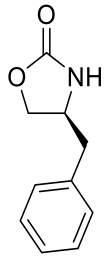

(1.59 g, 90%). Described previously.[1][2]

^{1}H NMR (400 MHz, $CDCl_3$): δ 7.34 (tt, *J* = 8.0, 1.7 Hz, 2H), 7.30 – 7.24 (m, 1H), 7.21 – 7.13 (m, 2H), 5.58 (s, 1H), 4.45 (t, *J* = 8.2 Hz, 1H), 4.15 (dd, *J* = 8.5, 5.5 Hz, 1H), 4.13 – 4.07 (m, 1H), 2.92 – 2.83 (m, 2H).

^{13}C {^{1}H} NMR (101 MHz, $CDCl_3$): δ 159.7, 135.9, 129.0, 128.9, 127.1, 69.5, 53.8, 41.3.

But-2-yn-1-yl ethyl carbonate

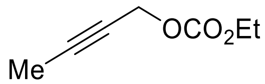

(1.01 mL, 94%). Described previously.[3]

^{1}H NMR (400 MHz, $CDCl_3$): δ 4.70 – 4.63 (m, 2H), 4.25 – 4.14 (m, 2H), 1.89 – 1.81 (m, 3H), 1.34 – 1.25 (m, 3H).

^{13}C {^{1}H} NMR (126 MHz, $CDCl_3$): δ 154.7, 83.9, 72.7, 64.3, 55.9, 14.2, 3.6.

Ethyl (4-phenylbut-3-yn-2-yl) carbonate

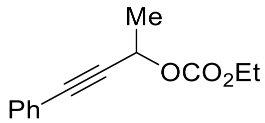

(2.8841 g, 90%). Described previously.[3]

^{1}H NMR (500 MHz, $CDCl_3$): δ 7.47 – 7.40 (m, 2H), 7.34 – 7.28 (m, 3H), 5.55 (q, *J* = 6.7 Hz, 1H), 4.24 (q, *J* = 7.1 Hz, 2H), 1.64 (d, *J* = 6.7 Hz, 3H), 1.33 (t, *J* = 7.1 Hz, 3H).

^{13}C {^{1}H} NMR (101 MHz, $CDCl_3$): δ 154.2, 131.8, 128.7, 128.2, 122.2, 86.7, 85.3, 64.6, 64.2, 21.5, 14.2.

^{1}H NMR (400 MHz, $CDCl_3$)

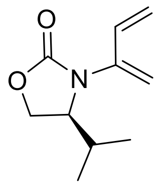

1

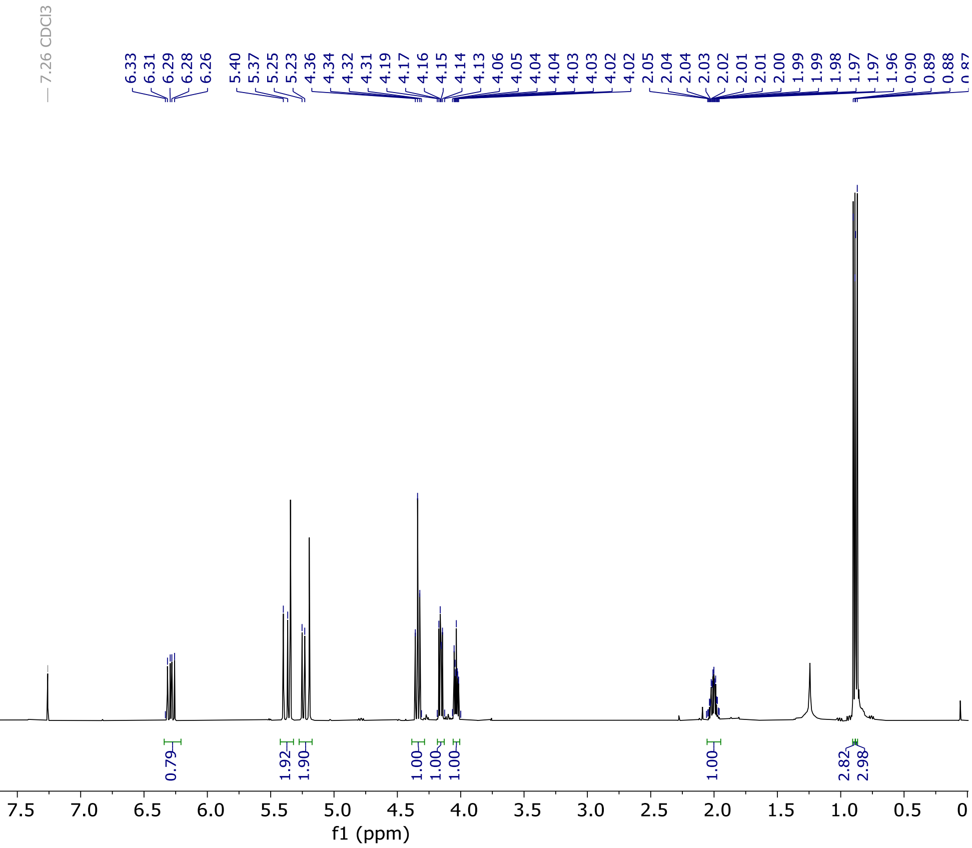

^{13}C {^{1}H} NMR (126 MHz, $CDCl_3$)

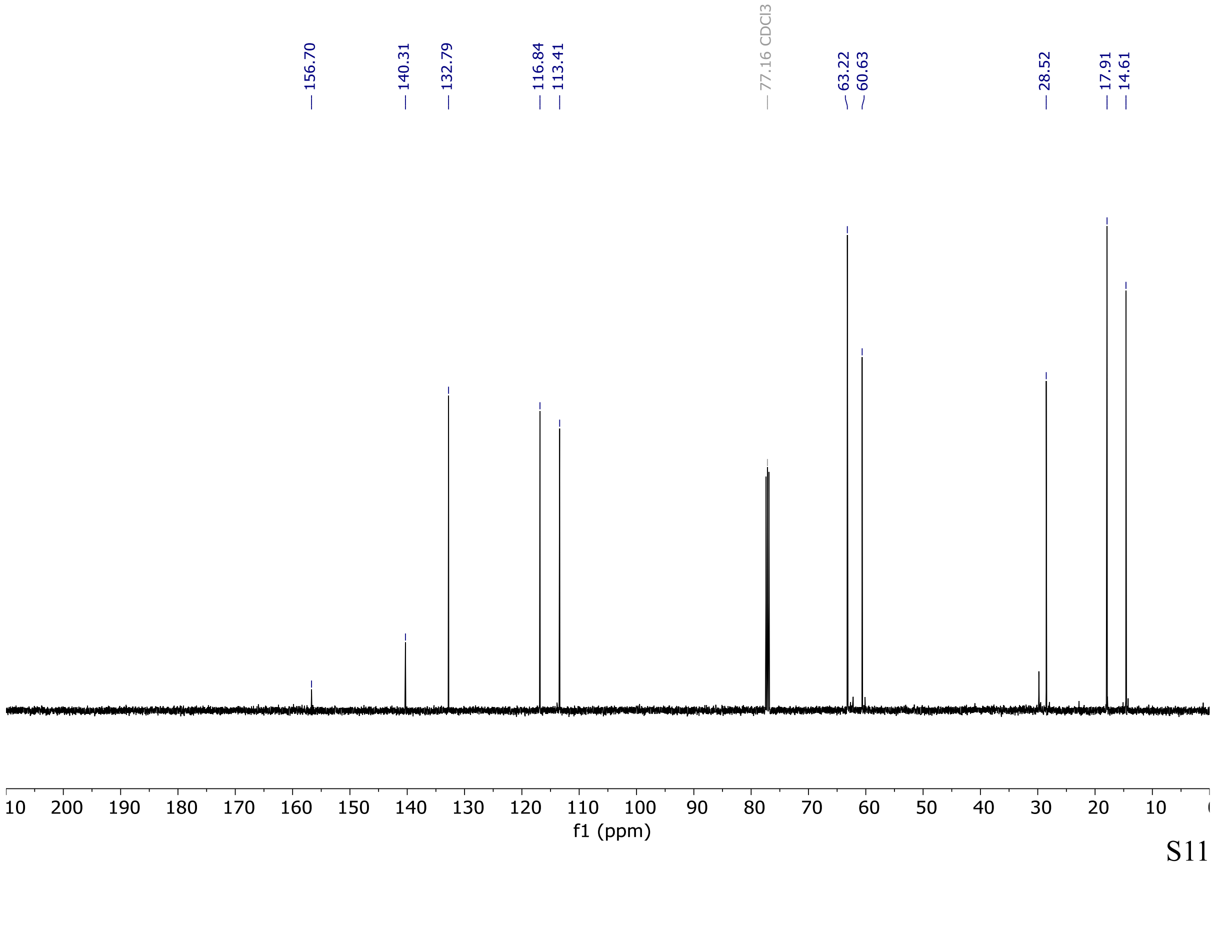

^{1}H NMR (400 MHz, $CDCl_3$)

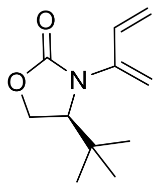

2

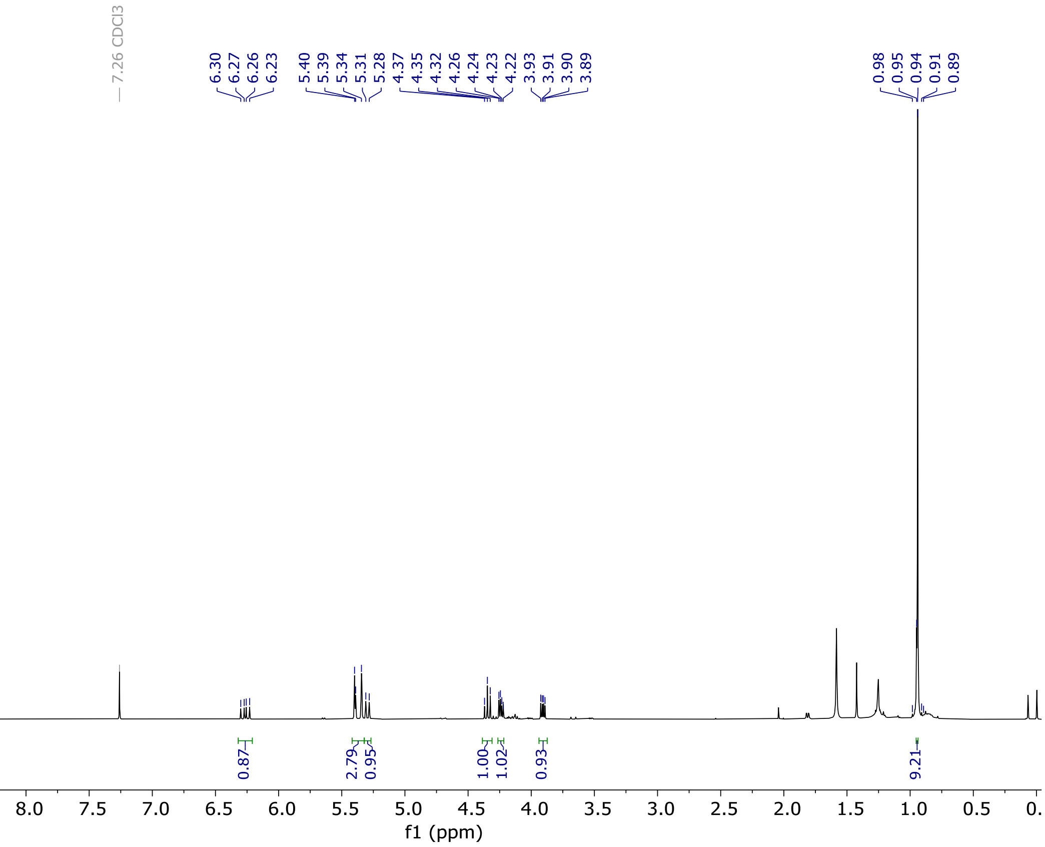

^{13}C {^{1}H} NMR (101 MHz, $CDCl_3$)

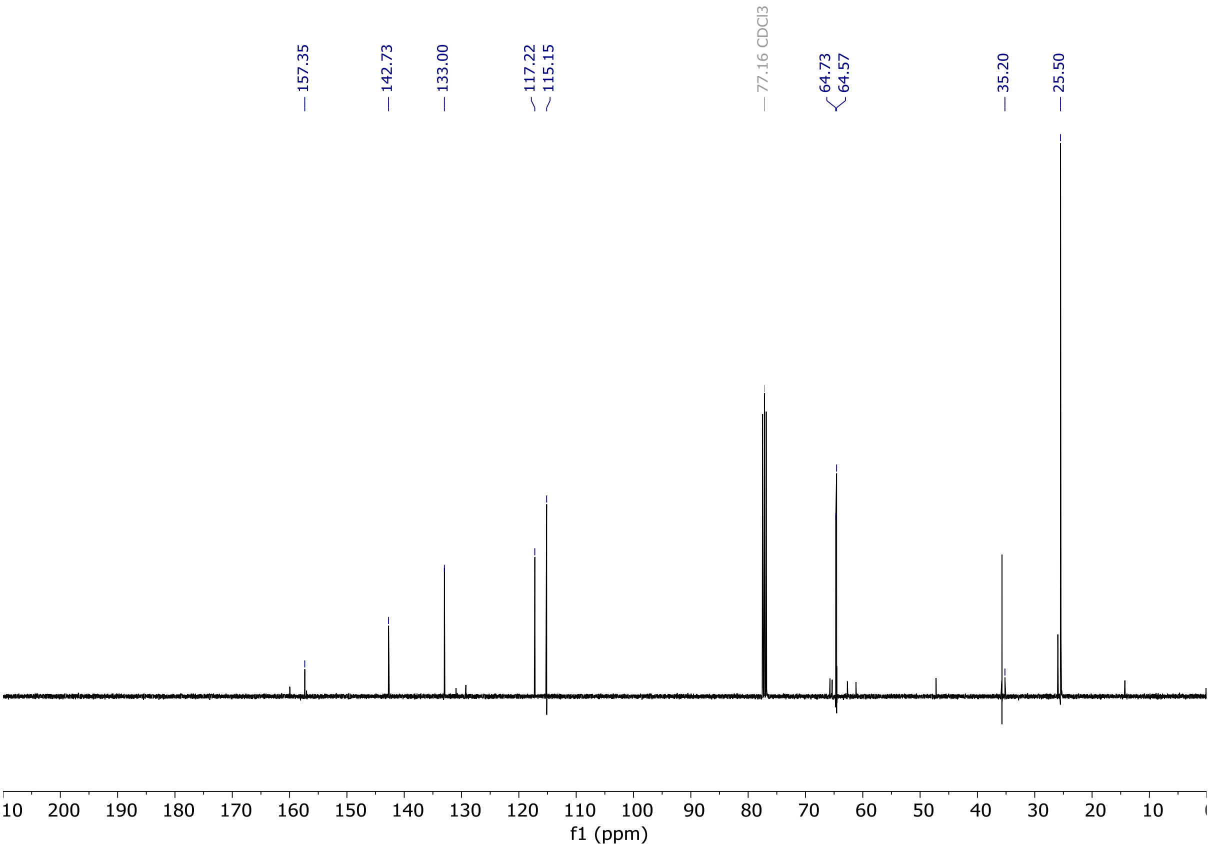

^{1}H NMR (500 MHz, $CDCl_3$)

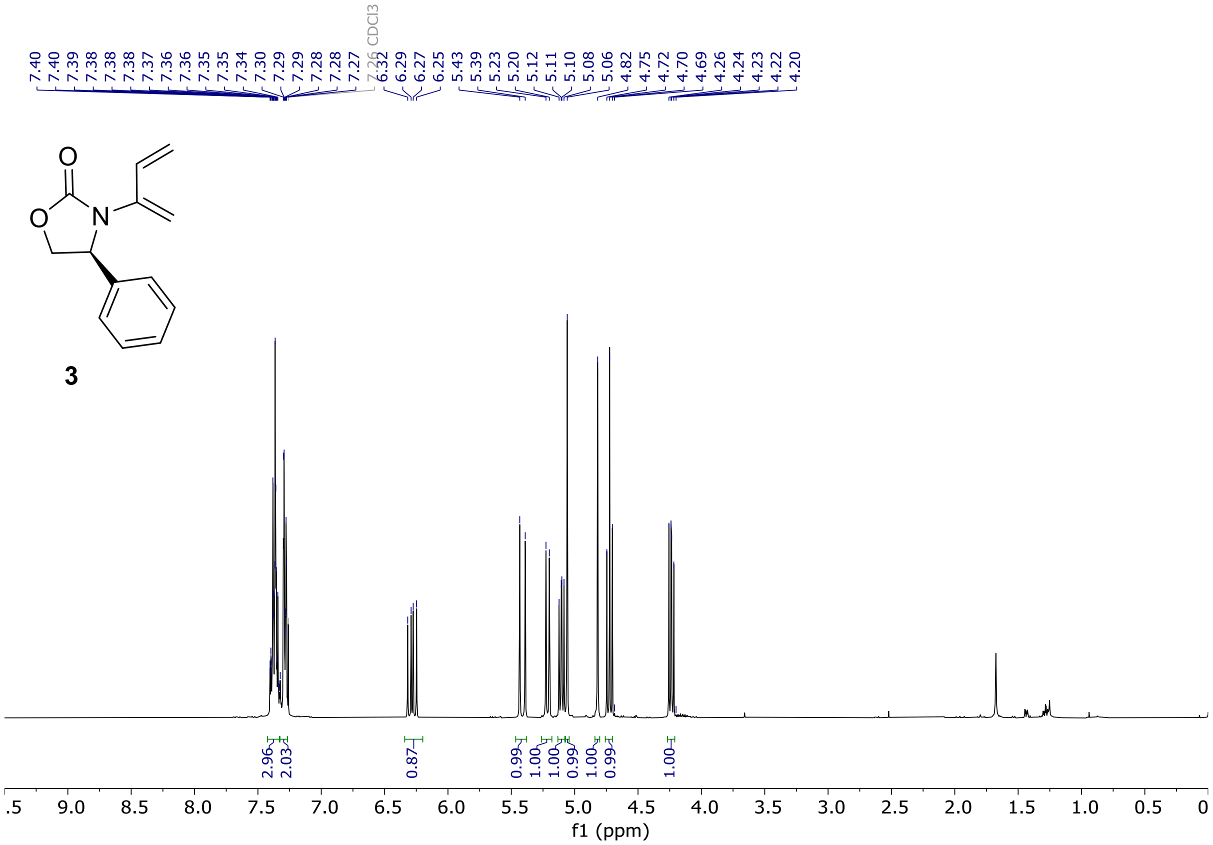

^{13}C {^{1}H} NMR (101 MHz, $CDCl_3$)

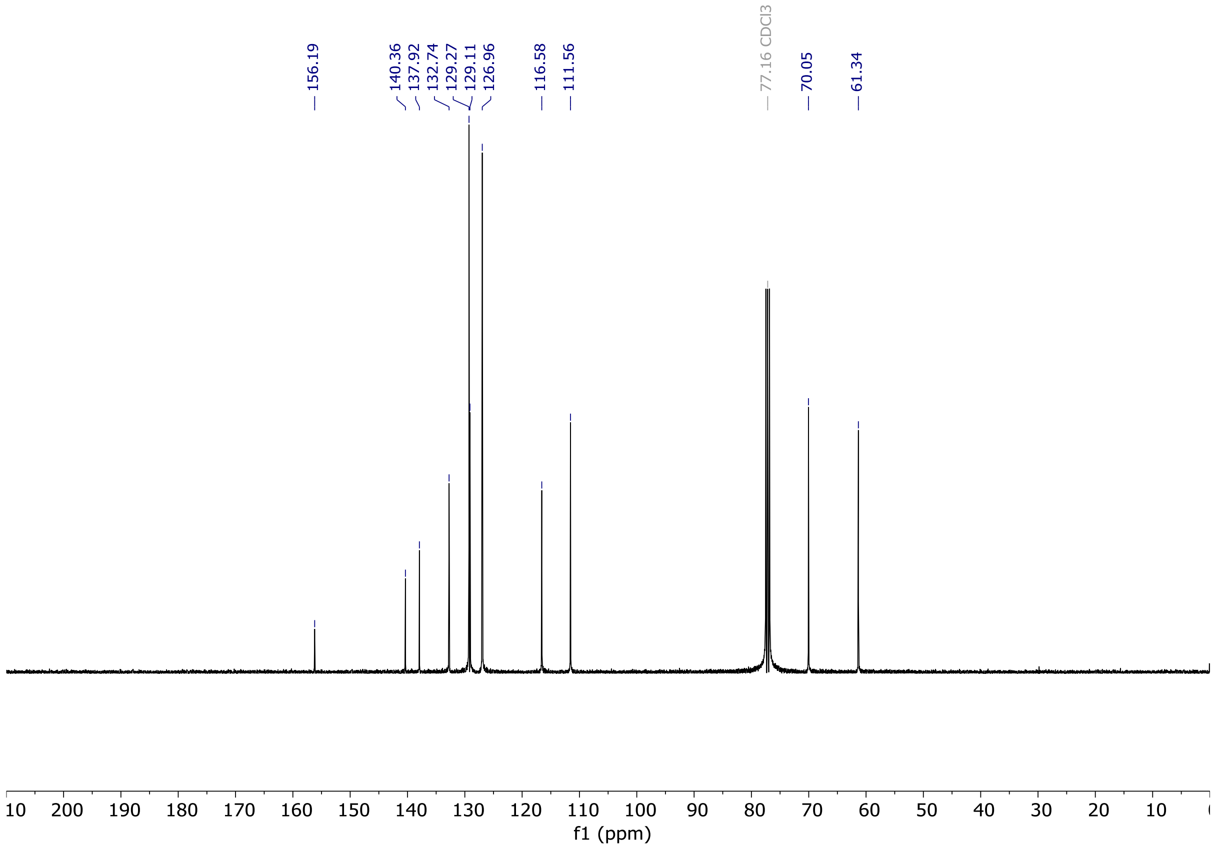

^{1}H NMR (400 MHz, $CDCl_3$)

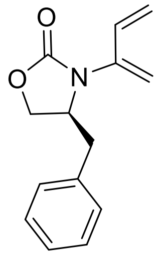

4

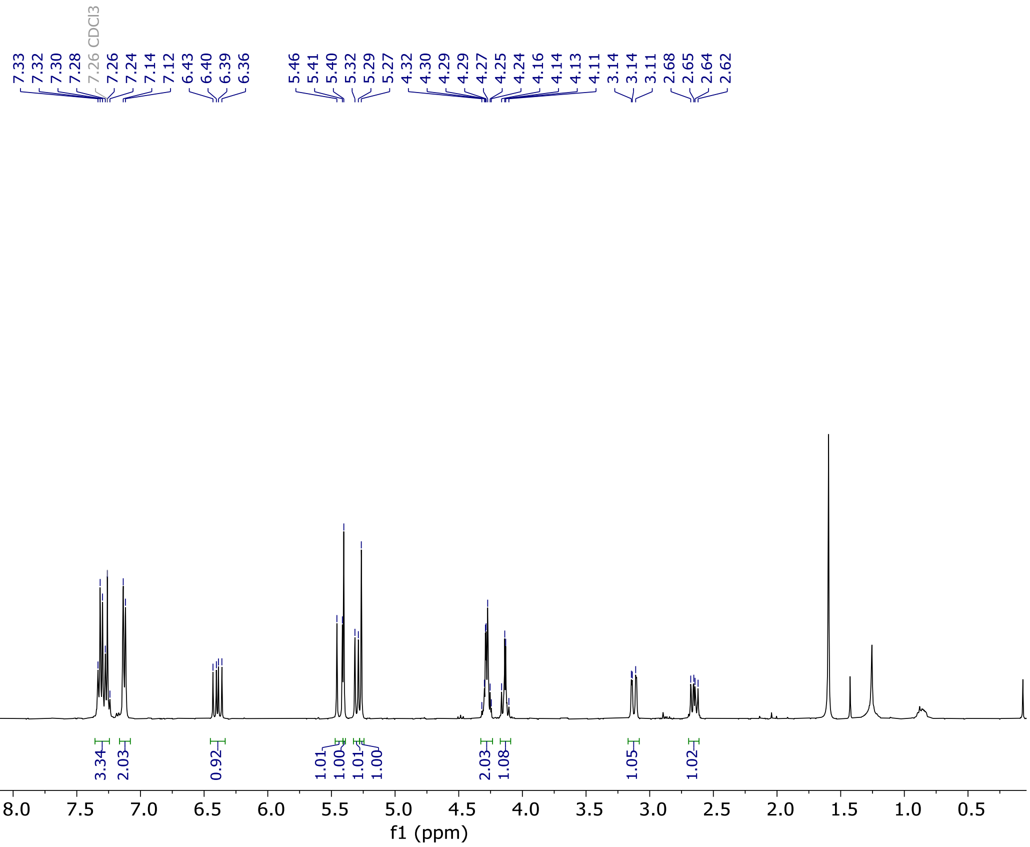

$^{13}C\{^{1}H\}$ NMR (101 MHz, $CDCl_3$)

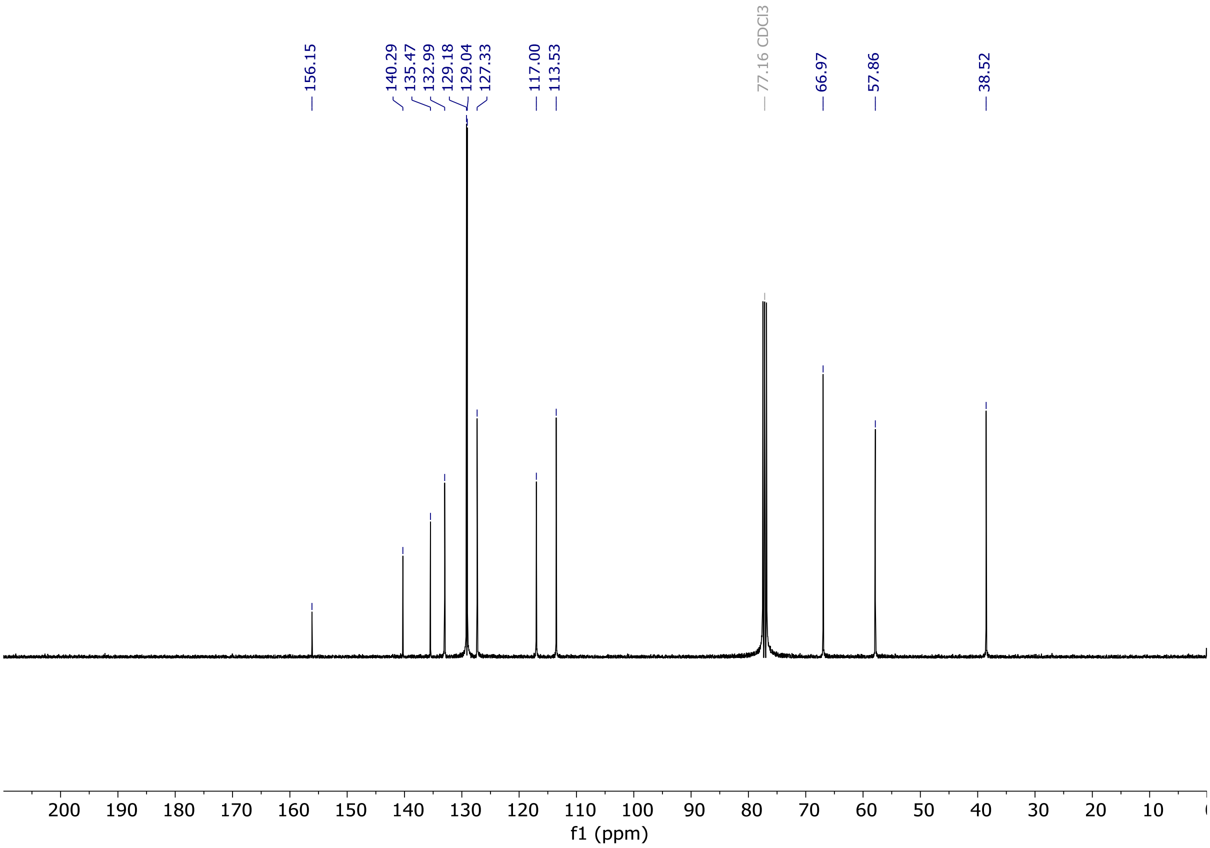

^{1}H NMR (400 MHz, CDCl$_3$)

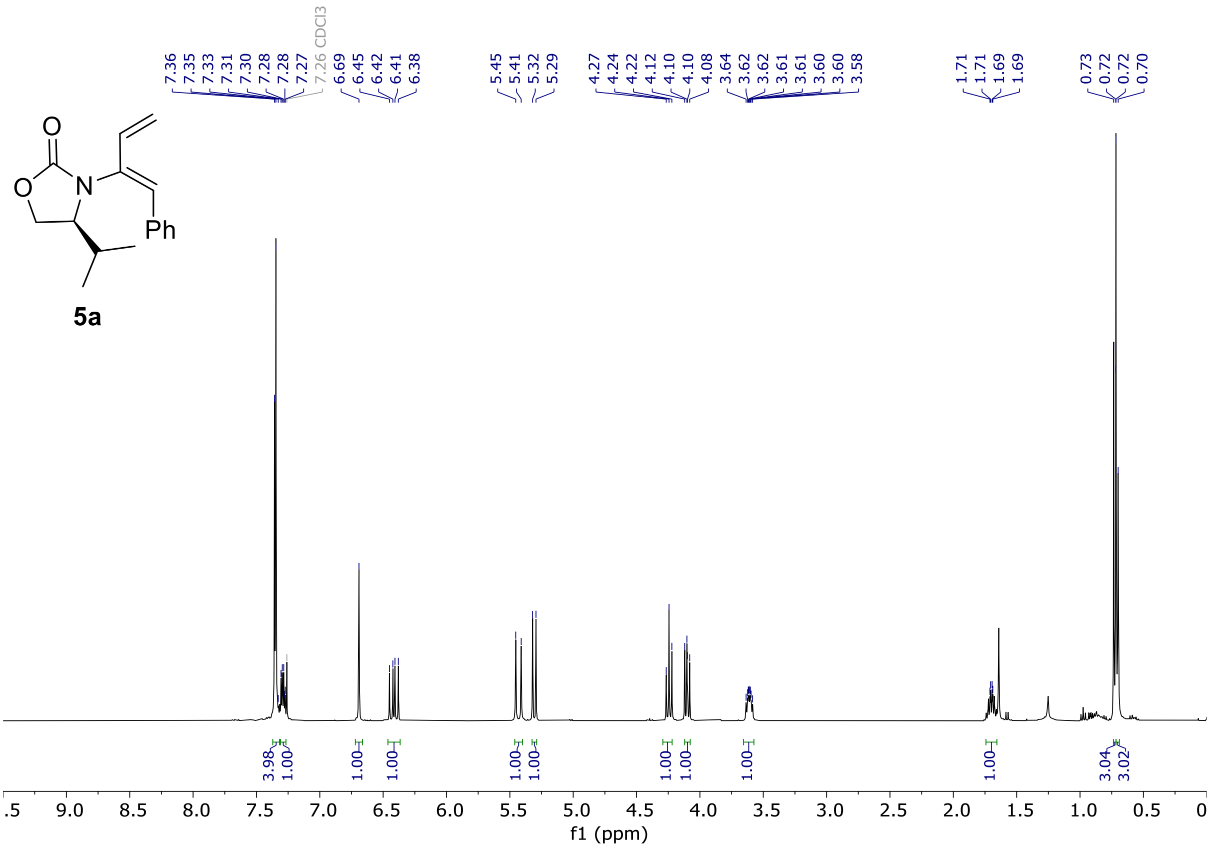

^{13}C {^{1}H} NMR (101 MHz, CDCl$_3$)

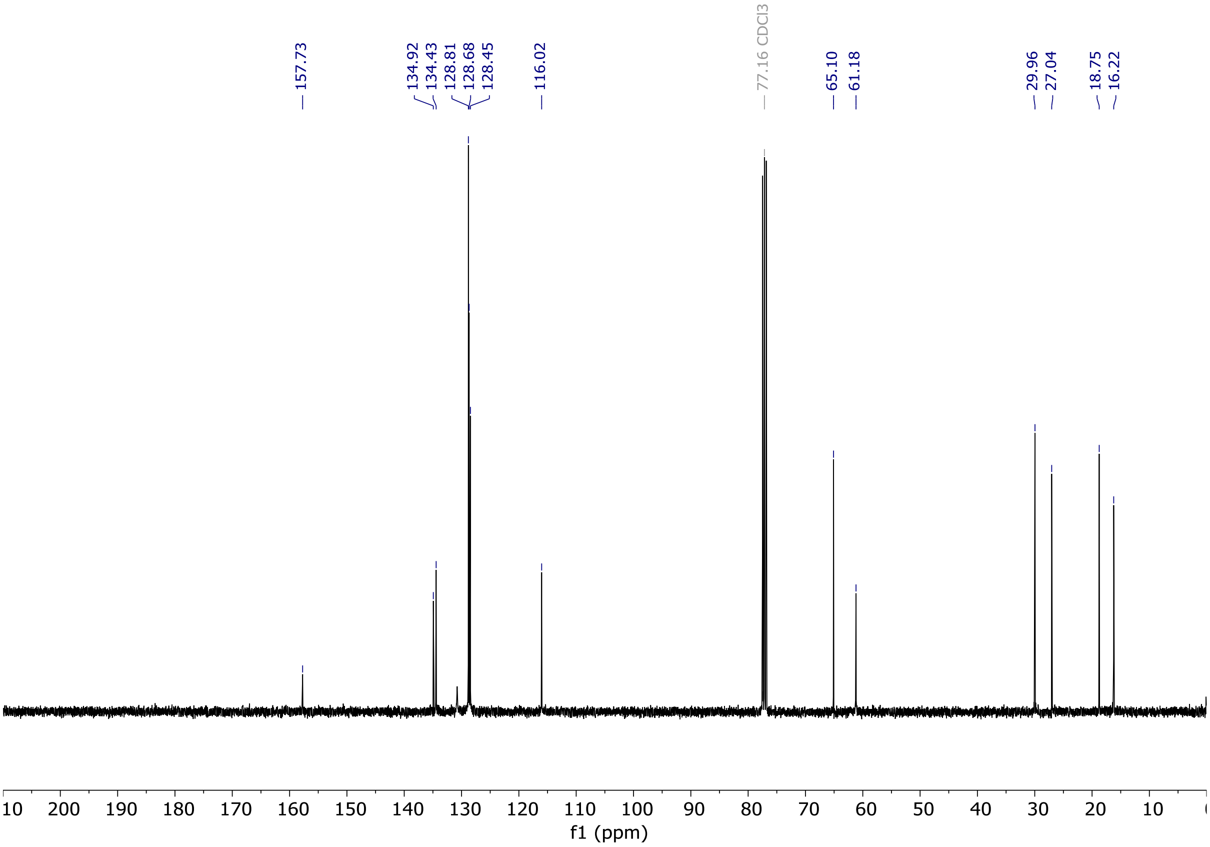

^{1}H NMR (400 MHz, $CDCl_3$)

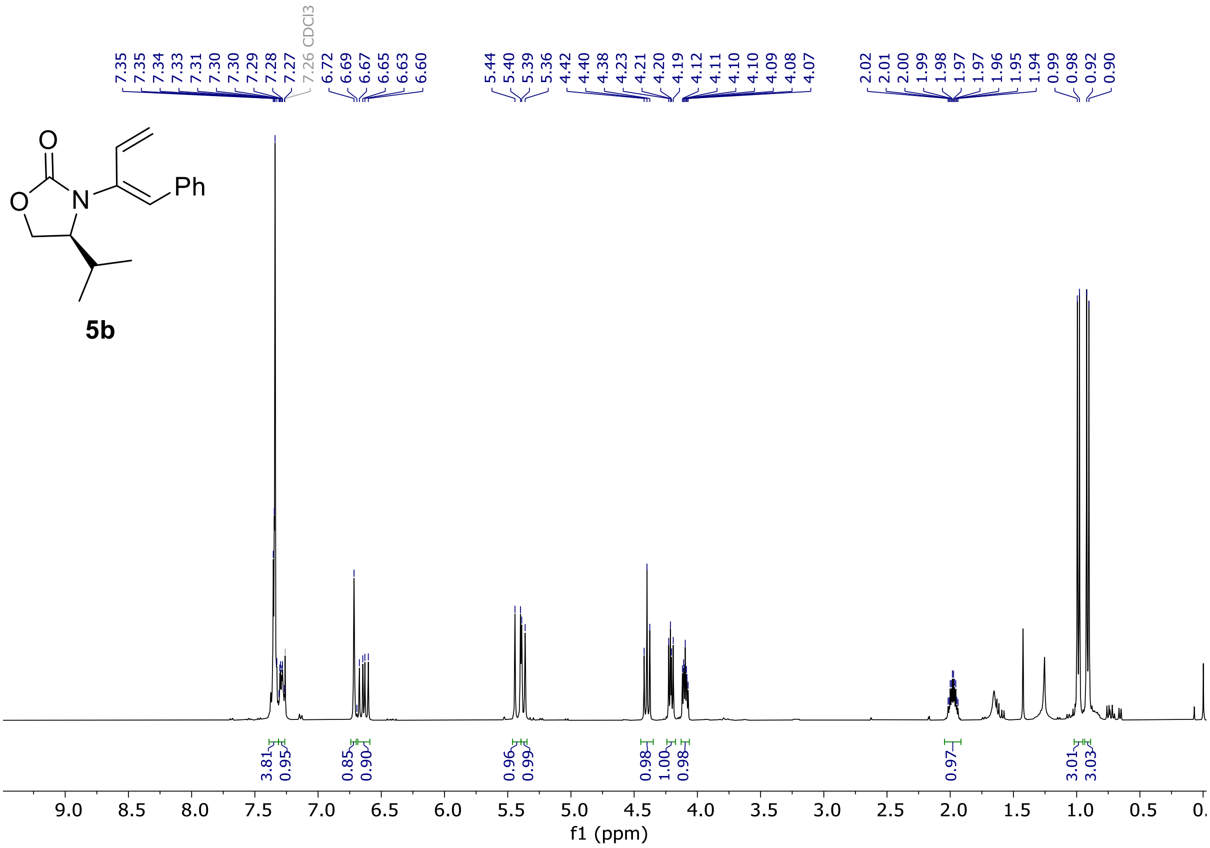

5b

^{13}C {^{1}H} NMR (126 MHz, $CDCl_3$)

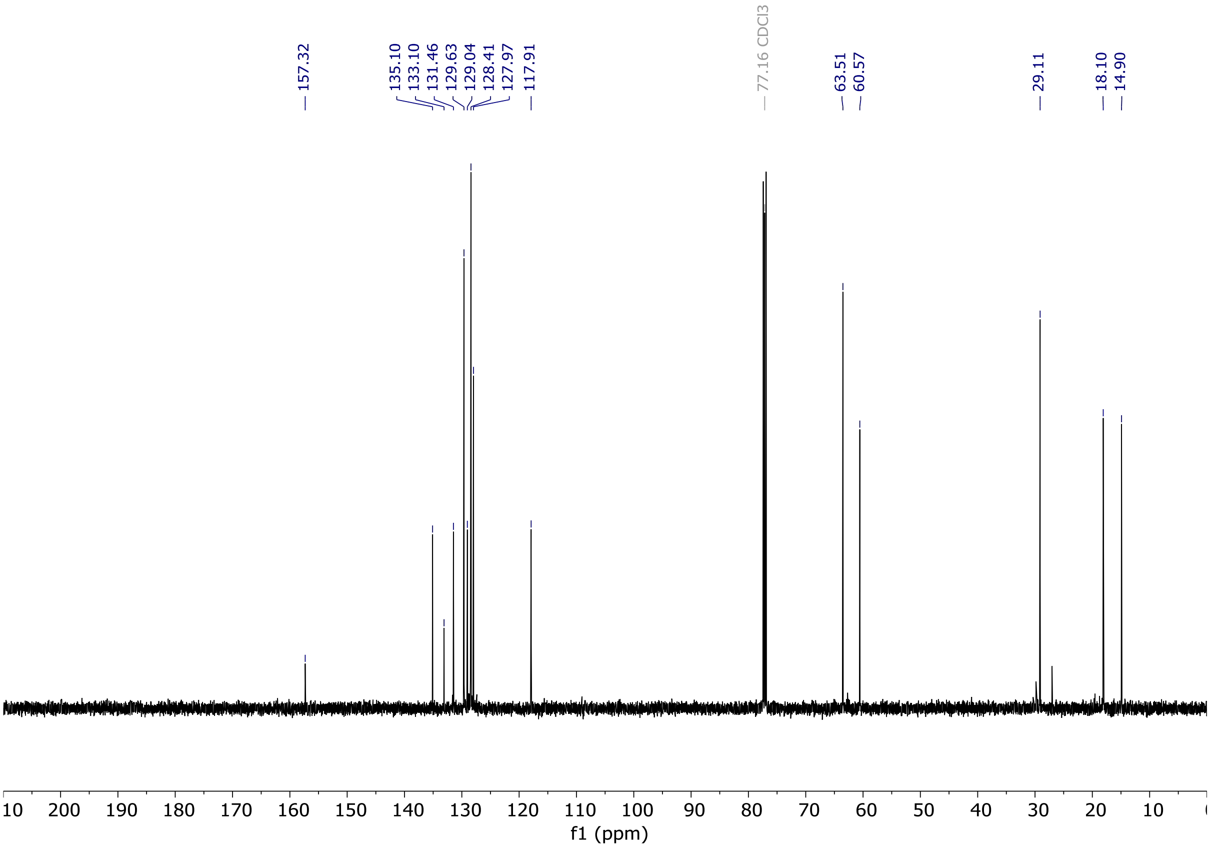

^{1}H NMR (400 MHz, $CDCl_3$)

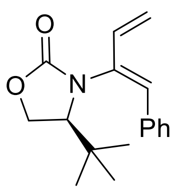

6a

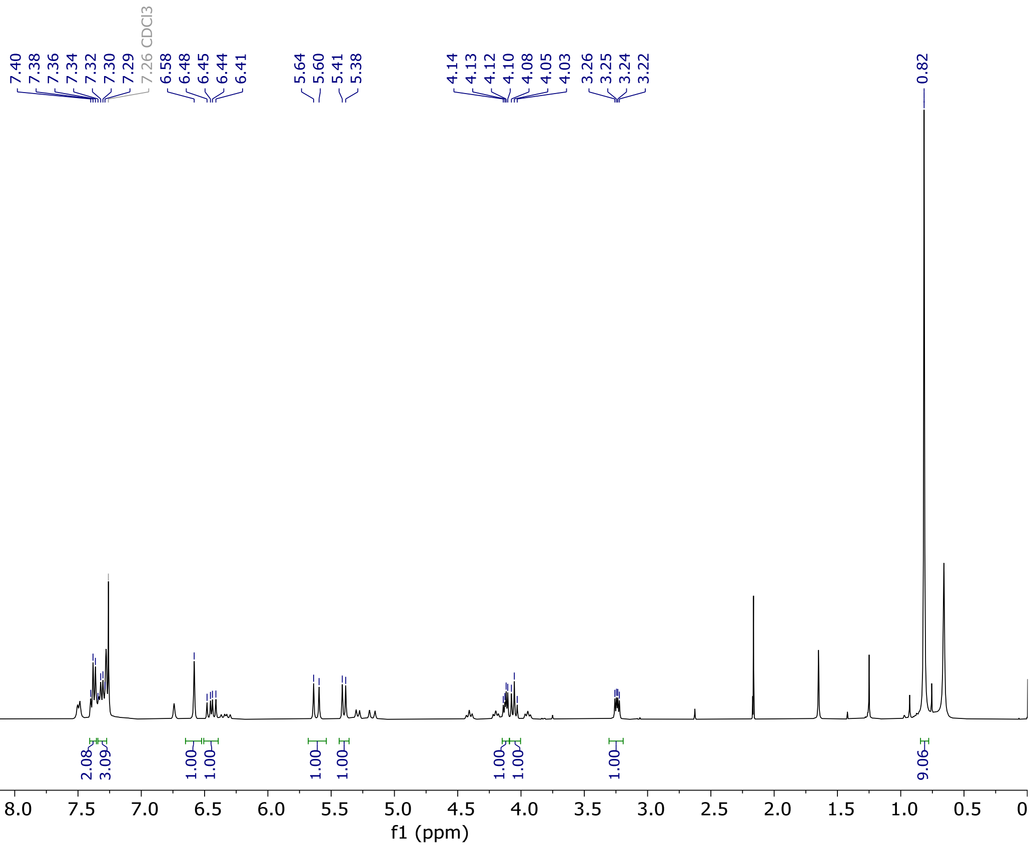

^{13}C {^{1}H} NMR (101 MHz, *d*-DMSO)

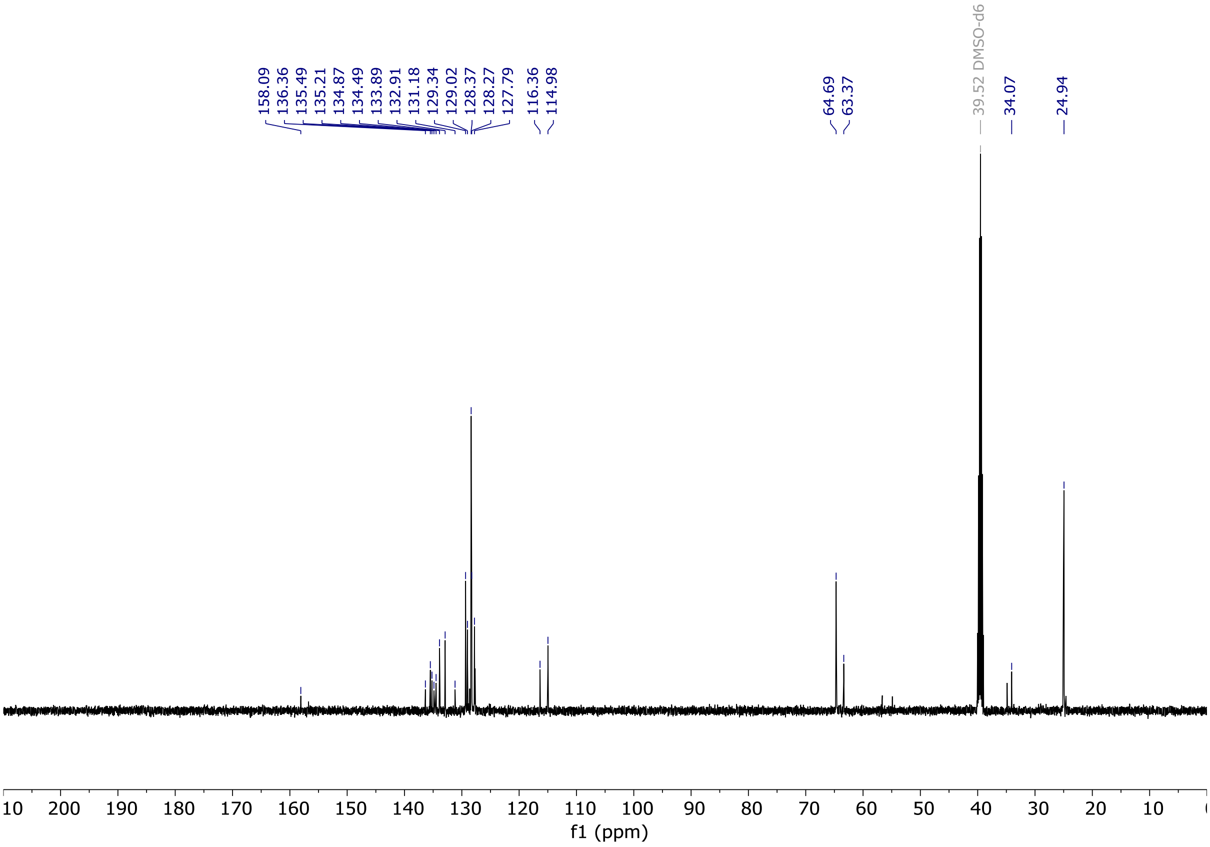

^{1}H NMR (400 MHz, $CDCl_3$)

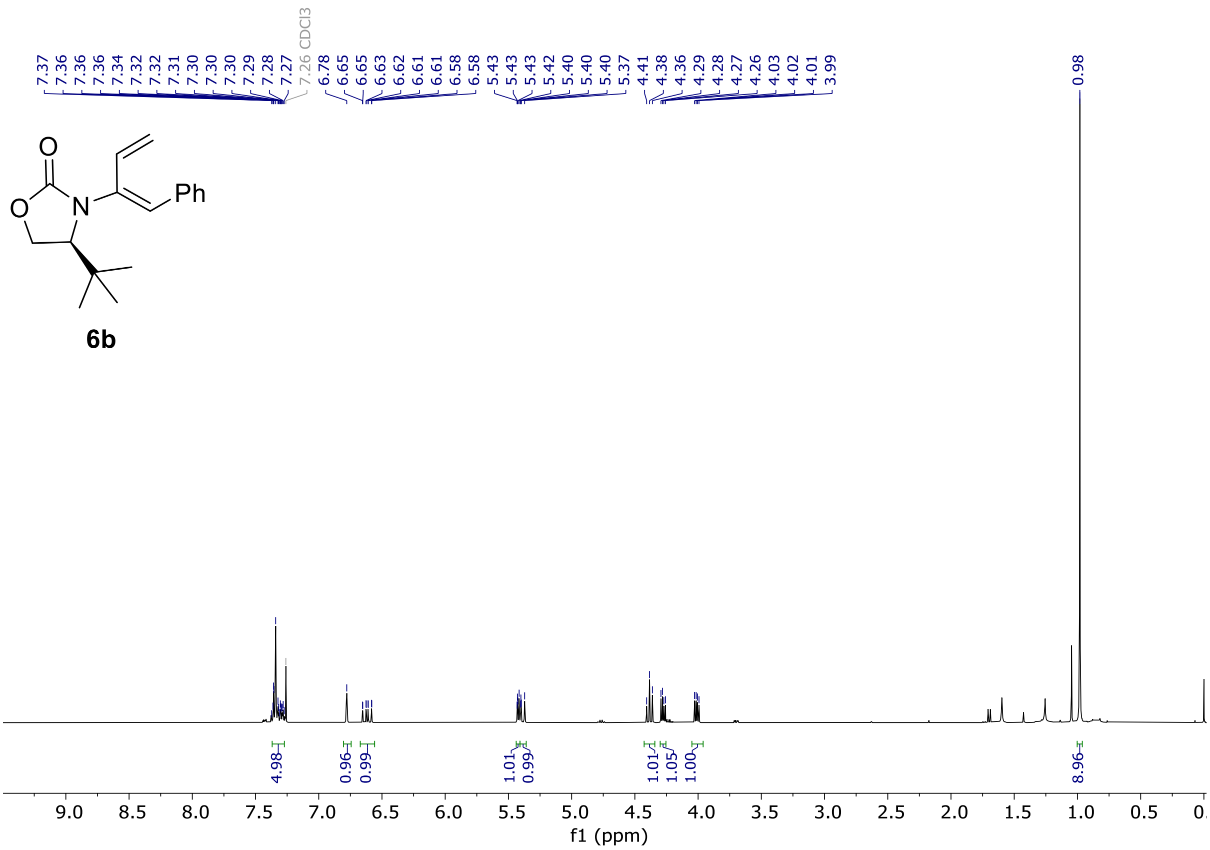

6b

^{13}C {^{1}H} NMR (101 MHz, $CDCl_3$)

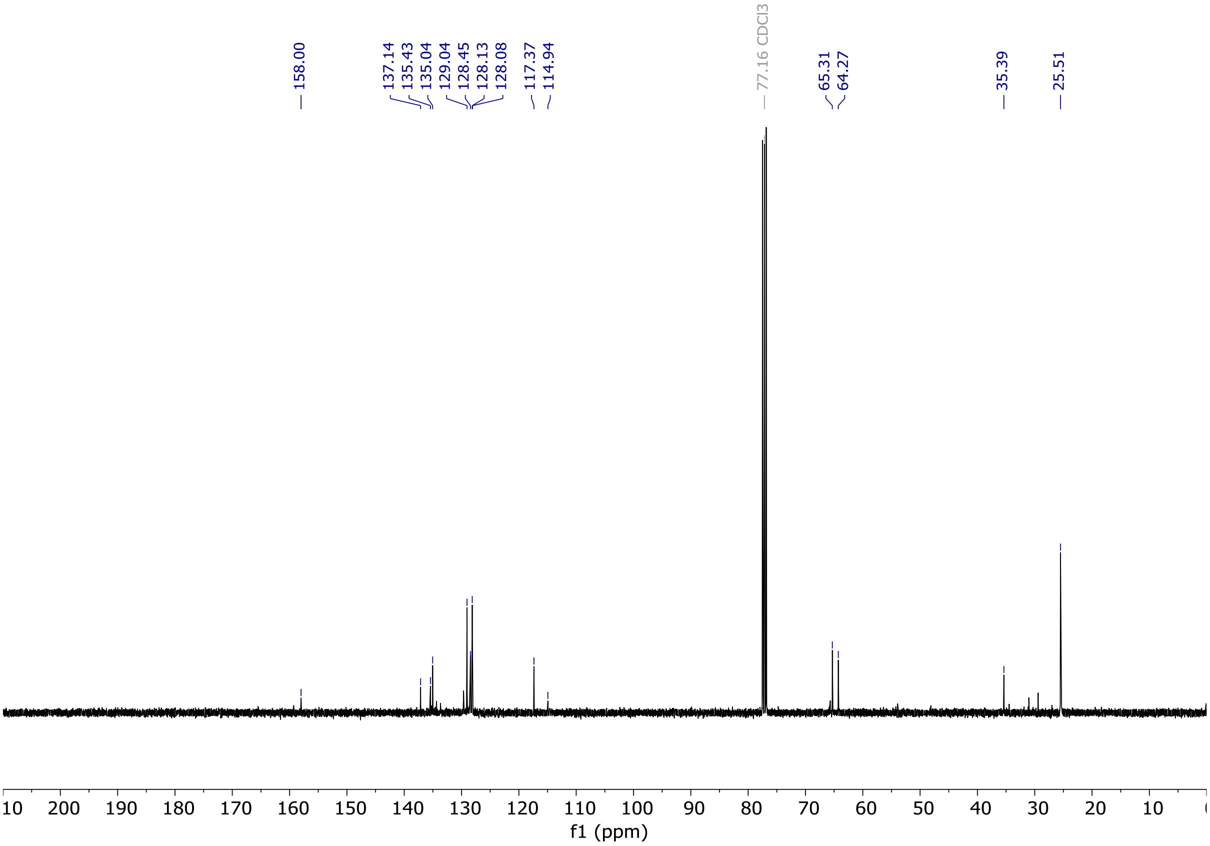

^{1}H NMR (400 MHz, CDCl$_3$)

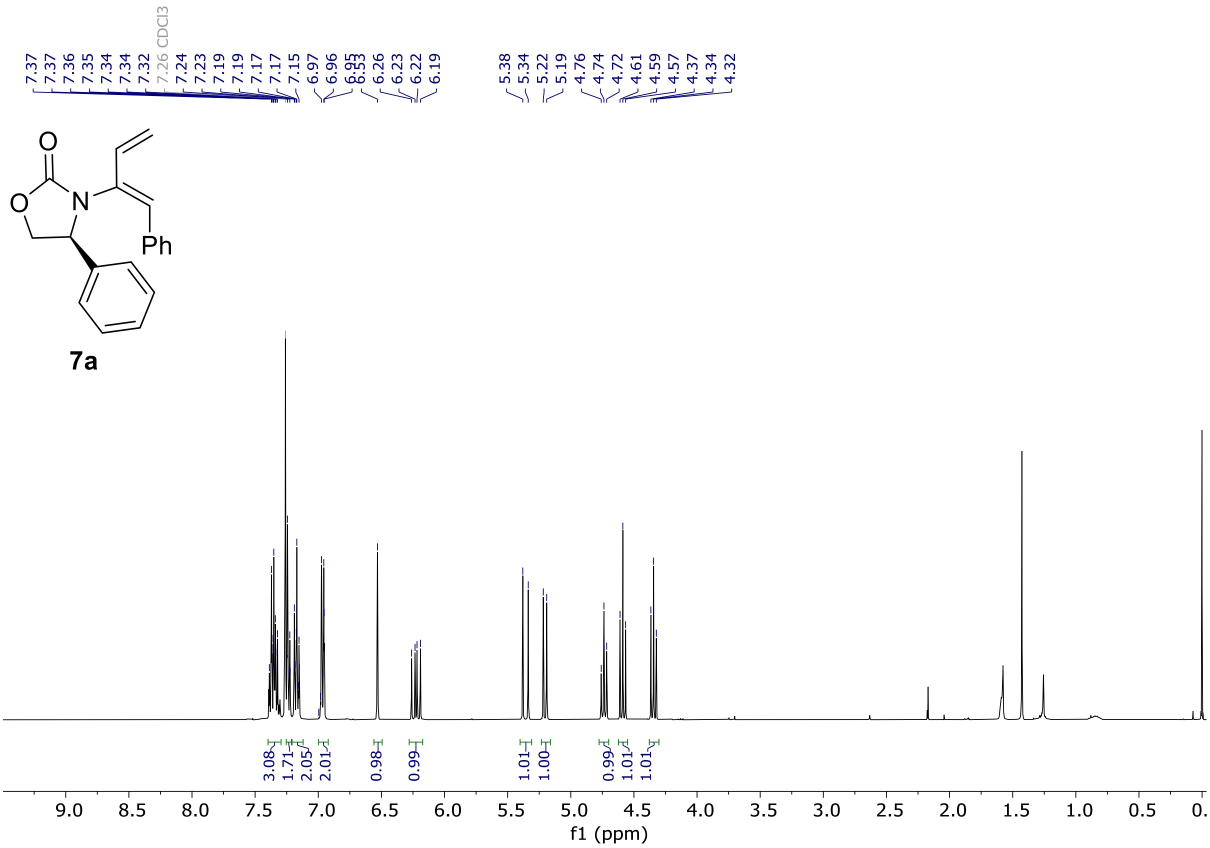

7a

^{13}C {^{1}H} NMR (101 MHz, CDCl$_3$)

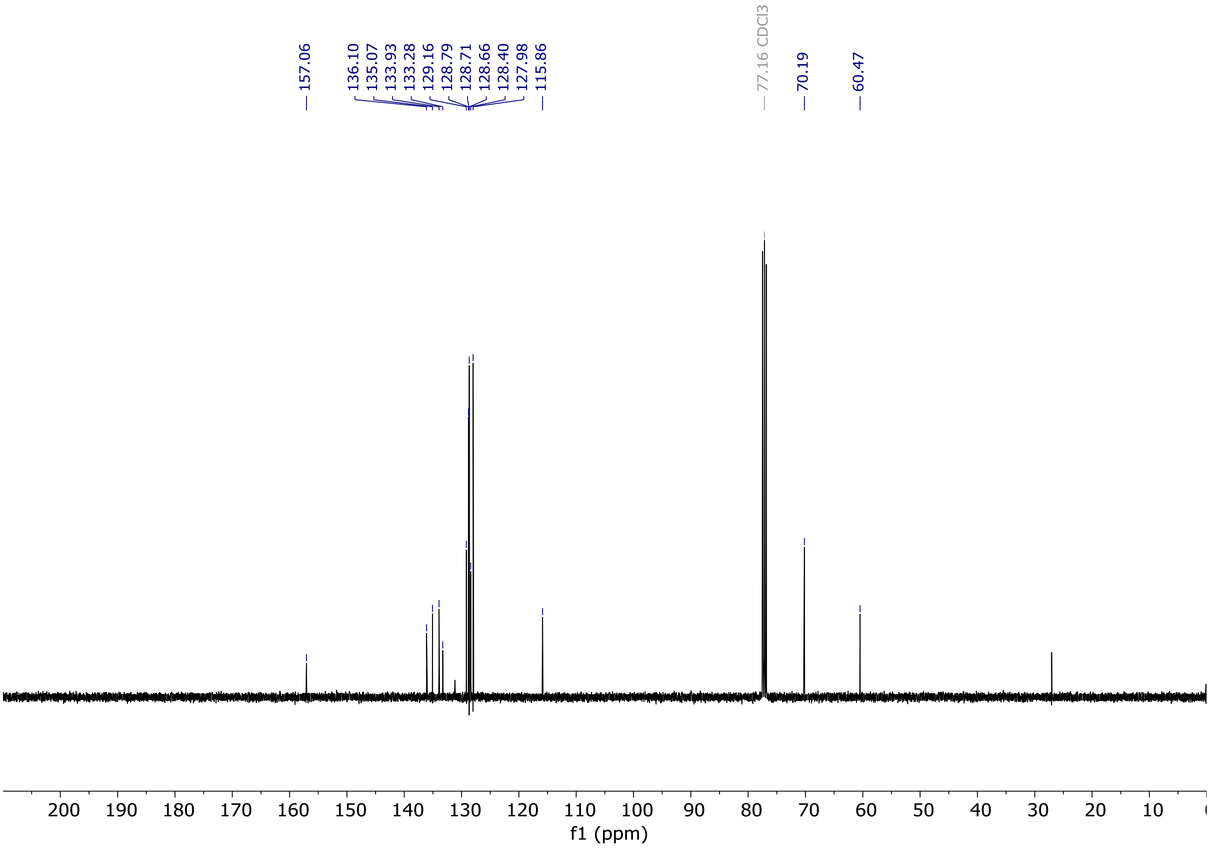

^{1}H NMR (400 MHz, $CDCl_3$)

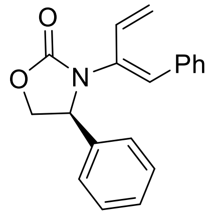

7b

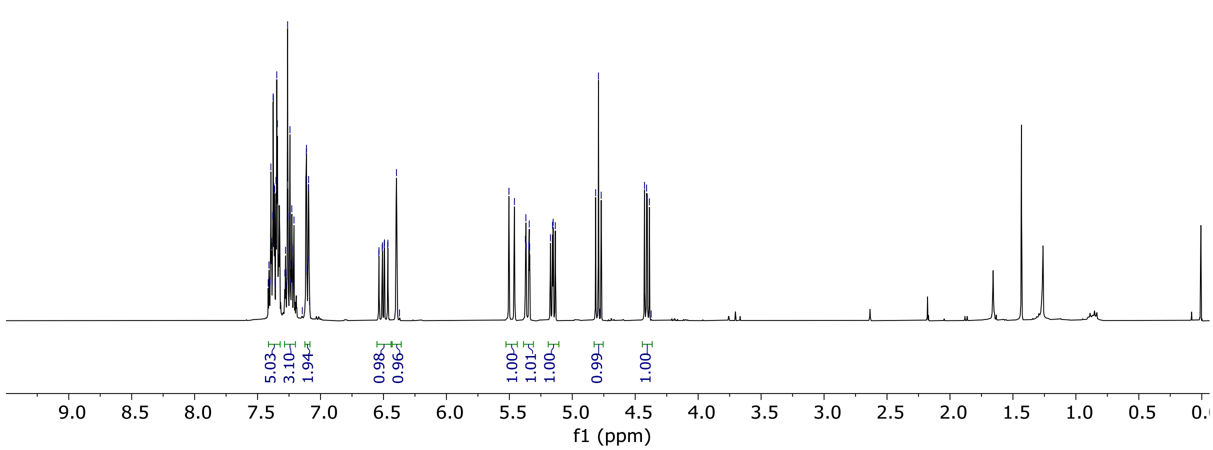

^{13}C {^{1}H} NMR (101 MHz, $CDCl_3$)

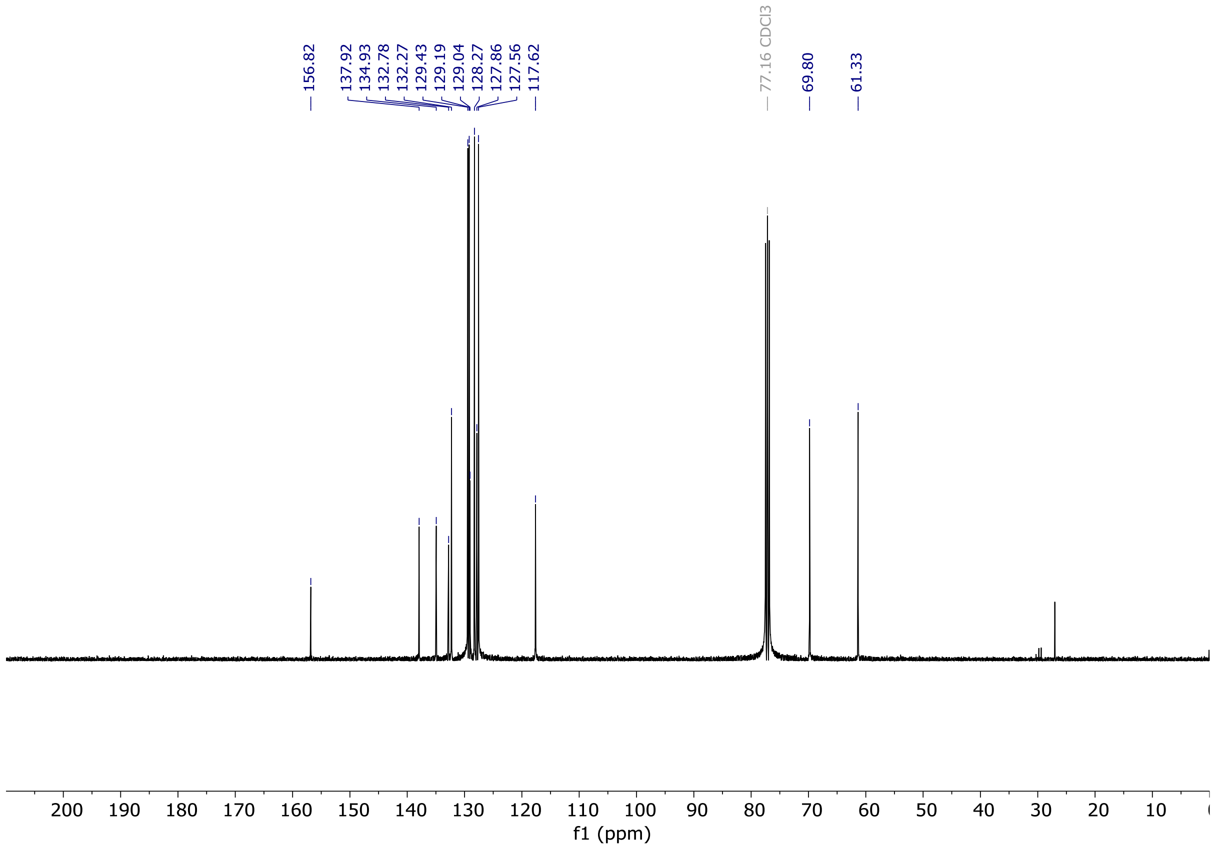

^{1}H NMR (400 MHz, CDCl$_3$)

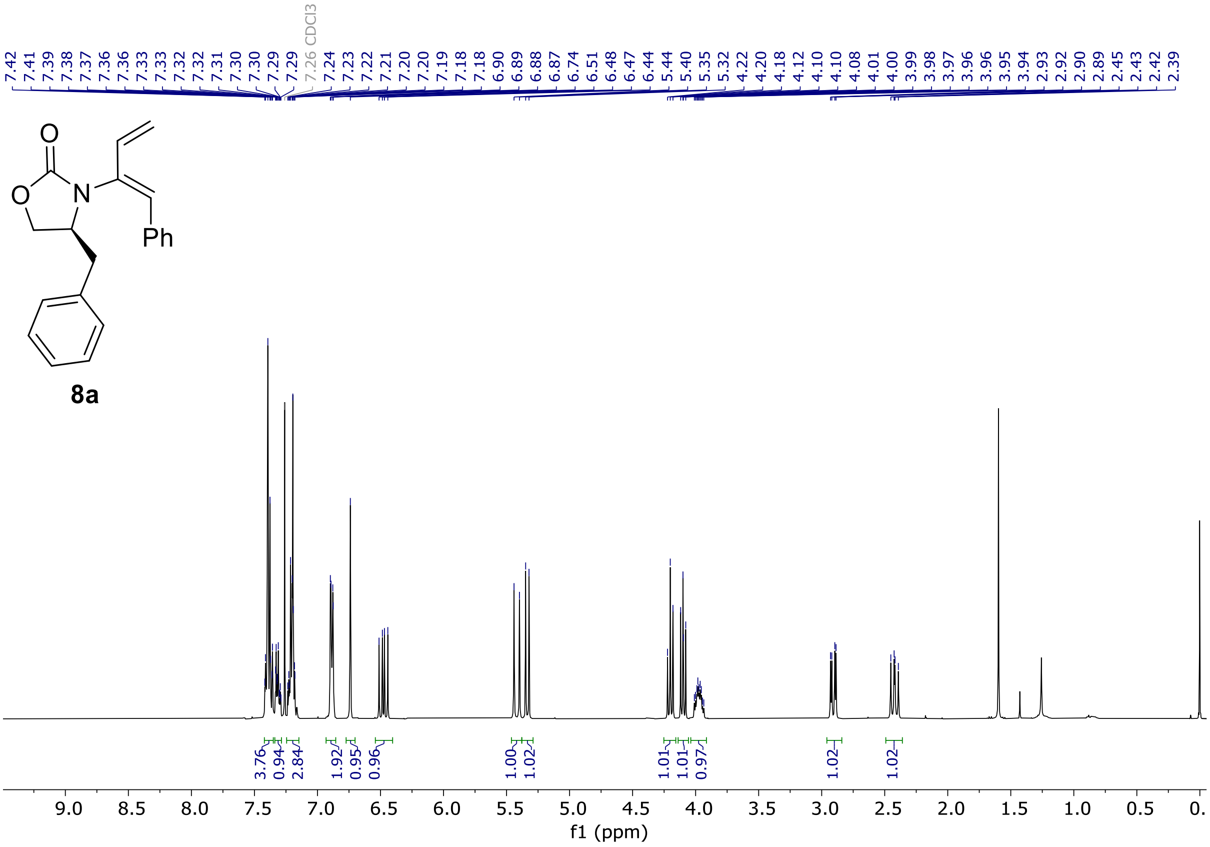

^{13}C {^{1}H} NMR (101 MHz, CDCl$_3$)

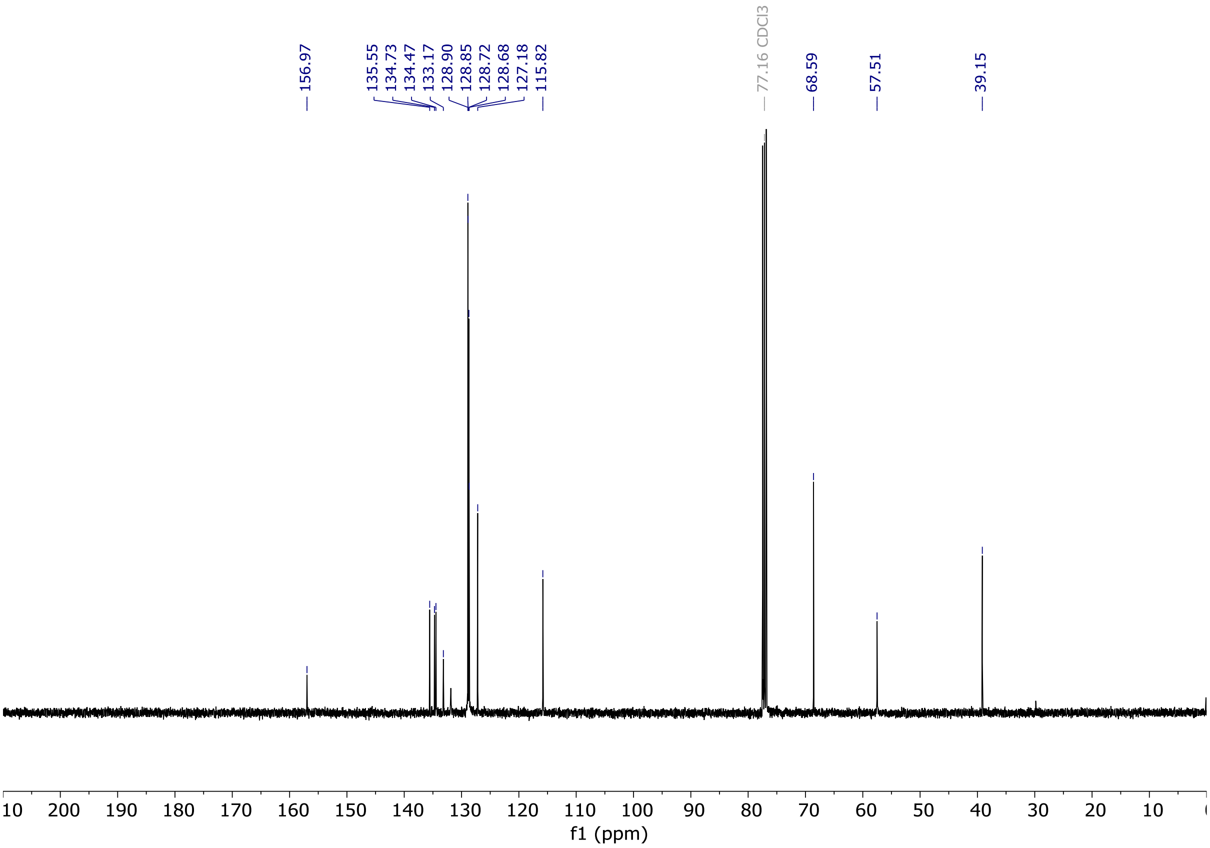

^{1}H NMR (500 MHz, $CDCl_3$)

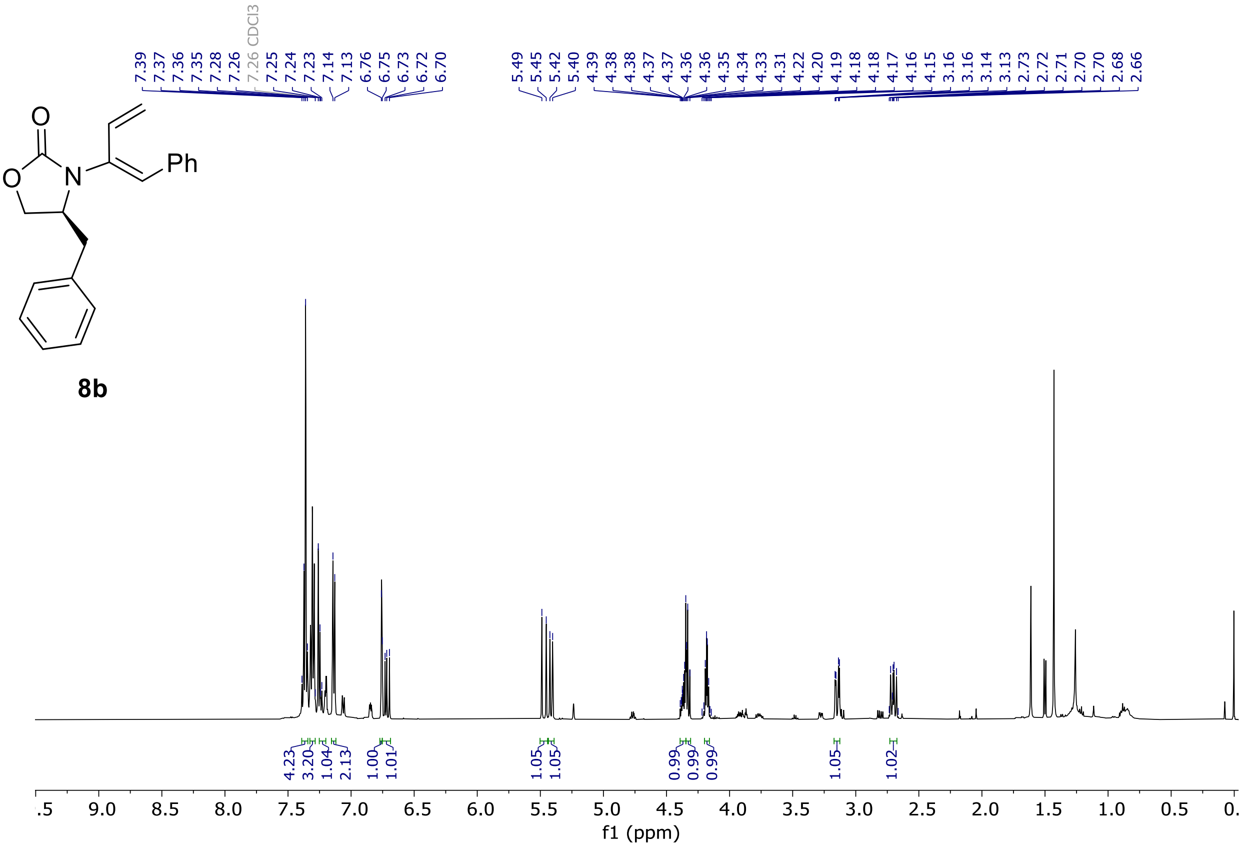

8b

^{13}C {^{1}H} NMR (126 MHz, $CDCl_3$)

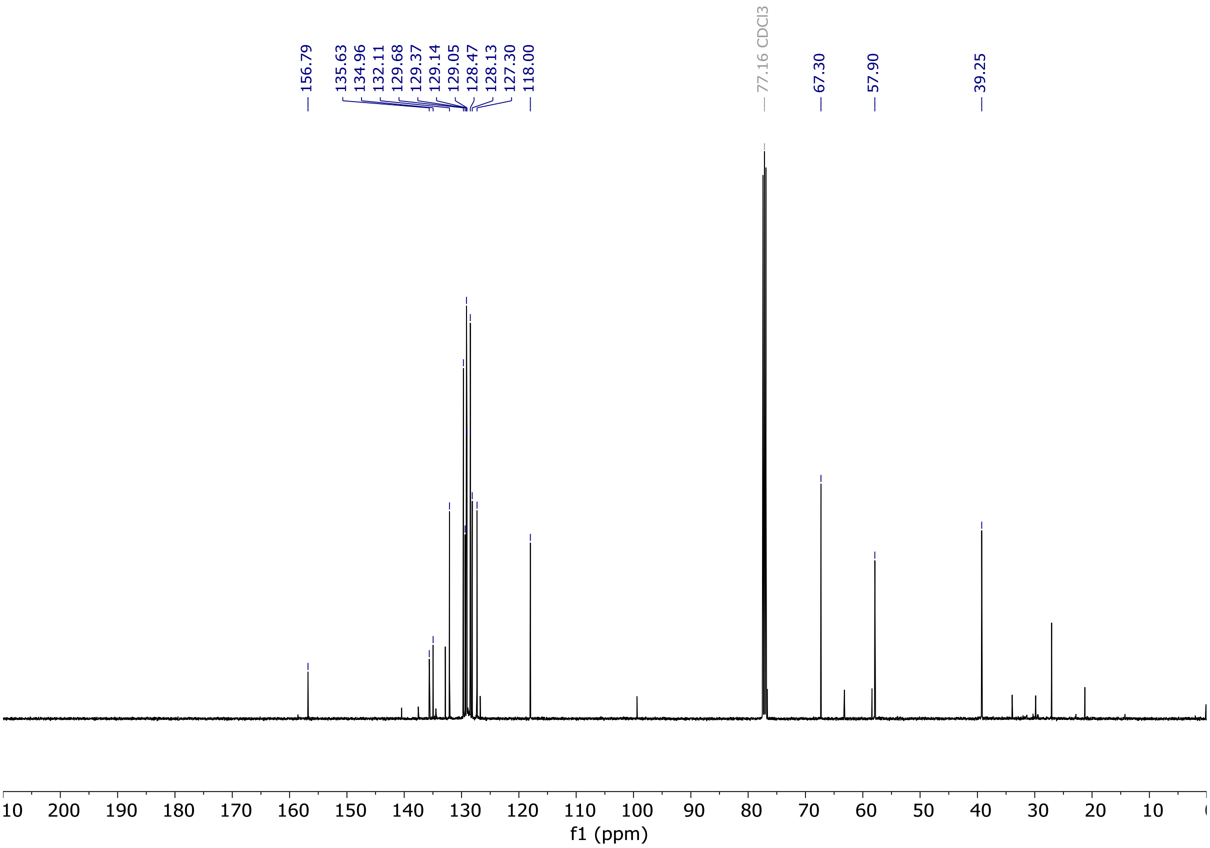

^{1}H NMR (400 MHz, $CDCl_3$)

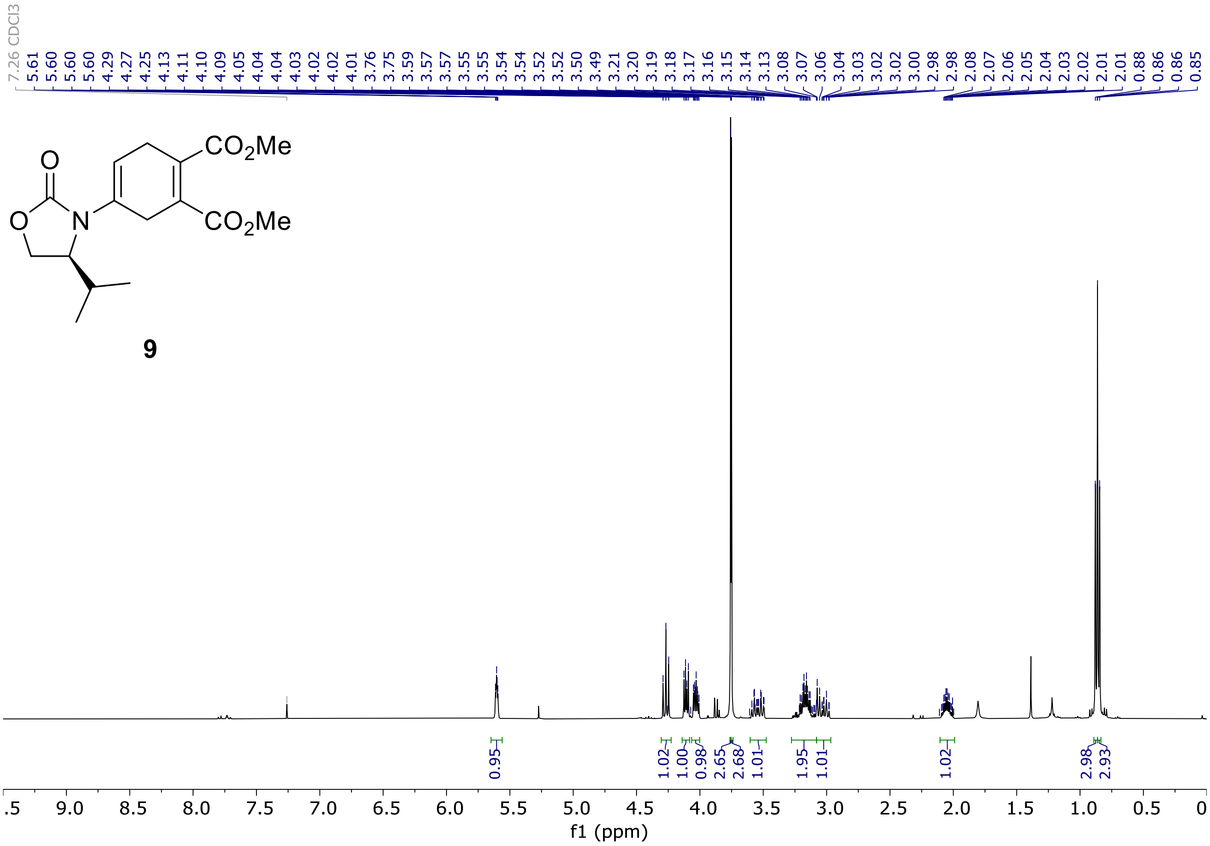

^{13}C {^{1}H} NMR (101 MHz, $CDCl_3$)

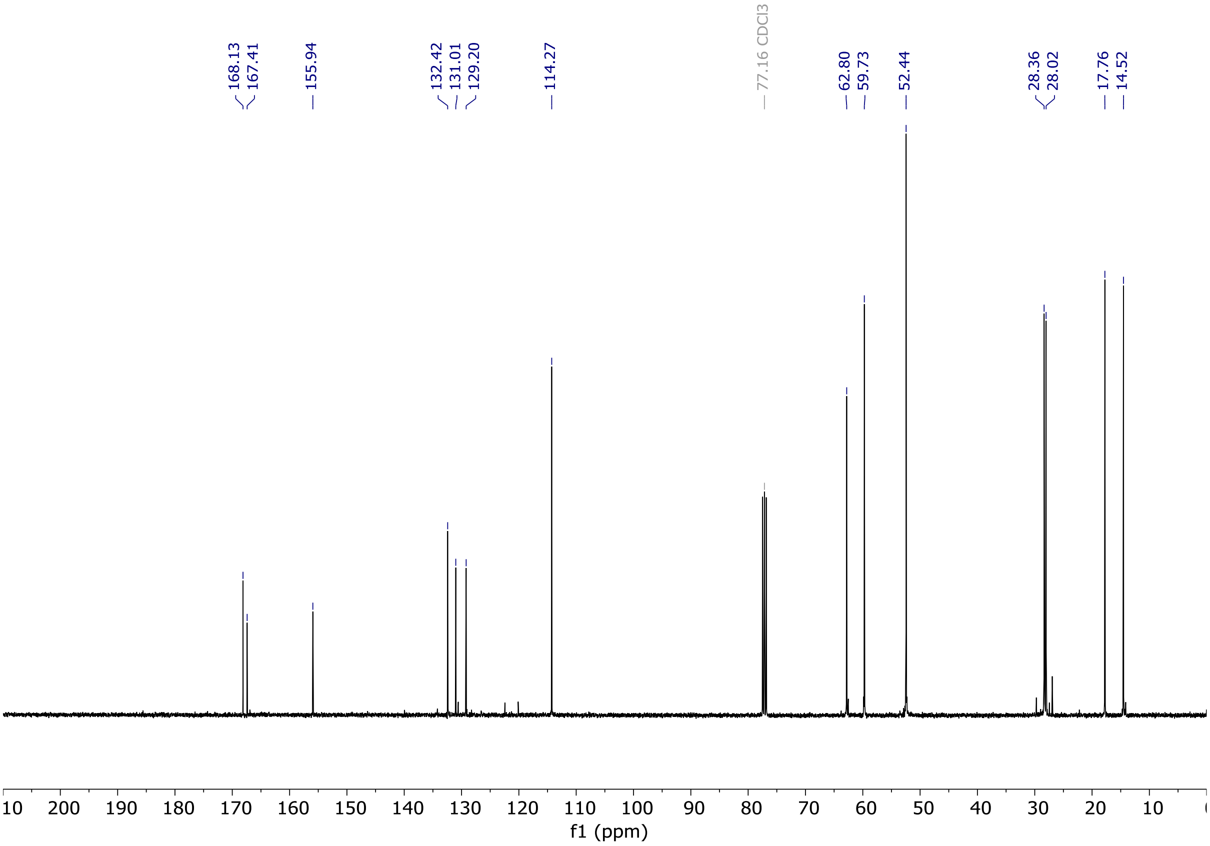

^{1}H NMR (400 MHz, $CDCl_3$)

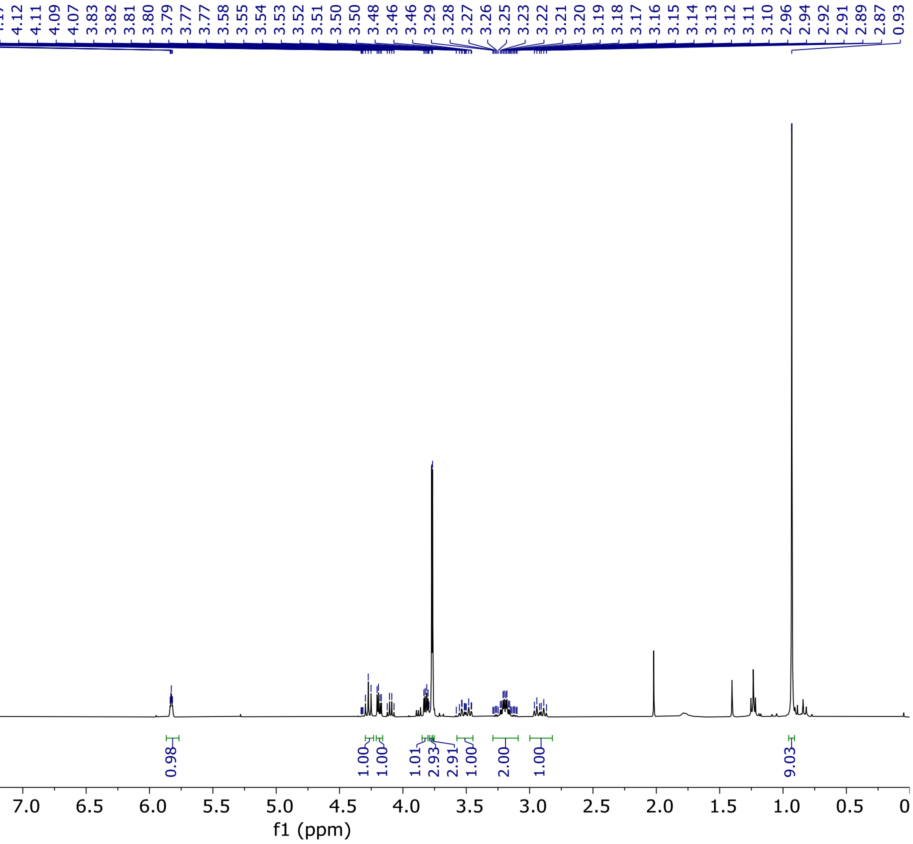

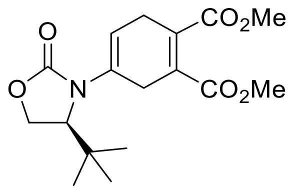

10

^{13}C {^{1}H} NMR (101 MHz, $CDCl_3$)

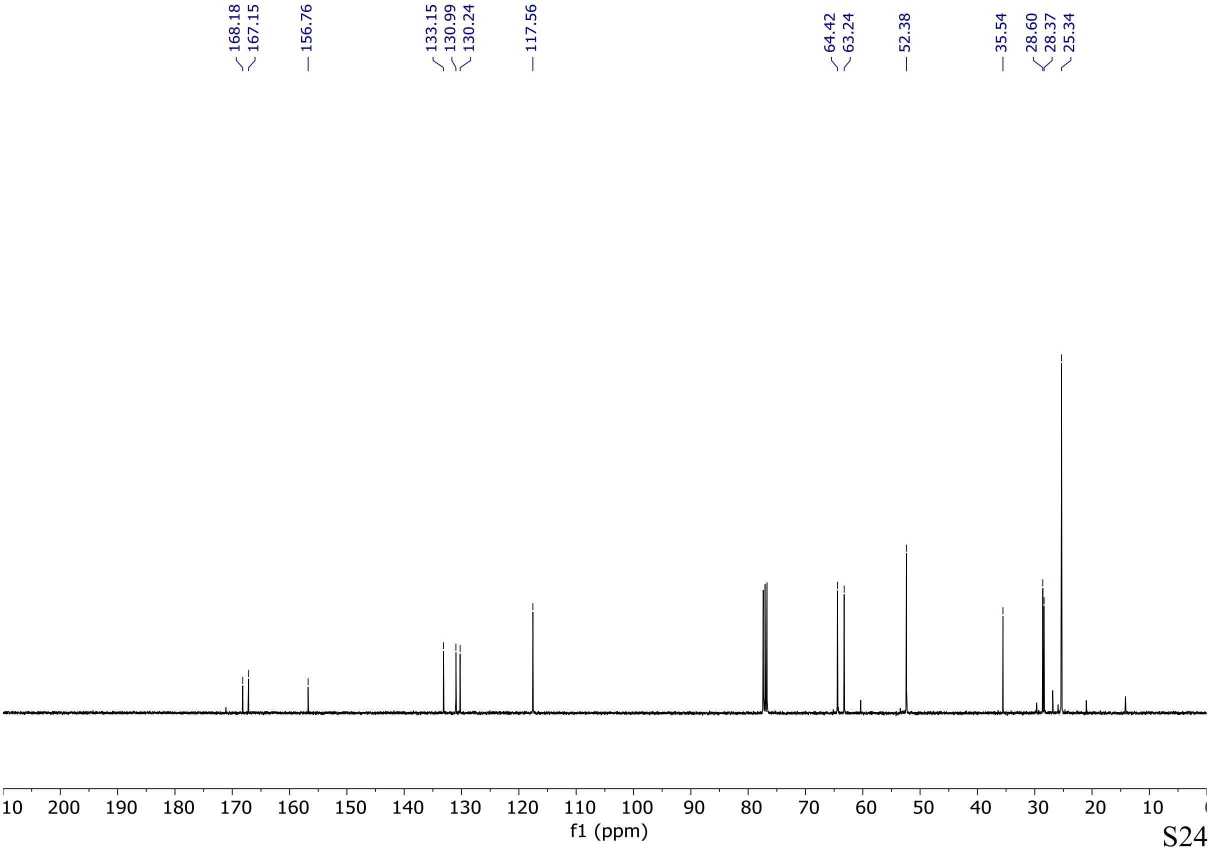

^{1}H NMR (400 MHz, $CDCl_3$)

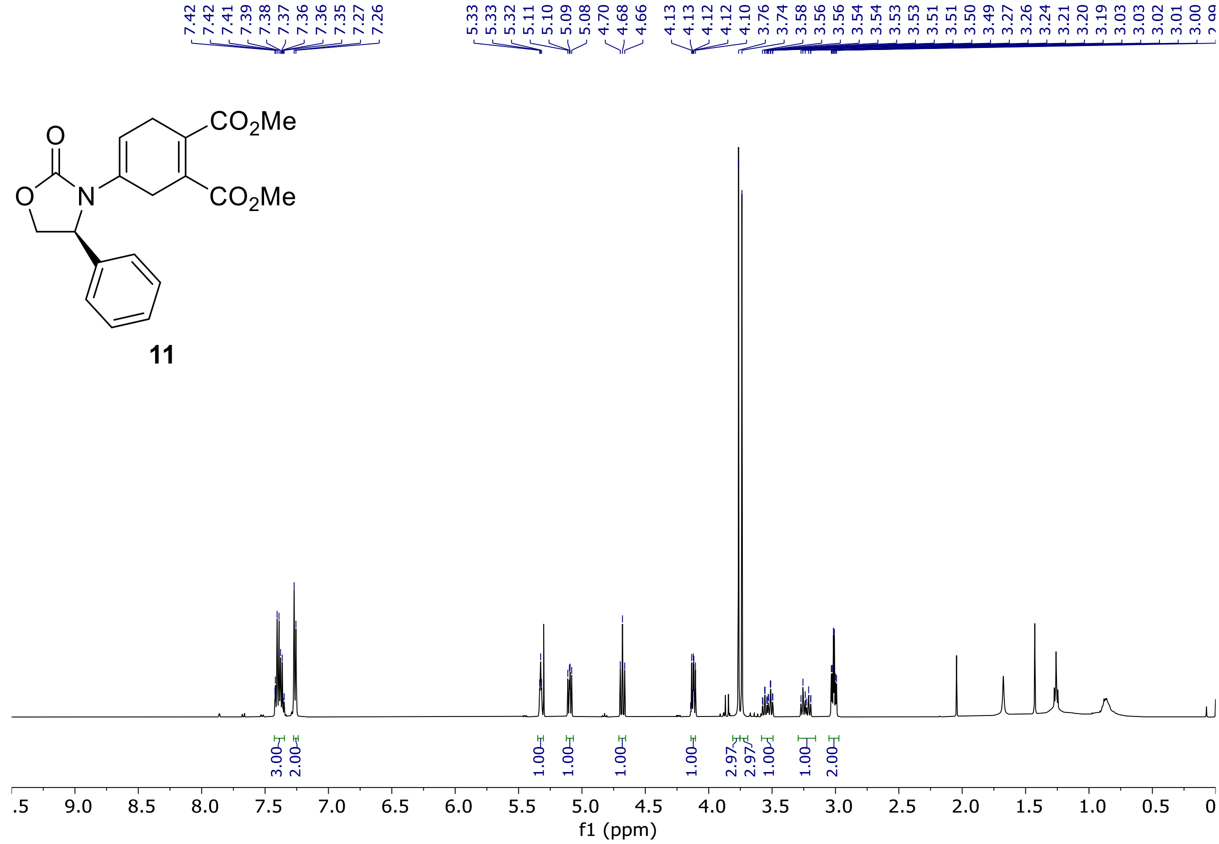

^{13}C {^{1}H} NMR (126 MHz, $CDCl_3$)

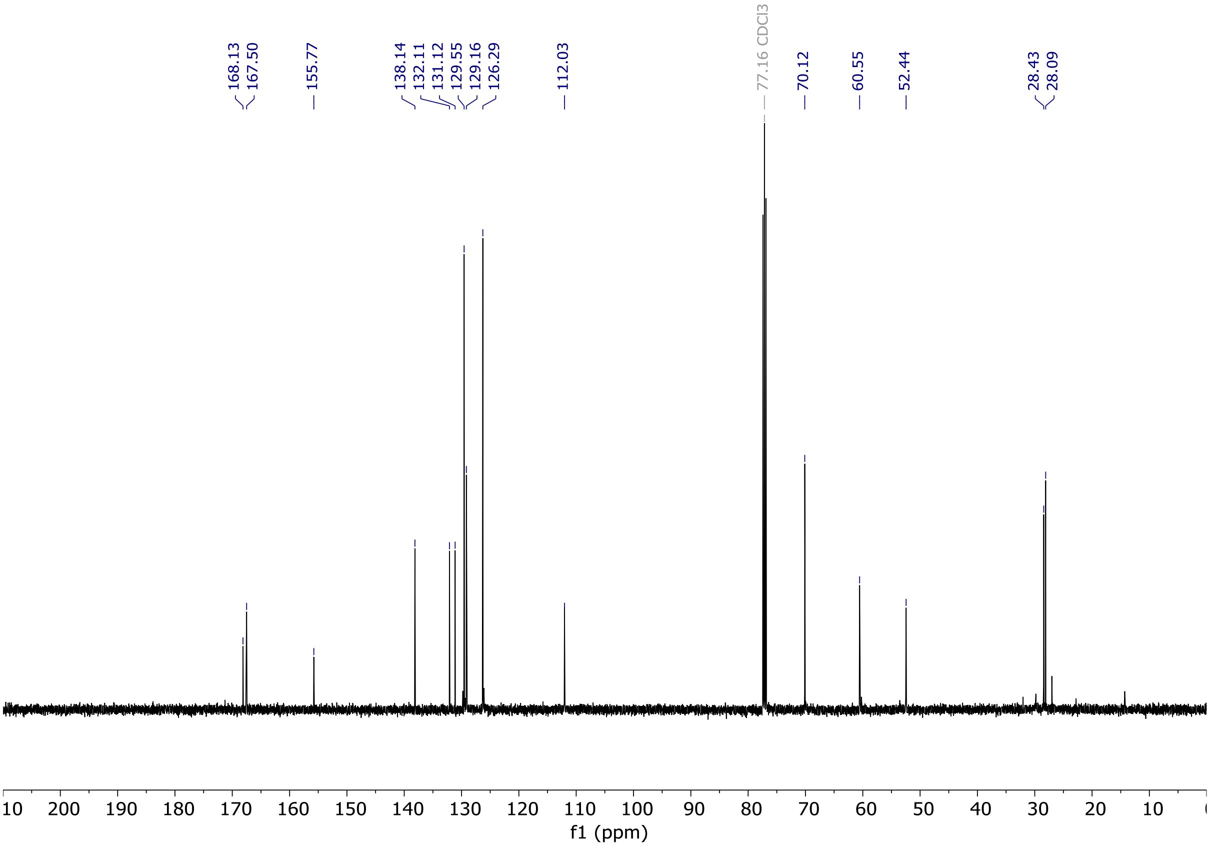

^{1}H NMR (400 MHz, $CDCl_3$)

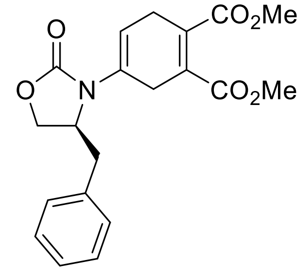

12

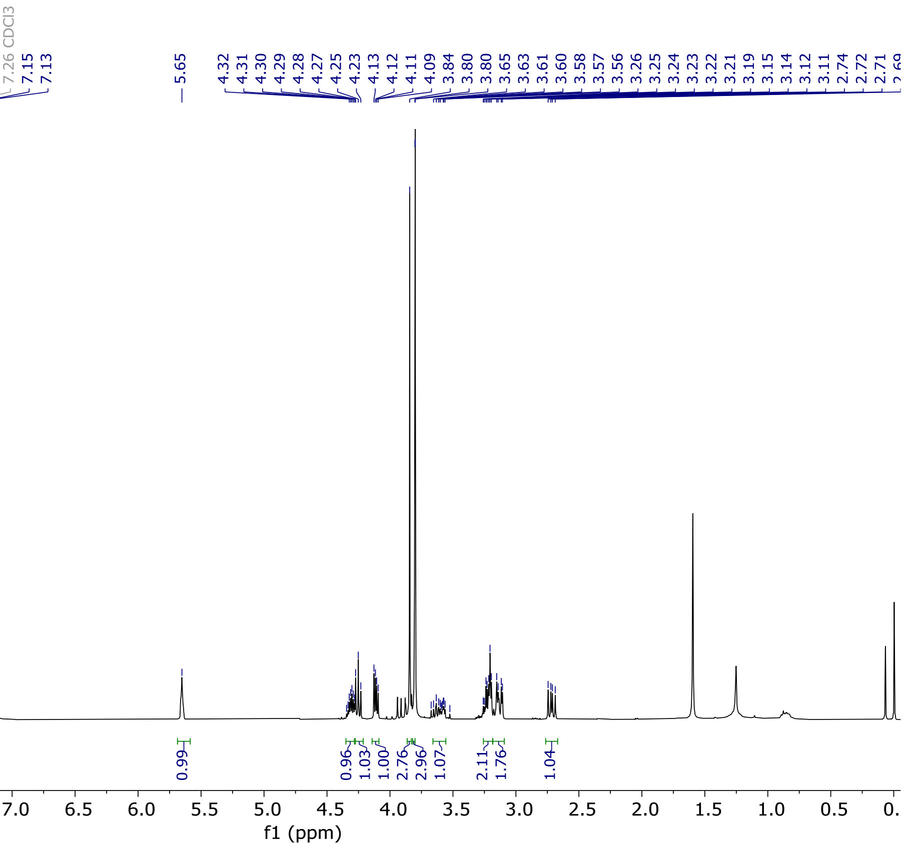

^{13}C {^{1}H} NMR (126 MHz, $CDCl_3$)

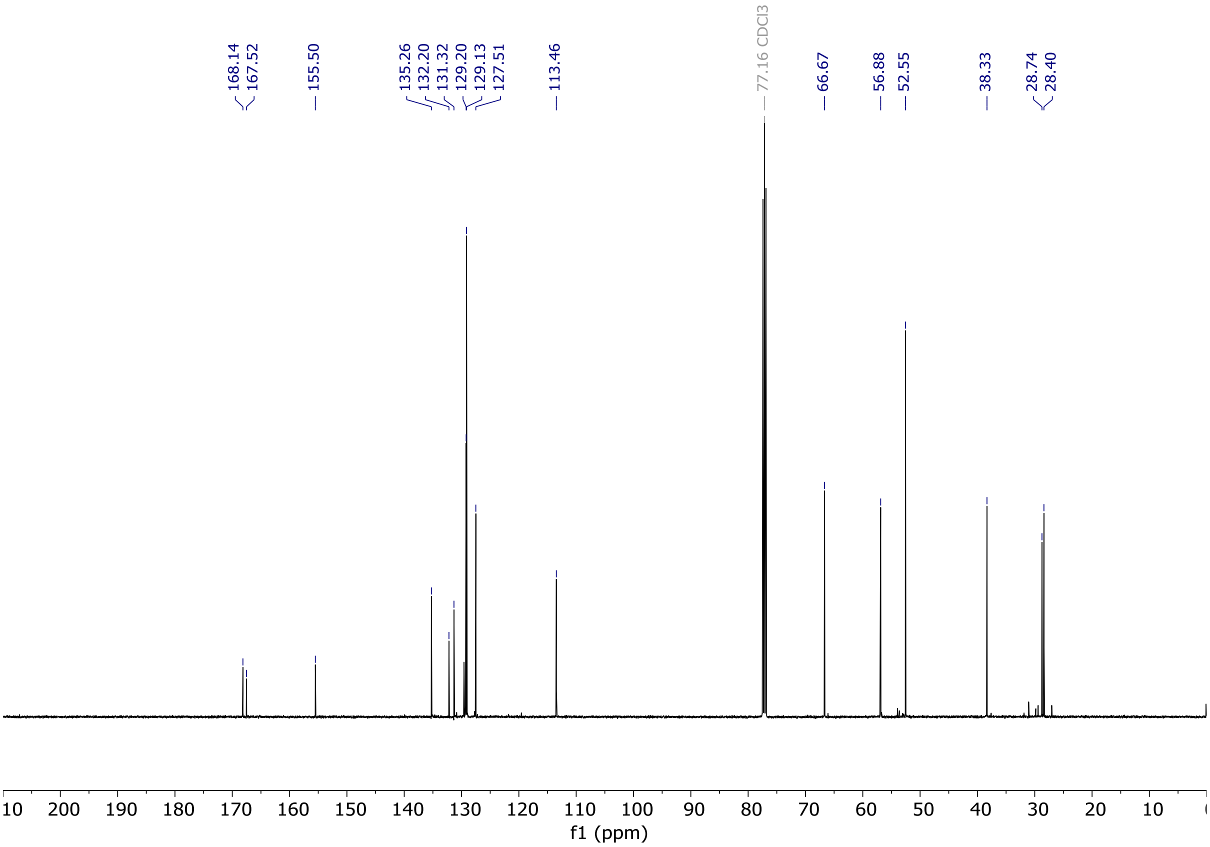

^{1}H NMR (400 MHz, $CDCl_3$)

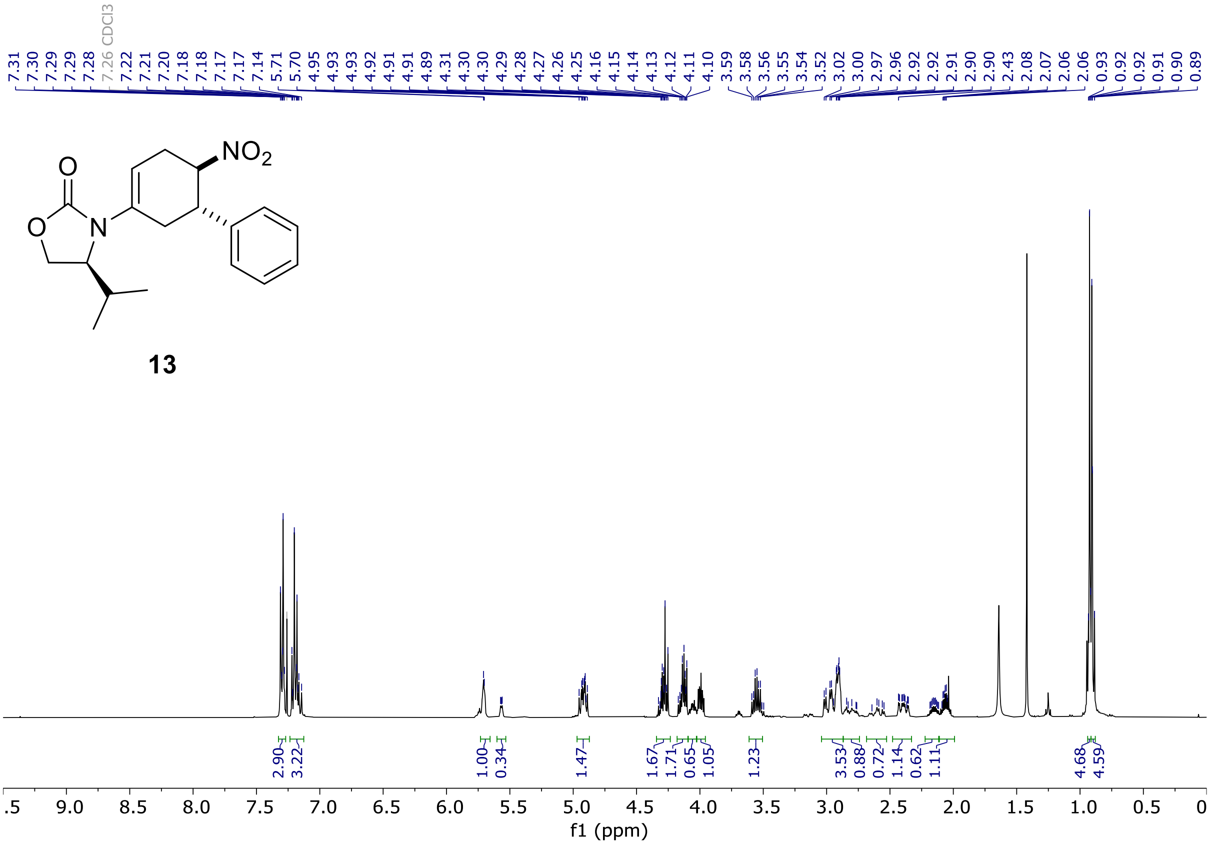

13

^{13}C {^{1}H} NMR (126 MHz, $CDCl_3$)

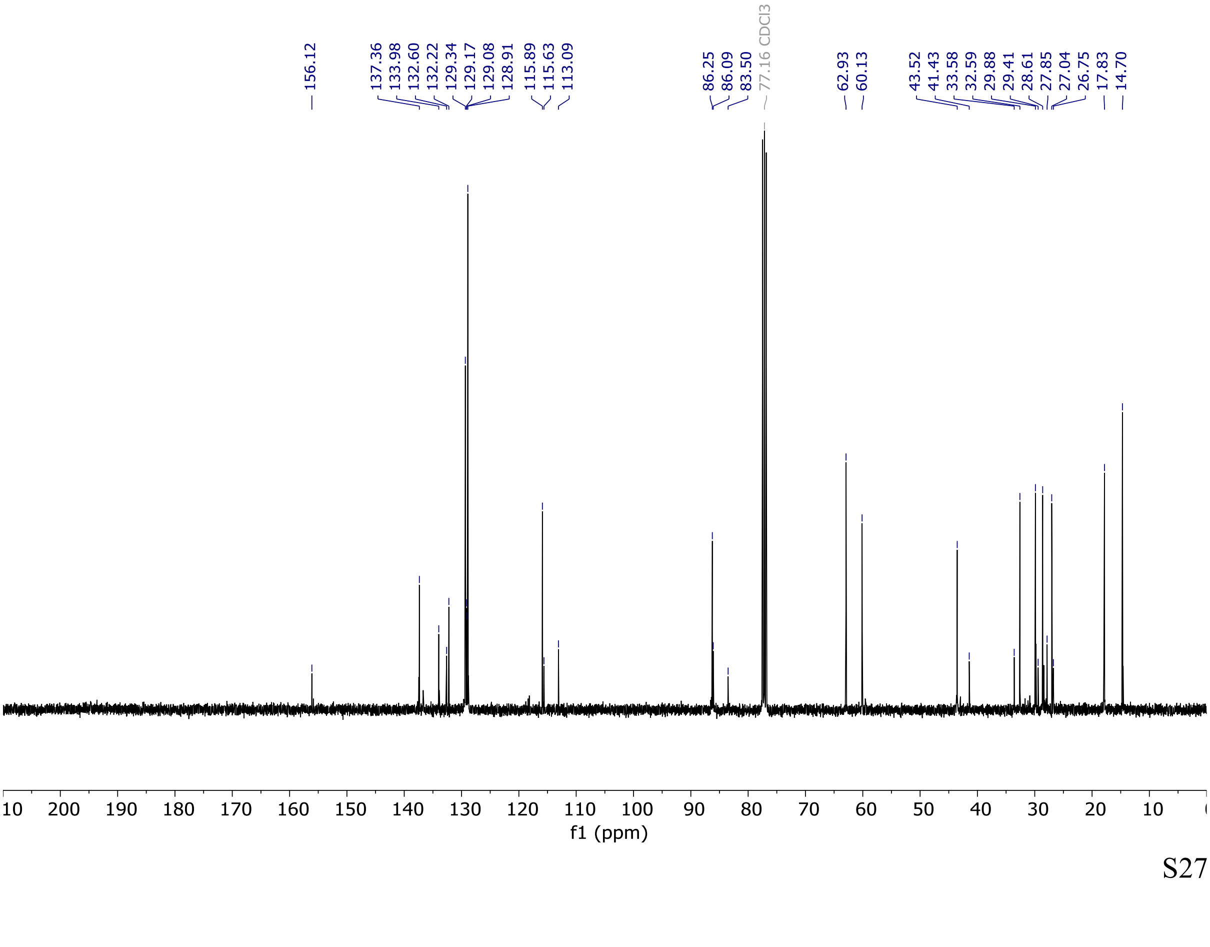

^{1}H NMR (400 MHz, $CDCl_3$)

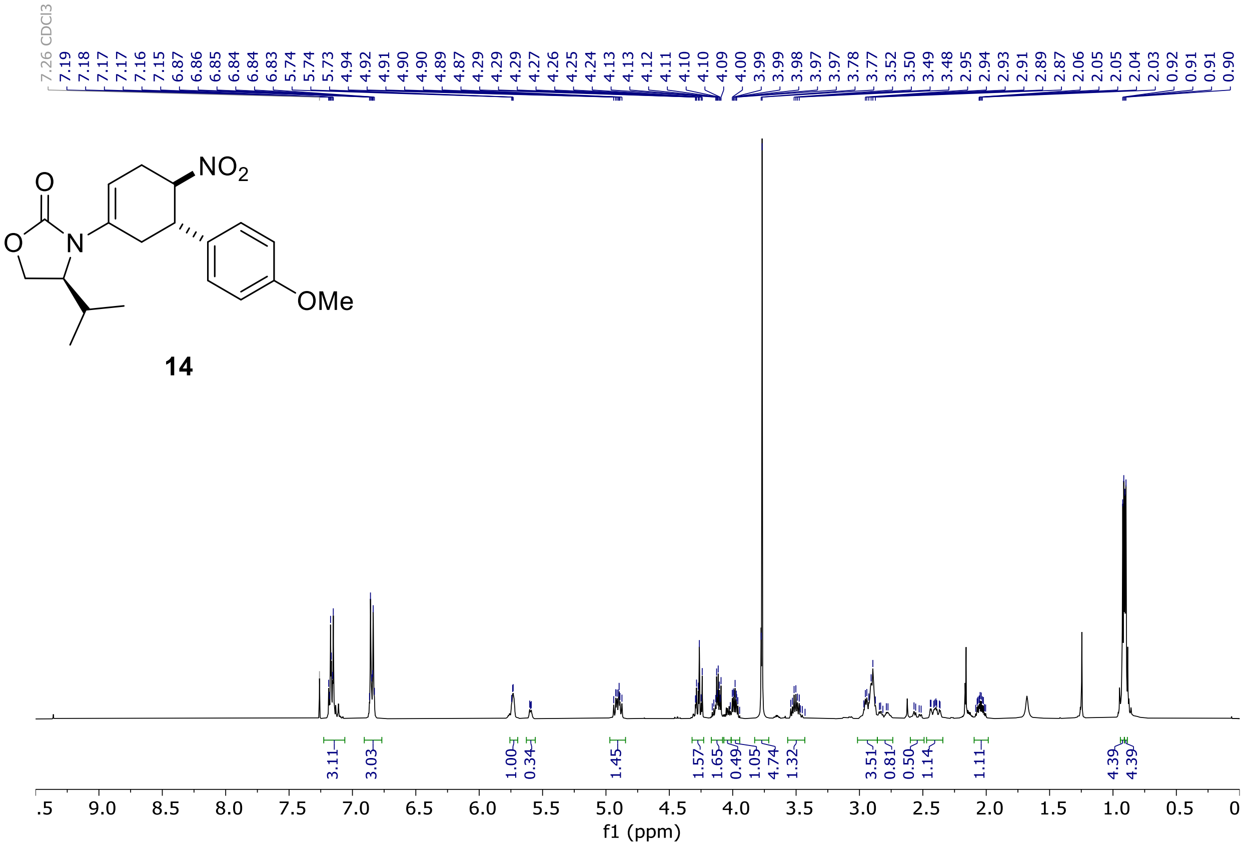

^{13}C {^{1}H} NMR (101 MHz, $CDCl_3$)

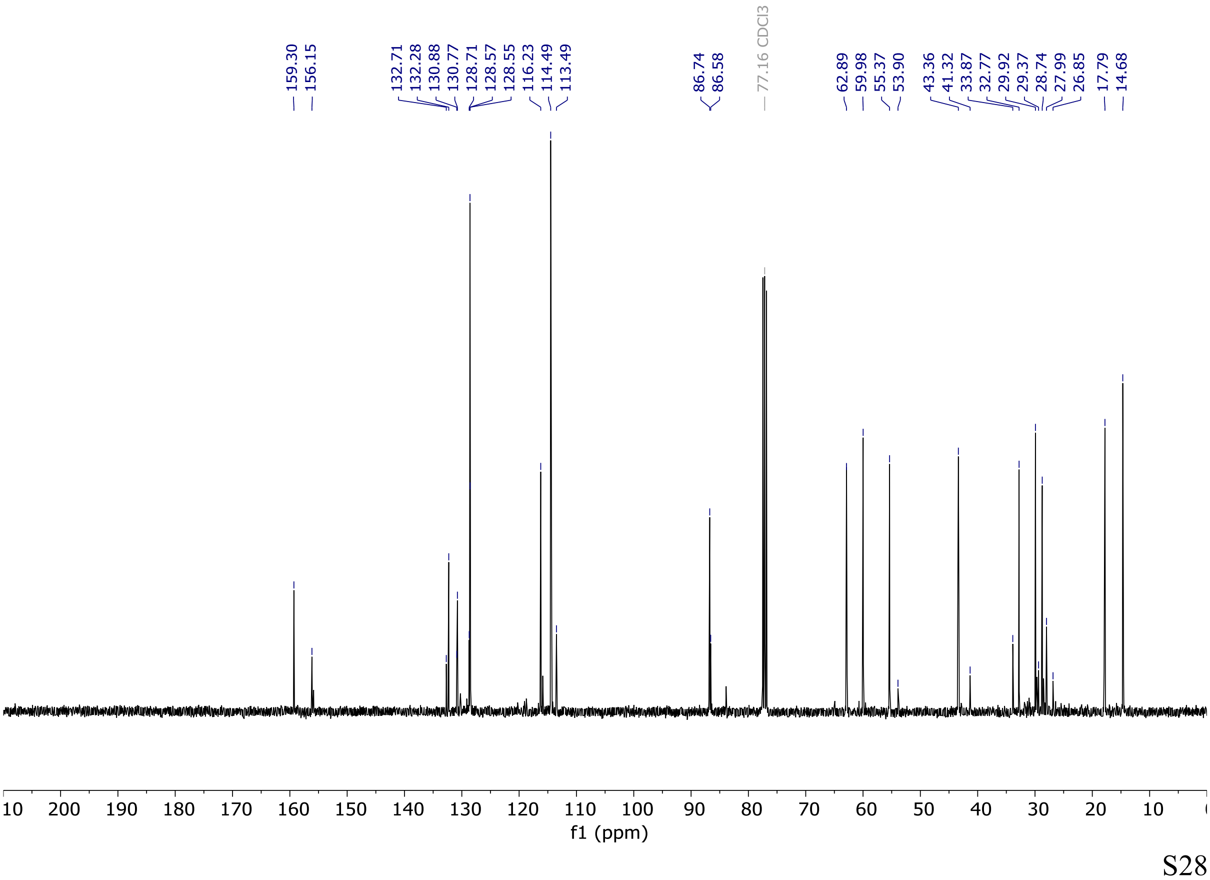

^{1}H NMR (400 MHz, $CDCl_3$)

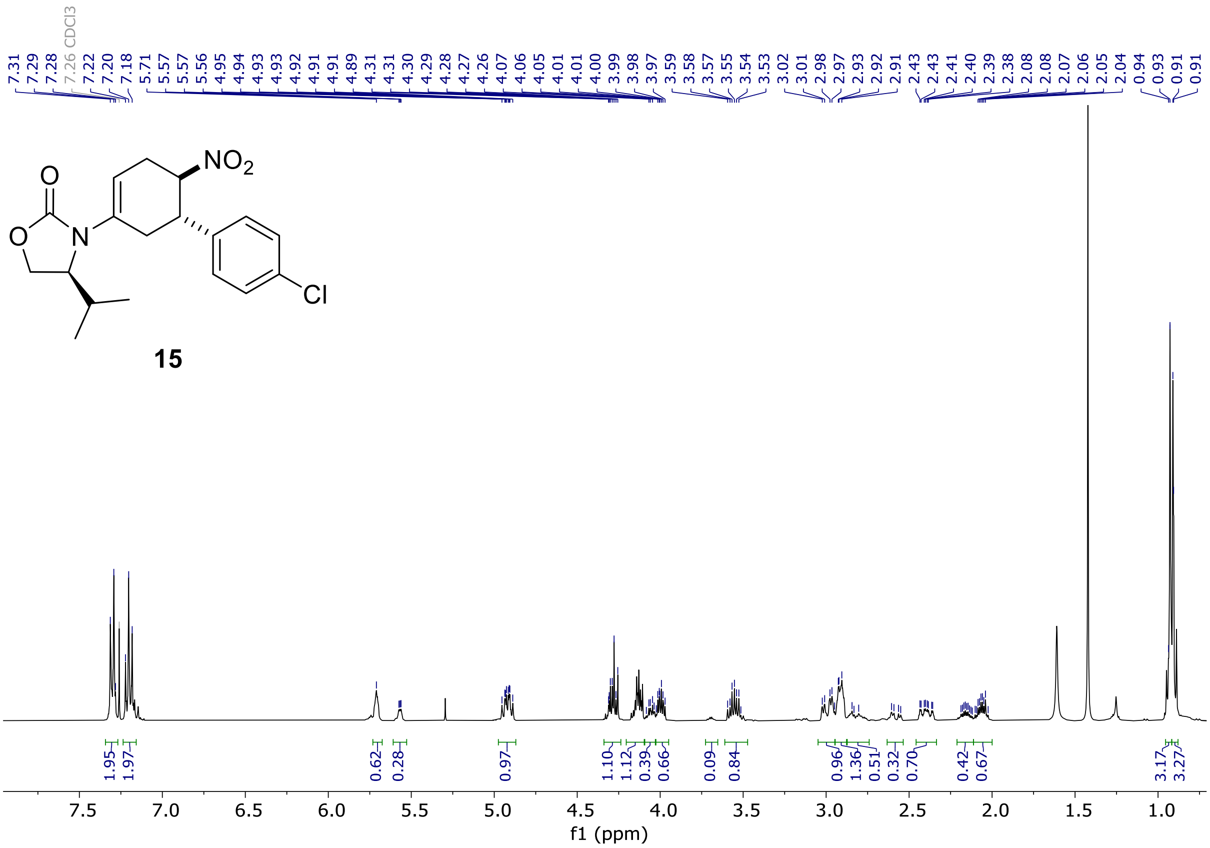

15

^{13}C {^{1}H} NMR (101 MHz, $CDCl_3$)

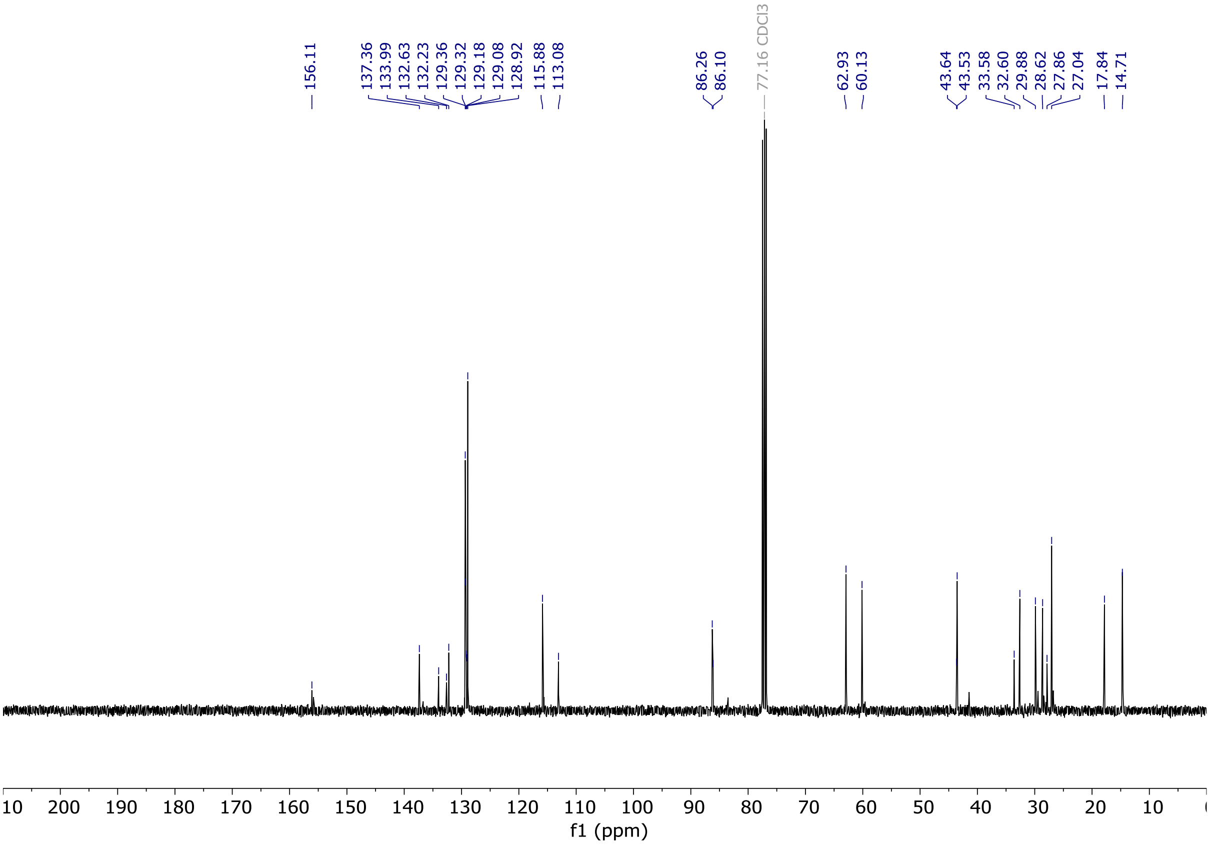

^{1}H NMR (500 MHz, $CDCl_3$)

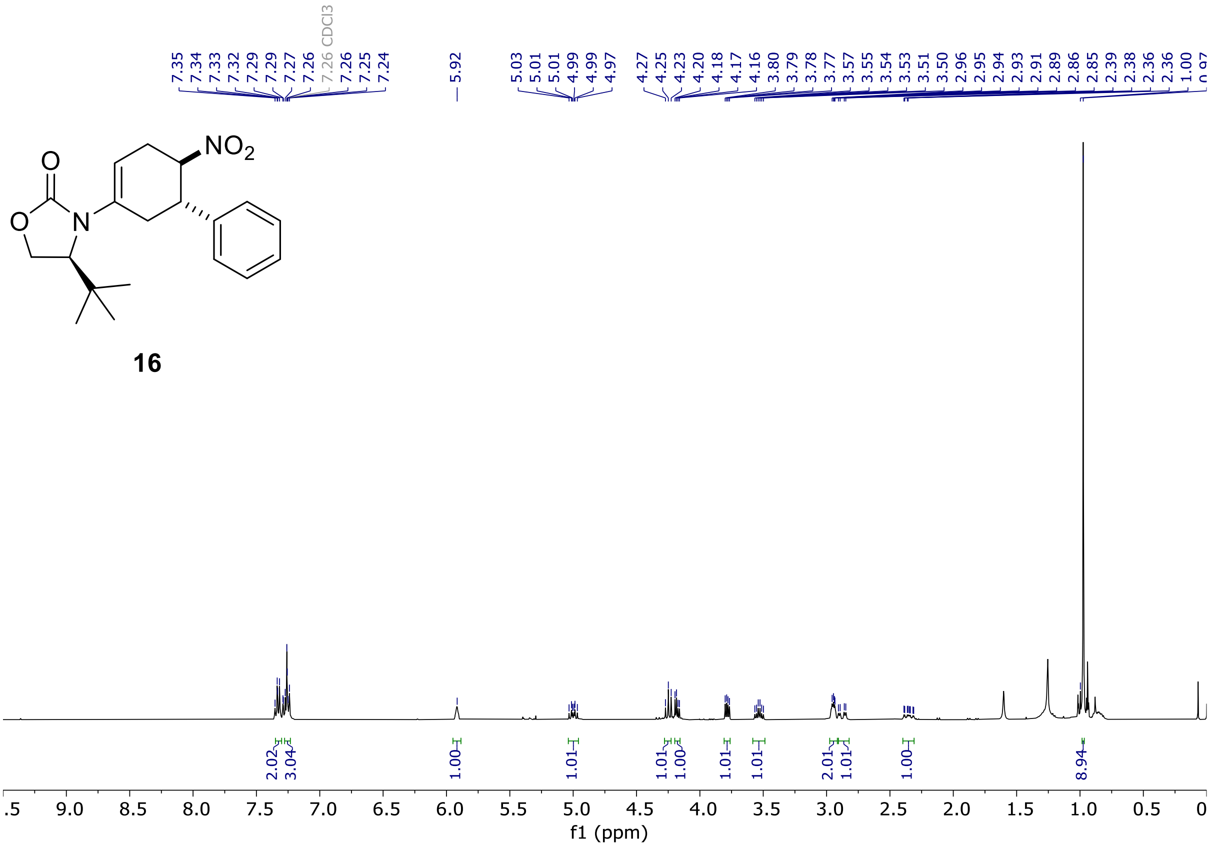

16

^{13}C {^{1}H} NMR (126 MHz, $CDCl_3$)

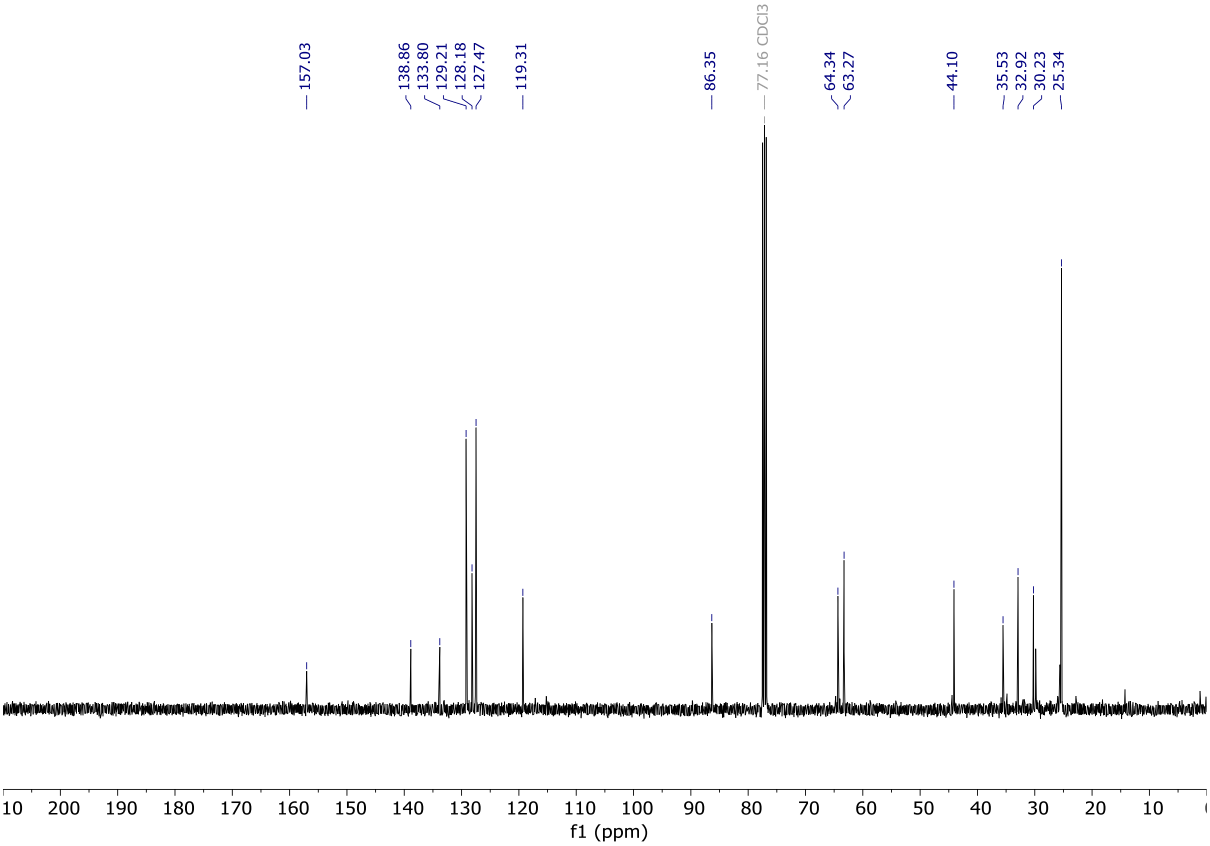

^{1}H NMR (400 MHz, $CDCl_3$)

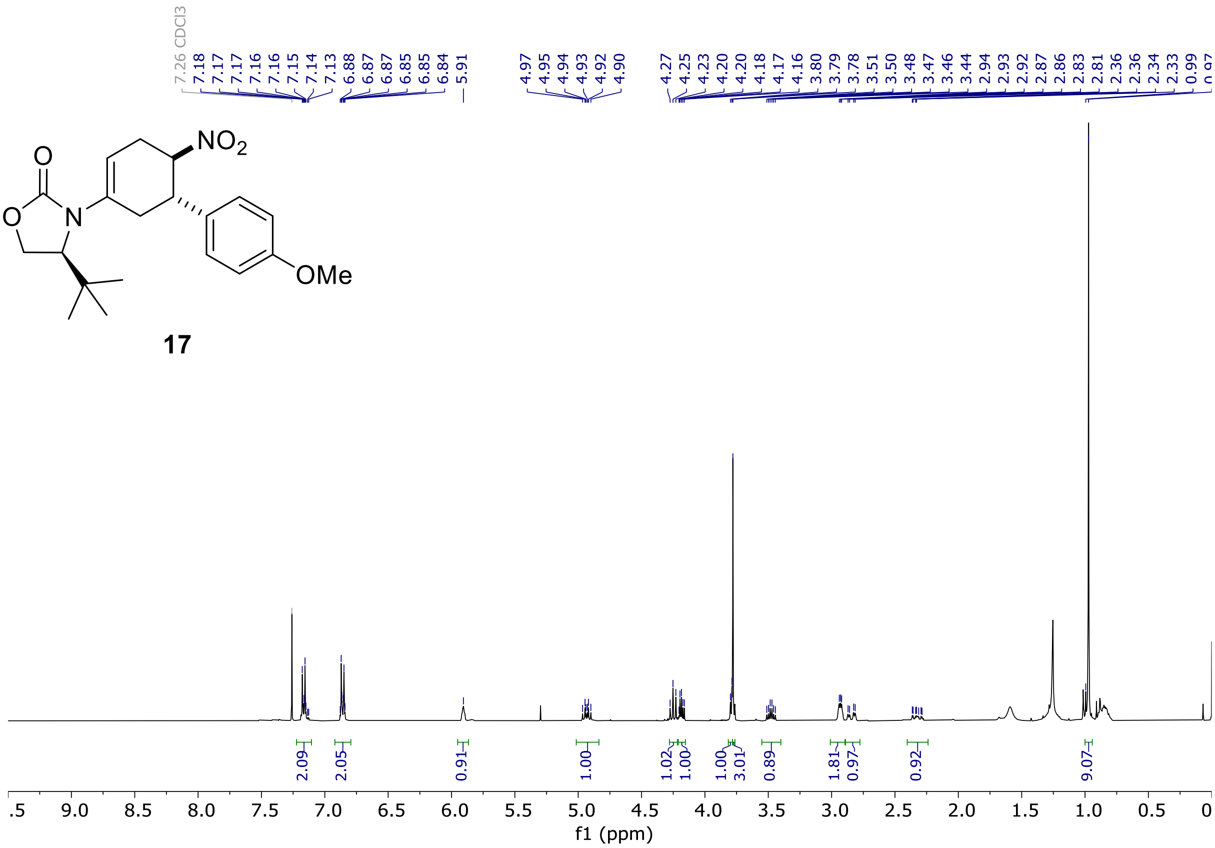

17

^{13}C {^{1}H} NMR (101 MHz, $CDCl_3$)

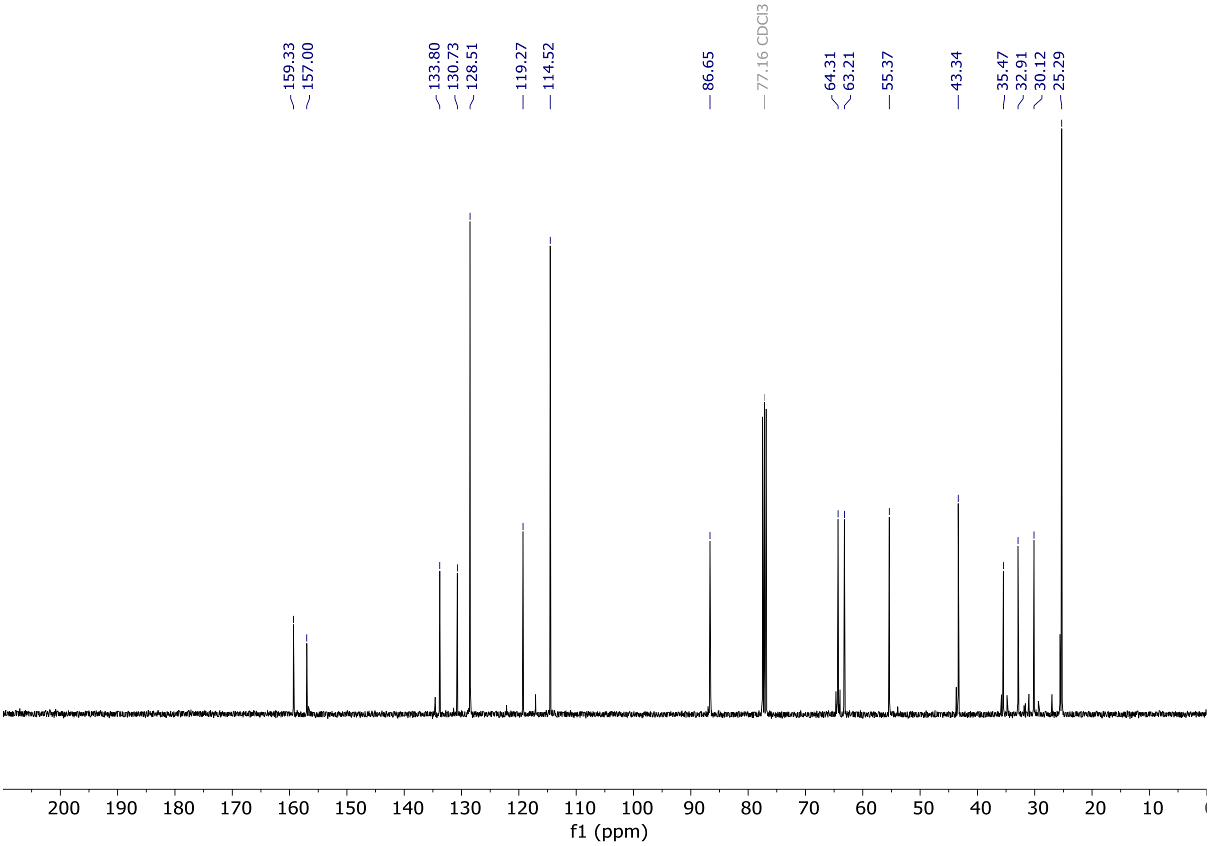

^{1}H NMR (400 MHz, CDCl$_3$)

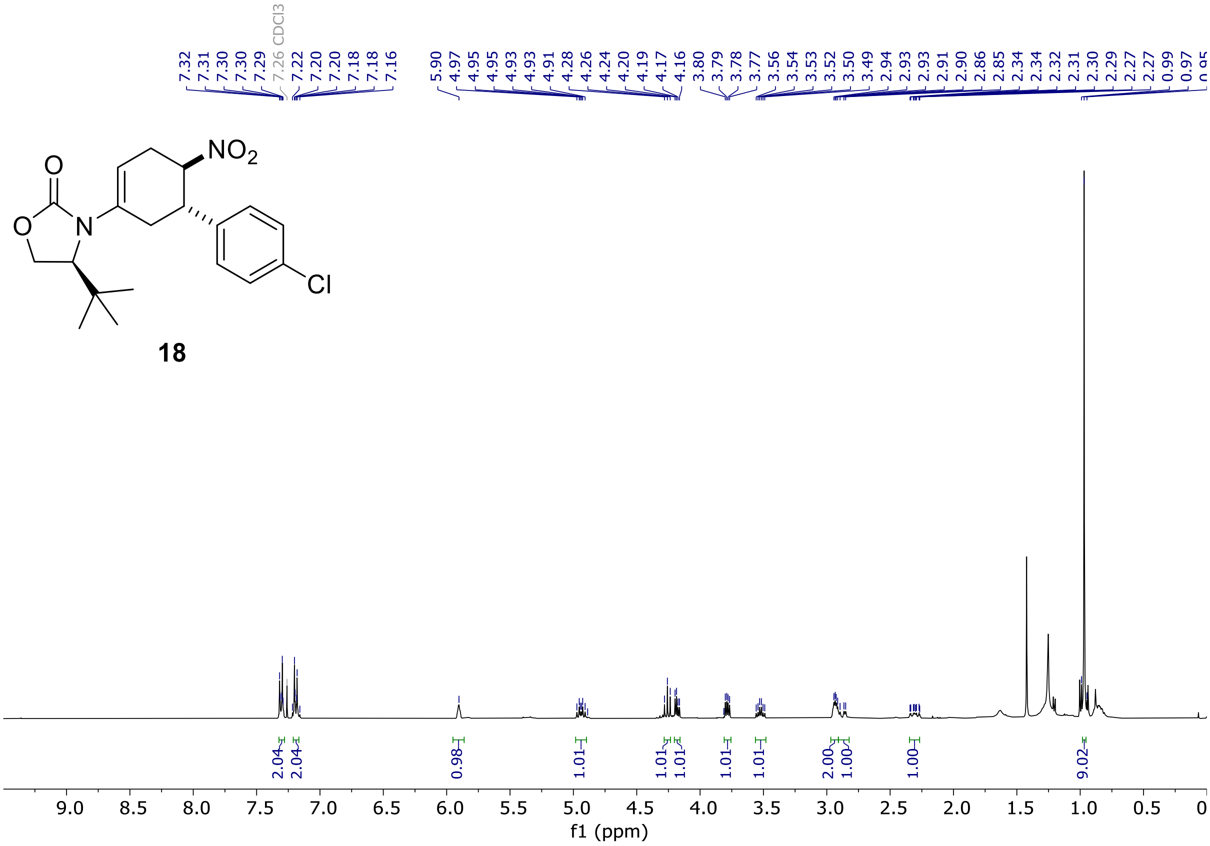

18

^{13}C {^{1}H} NMR (101 MHz, CDCl$_3$)

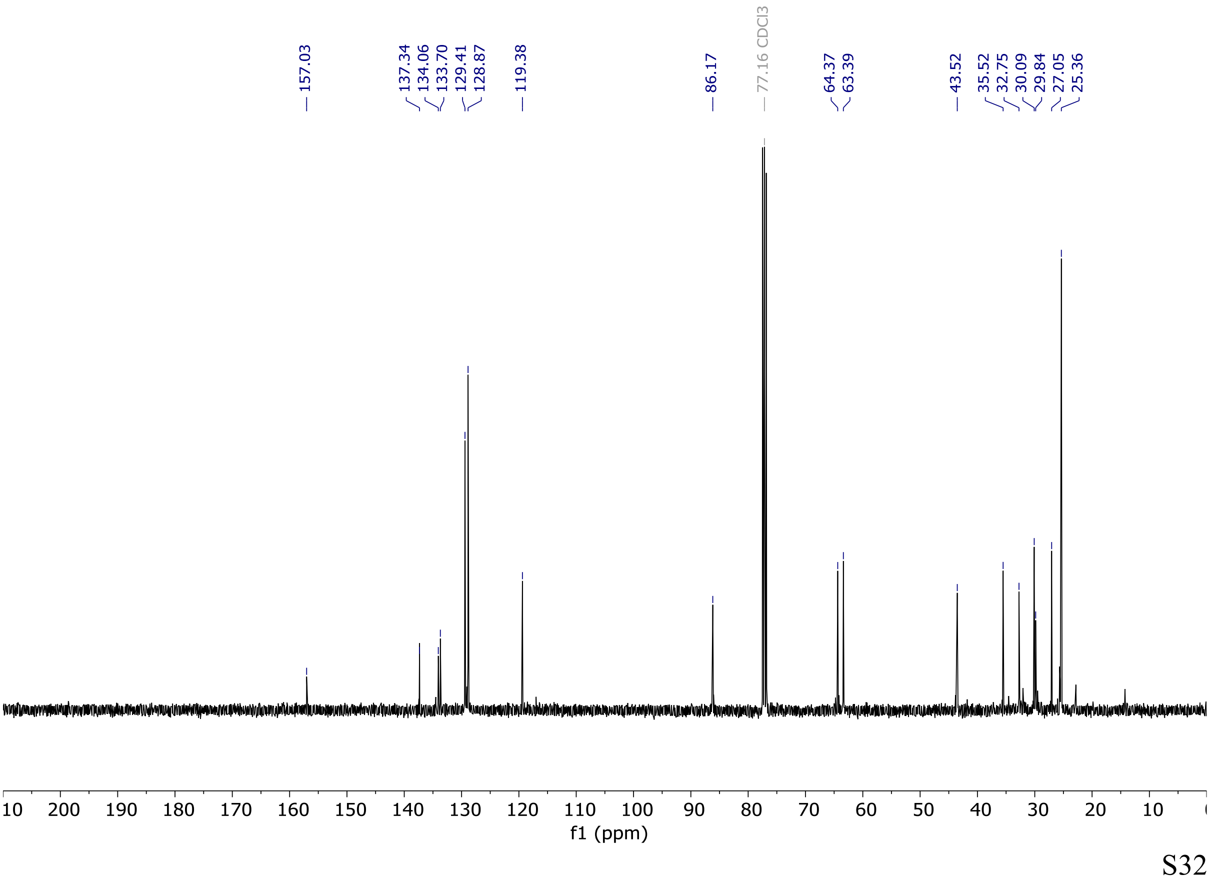

^{1}H NMR (400 MHz, $CDCl_3$)

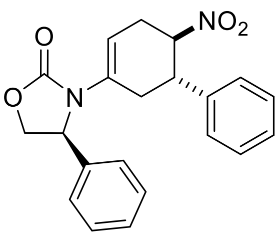

19

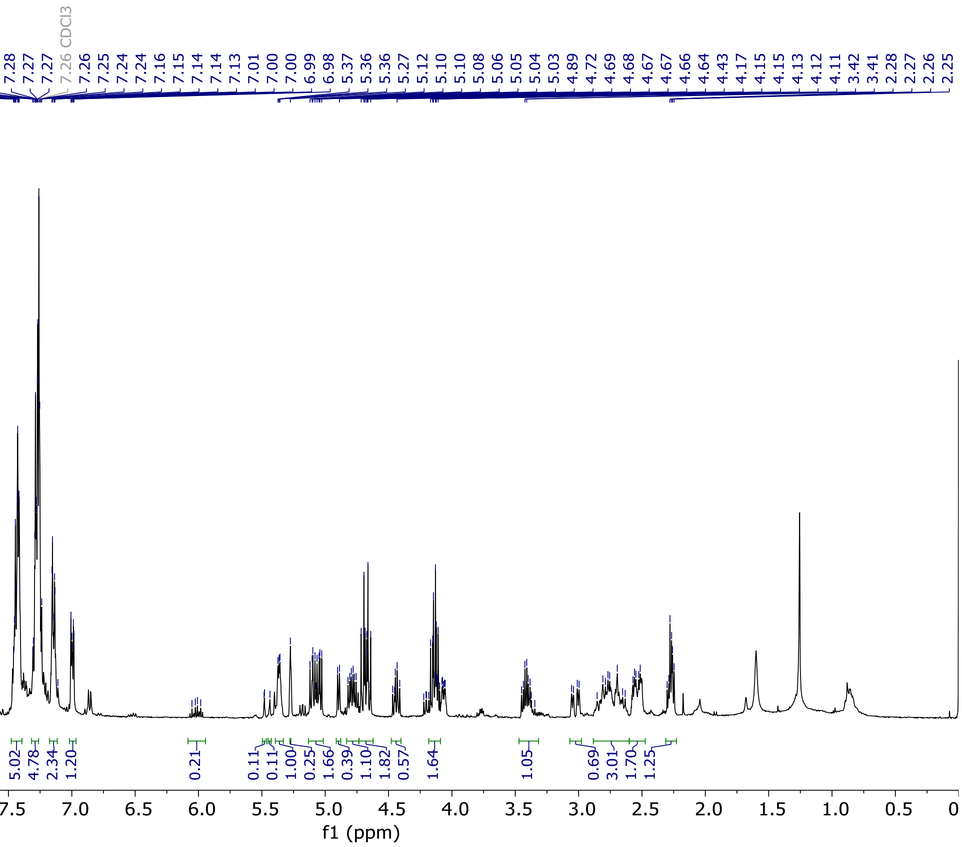

^{13}C {^{1}H} NMR (126 MHz, $CDCl_3$)

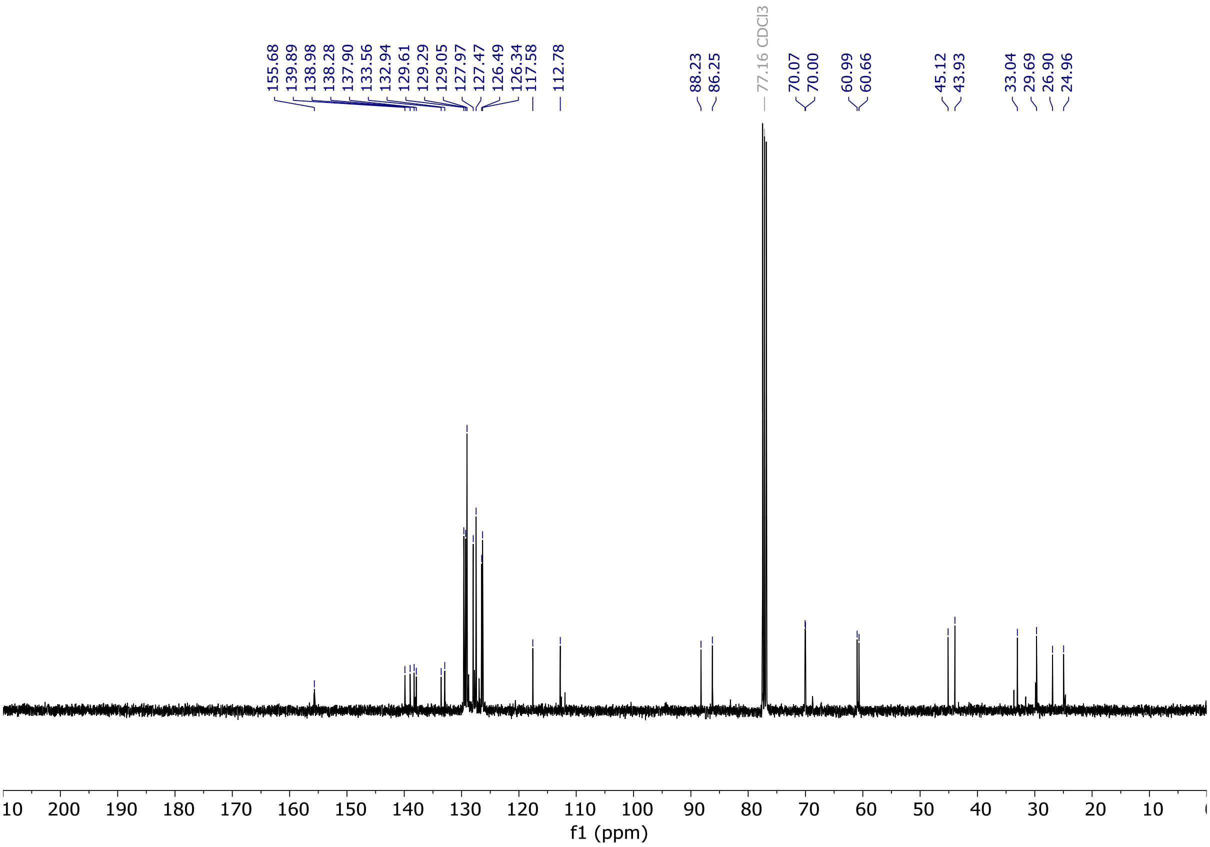

^{1}H NMR (400 MHz, $CDCl_3$)

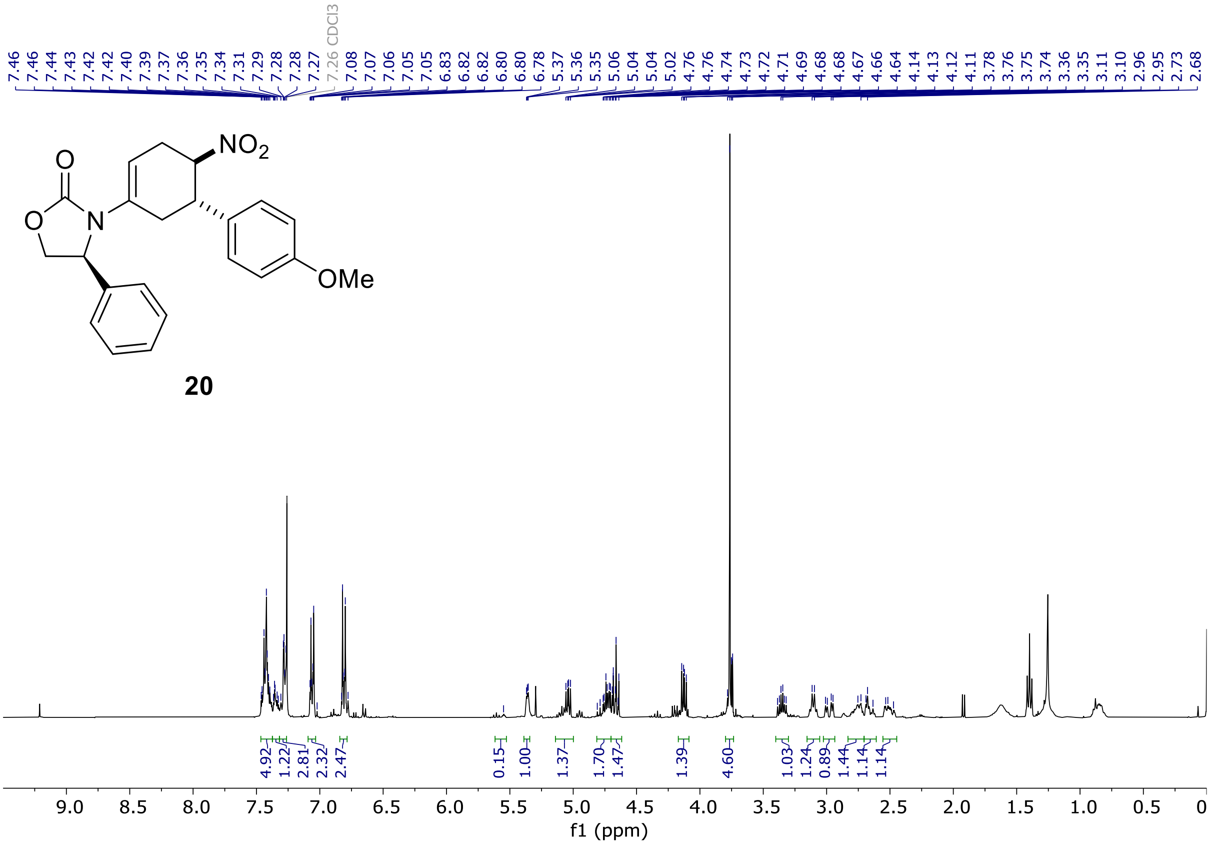

^{13}C {^{1}H} NMR (101 MHz, $CDCl_3$)

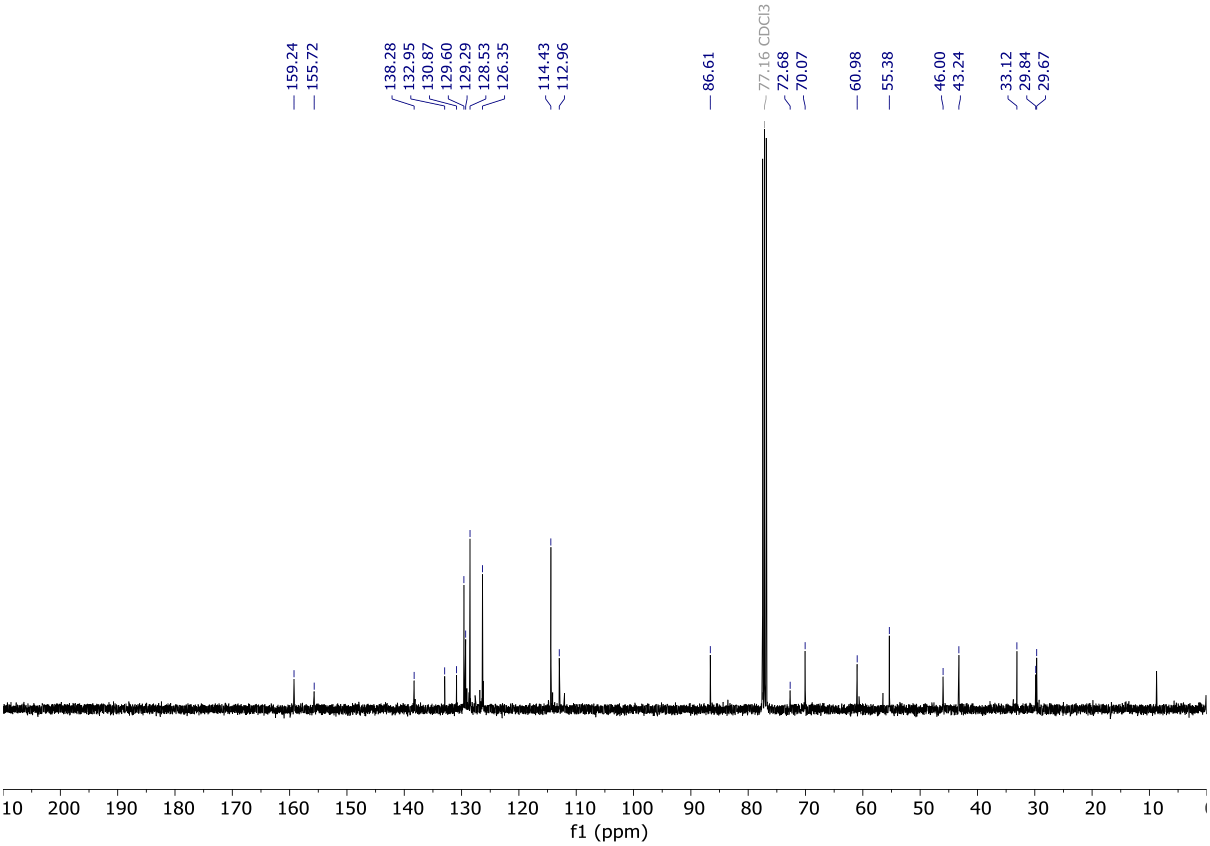

^{1}H NMR (500 MHz, CDCl$_3$)

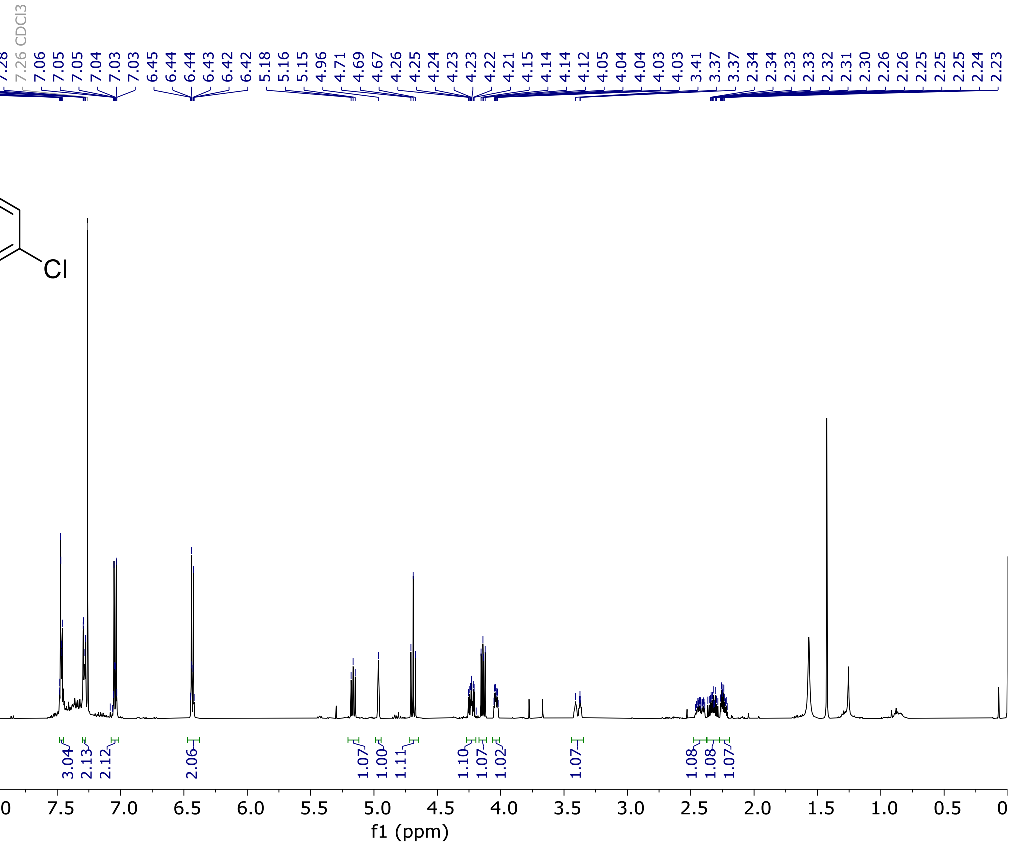

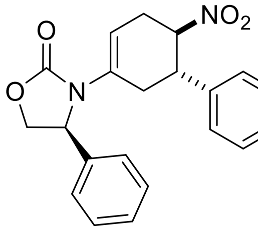

21

^{13}C {^{1}H} NMR (126 MHz, CDCl$_3$)

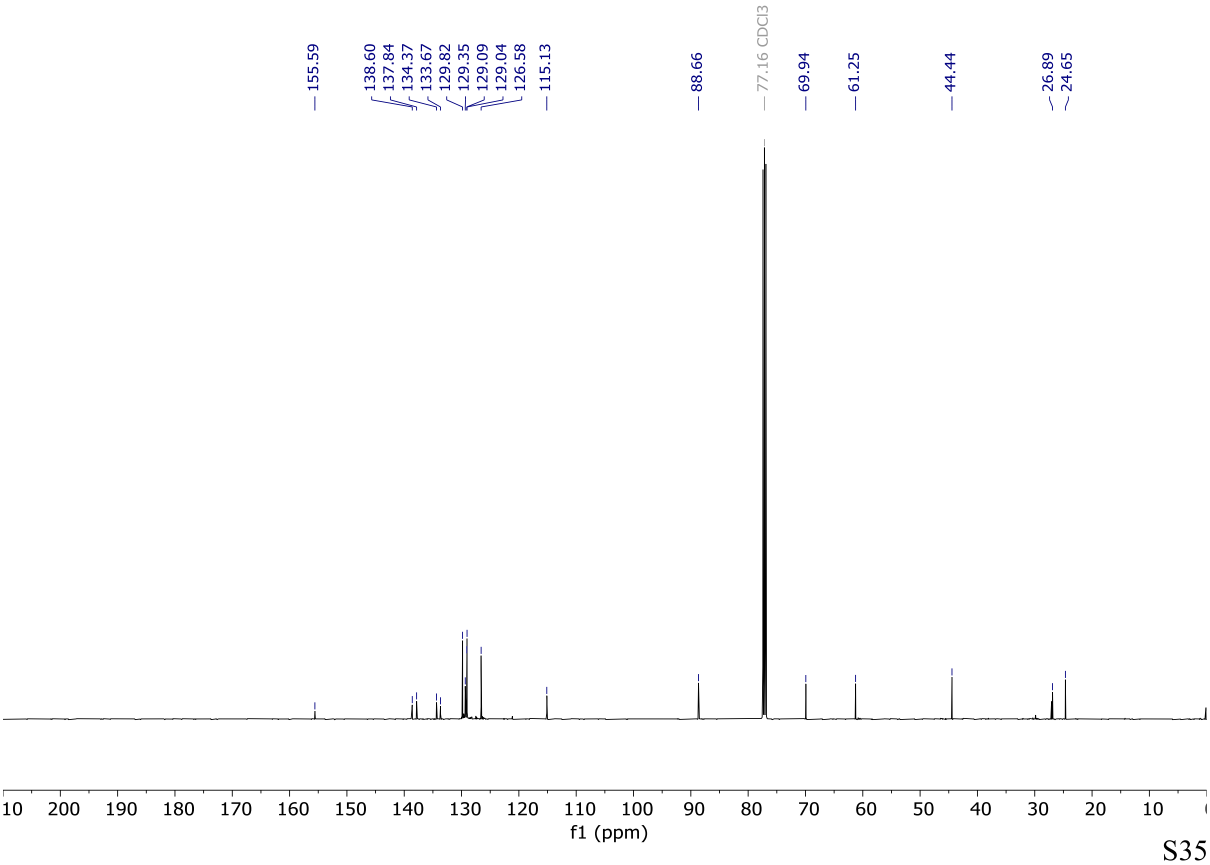

^{1}H NMR (400 MHz, $CDCl_3$)

22

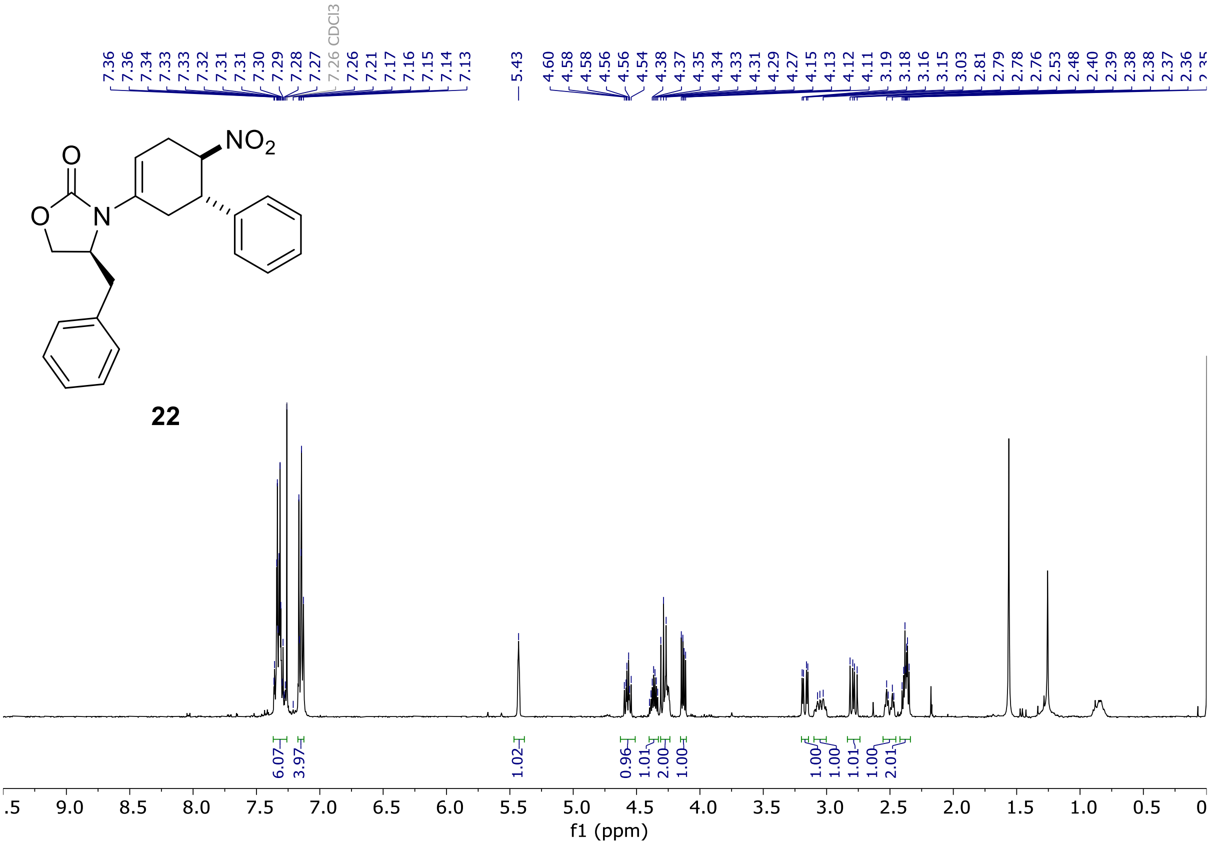

^{13}C {^{1}H} NMR (101 MHz, $CDCl_3$)

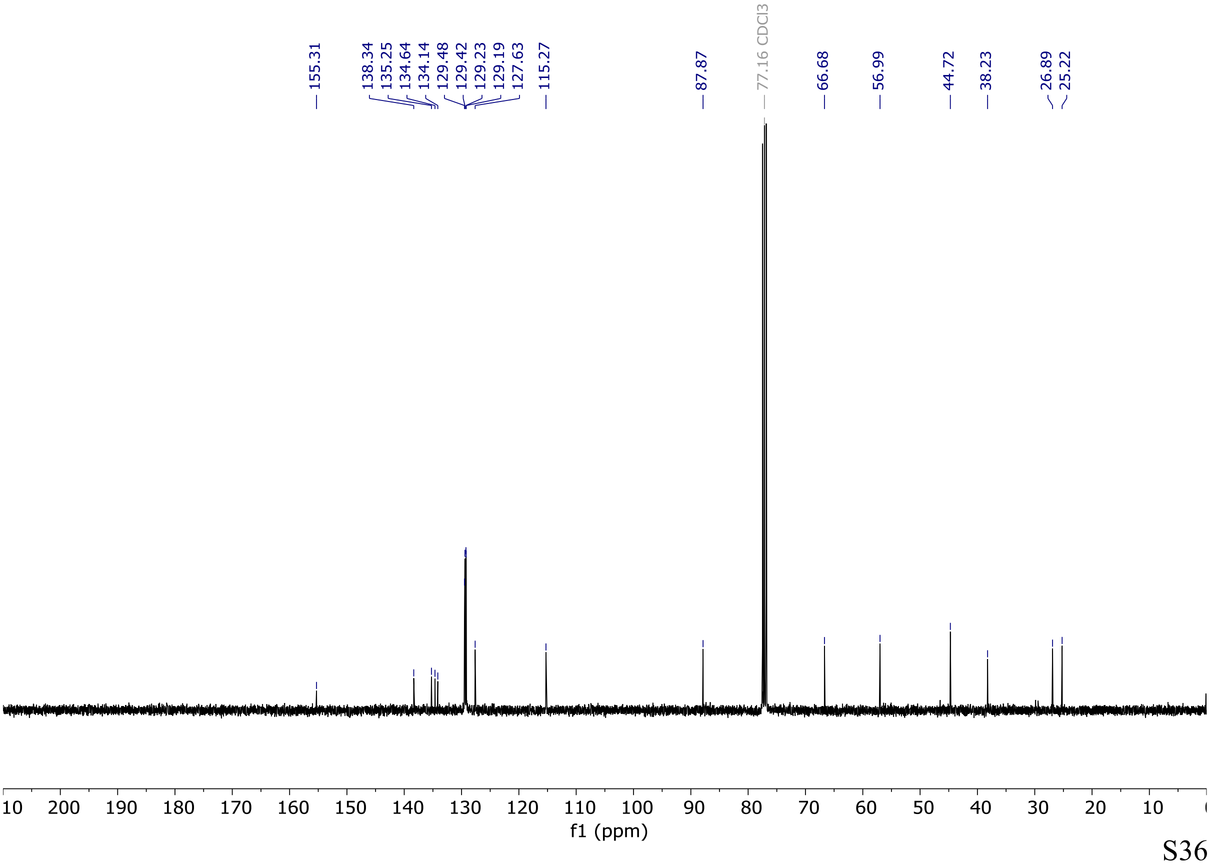

^{1}H NMR (500 MHz, CDCl$_3$)

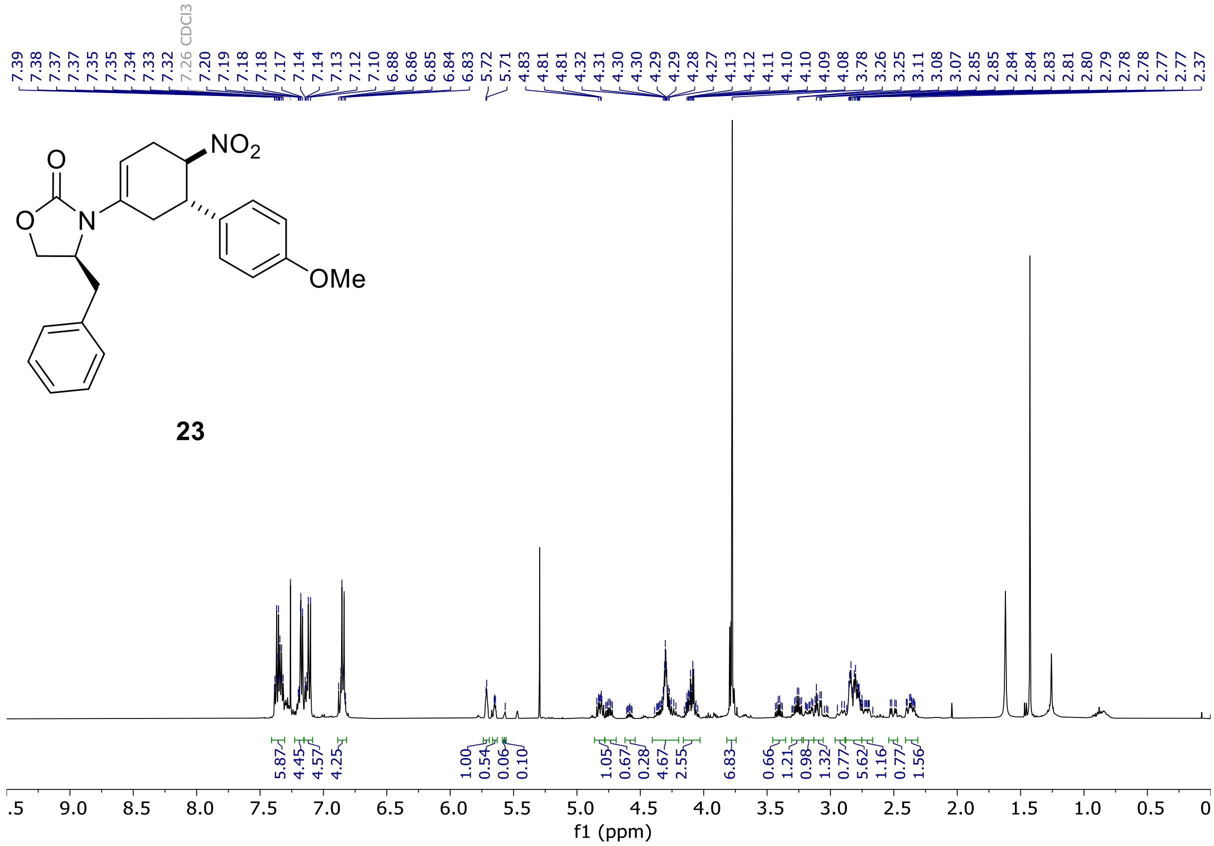

23

^{13}C {^{1}H} NMR (126 MHz, CDCl$_3$)

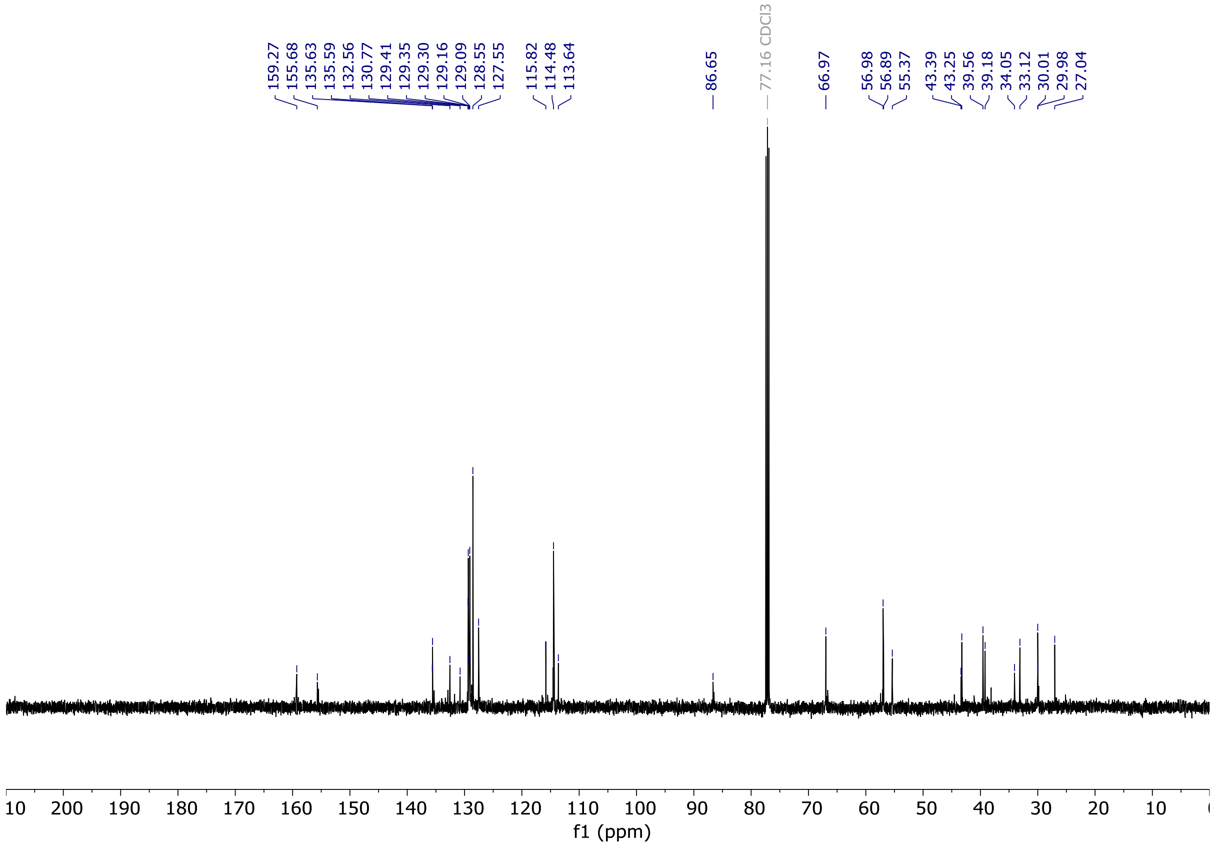

^{1}H NMR (400 MHz, $CDCl_3$)

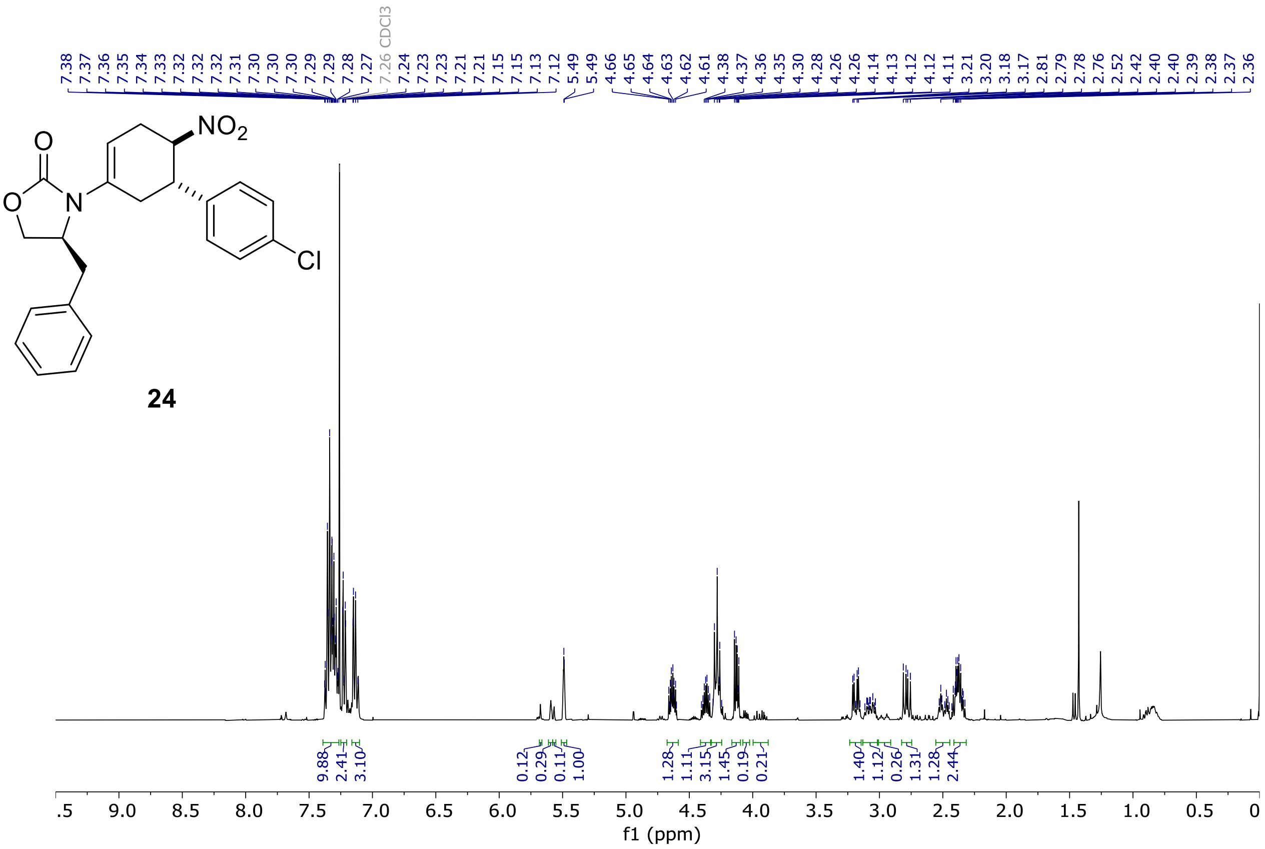

24

$^{13}C\{^{1}H\}$ NMR (101 MHz, $CDCl_3$)

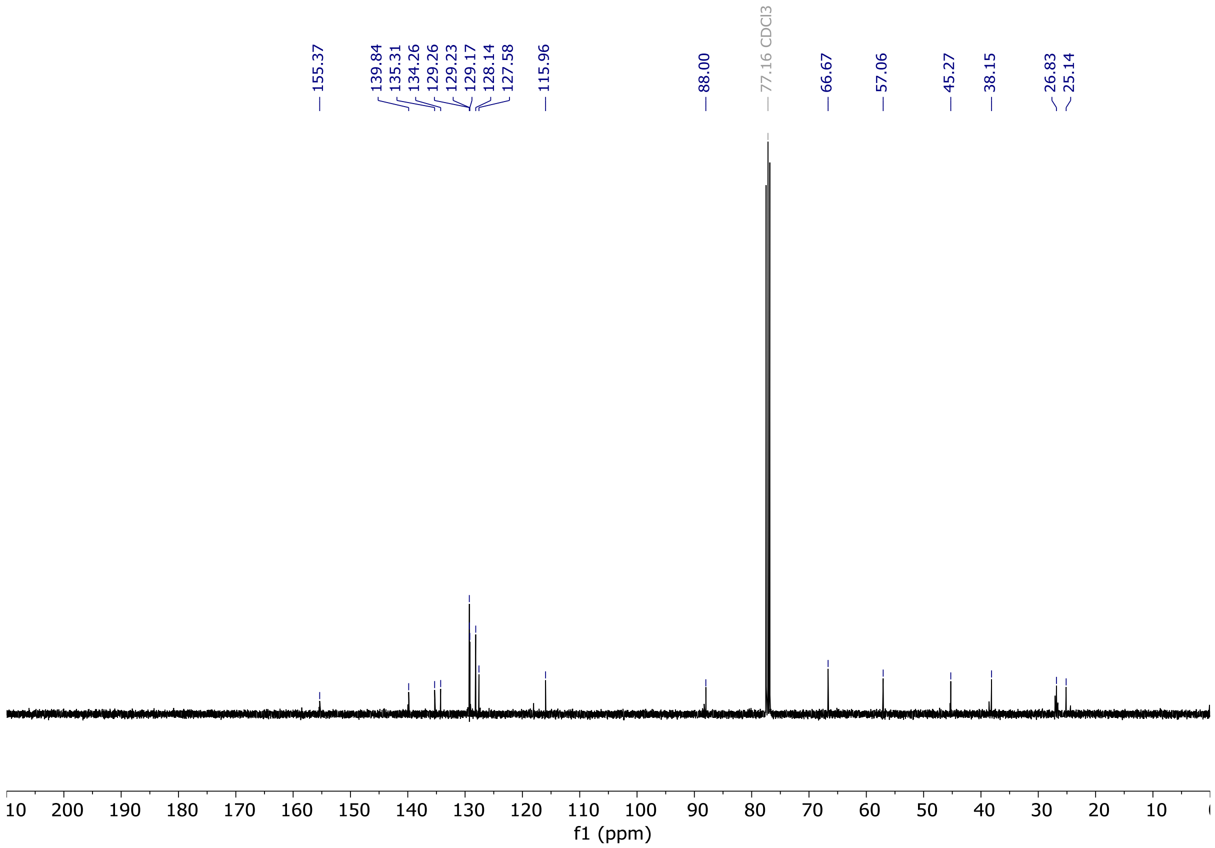

^{1}H NMR (500 MHz, $CDCl_3$)

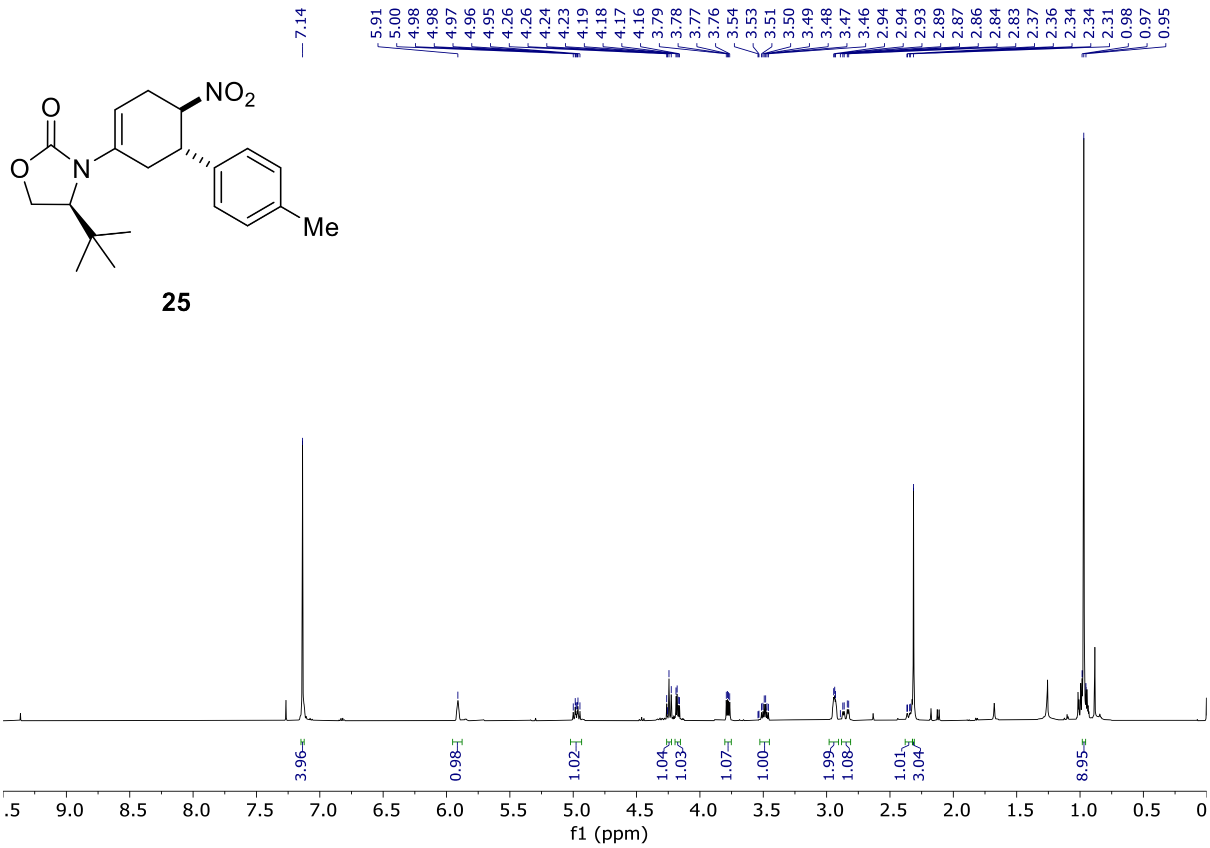

25

^{13}C {^{1}H} NMR (126 MHz, $CDCl_3$)

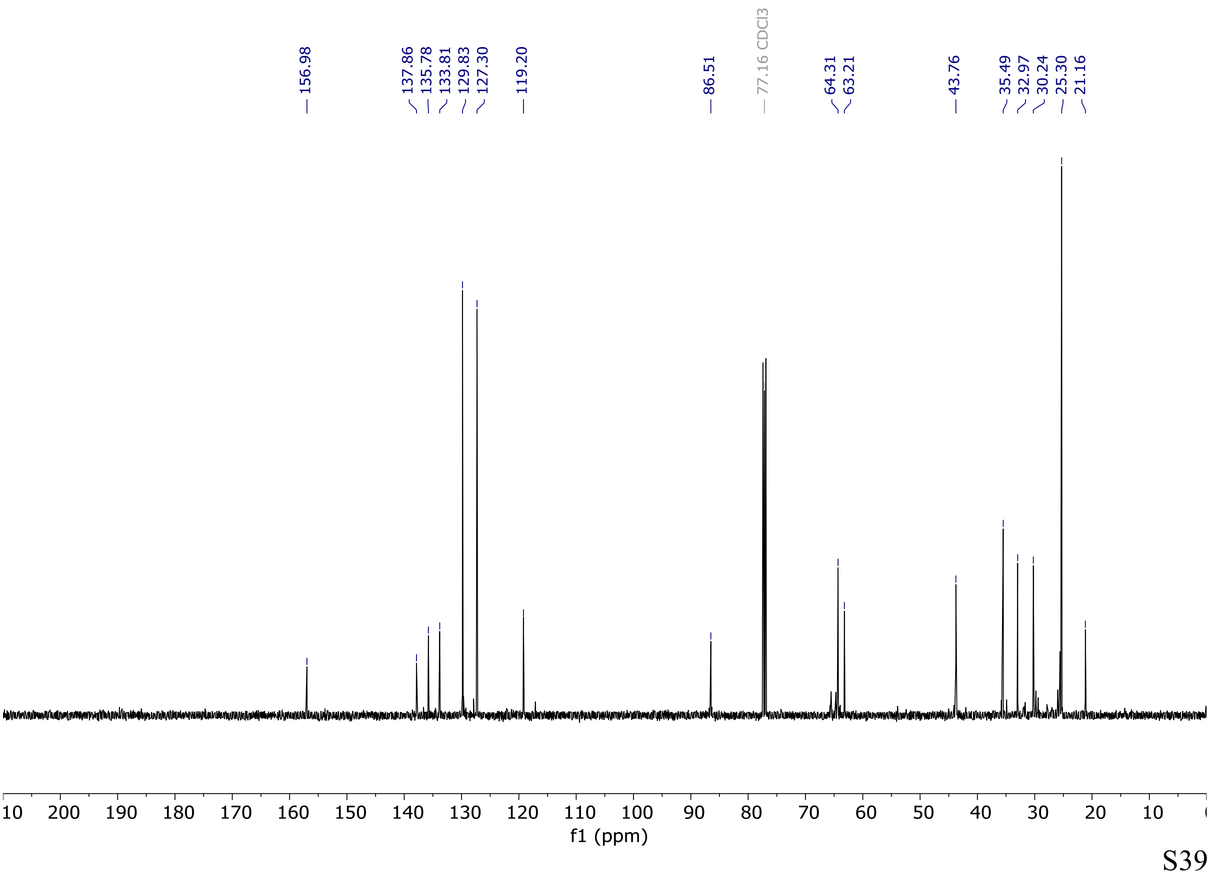

^{1}H NMR (400 MHz, $CDCl_3$)

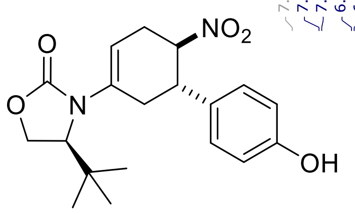

26

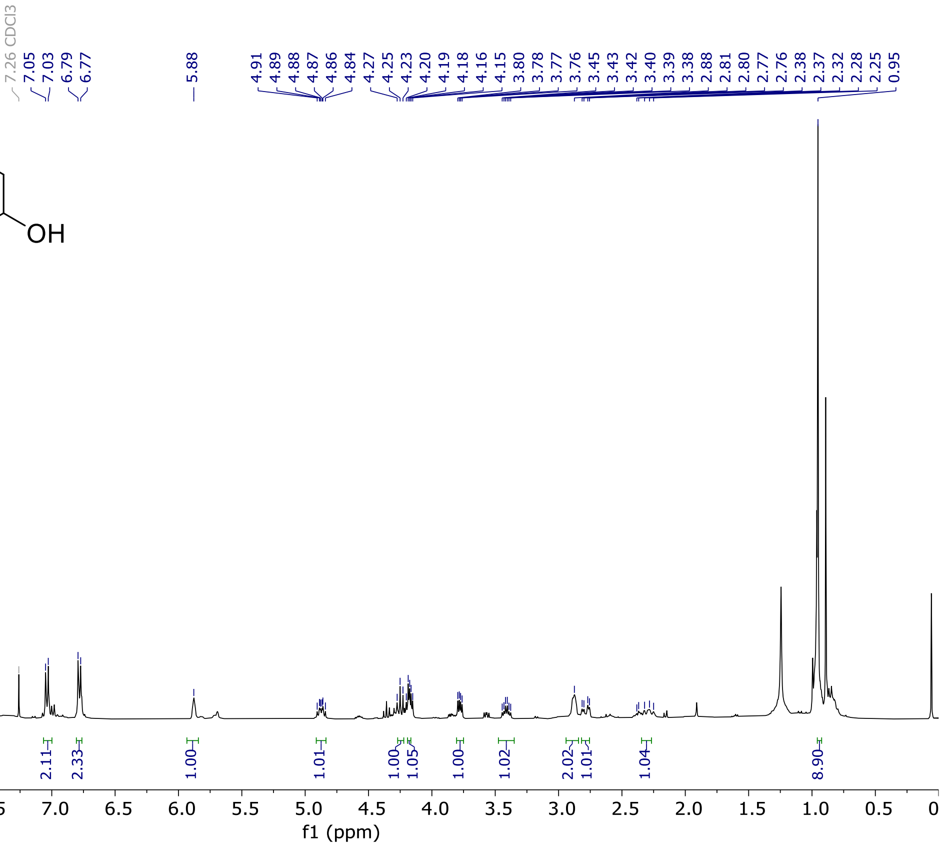

^{13}C {^{1}H} NMR (126 MHz, $CDCl_3$)

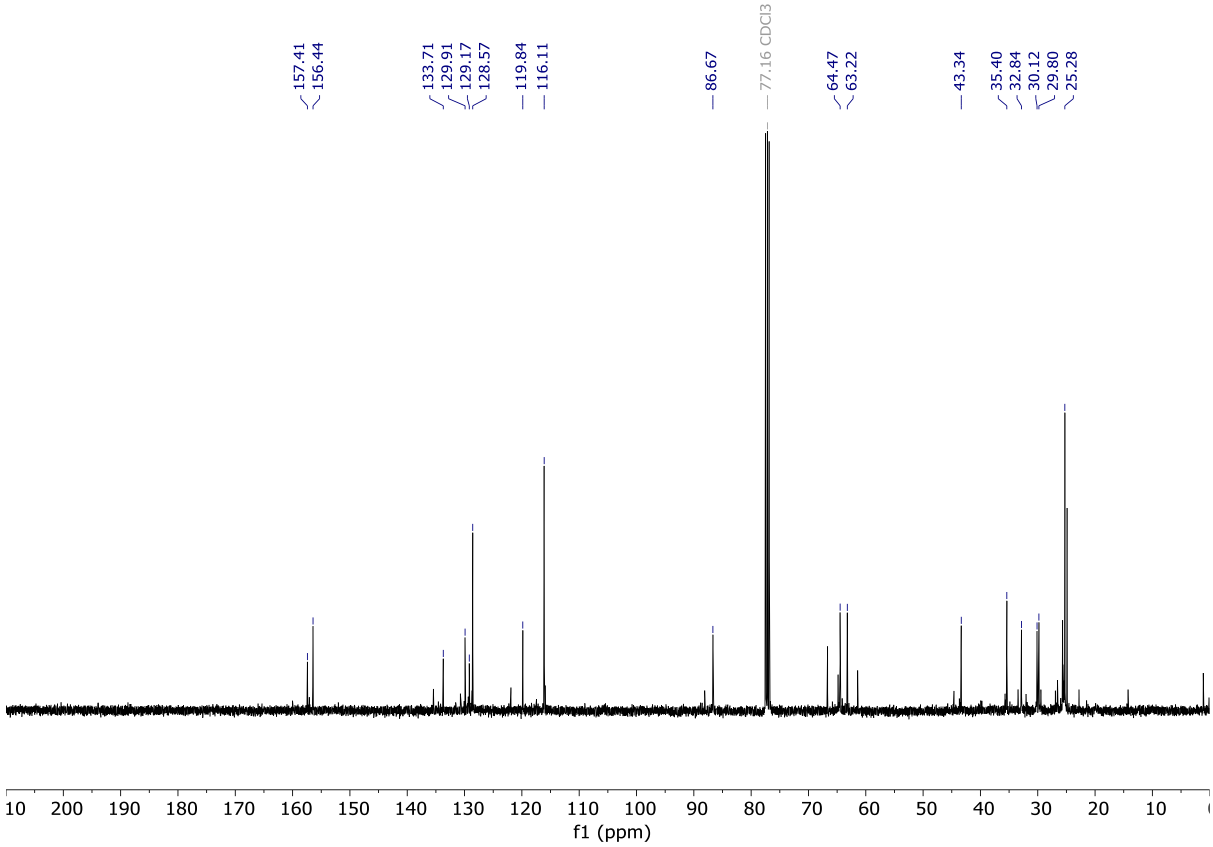

^{1}H NMR (500 MHz, $CDCl_3$)

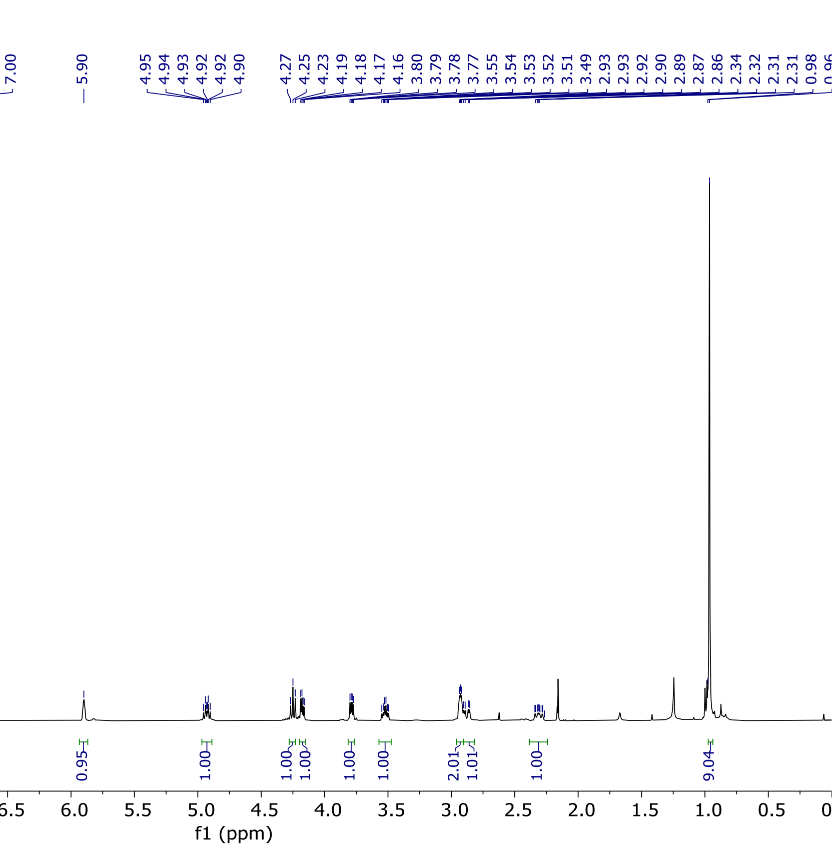

27

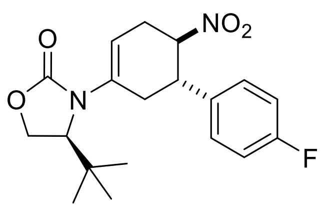

^{13}C {^{1}H} NMR (101 MHz, $CDCl_3$)

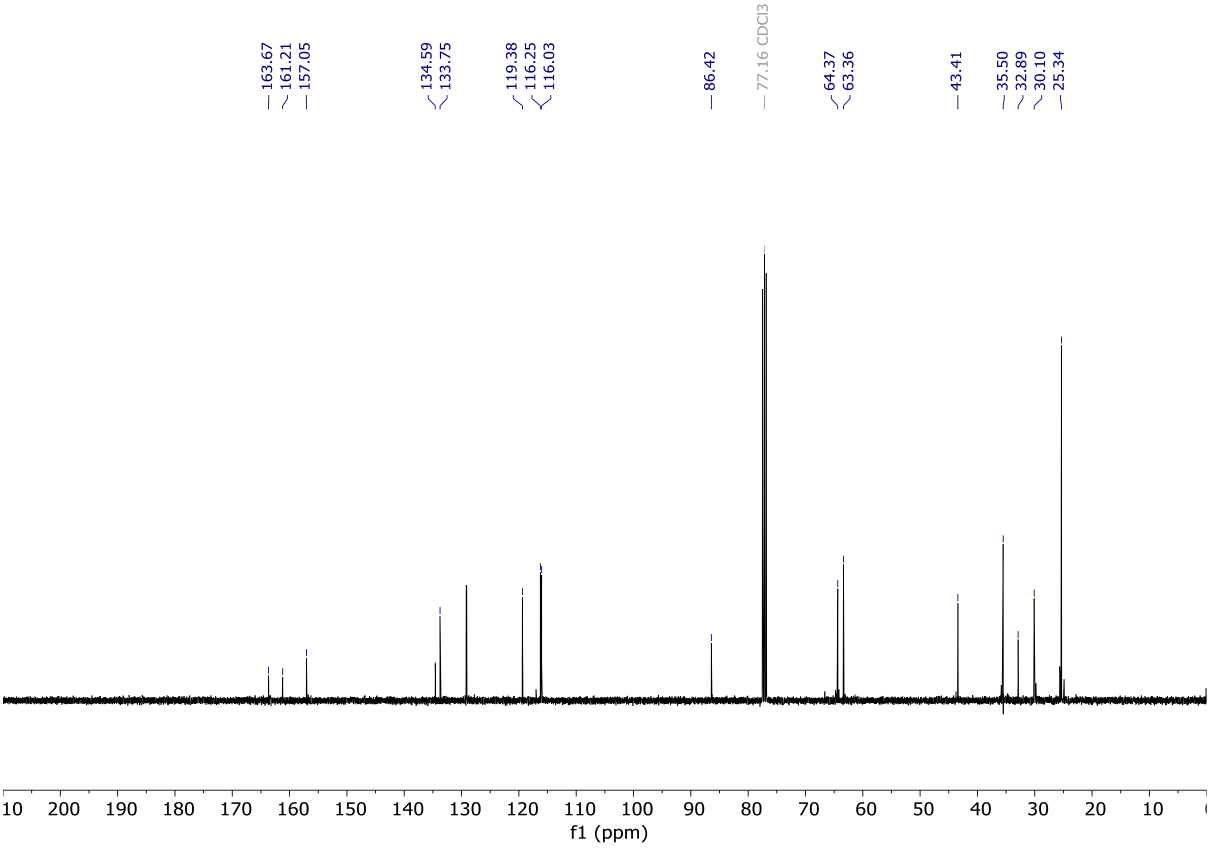

^{1}H NMR (500 MHz, $CDCl_3$)

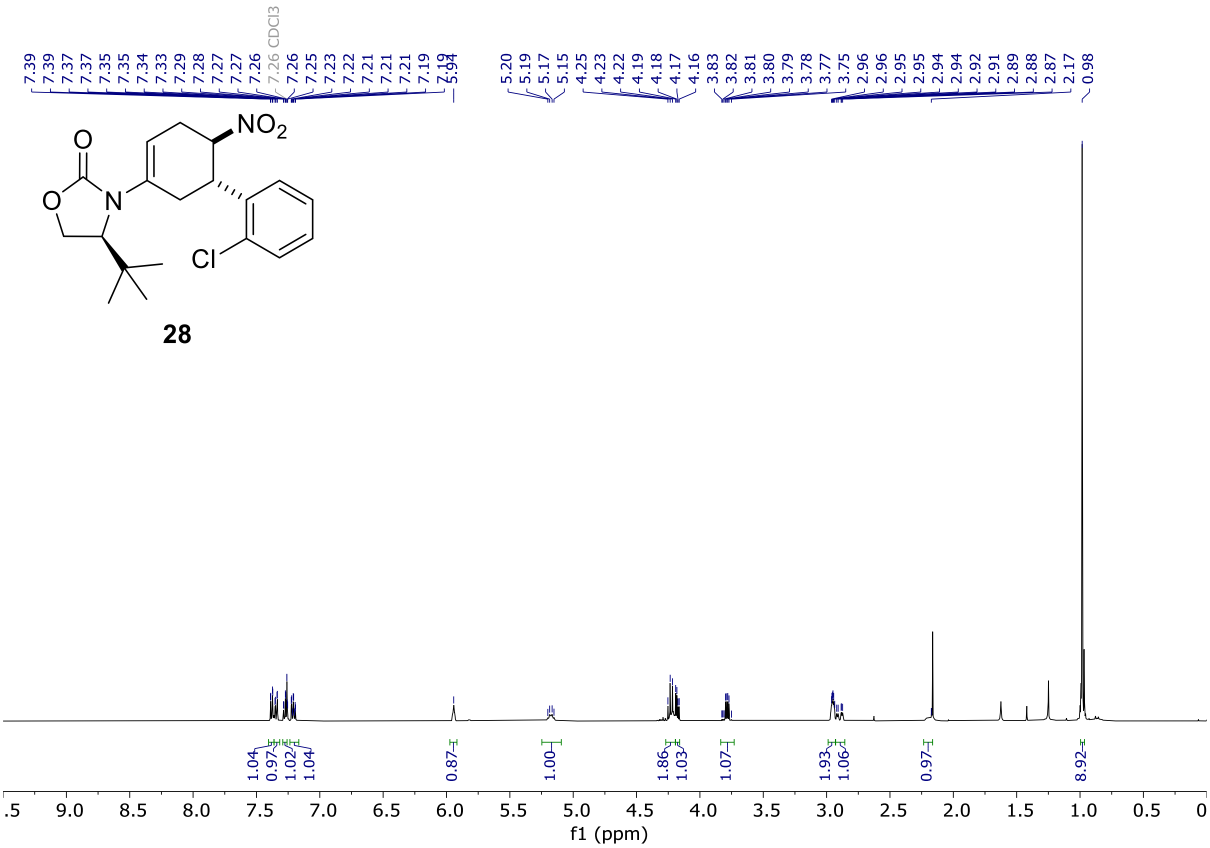

28

^{13}C {^{1}H} NMR (126 MHz, $CDCl_3$)

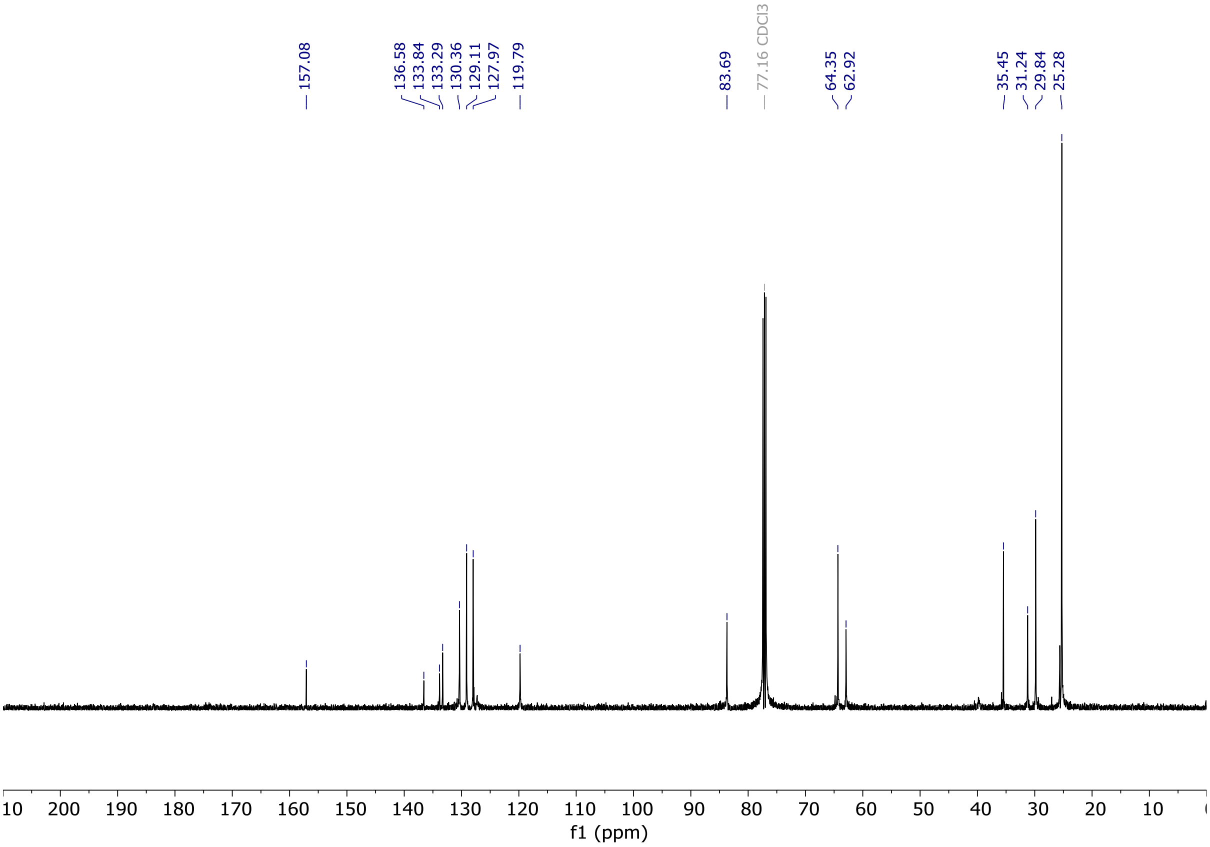

^{1}H NMR (400 MHz, $CDCl_3$)

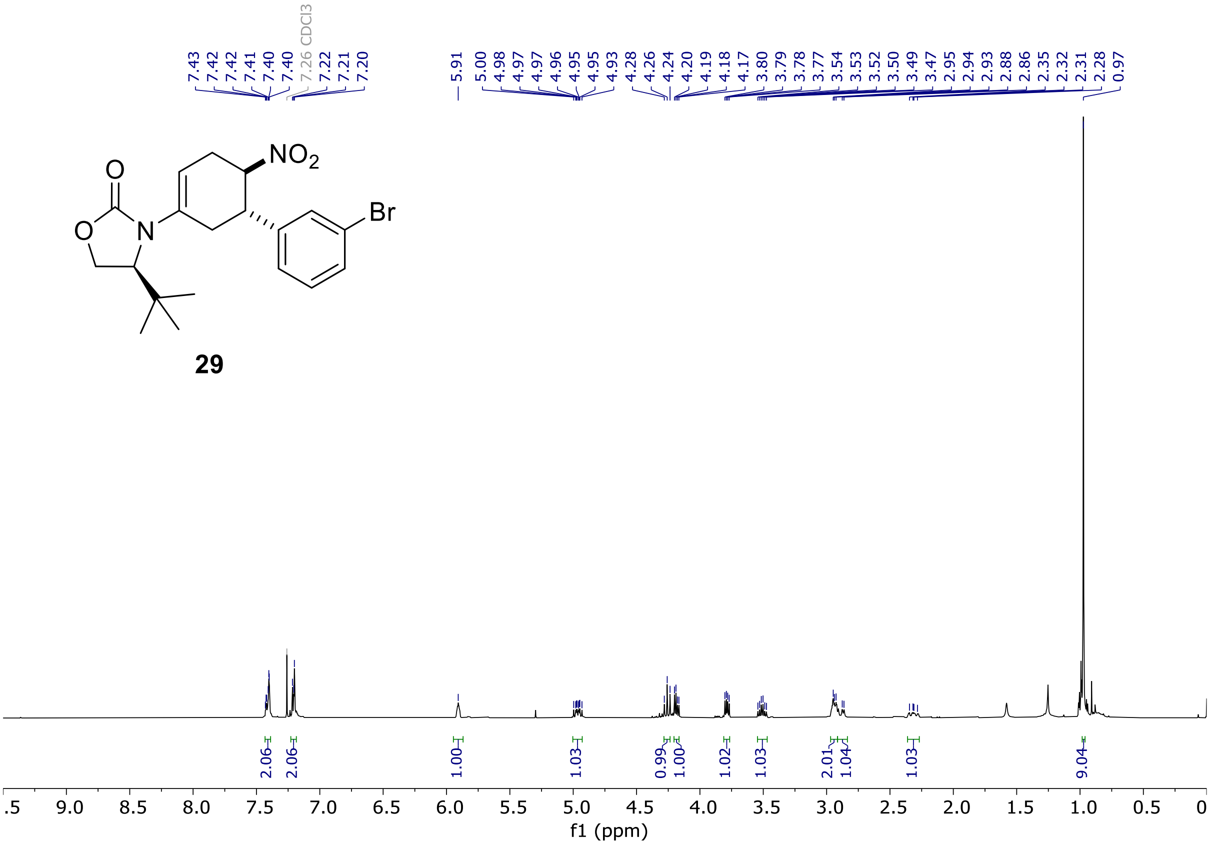

^{13}C {^{1}H} NMR (101 MHz, $CDCl_3$)

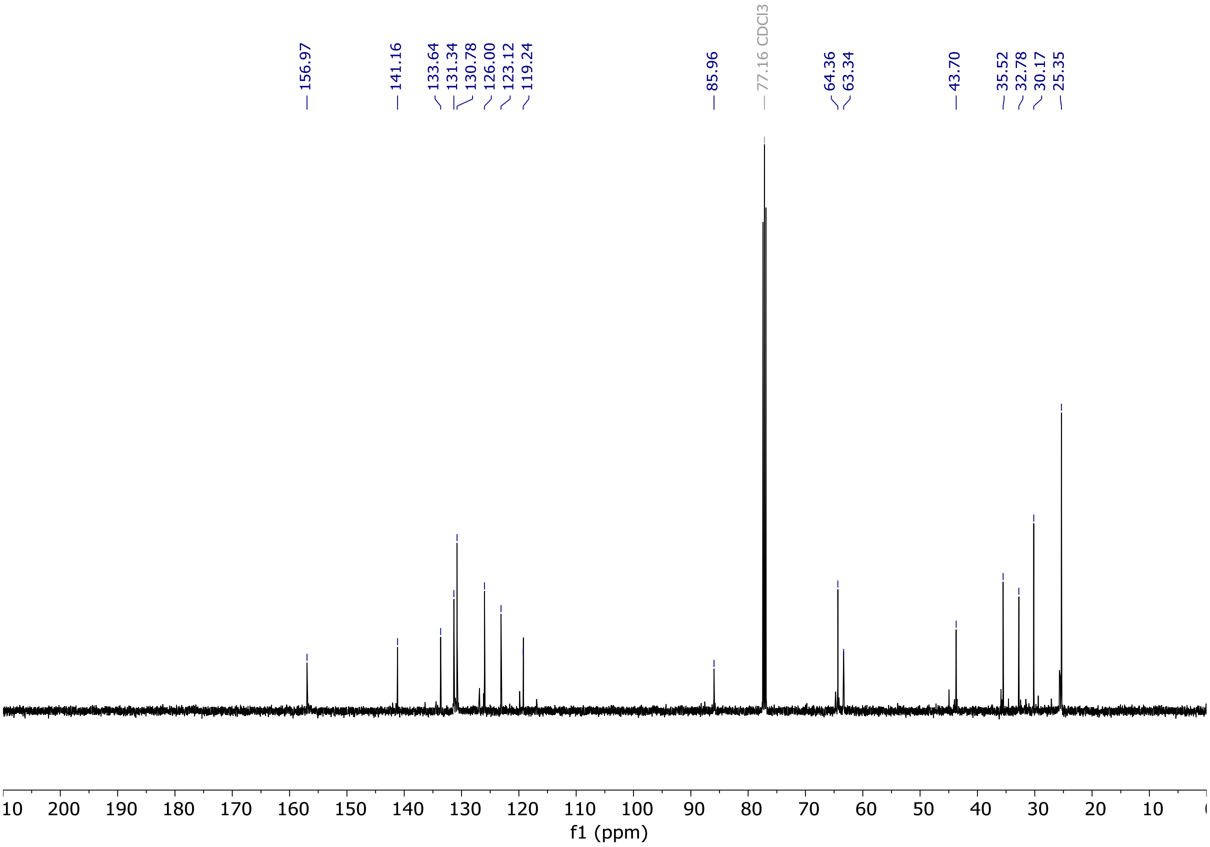

^{1}H NMR (400 MHz, $CDCl_3$)

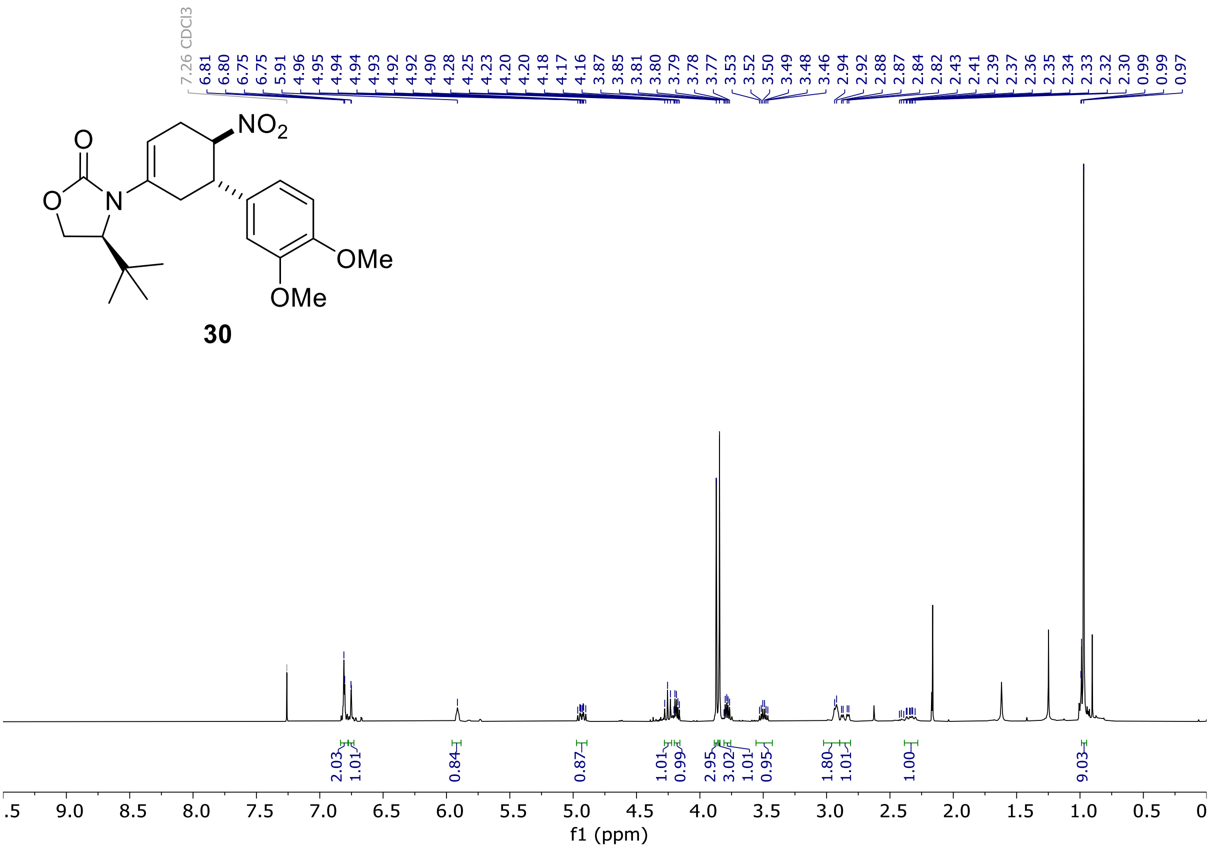

30

^{13}C {^{1}H} NMR (101 MHz, $CDCl_3$)

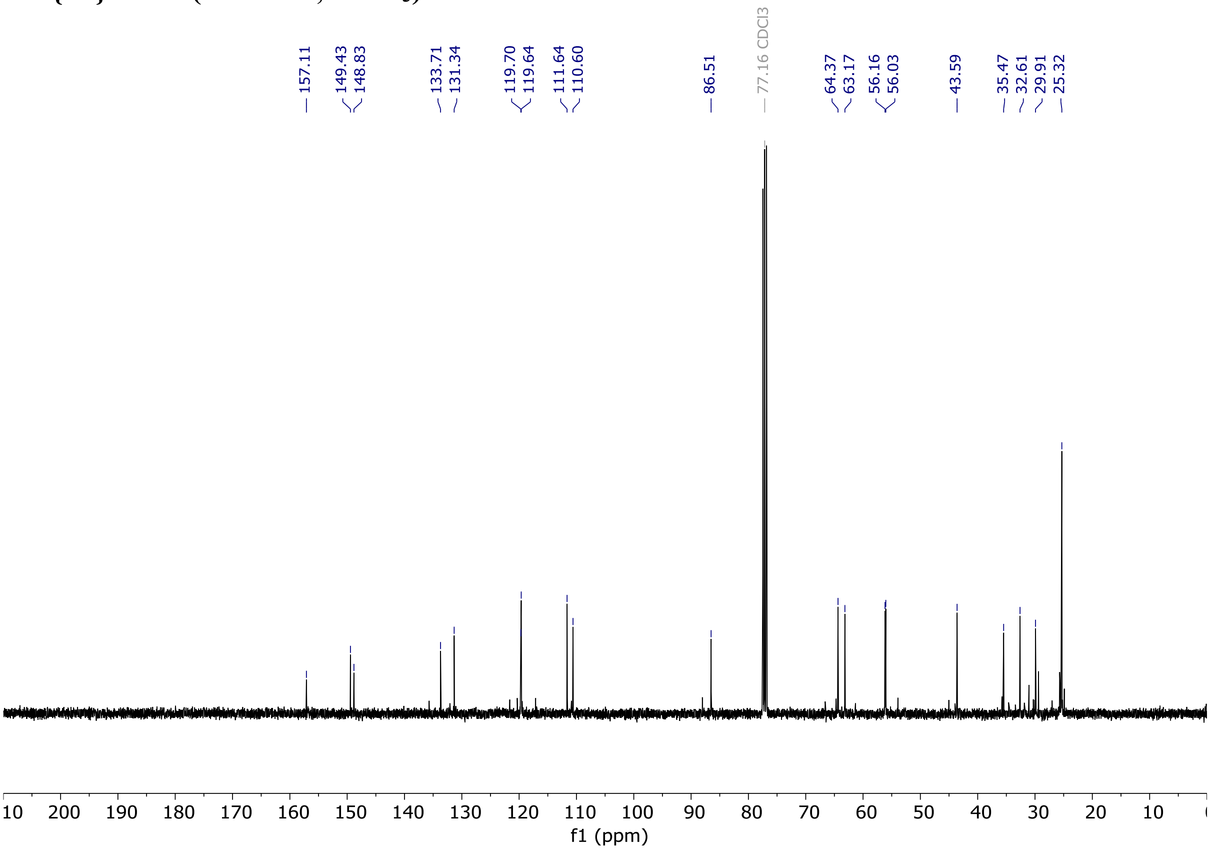

^{1}H NMR (400 MHz, $CDCl_3$)

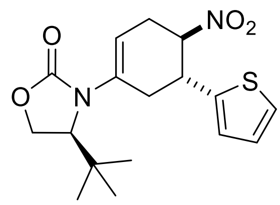

31

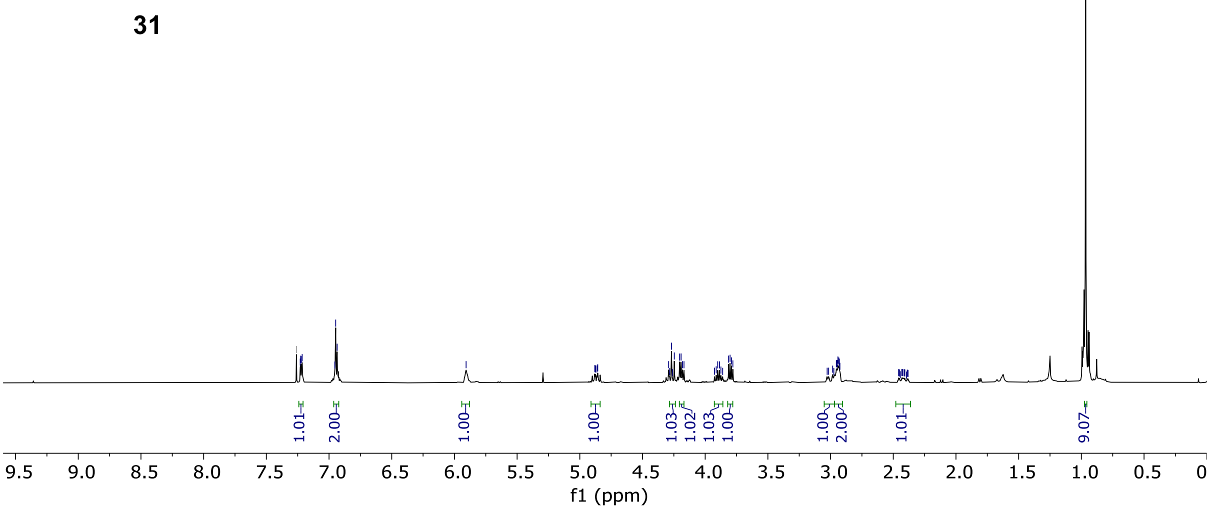

^{13}C {^{1}H} NMR (101 MHz, $CDCl_3$)

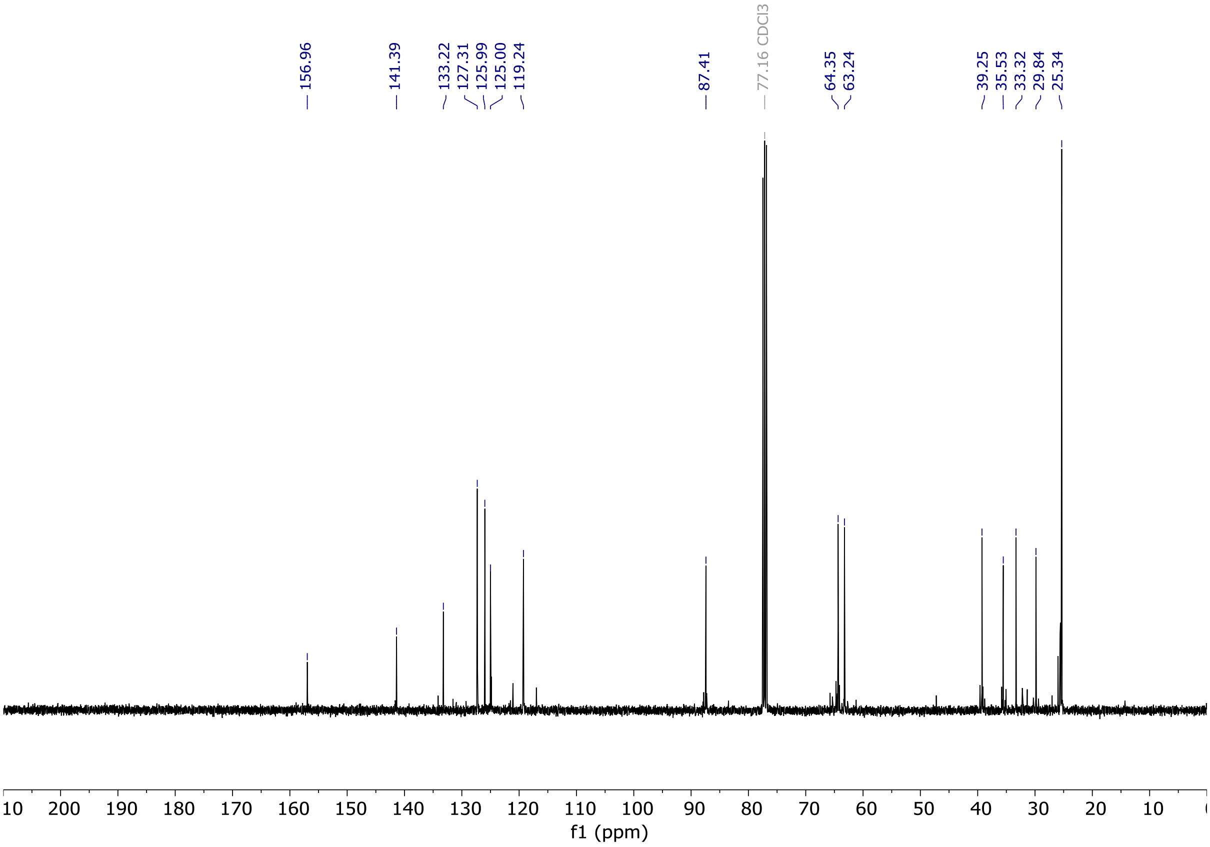

^{1}H NMR (400 MHz, $CDCl_3$)

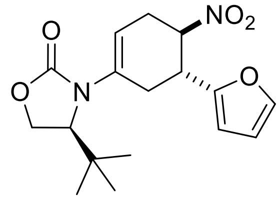

32

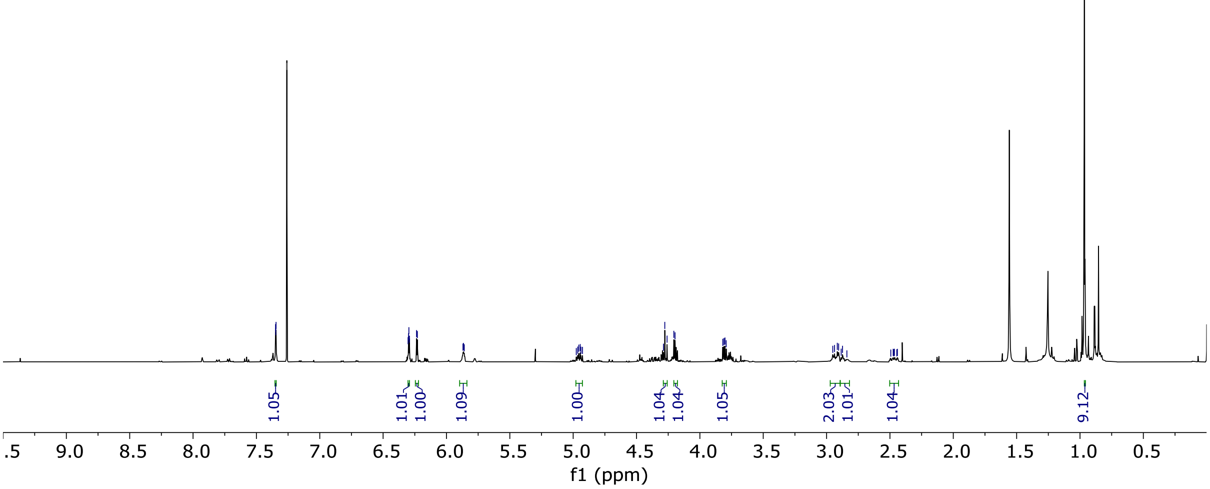

^{13}C {^{1}H} NMR (101 MHz, $CDCl_3$)

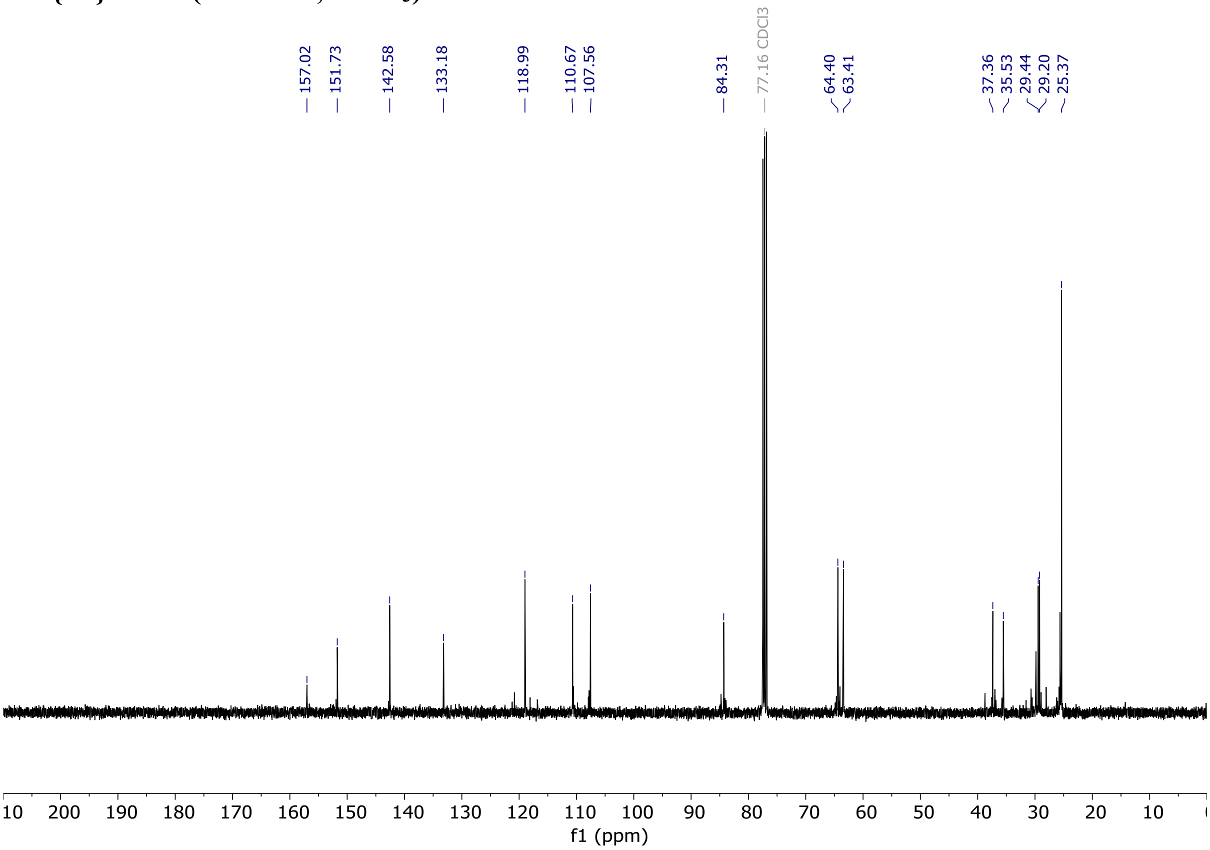

^{1}H NMR (500 MHz, $CDCl_3$)

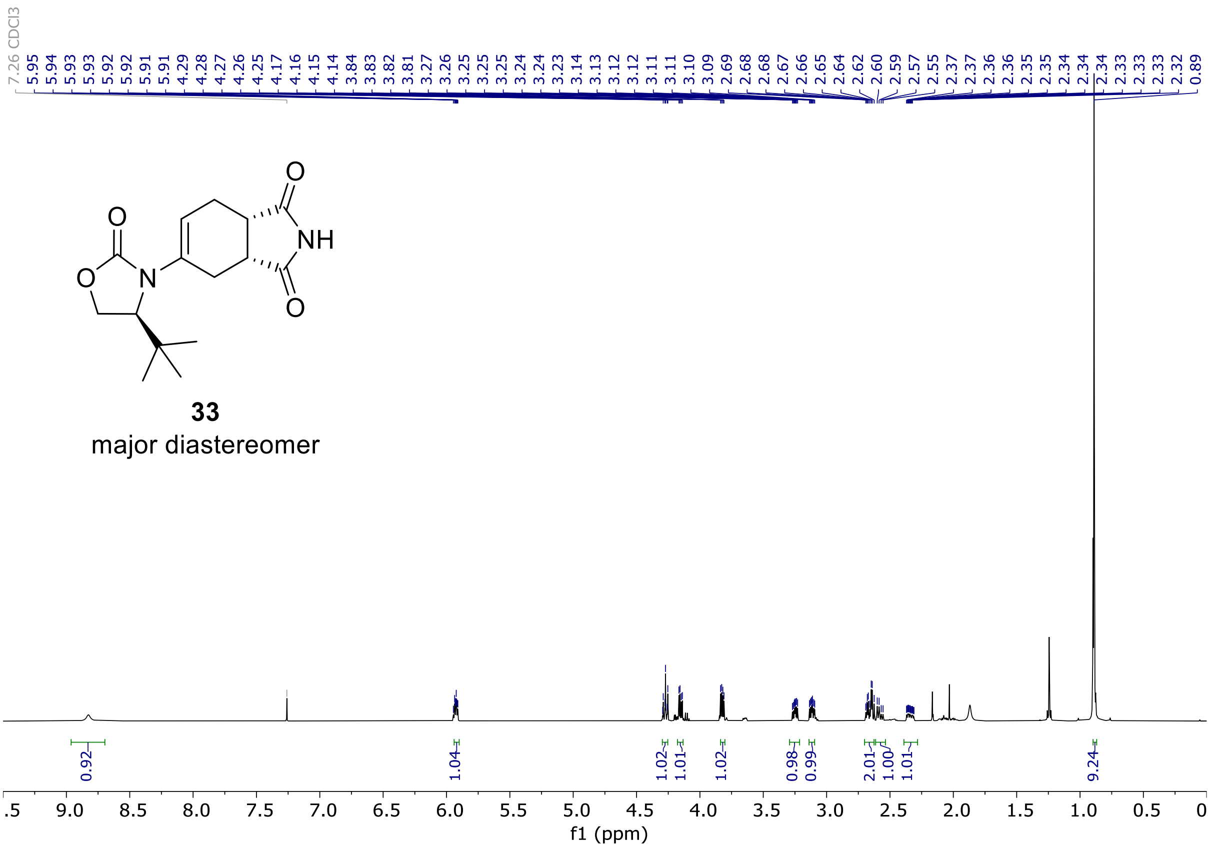

33
major diastereomer

^{13}C {^{1}H} NMR (126 MHz, $CDCl_3$)

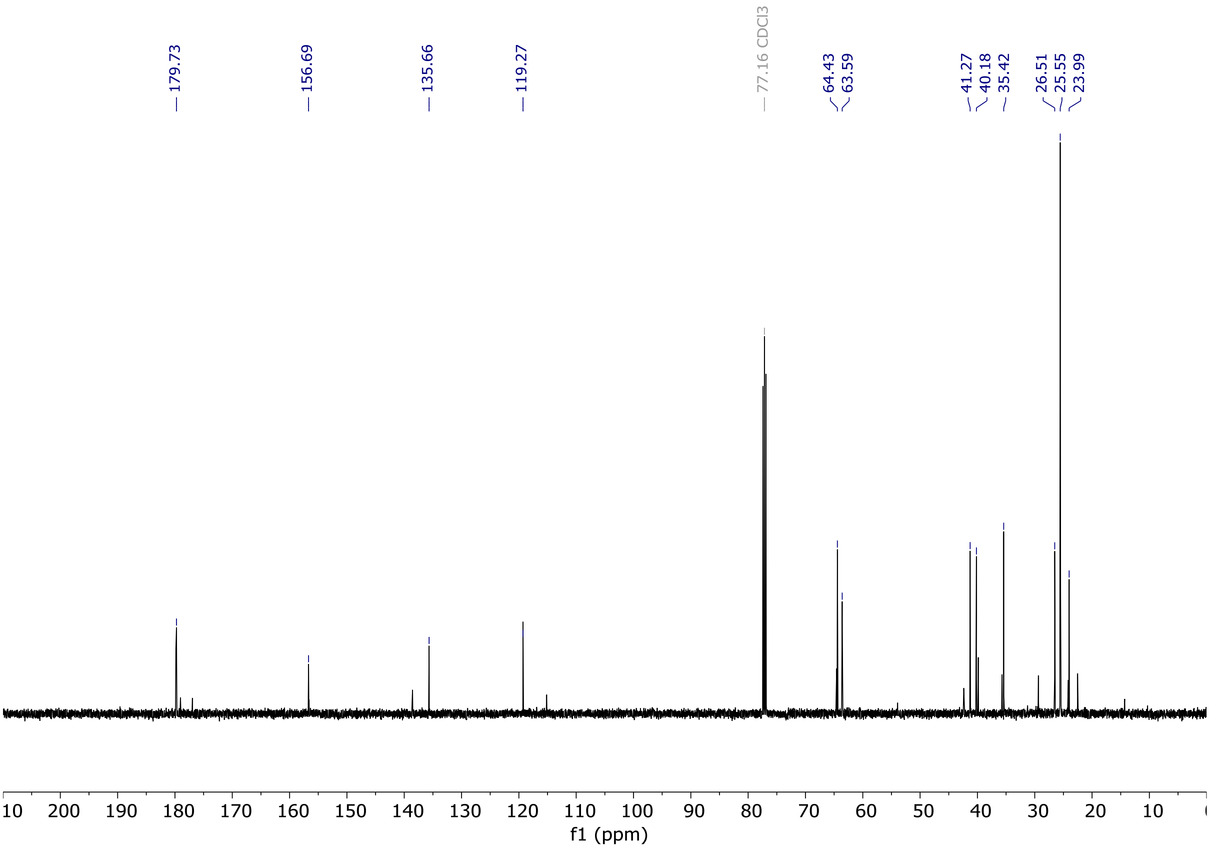

^{1}H NMR (500 MHz, $CDCl_3$)

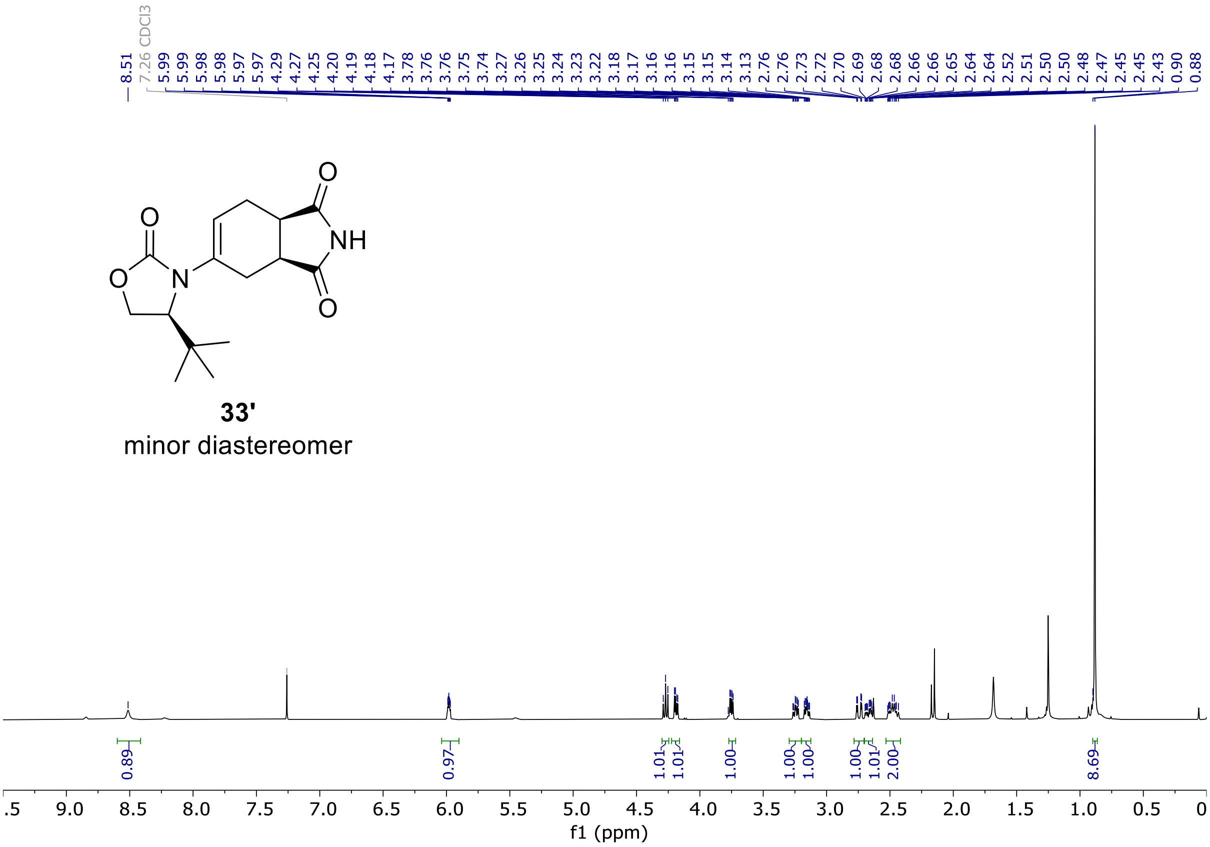

^{13}C {^{1}H} NMR (126 MHz, $CDCl_3$)

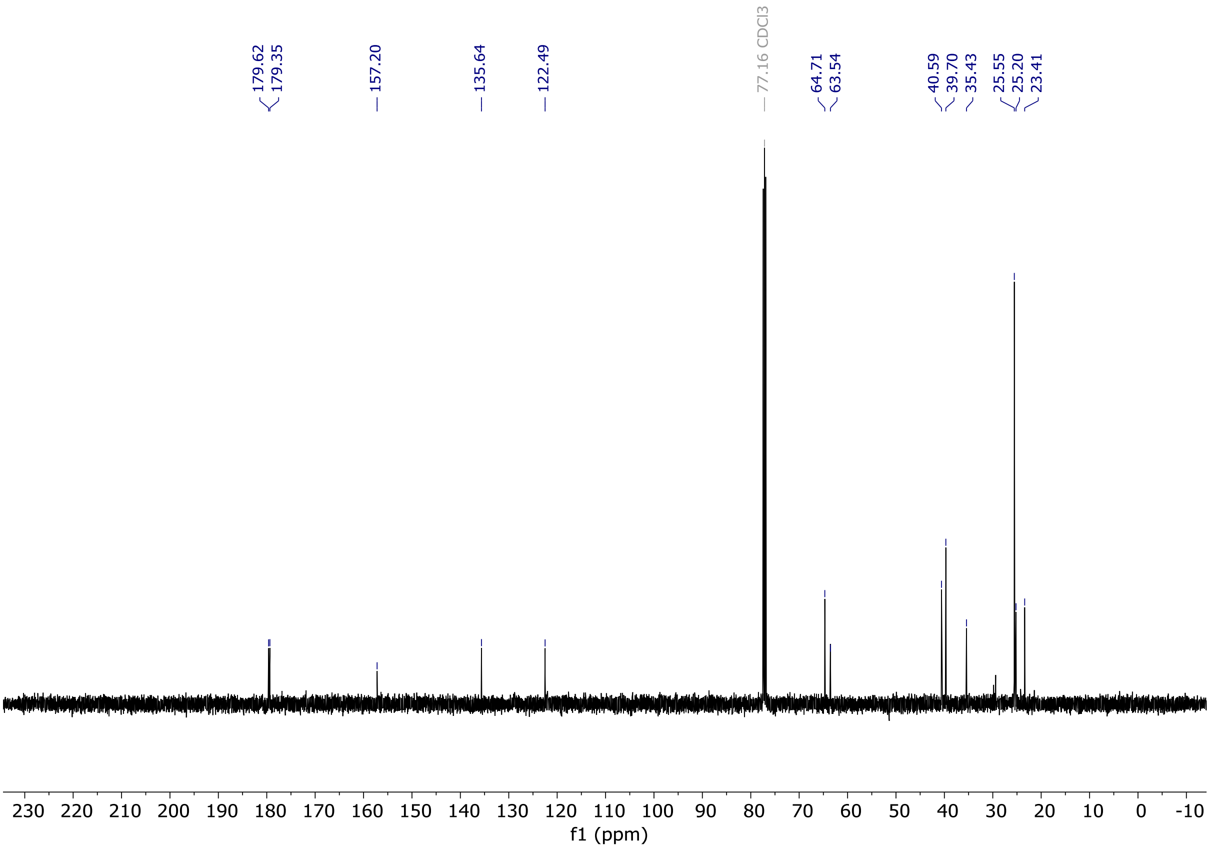

^{1}H NMR (400 MHz, $CDCl_3$)

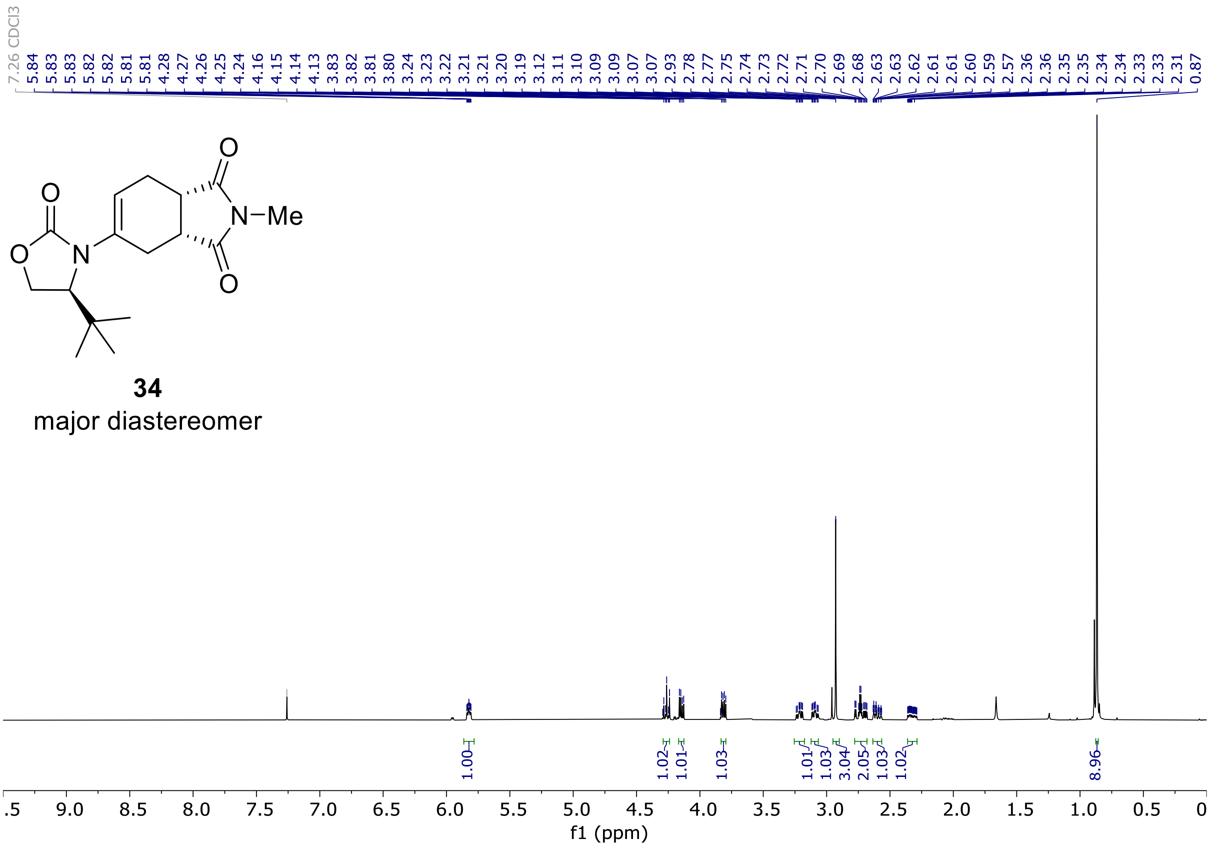

34

major diastereomer

^{13}C {^{1}H} NMR (101 MHz, $CDCl_3$)

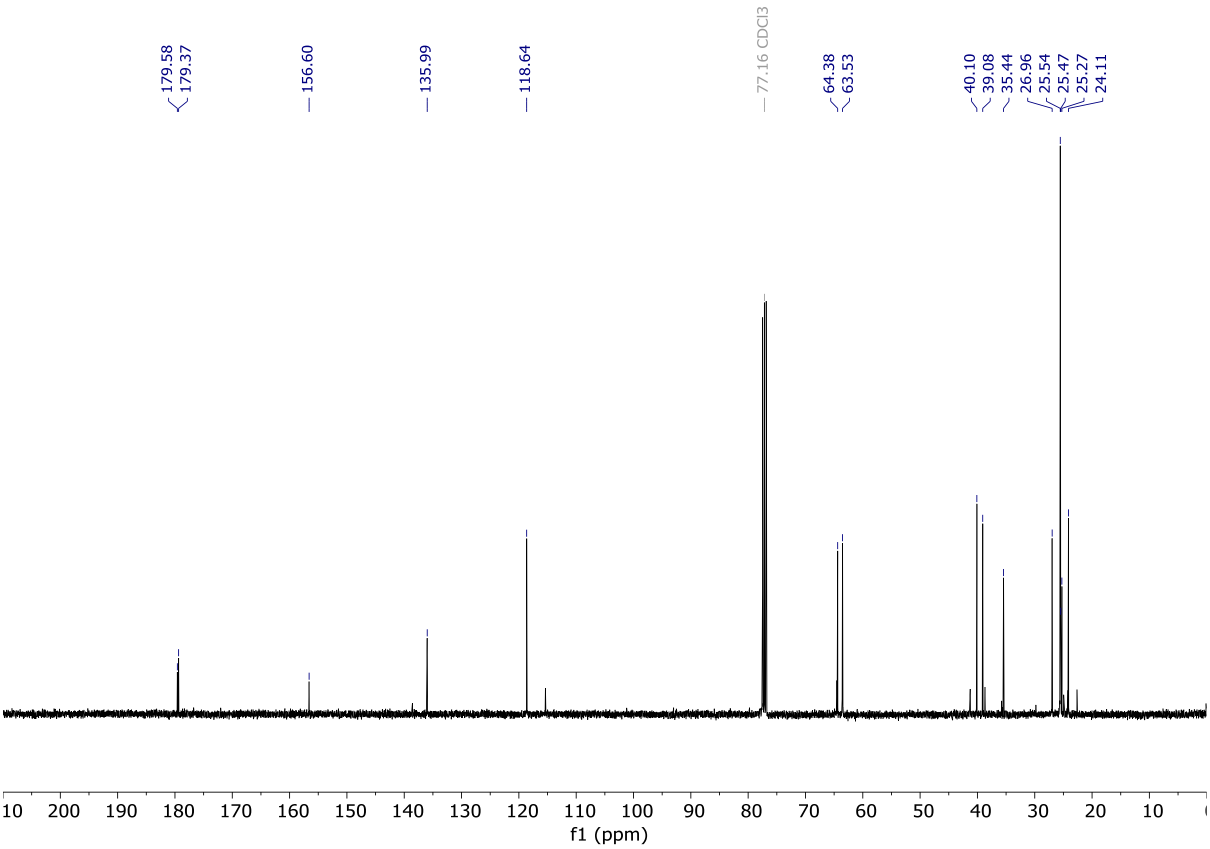

^{1}H NMR (500 MHz, CDCl$_3$)

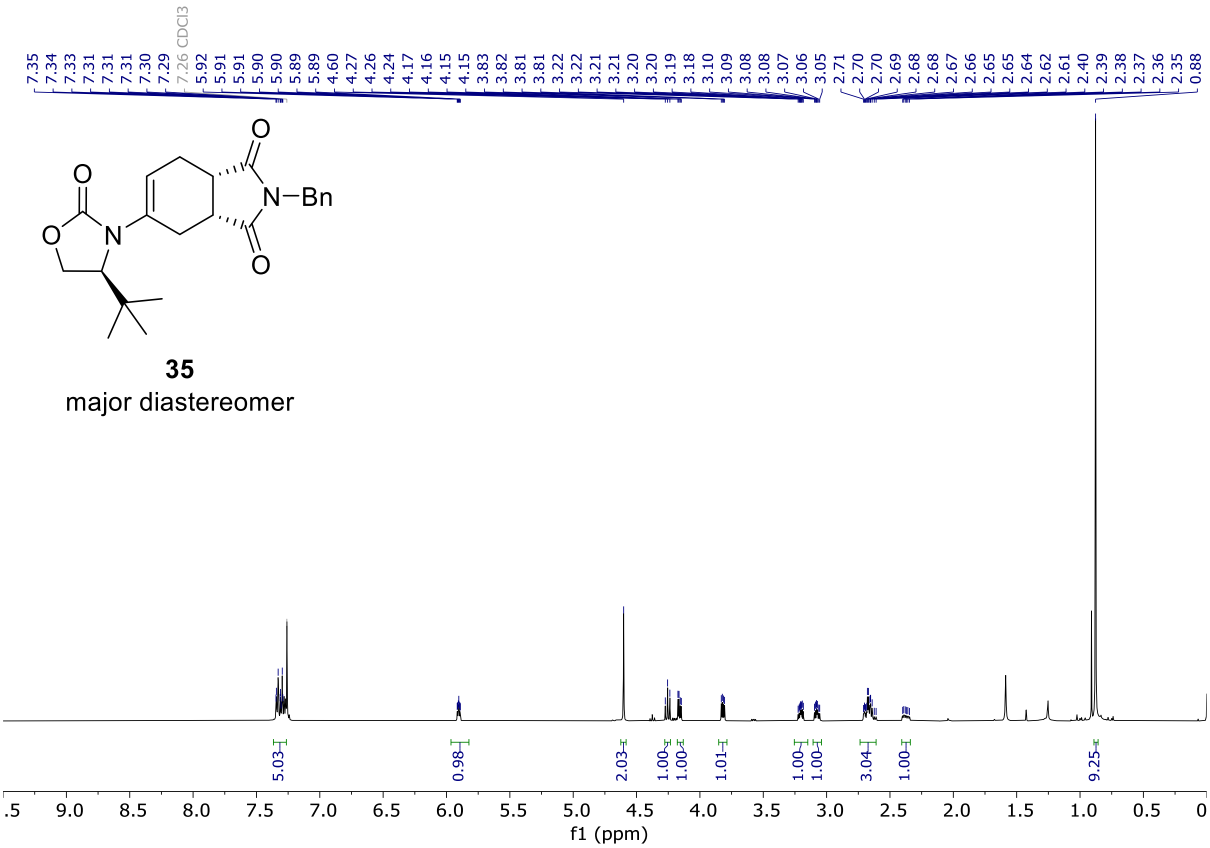

^{13}C {^{1}H} NMR (126 MHz, CDCl$_3$)

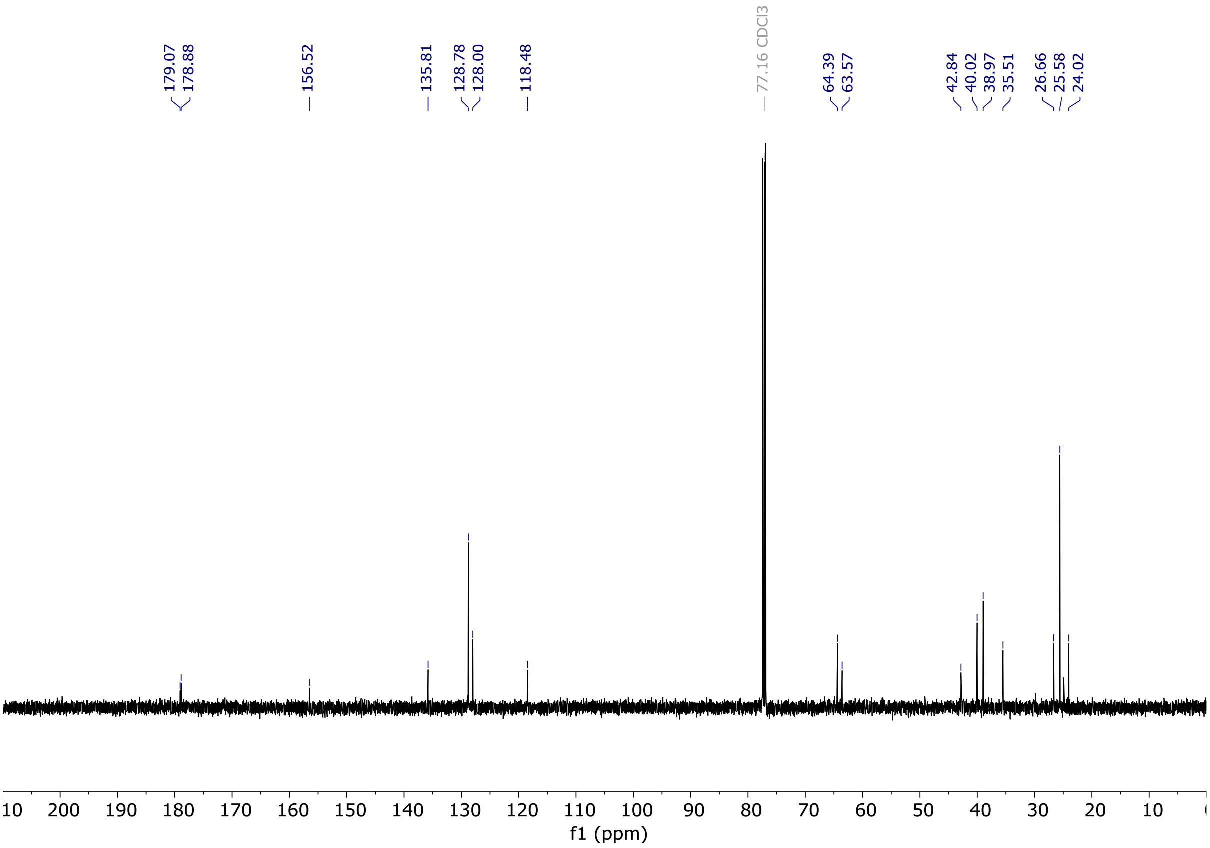

^{1}H NMR (500 MHz, $CDCl_3$)

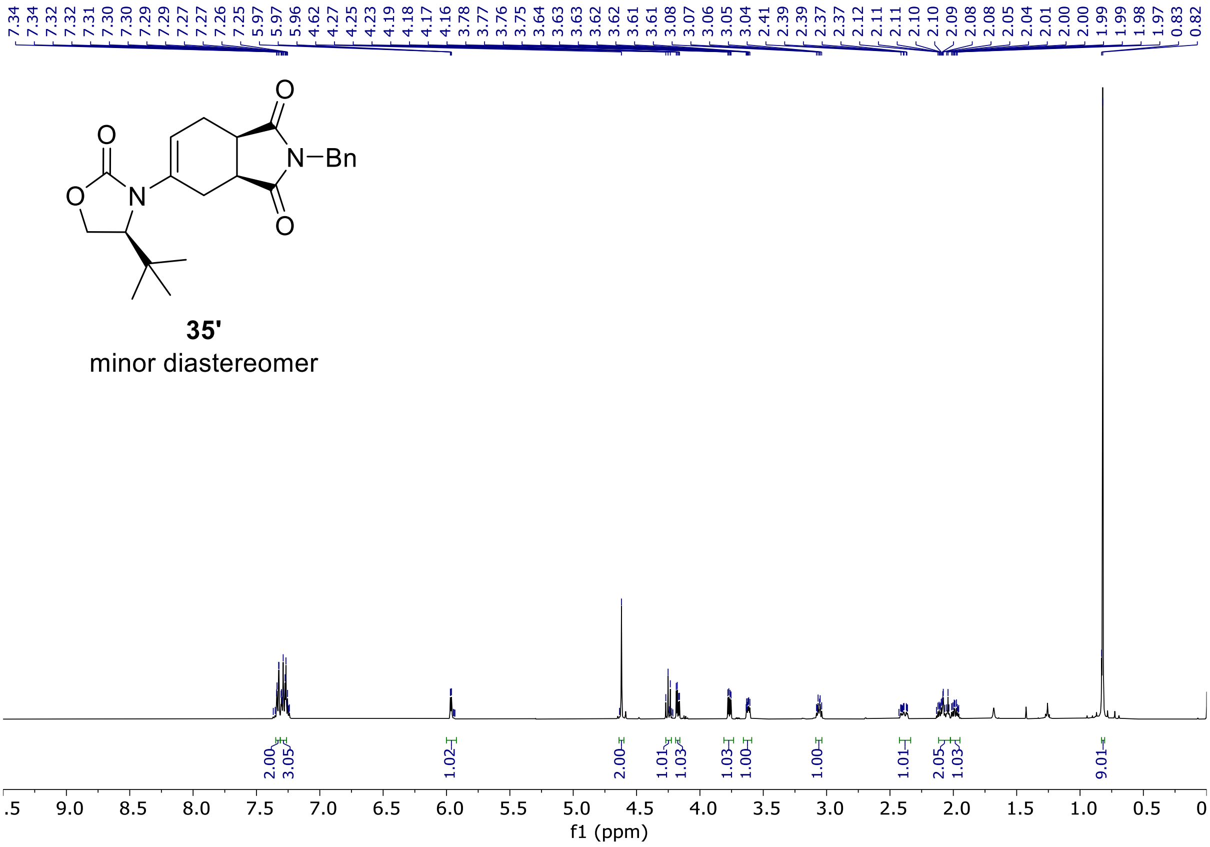

35'
minor diastereomer

^{13}C {^{1}H} NMR (126 MHz, $CDCl_3$)

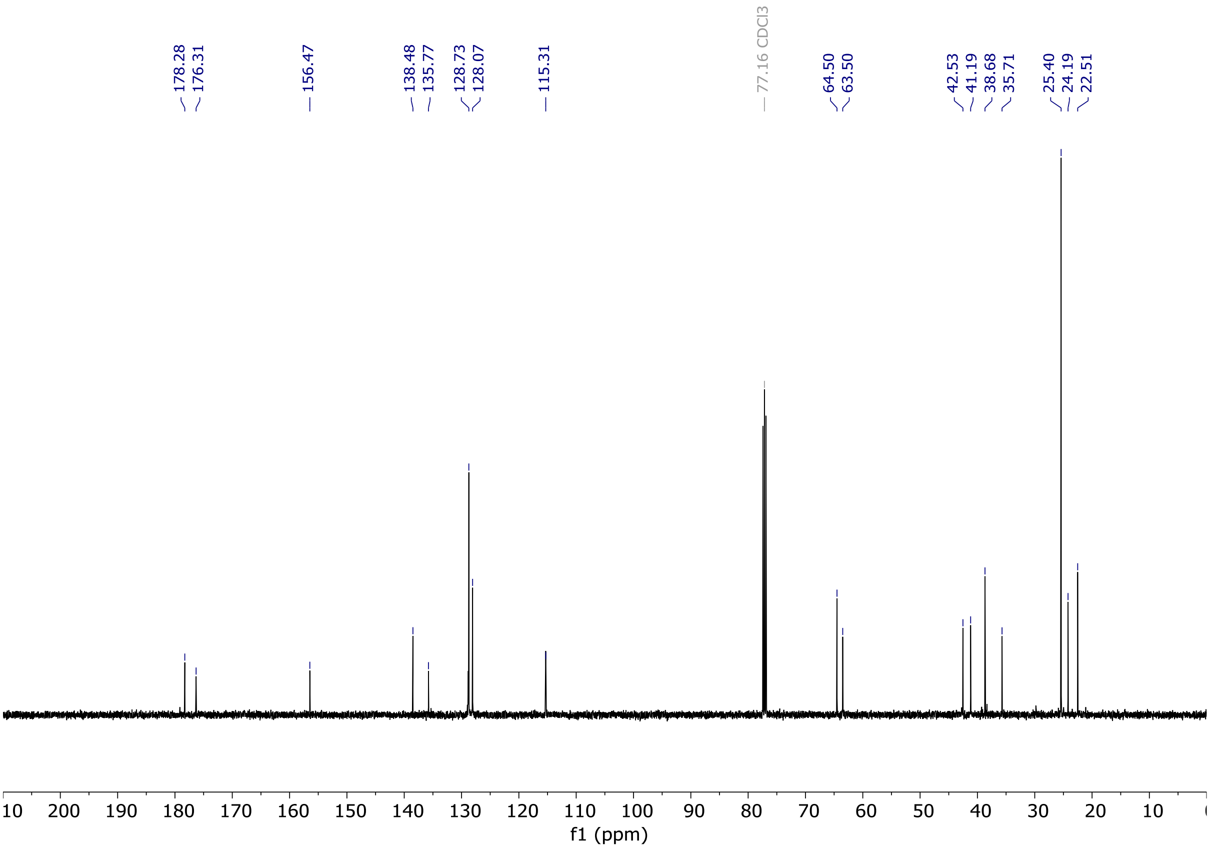

^{1}H NMR (500 MHz, $CDCl_3$)

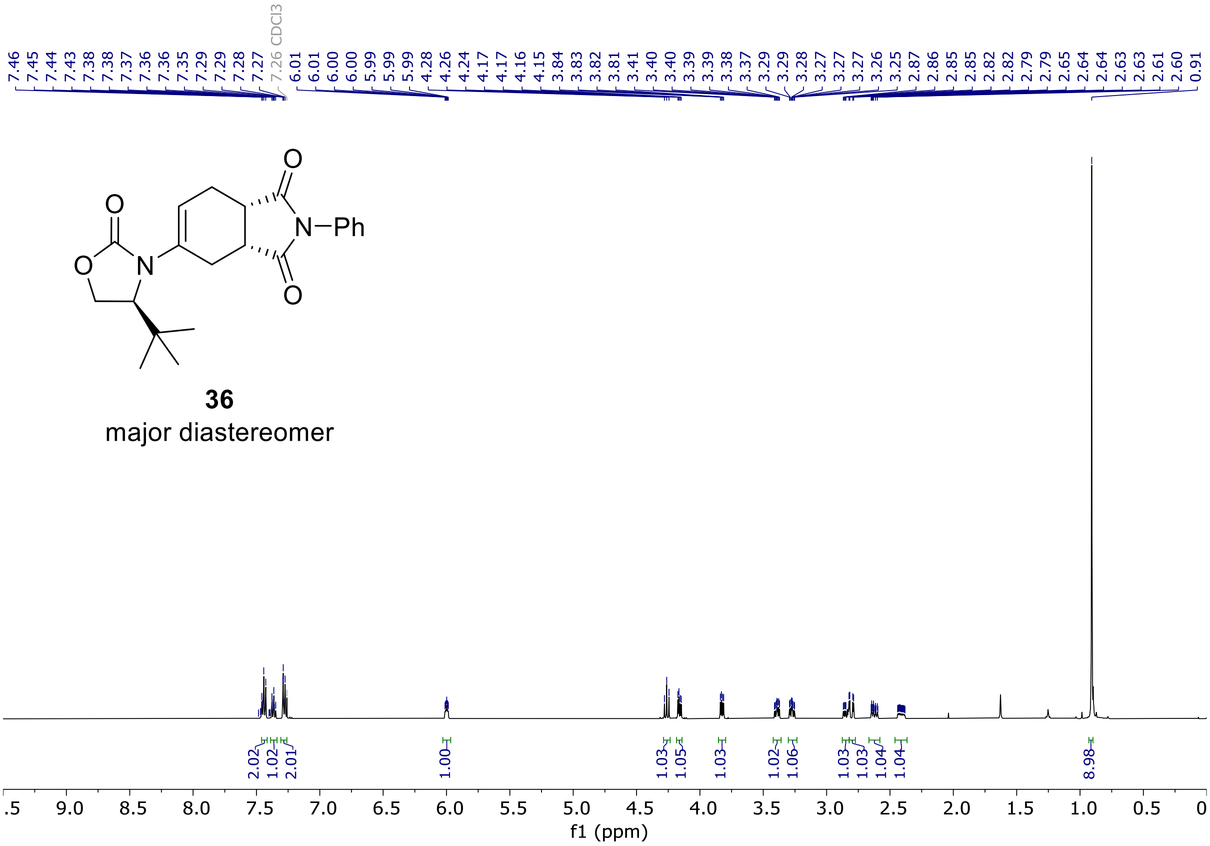

36
major diastereomer

^{13}C {^{1}H} NMR (126 MHz, $CDCl_3$)

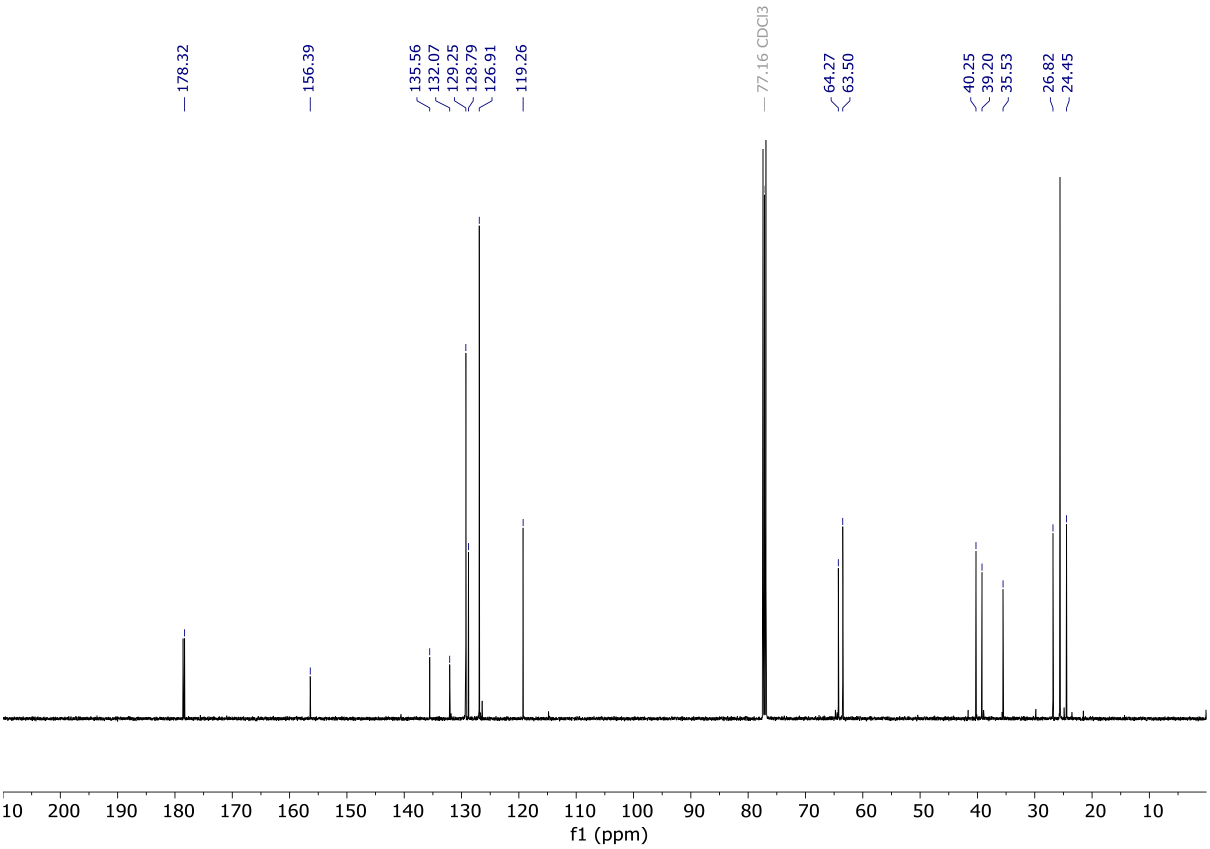

^{1}H NMR (500 MHz, $CDCl_3$)

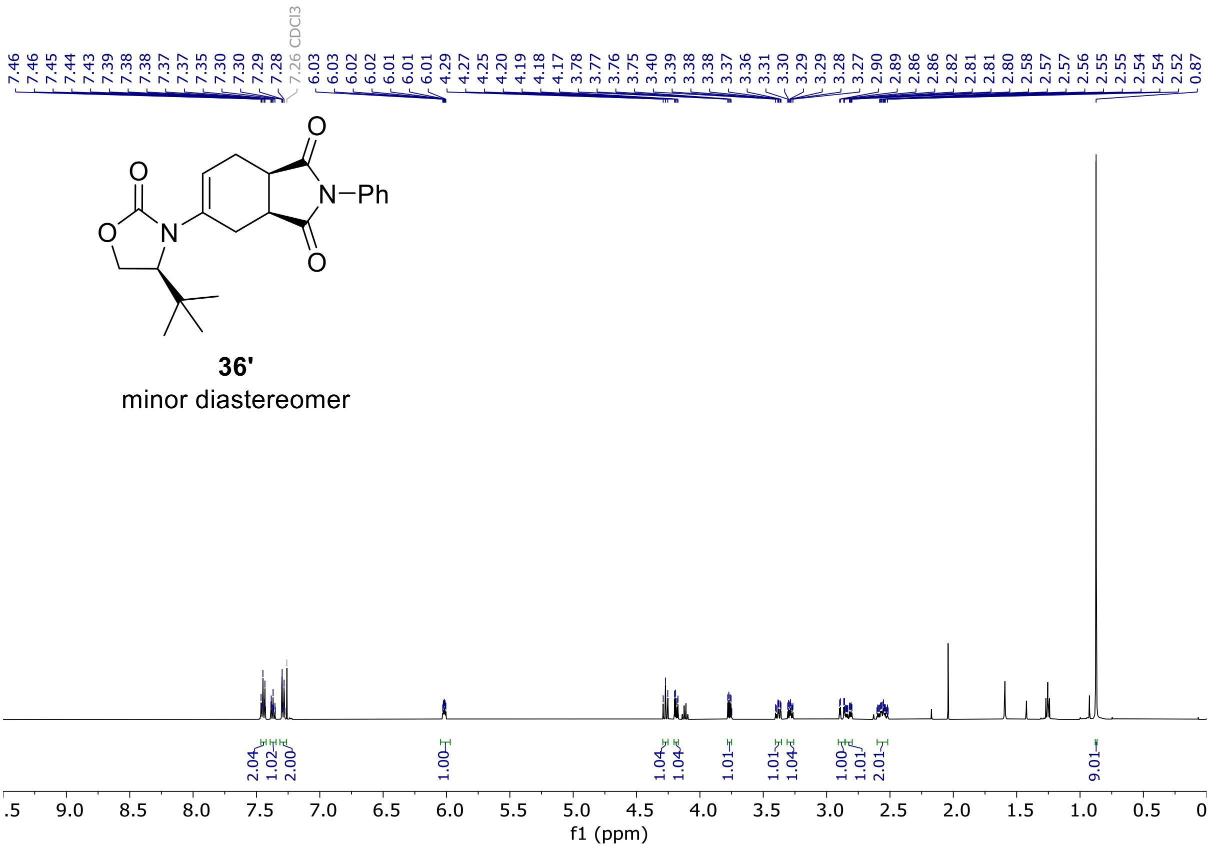

^{13}C {^{1}H} NMR (126 MHz, $CDCl_3$)

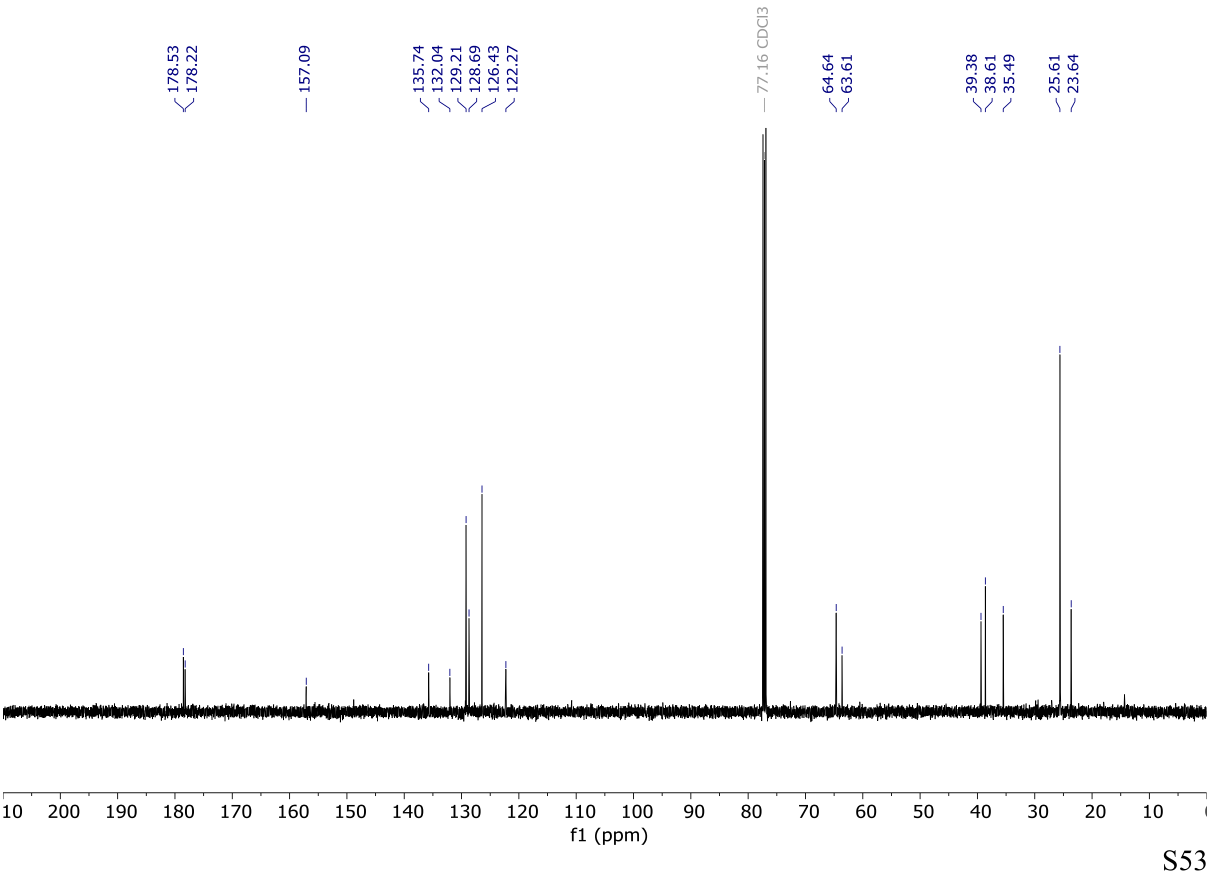

^{1}H NMR (500 MHz, $CDCl_3$)

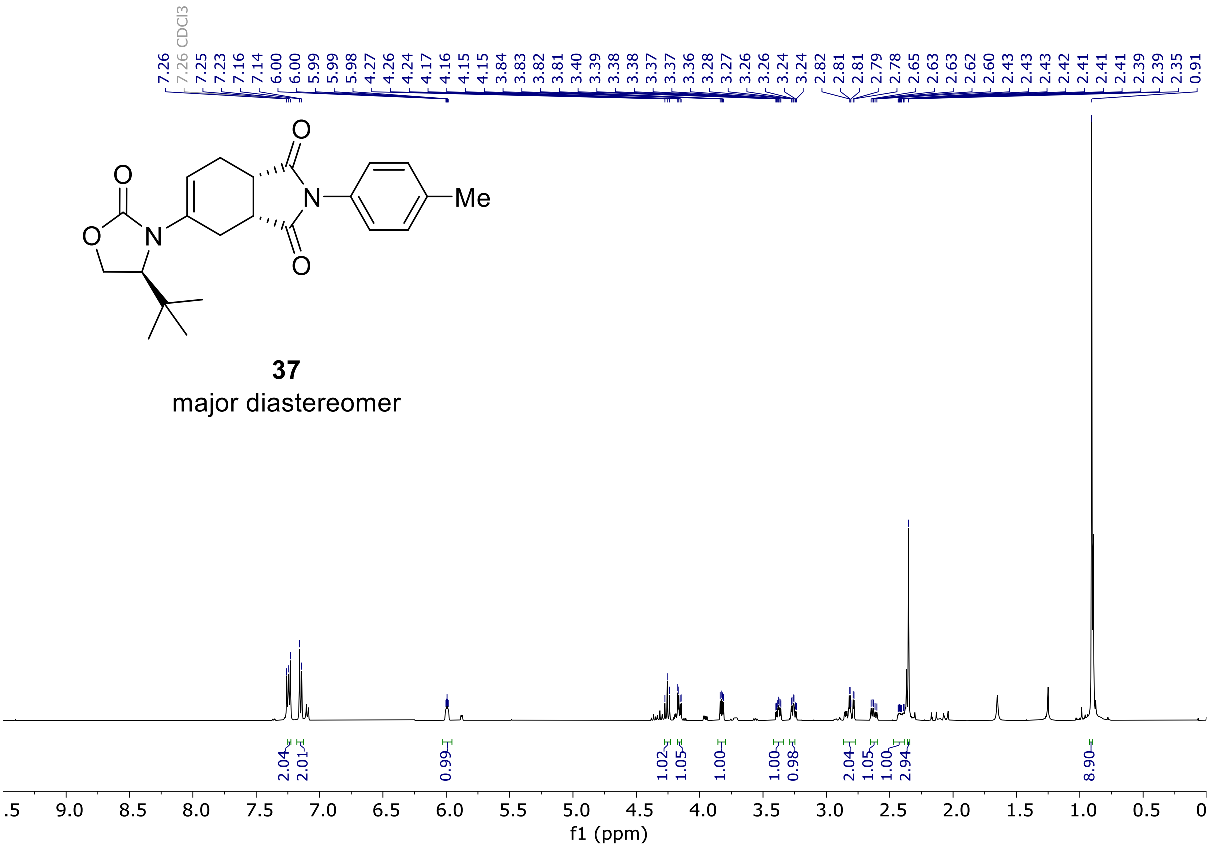

^{13}C {^{1}H} NMR (126 MHz, $CDCl_3$)

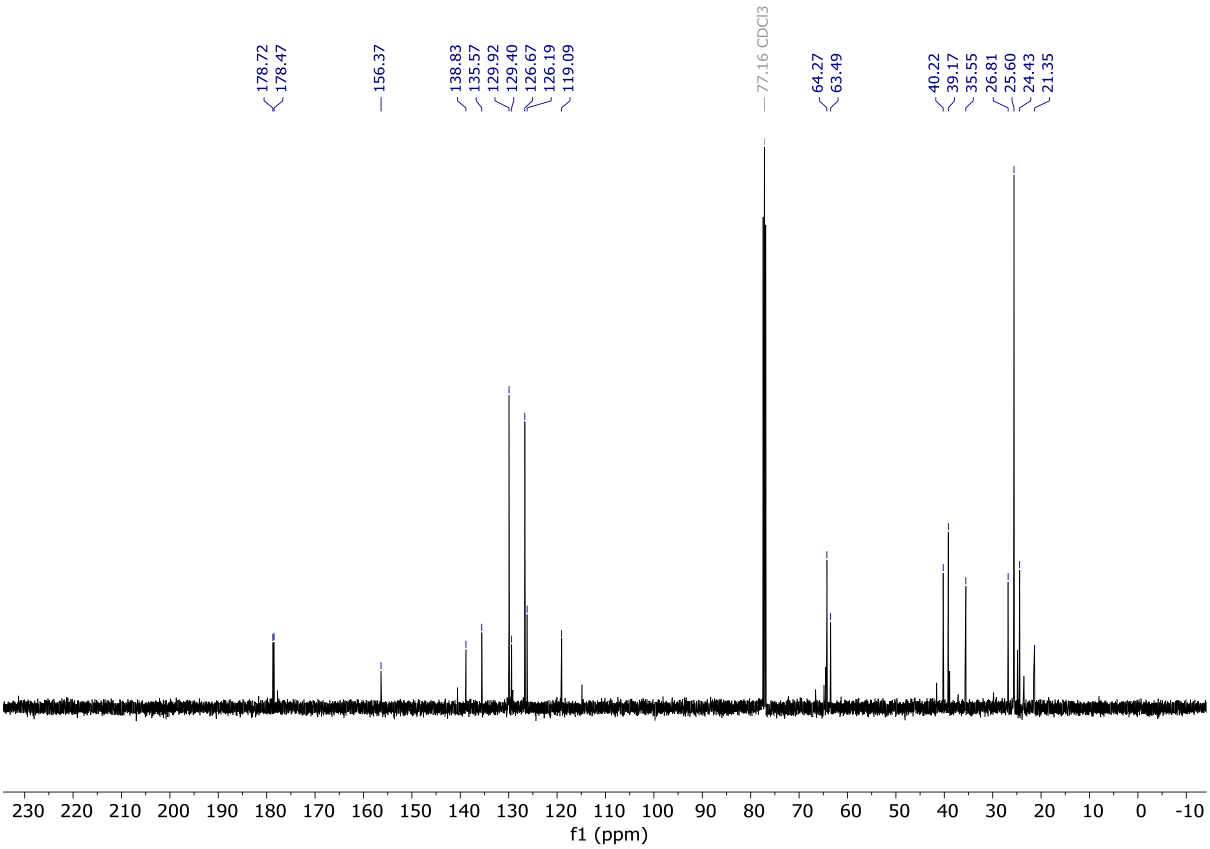

^{1}H NMR (500 MHz, $CDCl_3$)

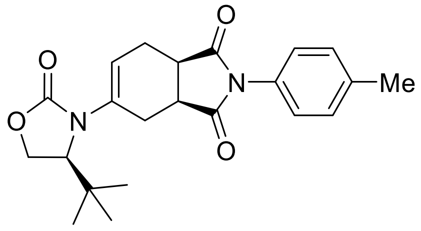

37'
minor diastereomer

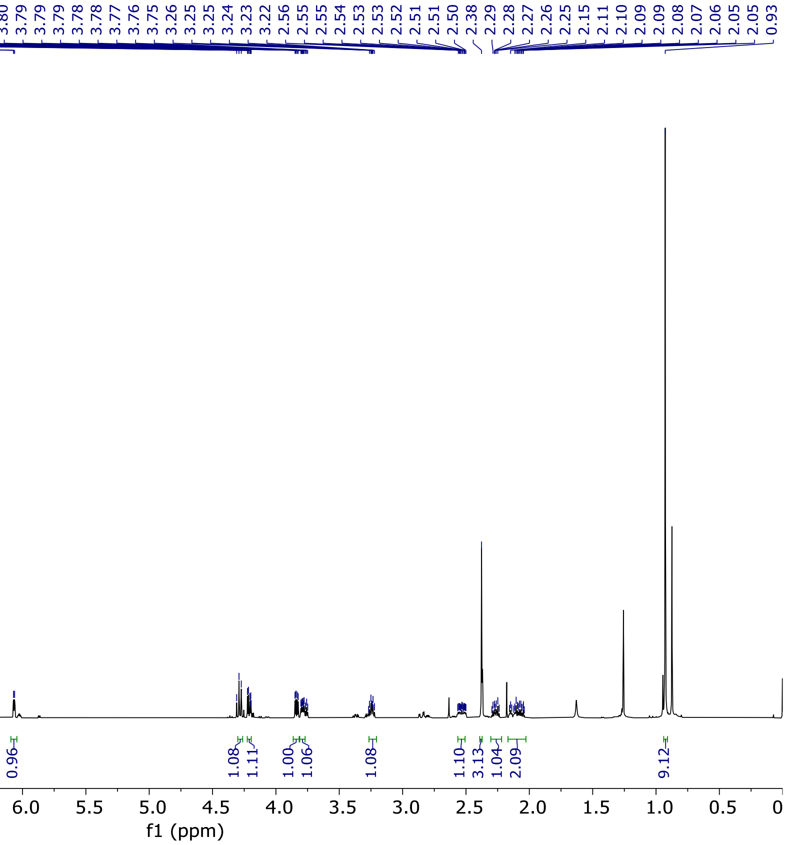

^{13}C {^{1}H} NMR (126 MHz, $CDCl_3$)

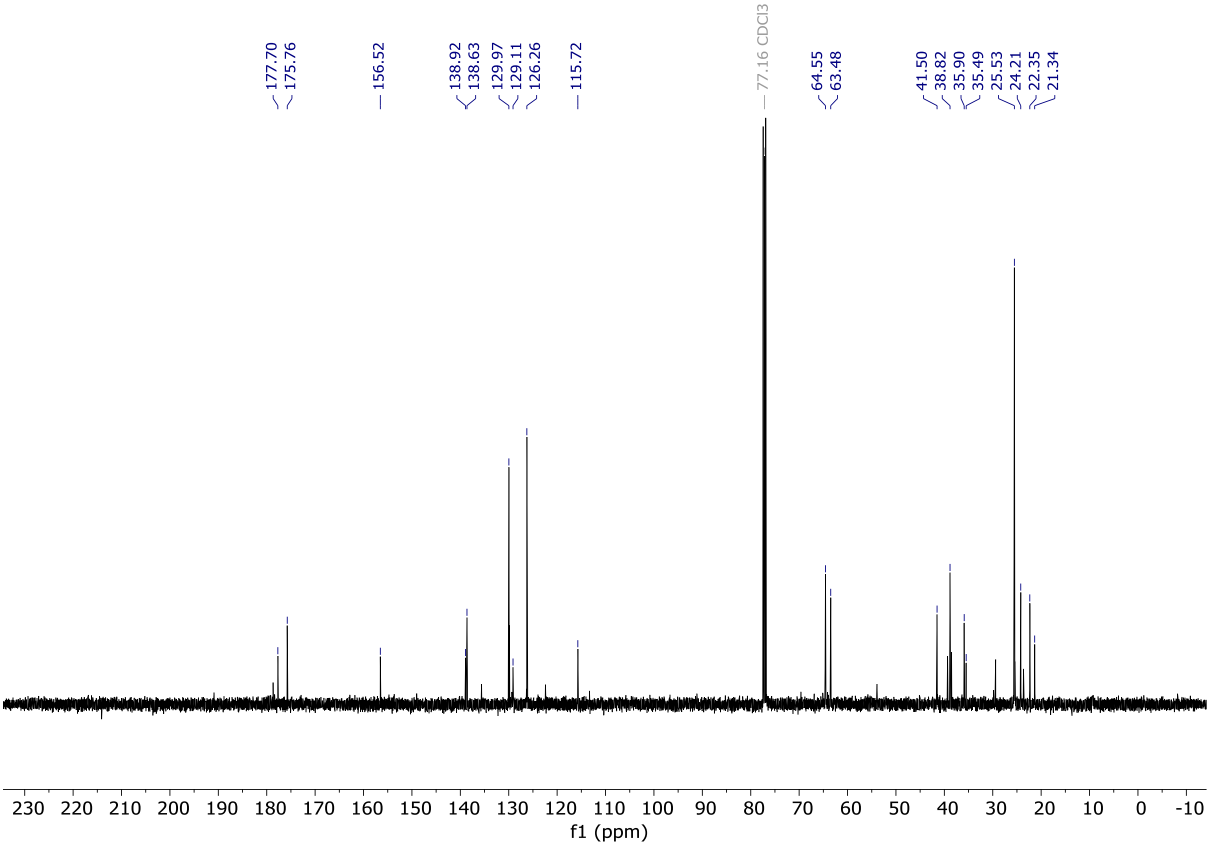

^{1}H NMR (500 MHz, $CDCl_3$)

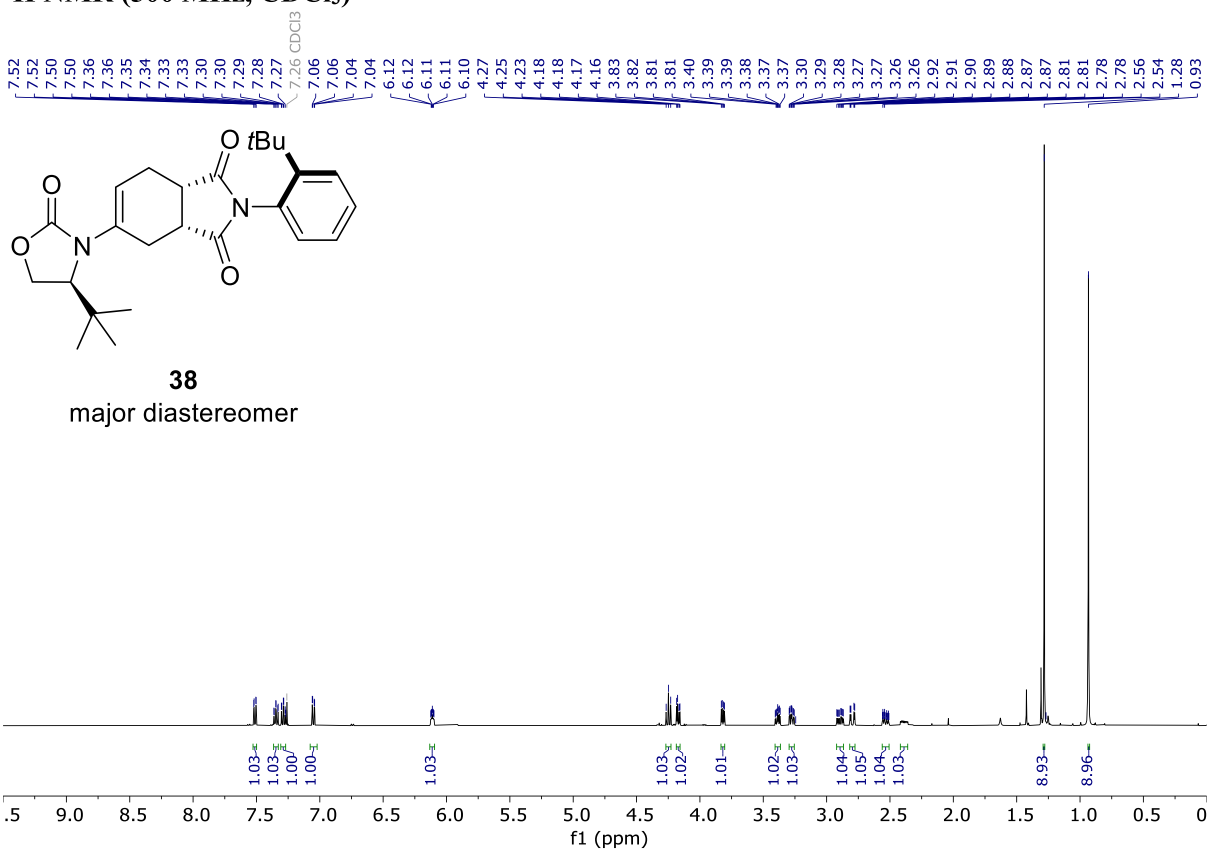

38

major diastereomer

^{13}C {^{1}H} NMR (126 MHz, $CDCl_3$)

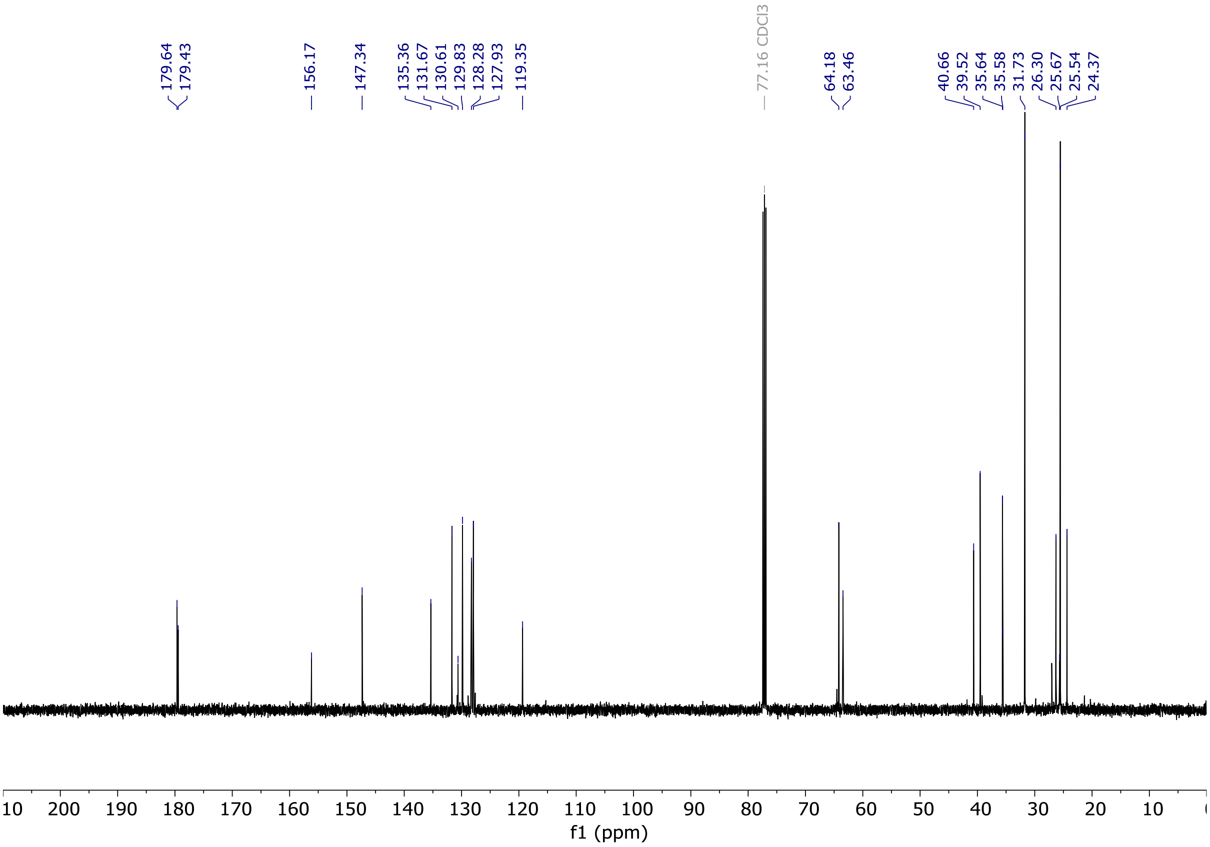

^{1}H NMR (400 MHz, $CDCl_3$)

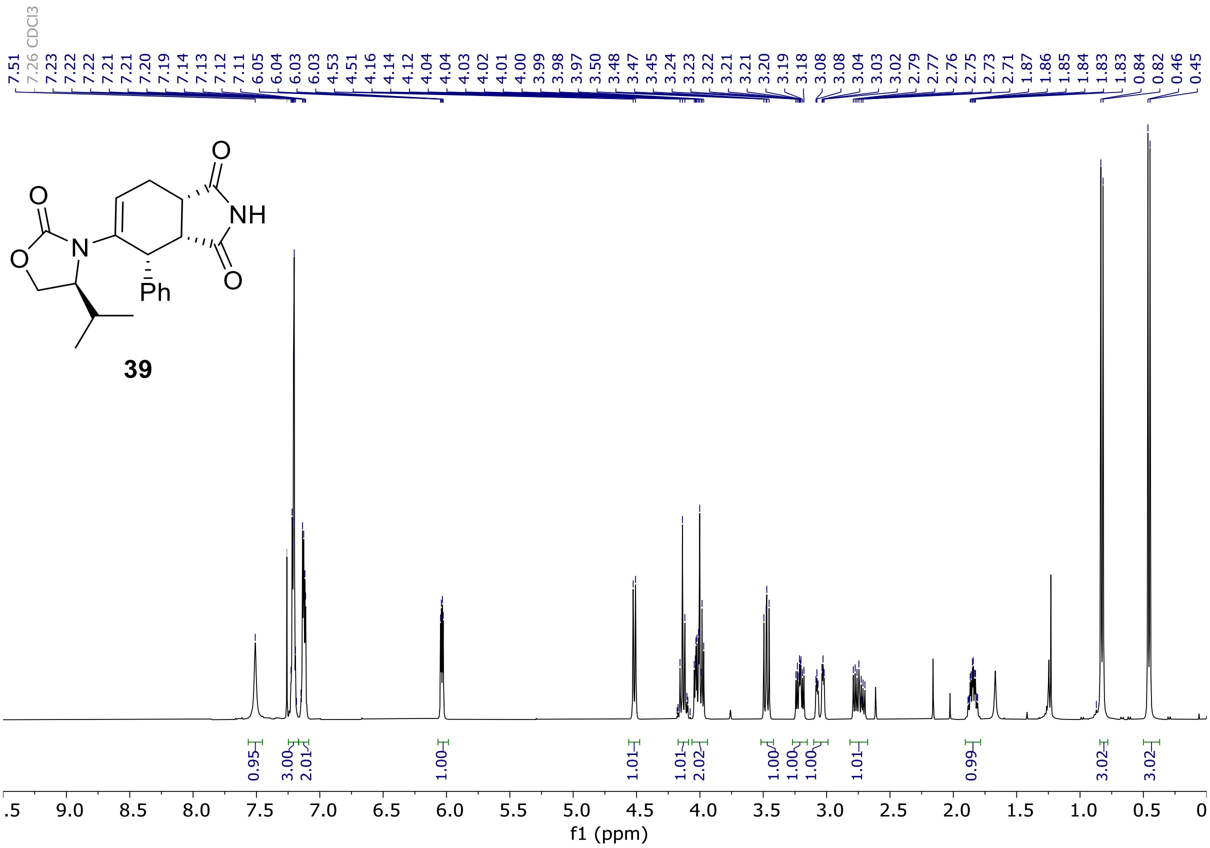

^{13}C {^{1}H} NMR (101 MHz, $CDCl_3$)

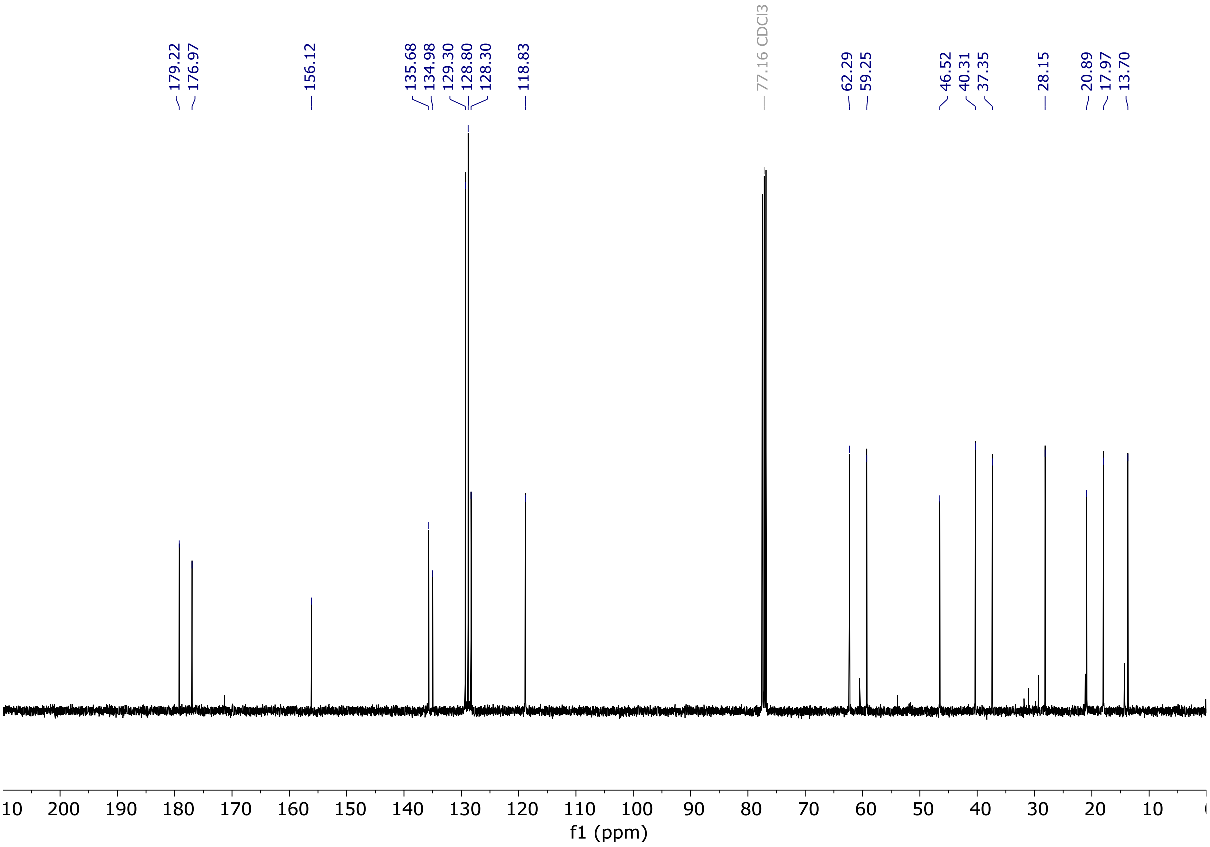

^{1}H NMR (400 MHz, $CDCl_3$)

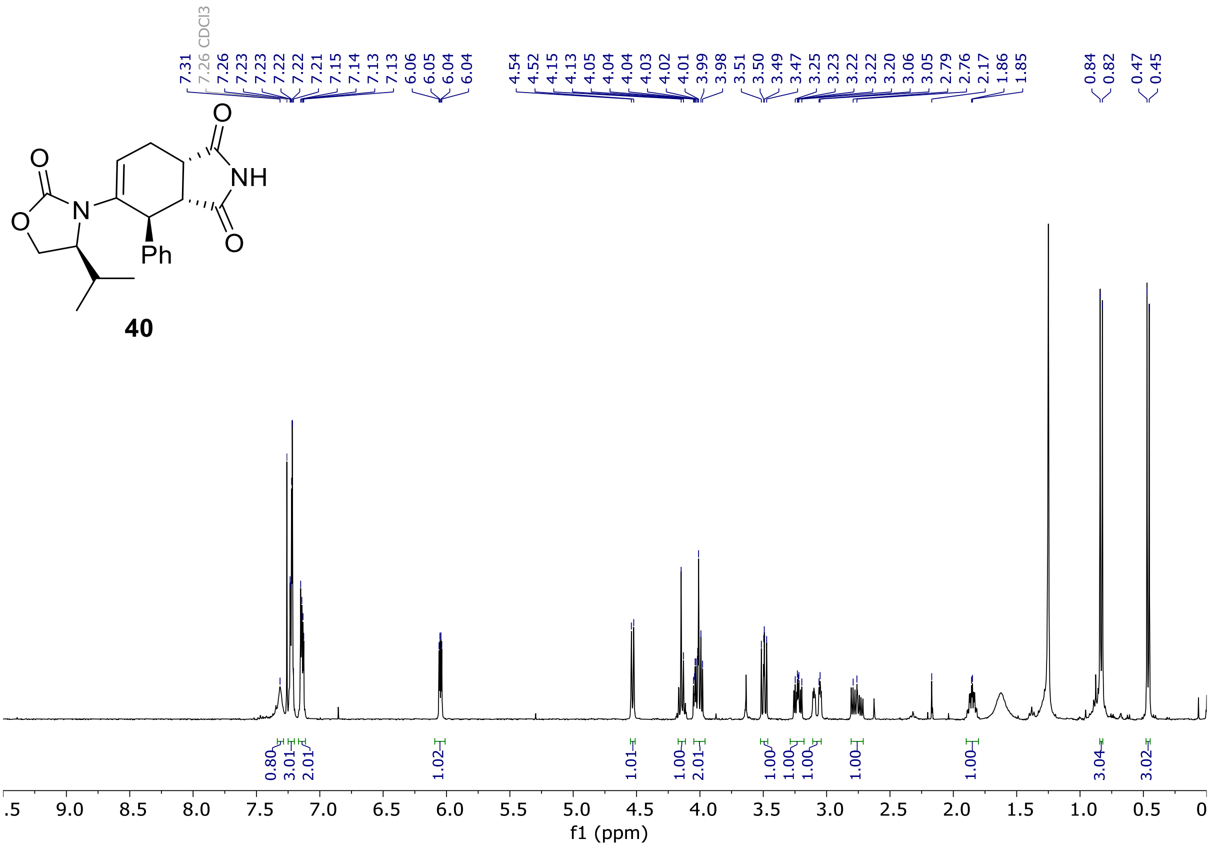

^{13}C {^{1}H} NMR (101 MHz, $CDCl_3$)

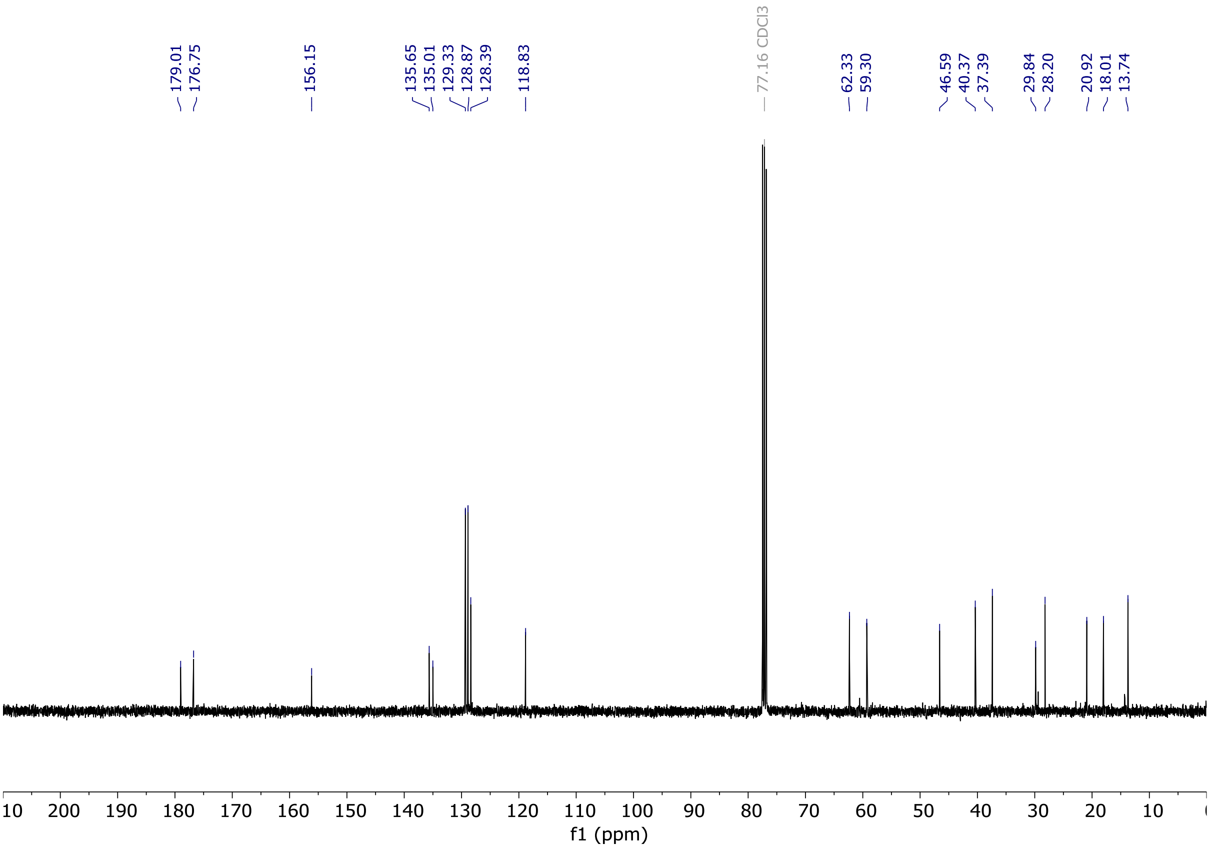

^{1}H NMR (400 MHz, $CDCl_3$)

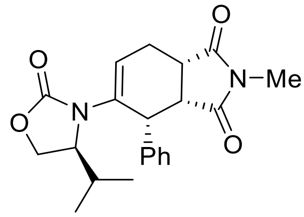

41

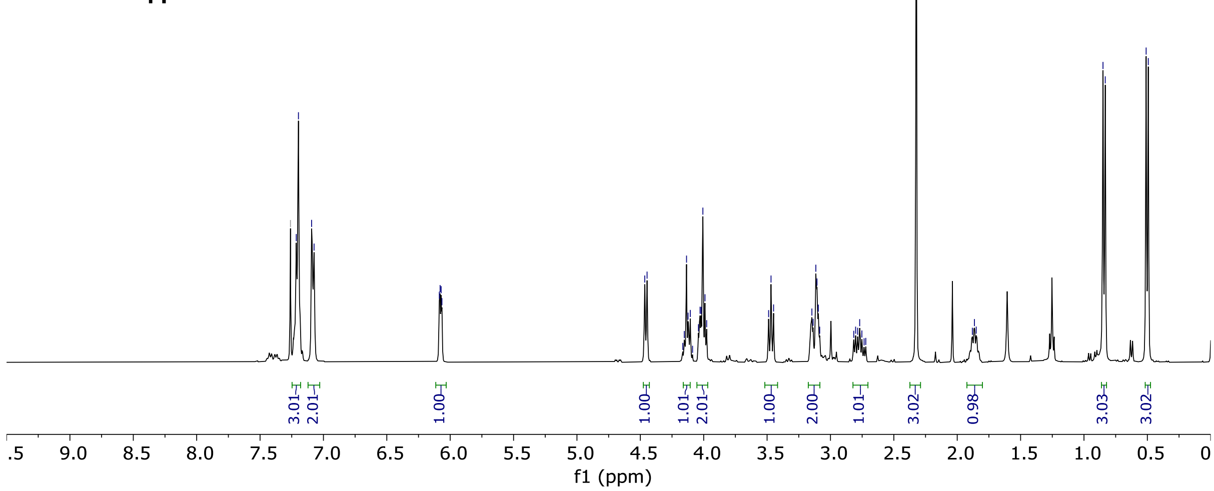

^{13}C {^{1}H} NMR (126 MHz, $CDCl_3$)

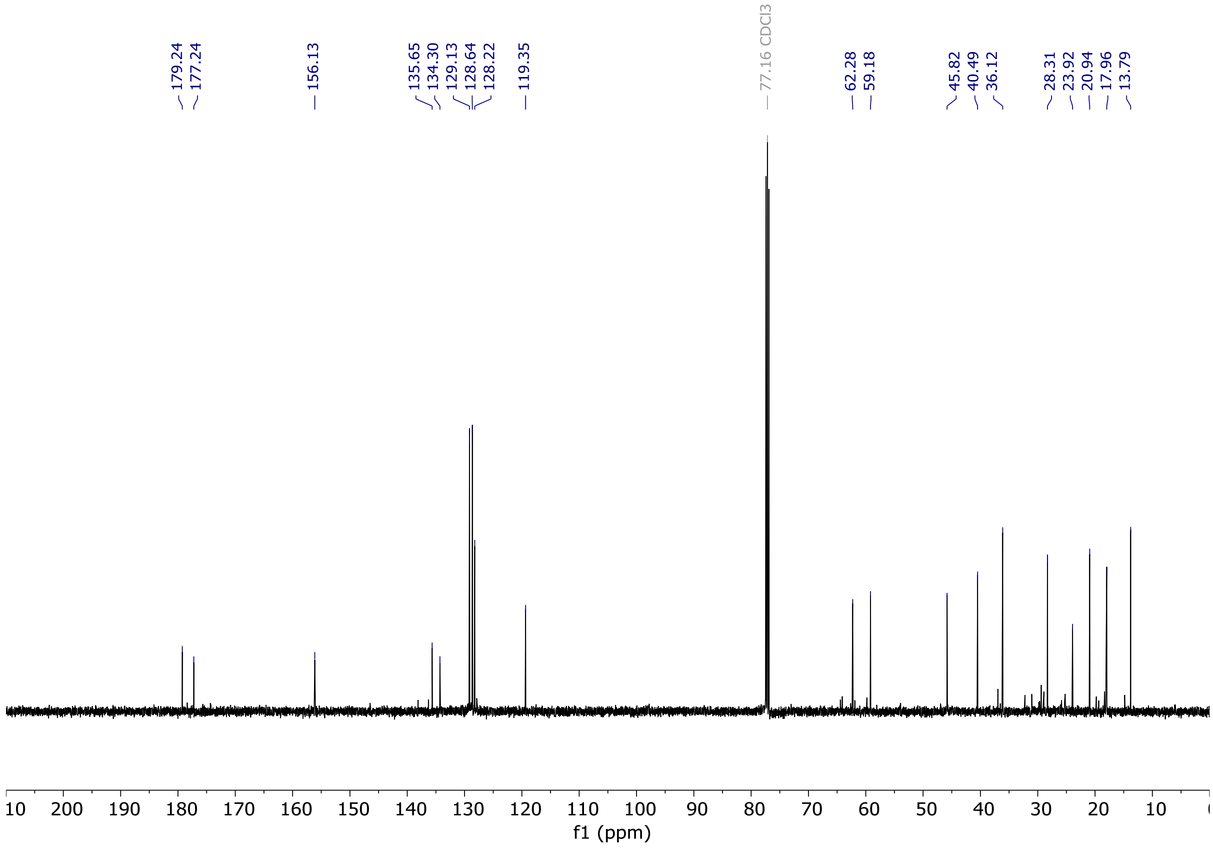

^1H NMR (400 MHz, $CDCl_3$)

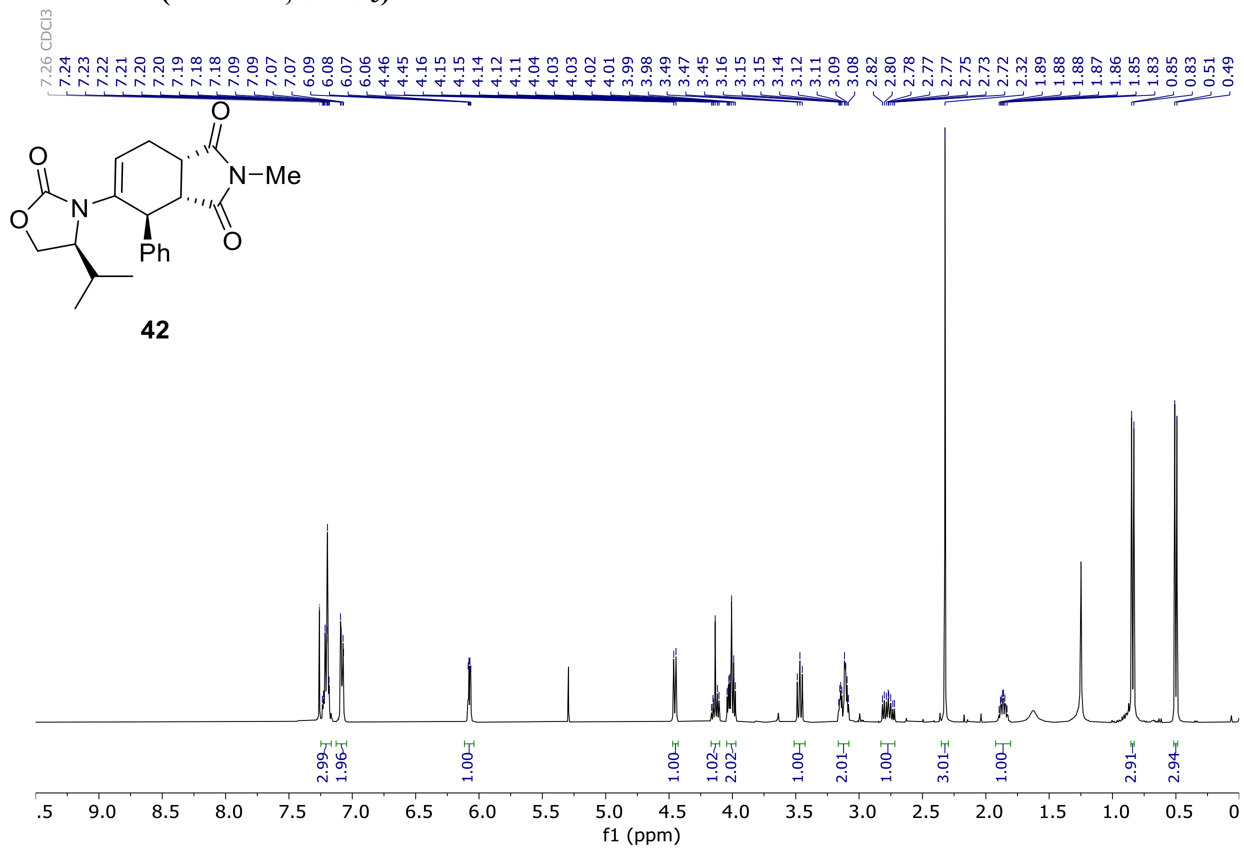

^{13}C {^1H} NMR (101 MHz, $CDCl_3$)

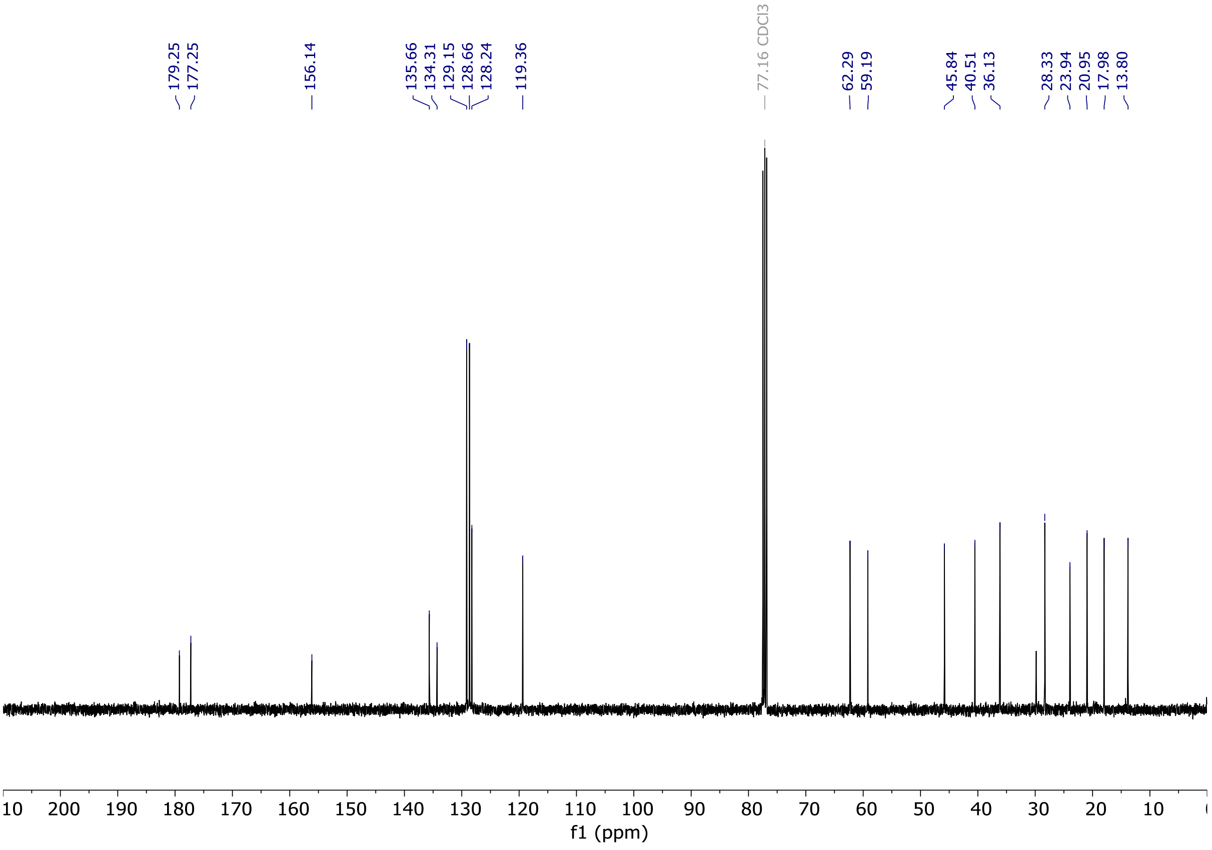

^{1}H NMR (400 MHz, $CDCl_3$)

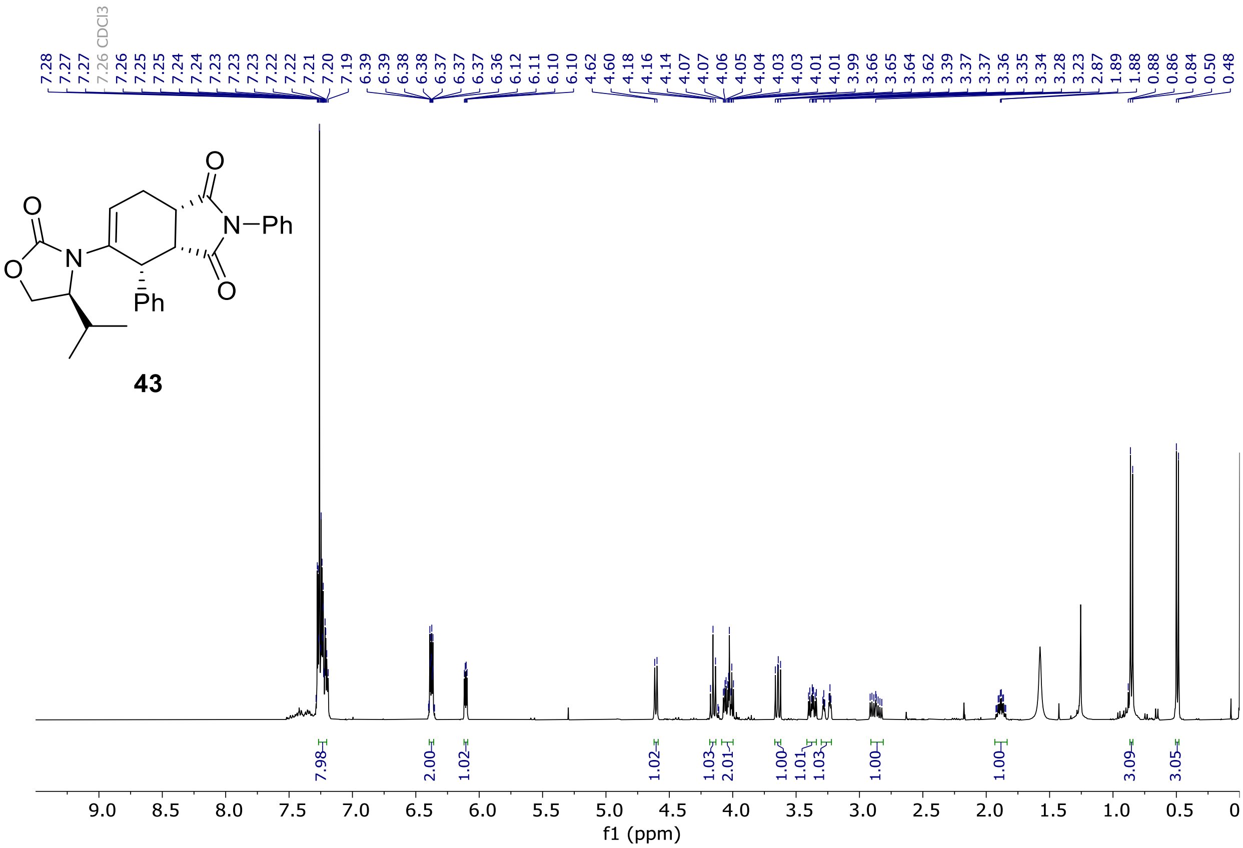

^{13}C {^{1}H} NMR (126 MHz, $CDCl_3$)

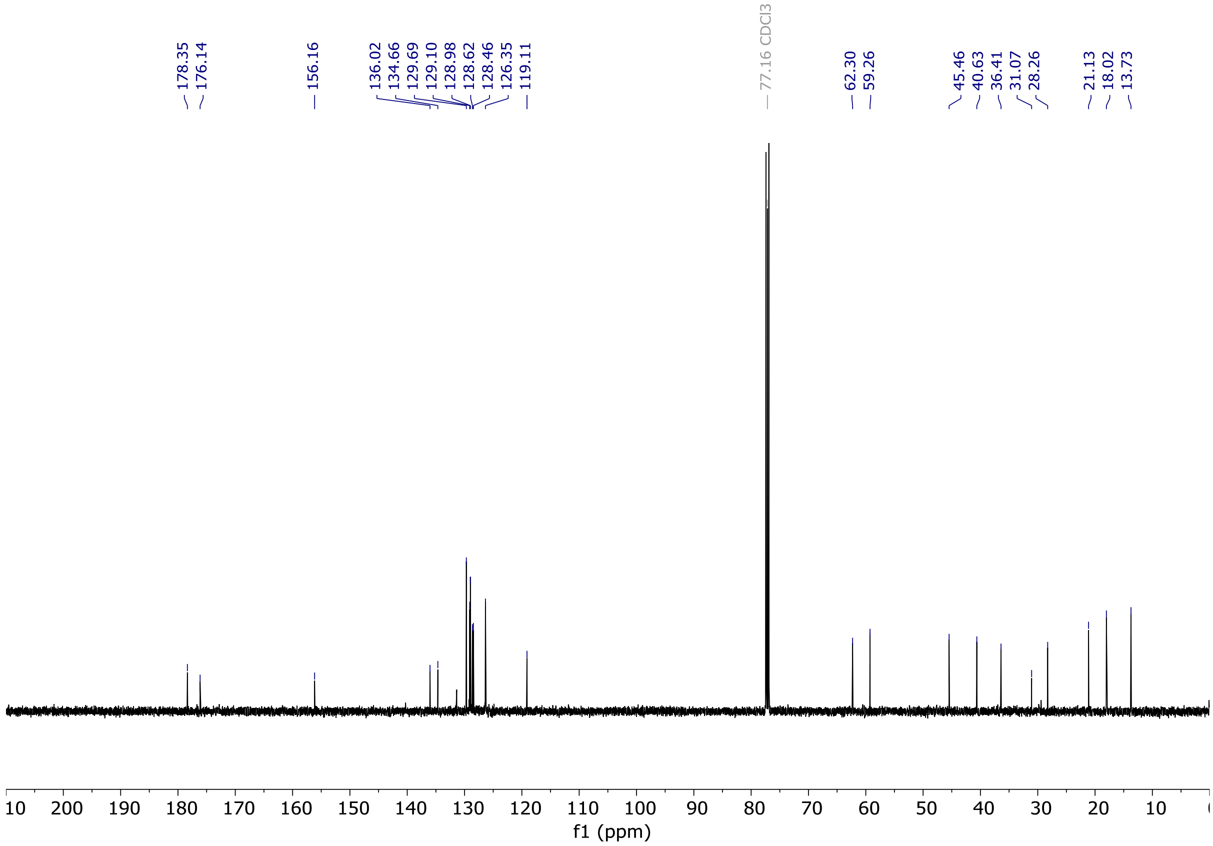

^{1}H NMR (400 MHz, $CDCl_3$)

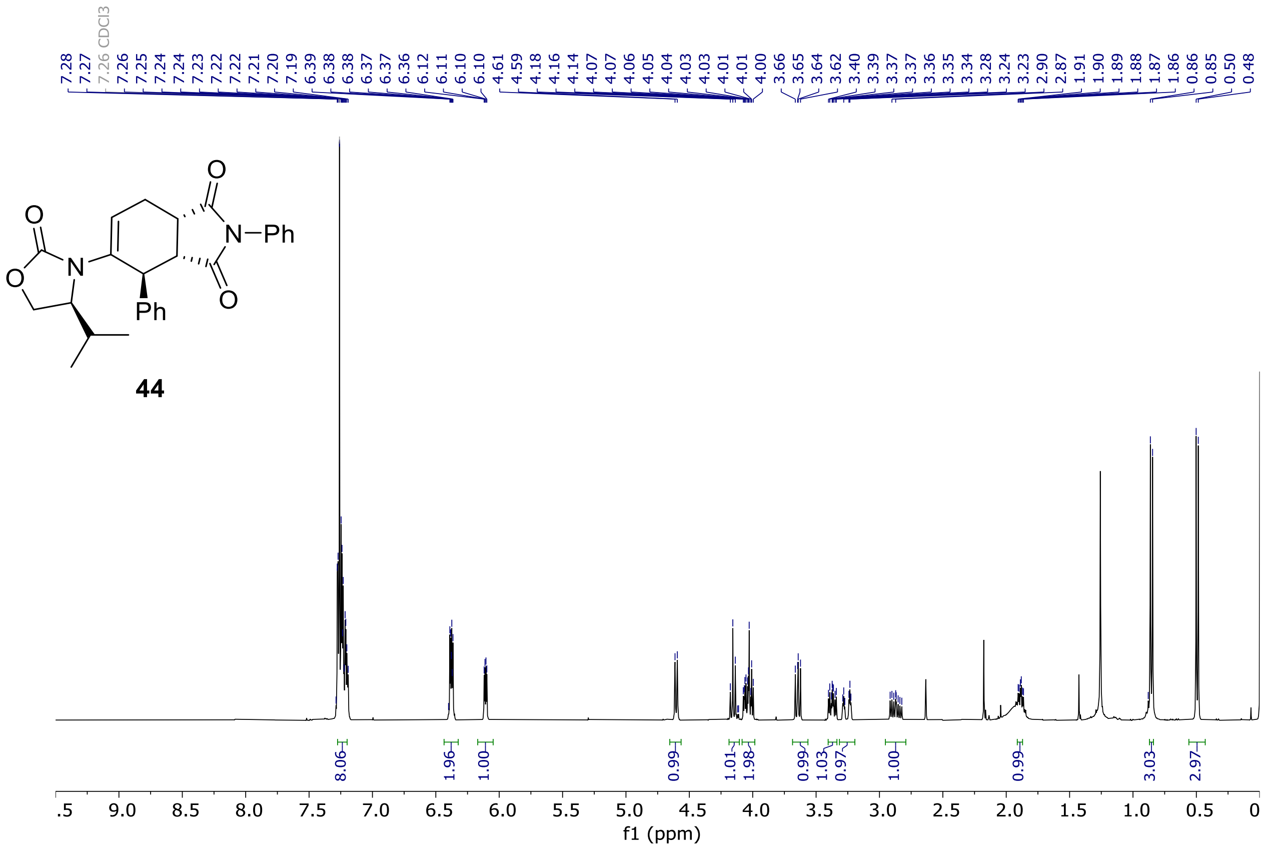

^{13}C {^{1}H} NMR (101 MHz, $CDCl_3$)

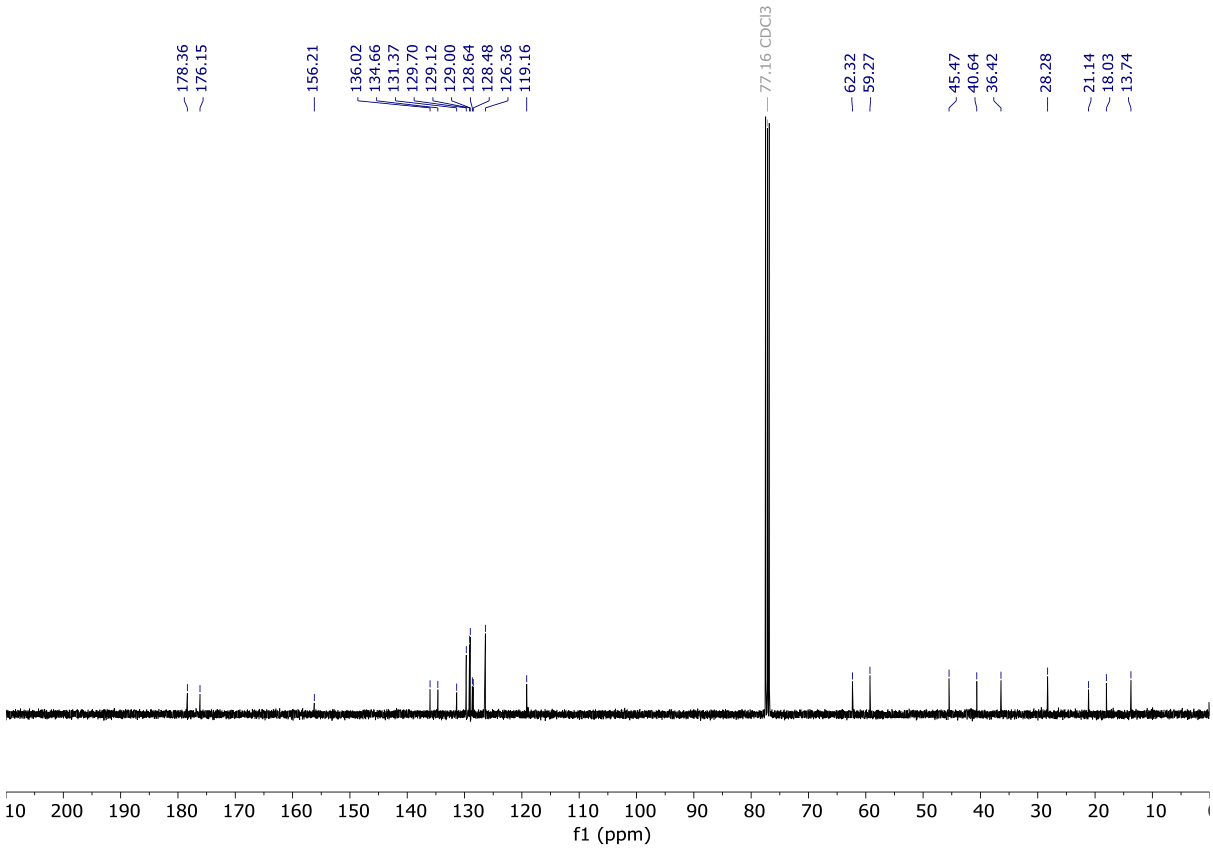

^{1}H NMR (500 MHz, $CDCl_3$)

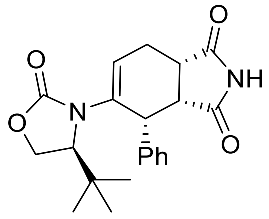

45
major diastereomer

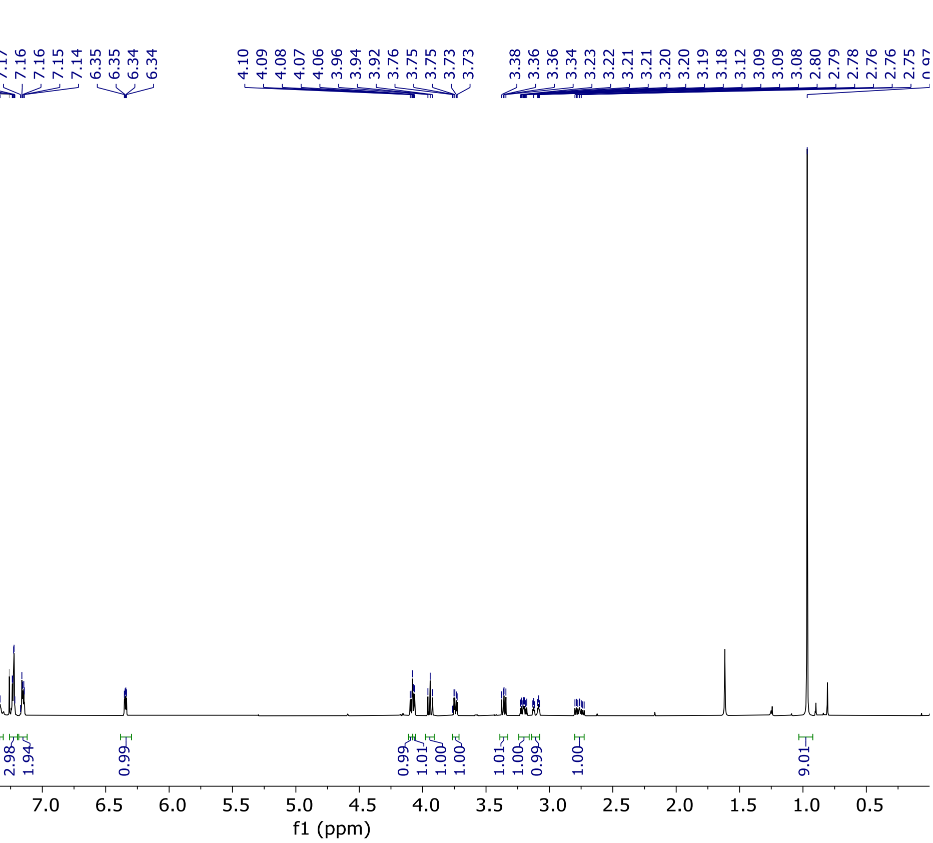

^{13}C {^{1}H} NMR (126 MHz, $CDCl_3$)

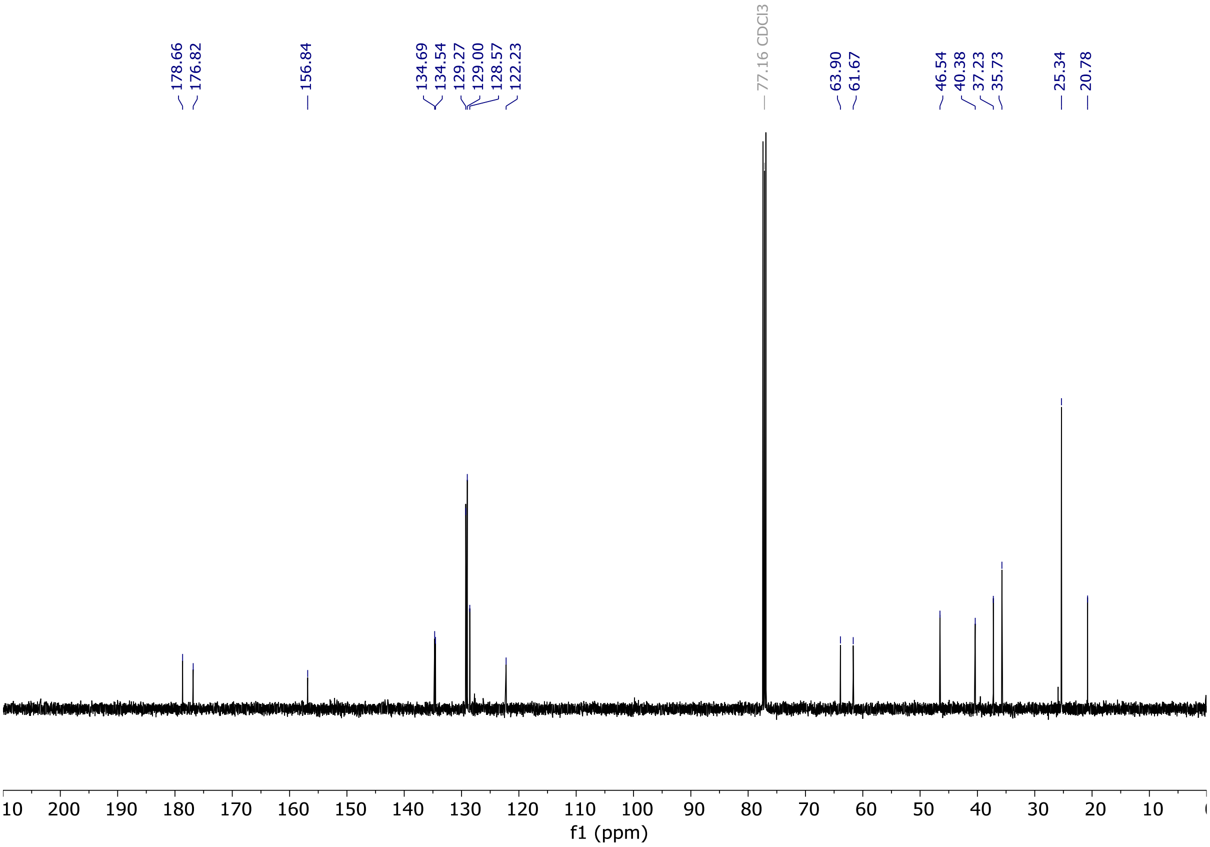

^{1}H NMR (500 MHz, $CDCl_3$)

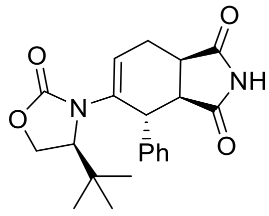

45'
minor diastereomer

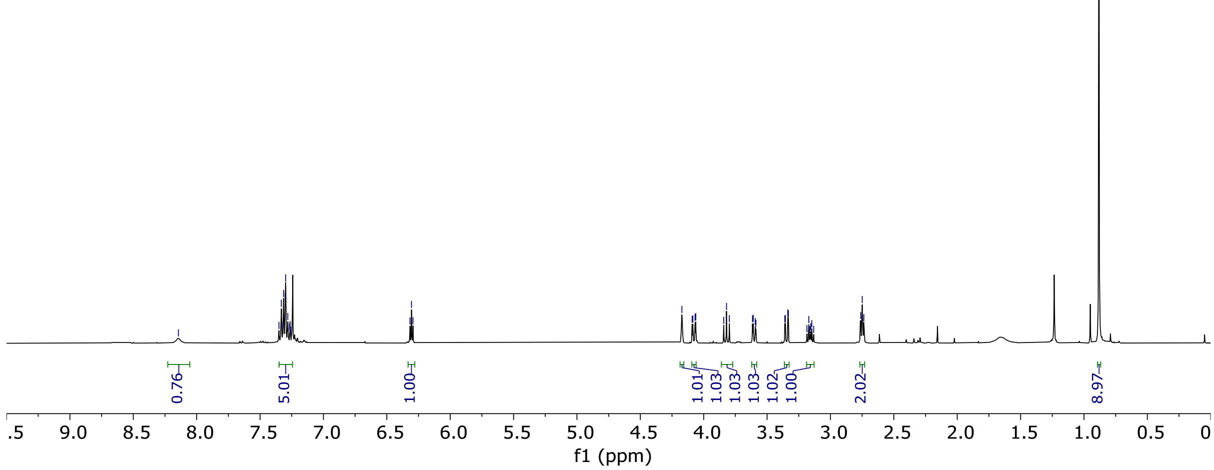

^{13}C {^{1}H} NMR (101 MHz, $CDCl_3$)

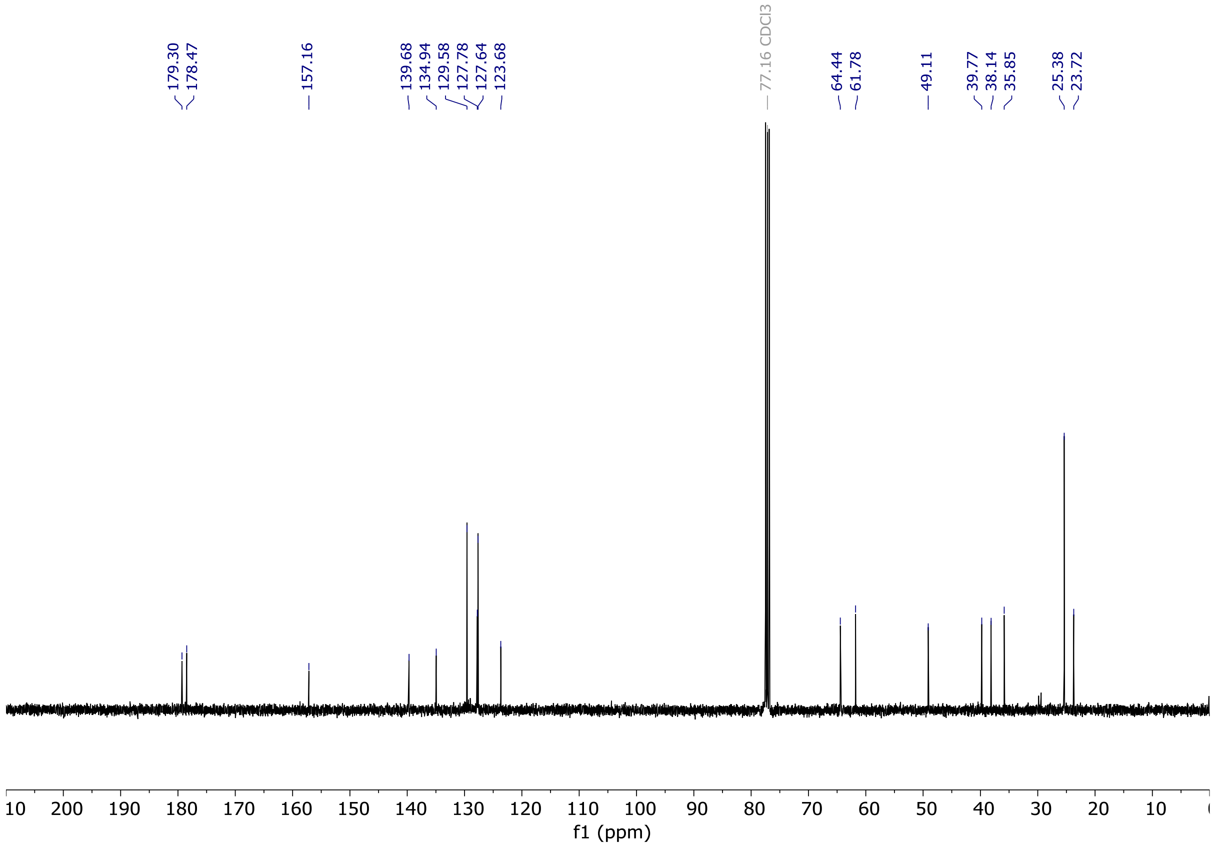

^{1}H NMR (500 MHz, *d*-DMSO)

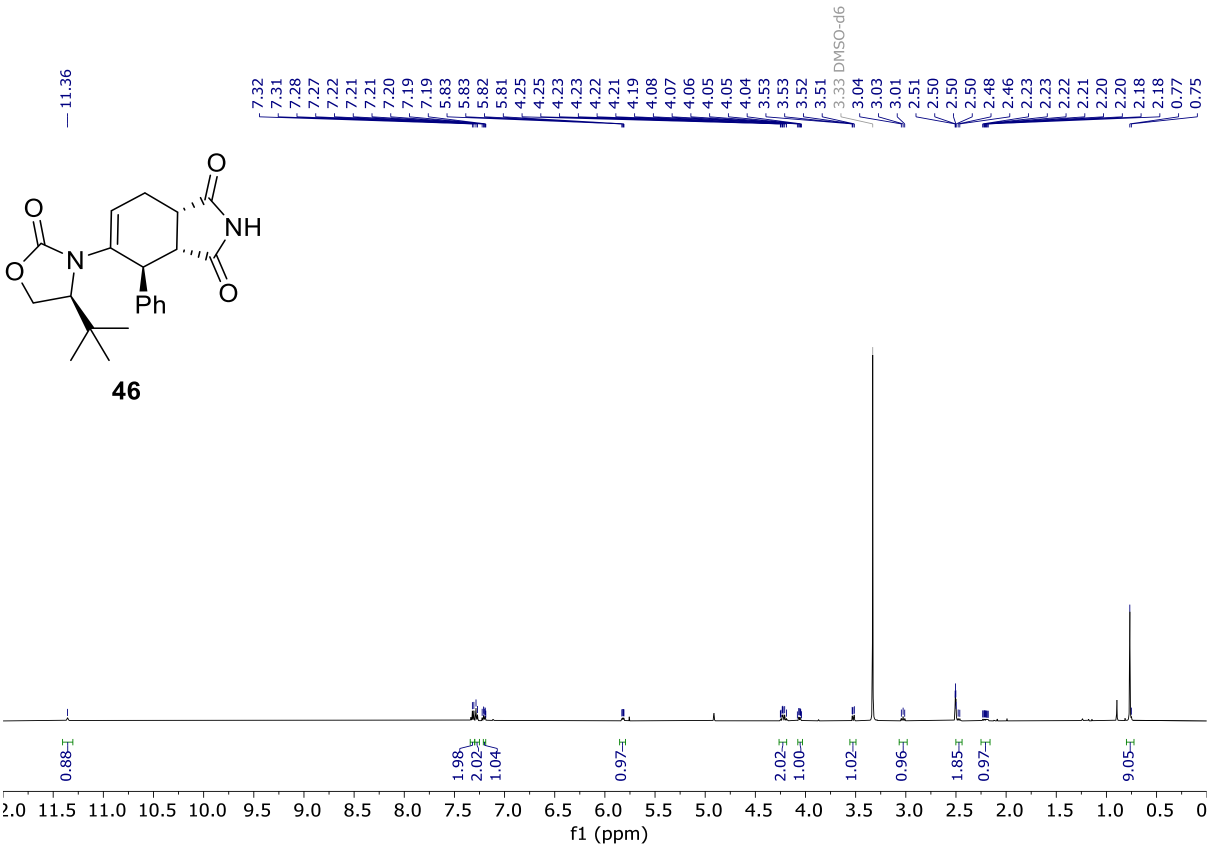

^{13}C {^{1}H} NMR (126 MHz, *d*-DMSO)

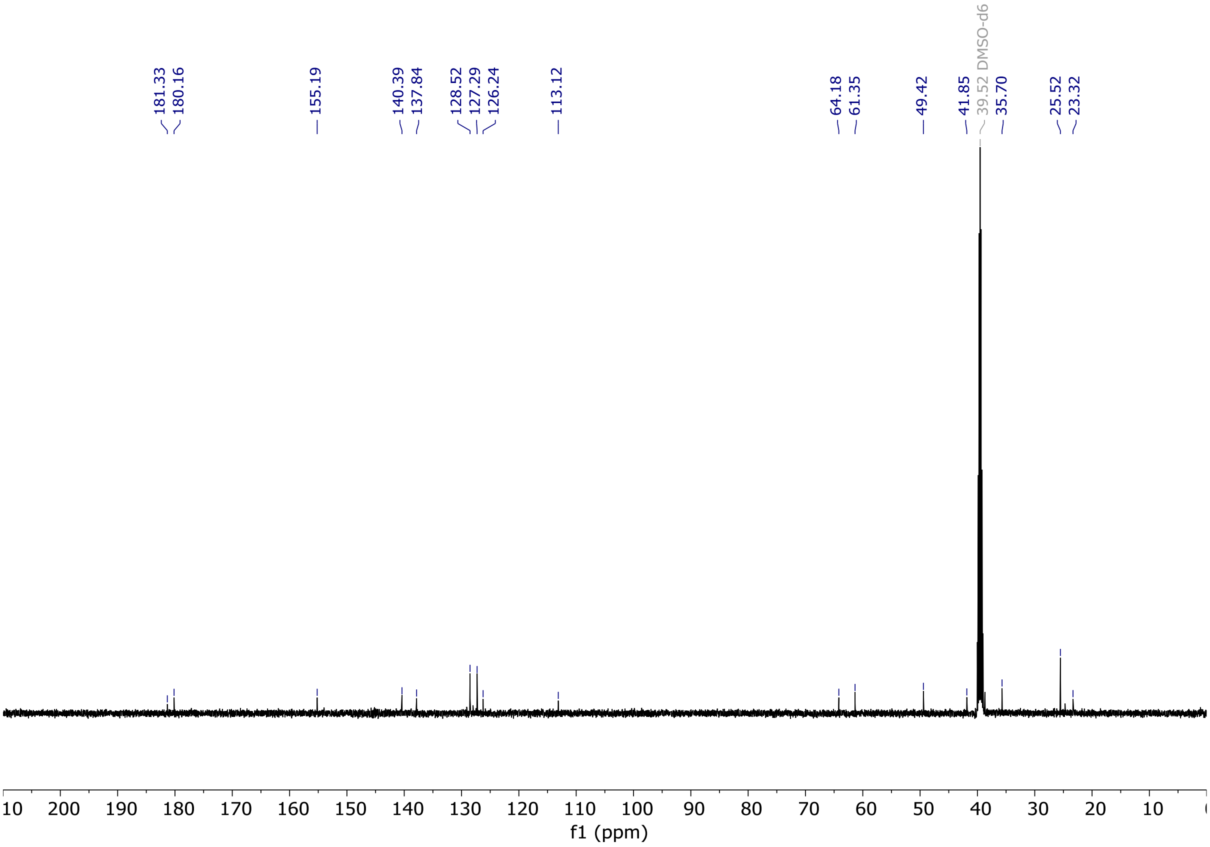

^{1}H NMR (400 MHz, $CDCl_3$)

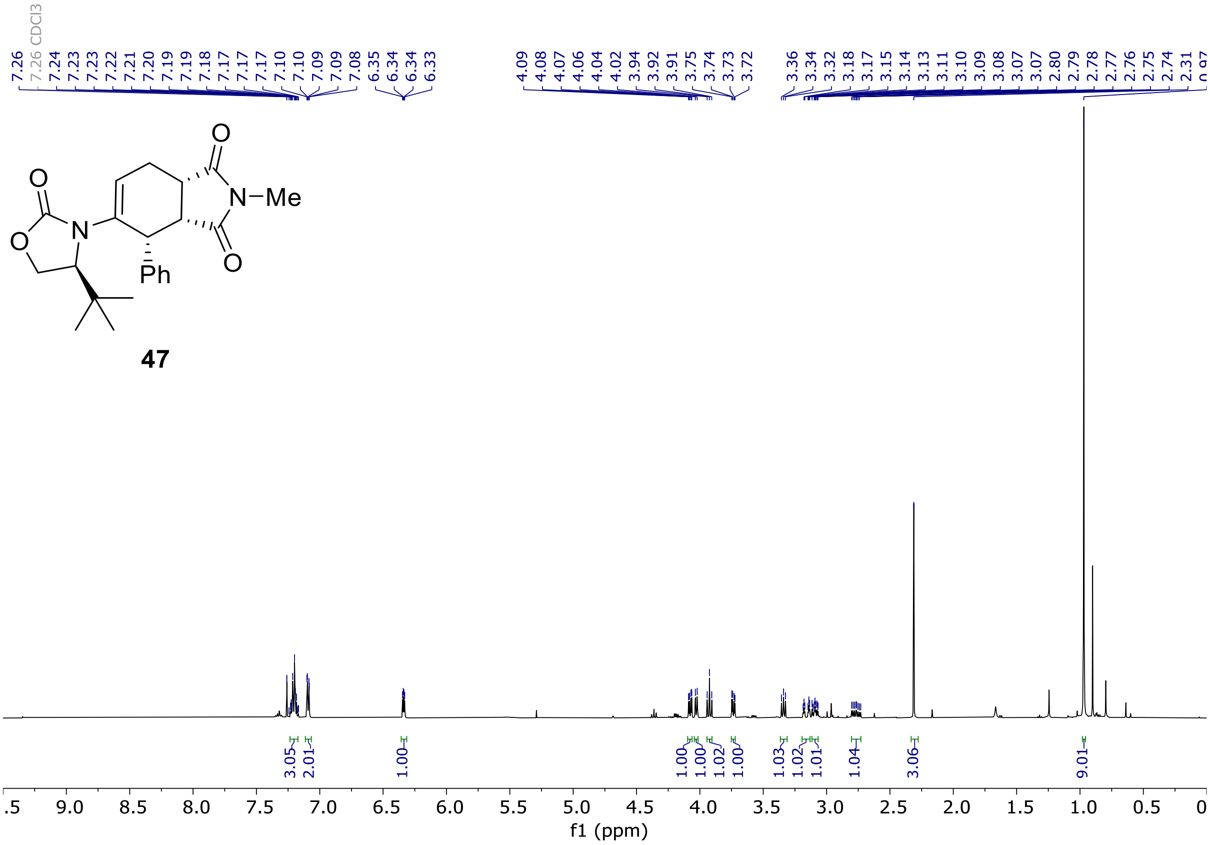

47

^{13}C {^{1}H} NMR (126 MHz, $CDCl_3$)

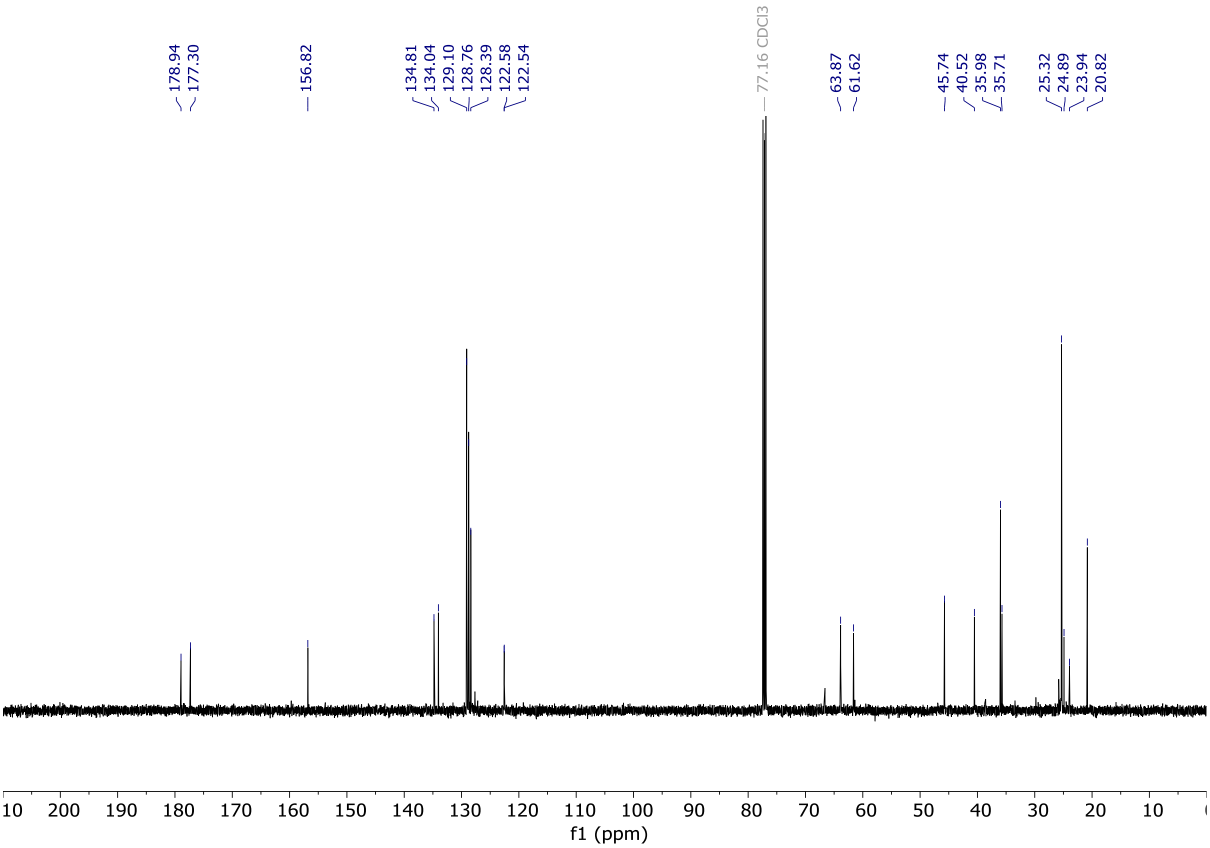

^{1}H NMR (500 MHz, $CDCl_3$)

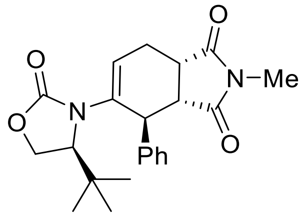

48

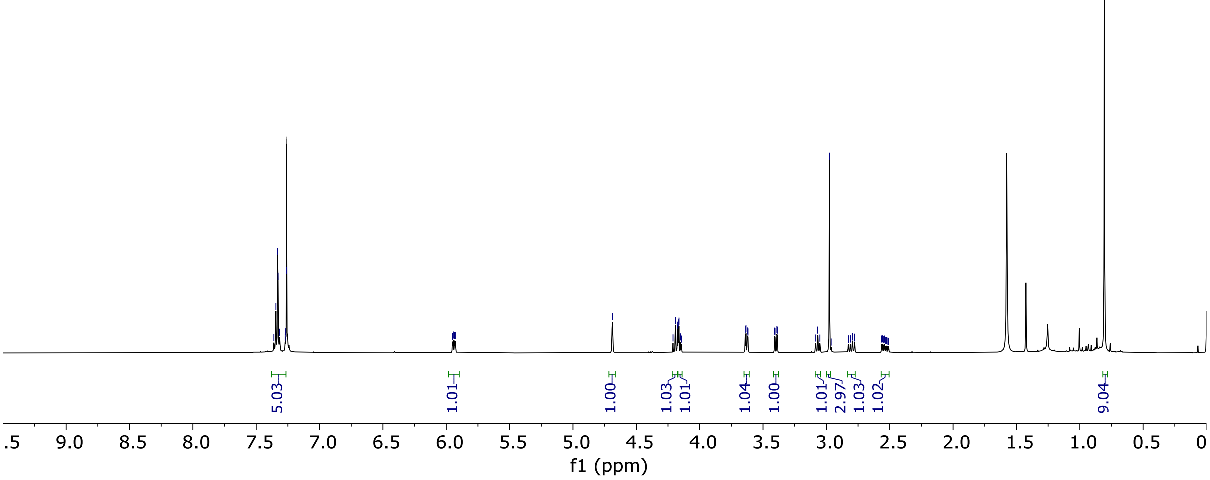

^{13}C {^{1}H} NMR (126 MHz, $CDCl_3$)

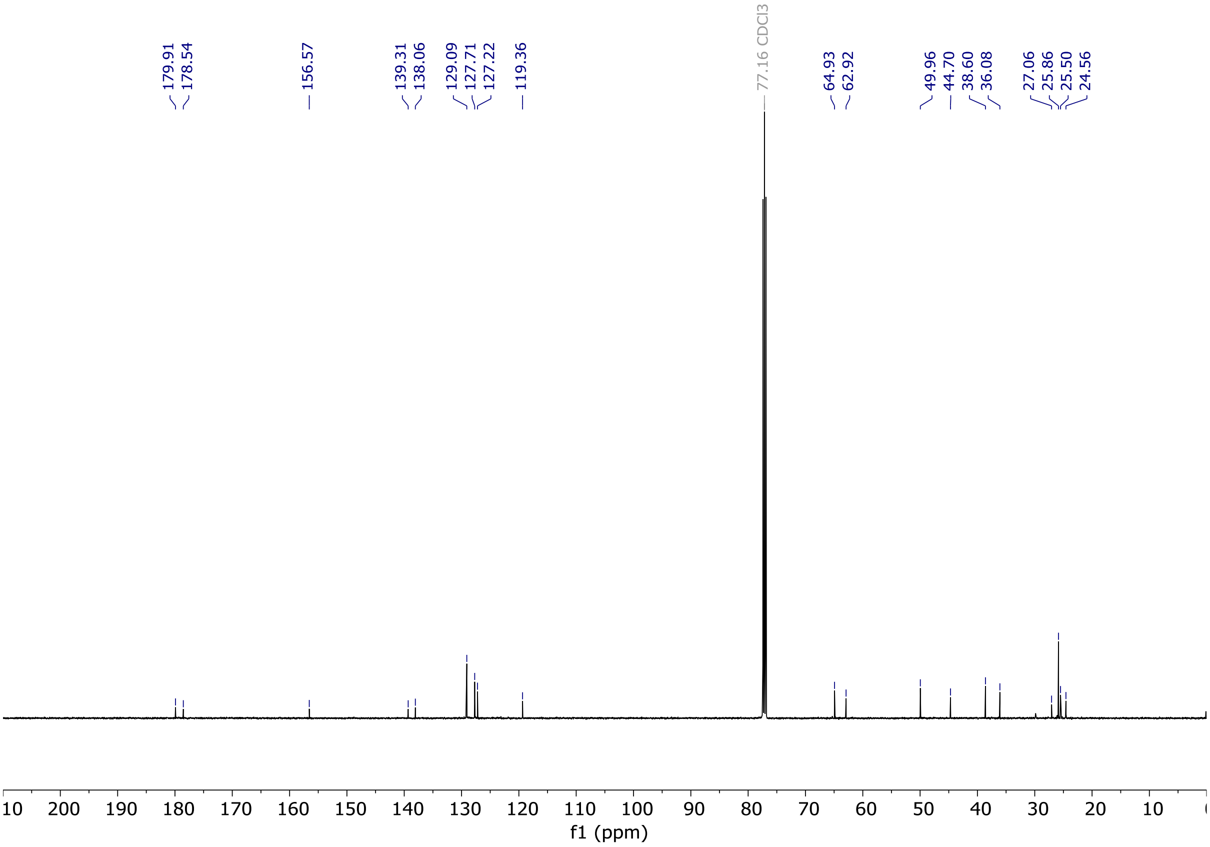

^{1}H NMR (400 MHz, $CDCl_3$)

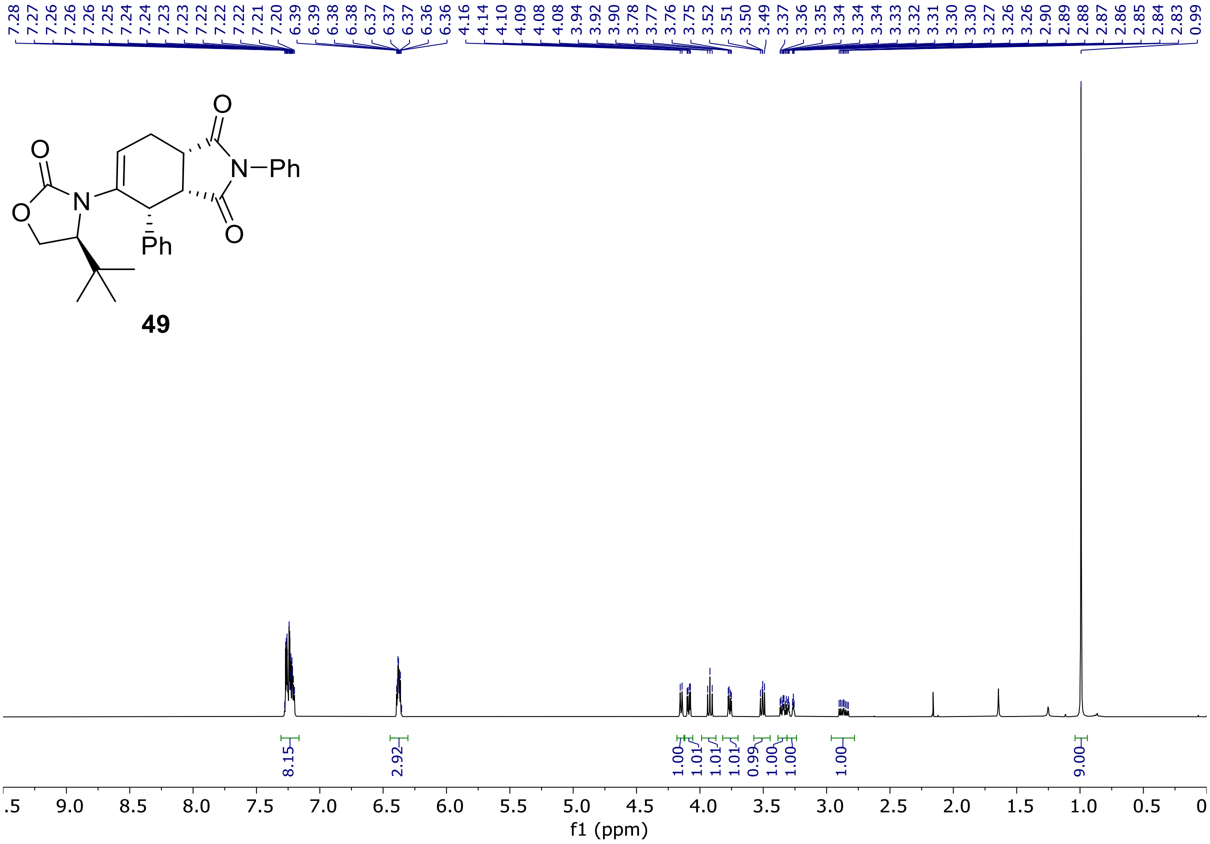

^{13}C {^{1}H} NMR (126 MHz, $CDCl_3$)

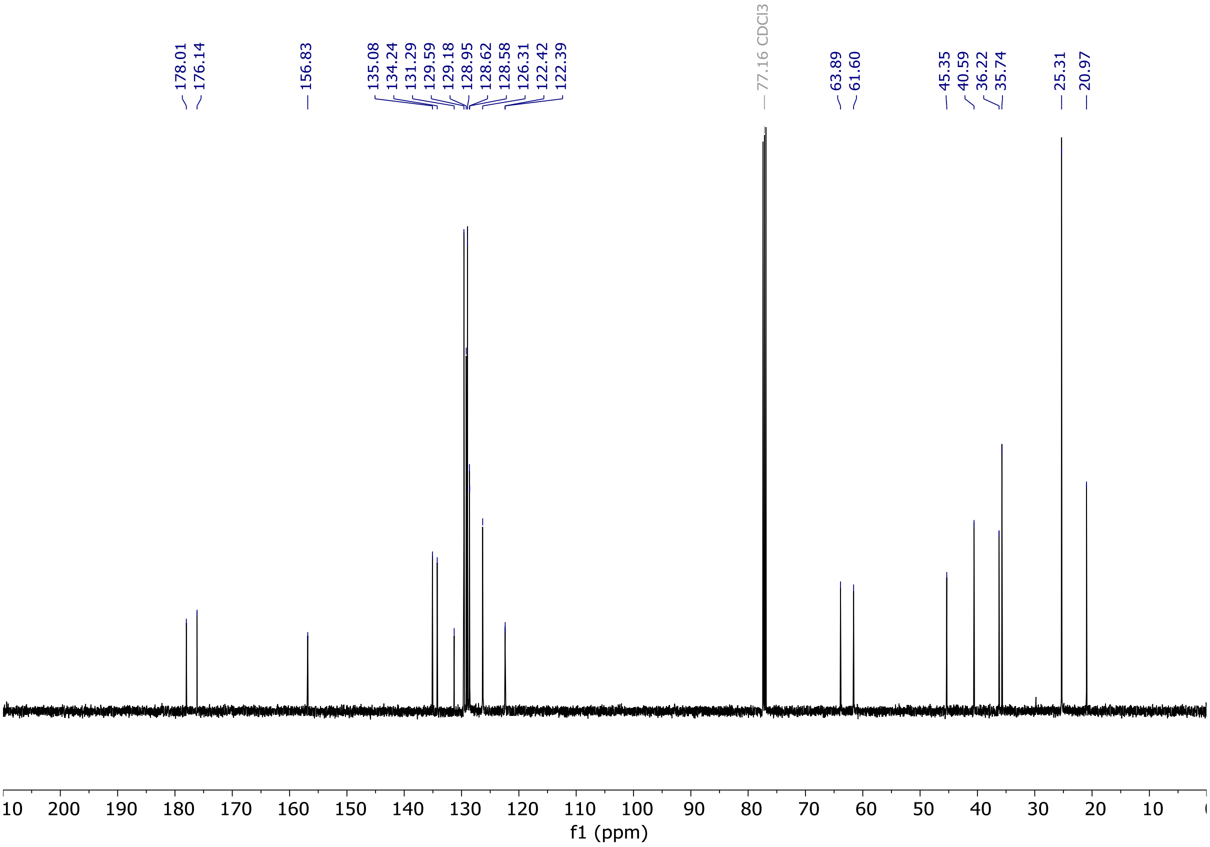

^{1}H NMR (400 MHz, $CDCl_3$)

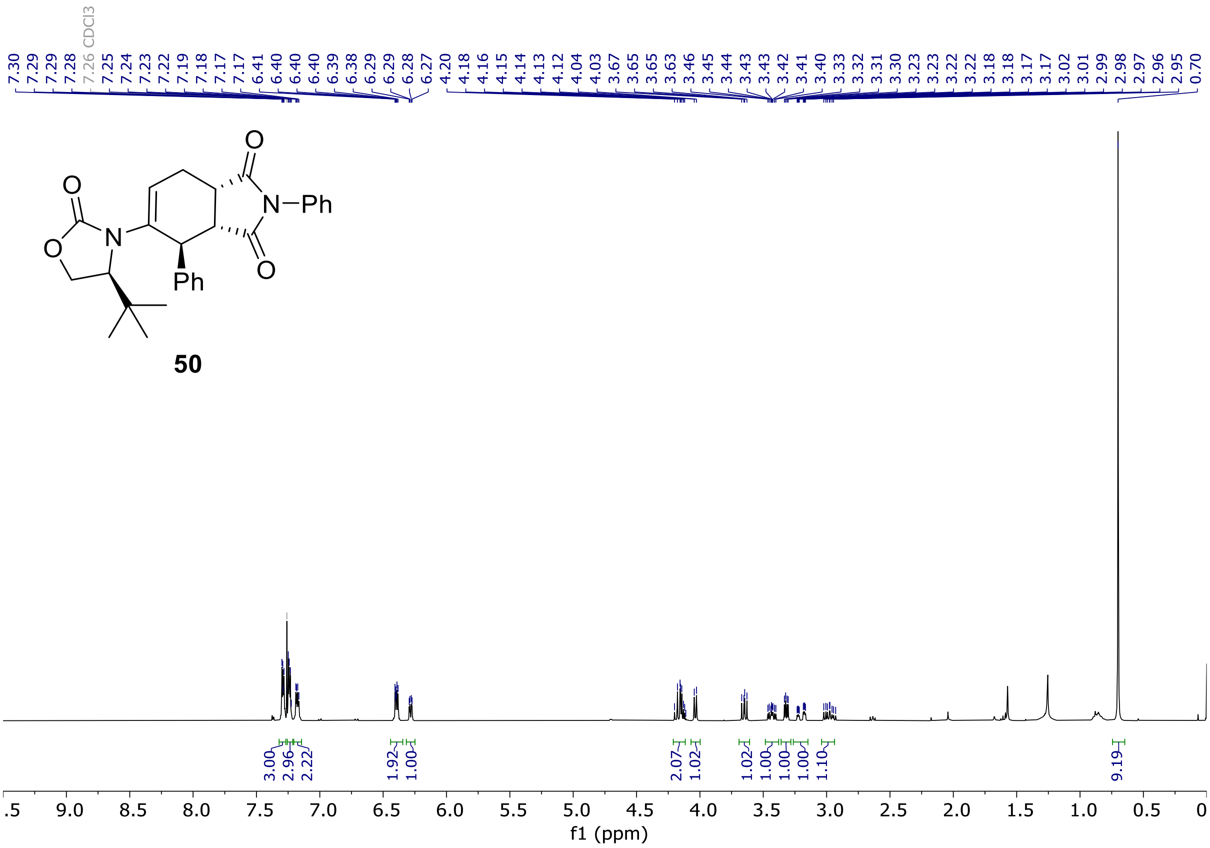

^{13}C {^{1}H} NMR (101 MHz, $CDCl_3$)

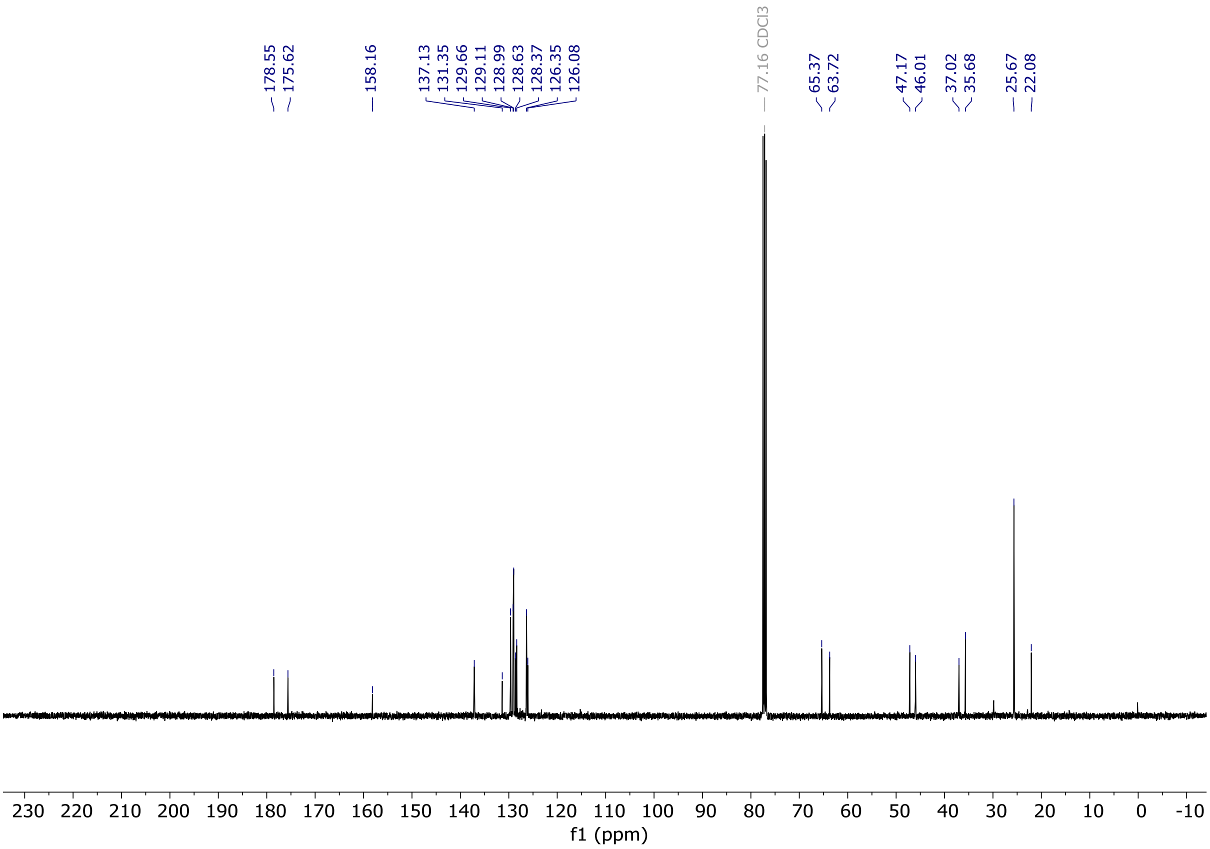

^{1}H NMR (400 MHz, $CDCl_3$)

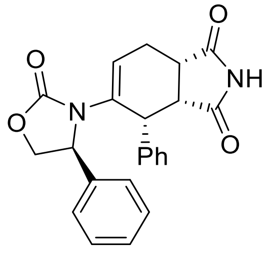

51

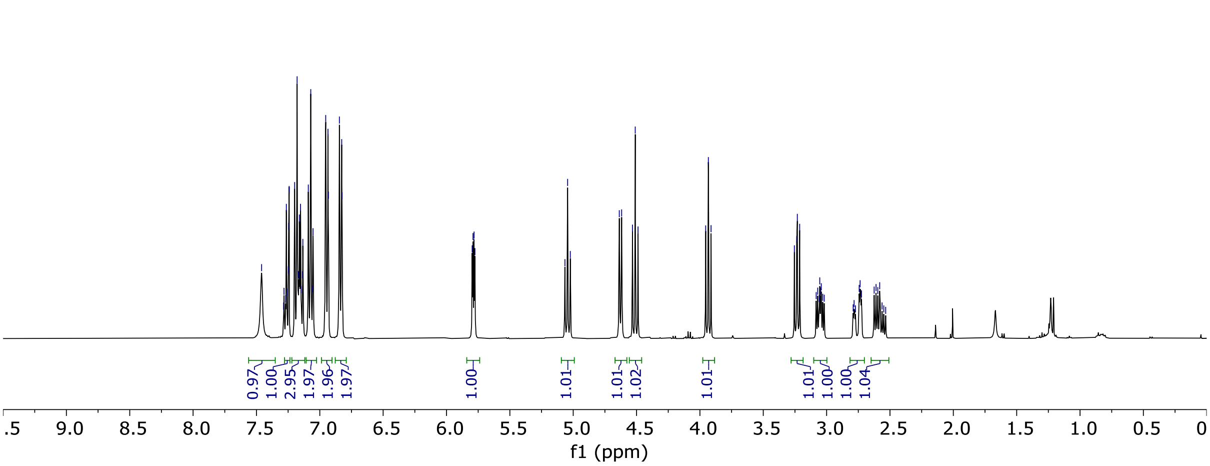

^{13}C {^{1}H} NMR (101 MHz, $CDCl_3$)

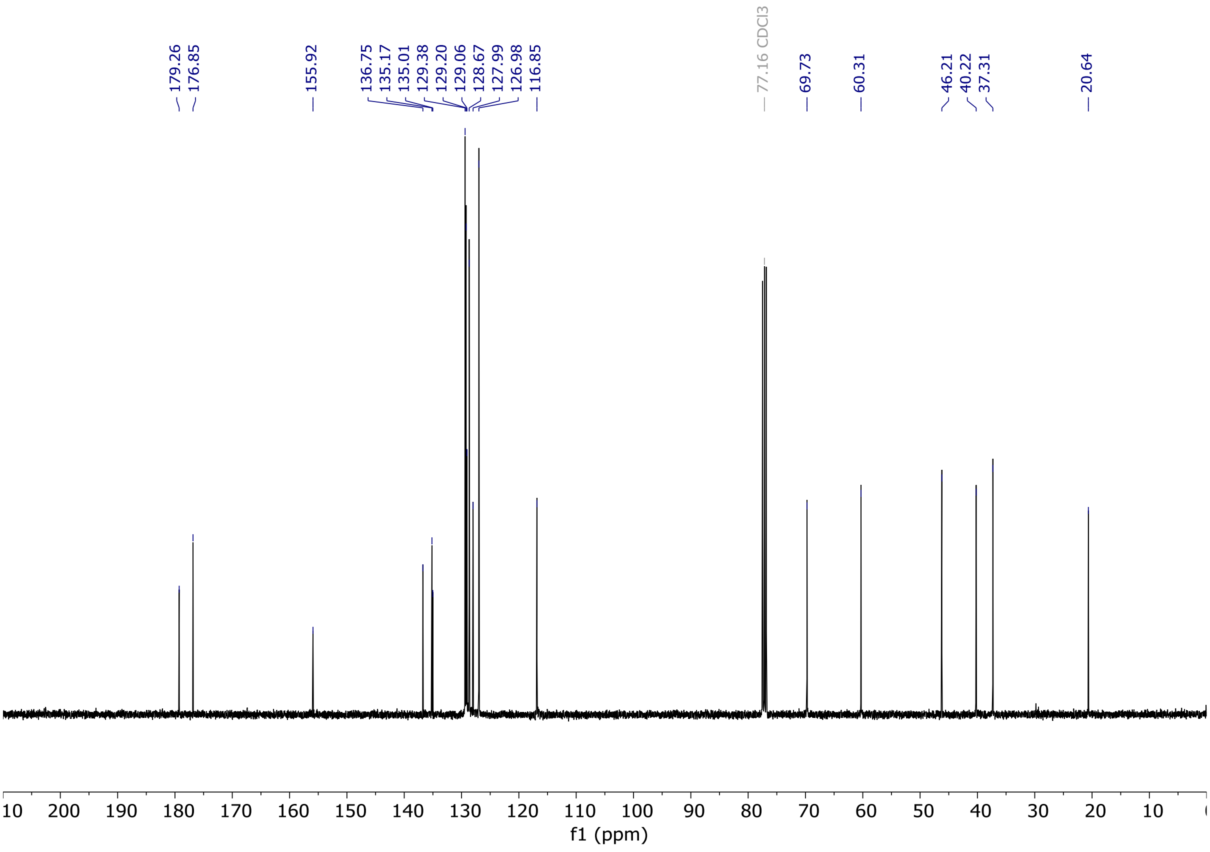

^{1}H NMR (400 MHz, $CDCl_3$)

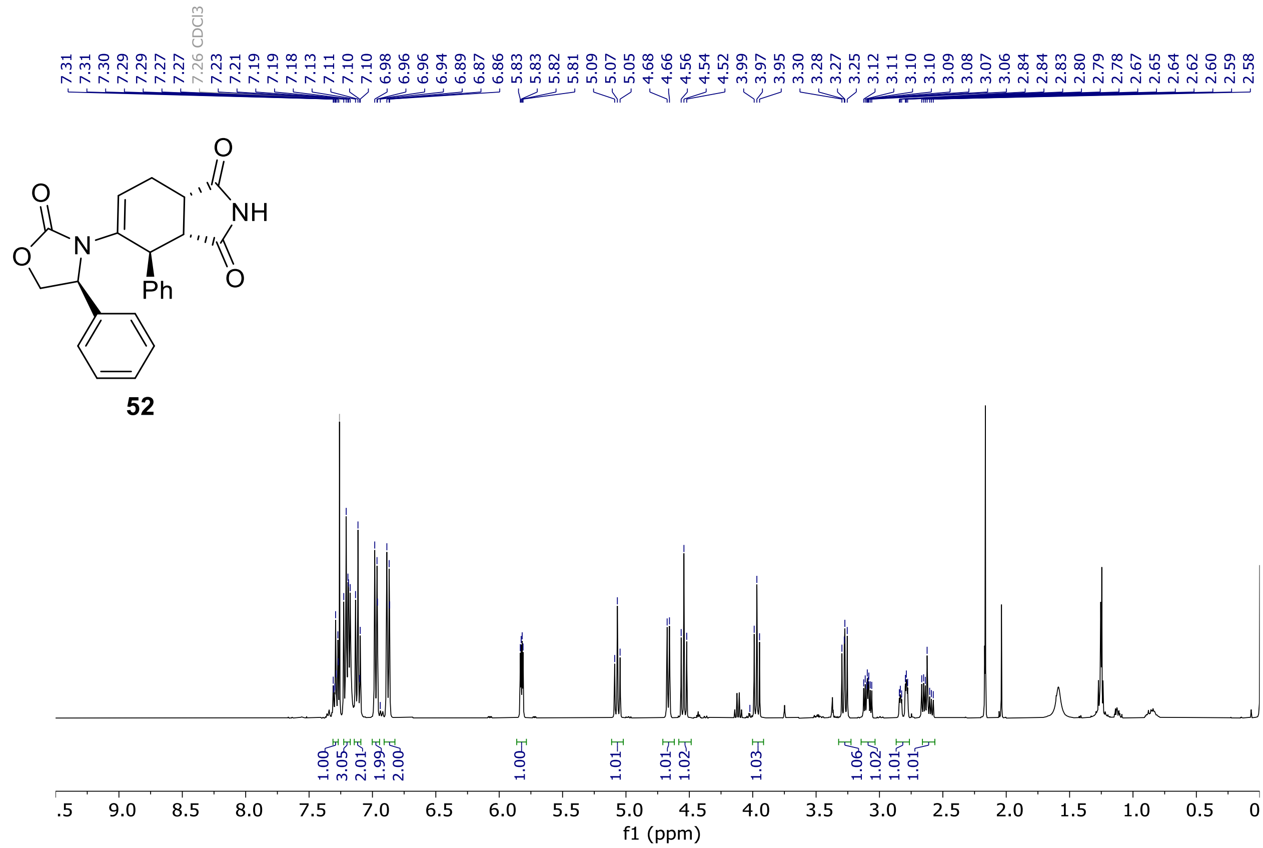

^{13}C {^{1}H} NMR (101 MHz, $CDCl_3$)

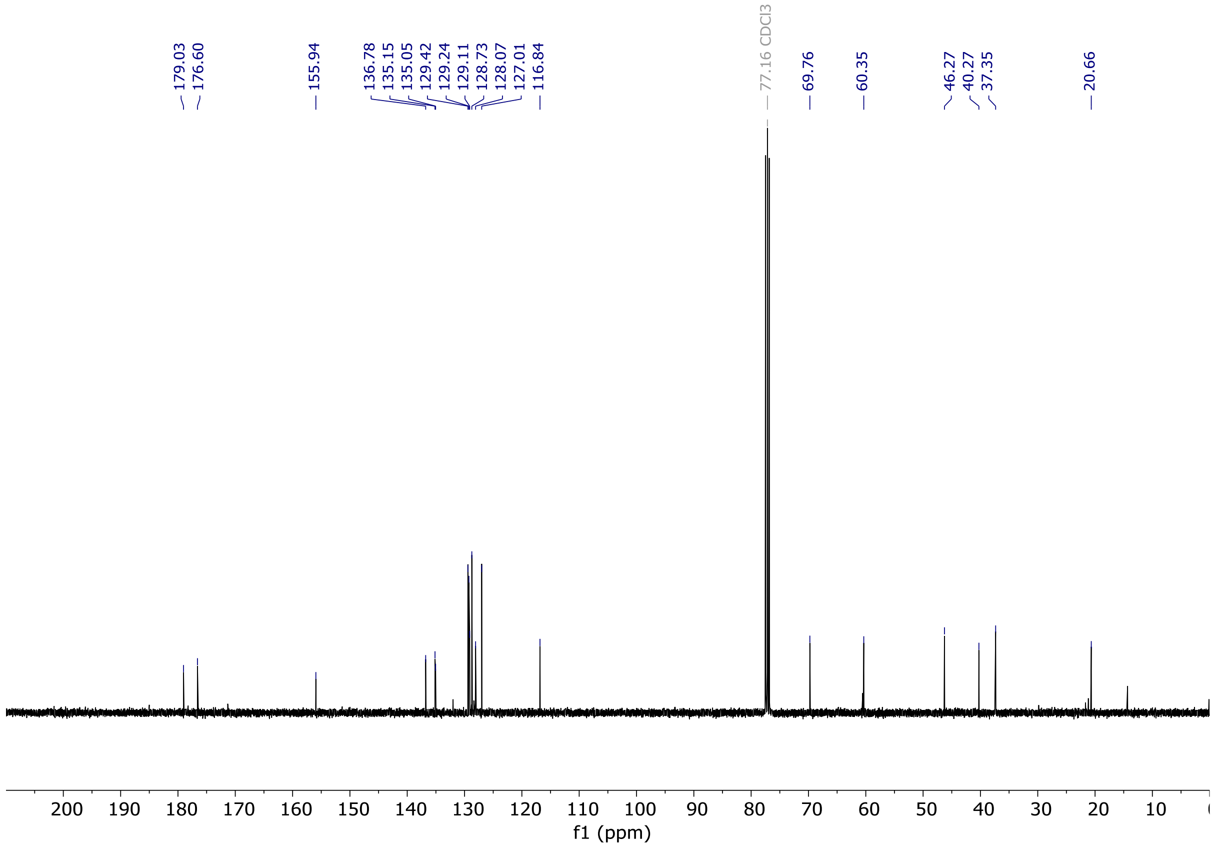

^{1}H NMR (400 MHz, $CDCl_3$)

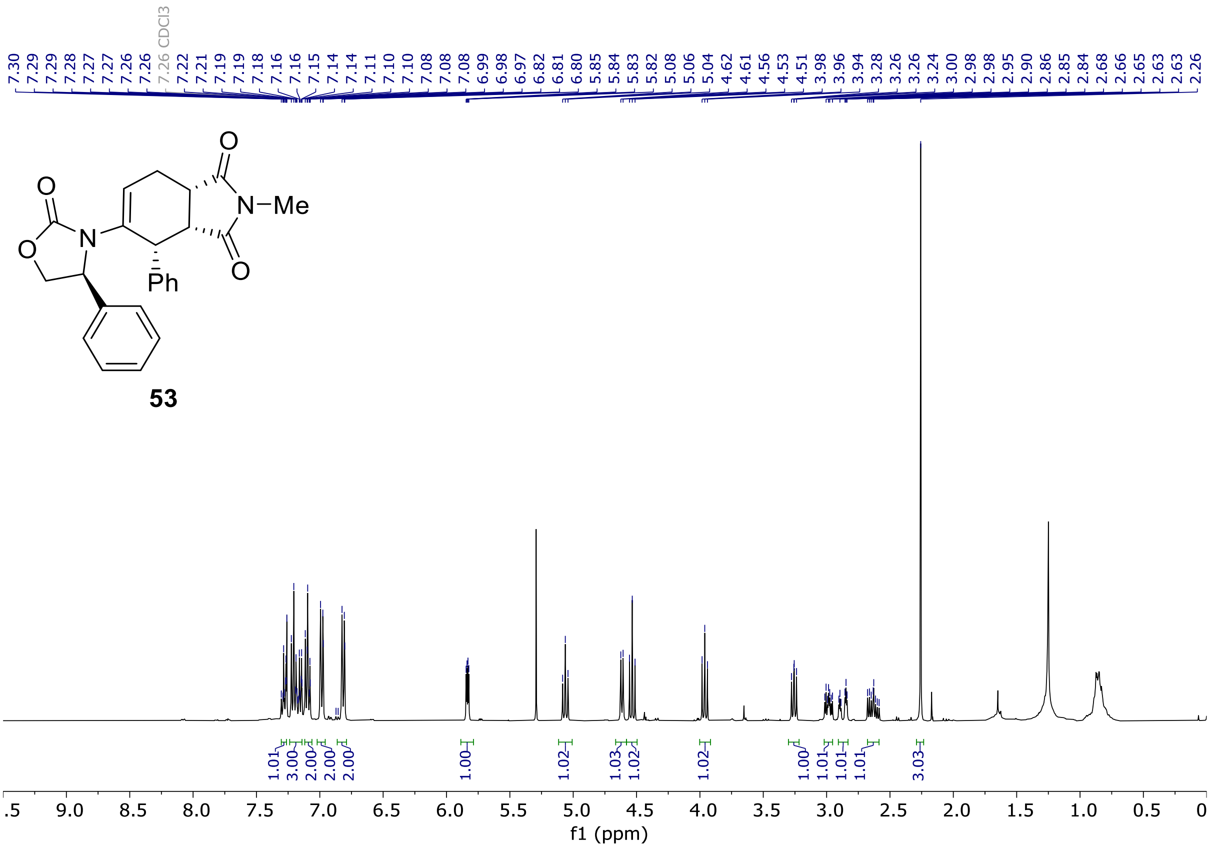

^{13}C {^{1}H} NMR (126 MHz, $CDCl_3$)

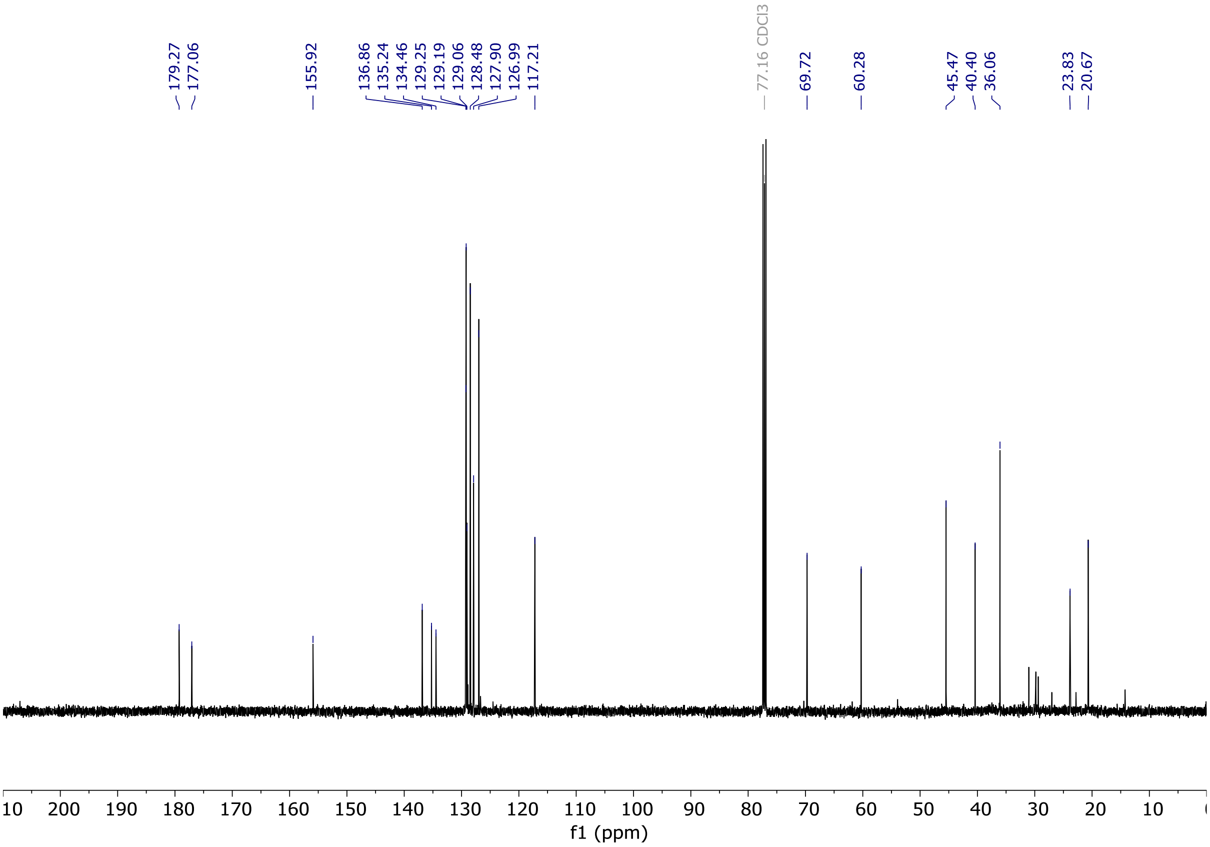

^{1}H NMR (400 MHz, CDCl$_3$)

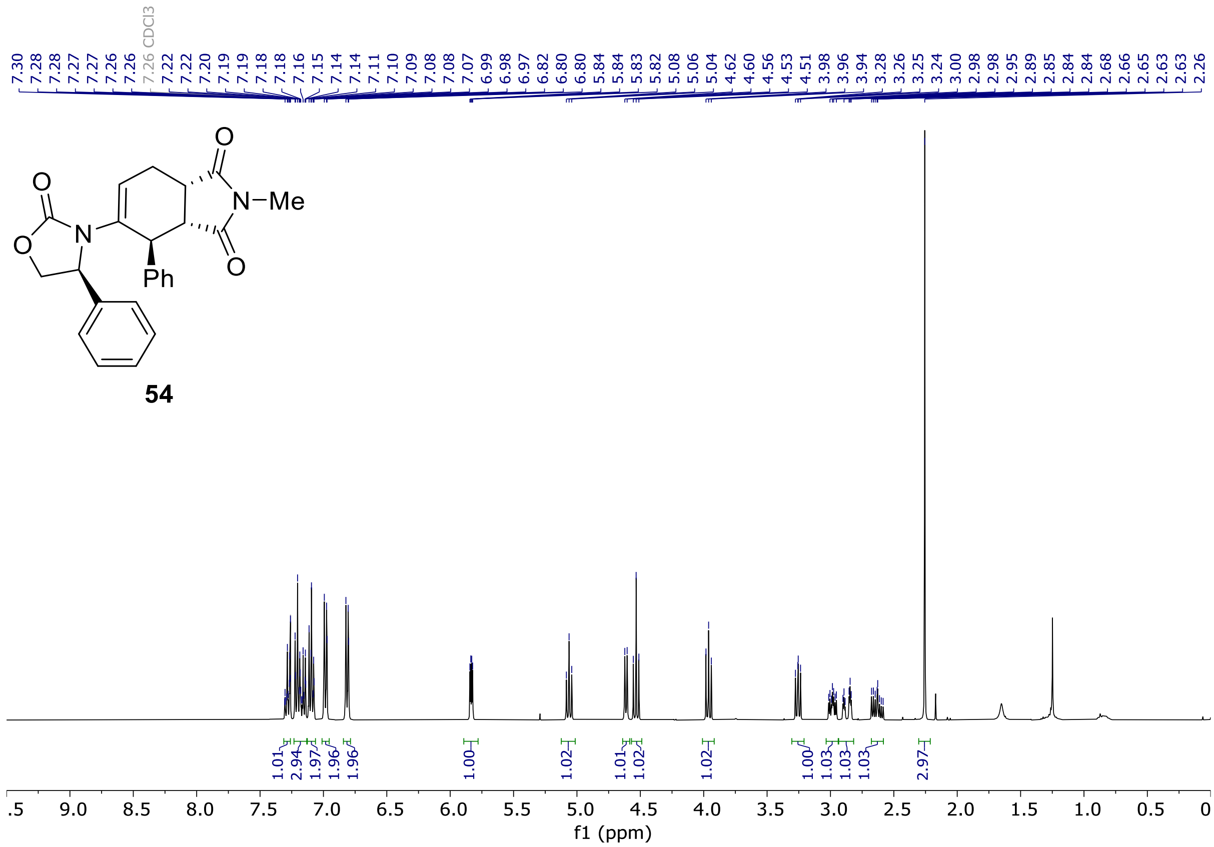

^{13}C {^{1}H} NMR (101 MHz, CDCl$_3$)

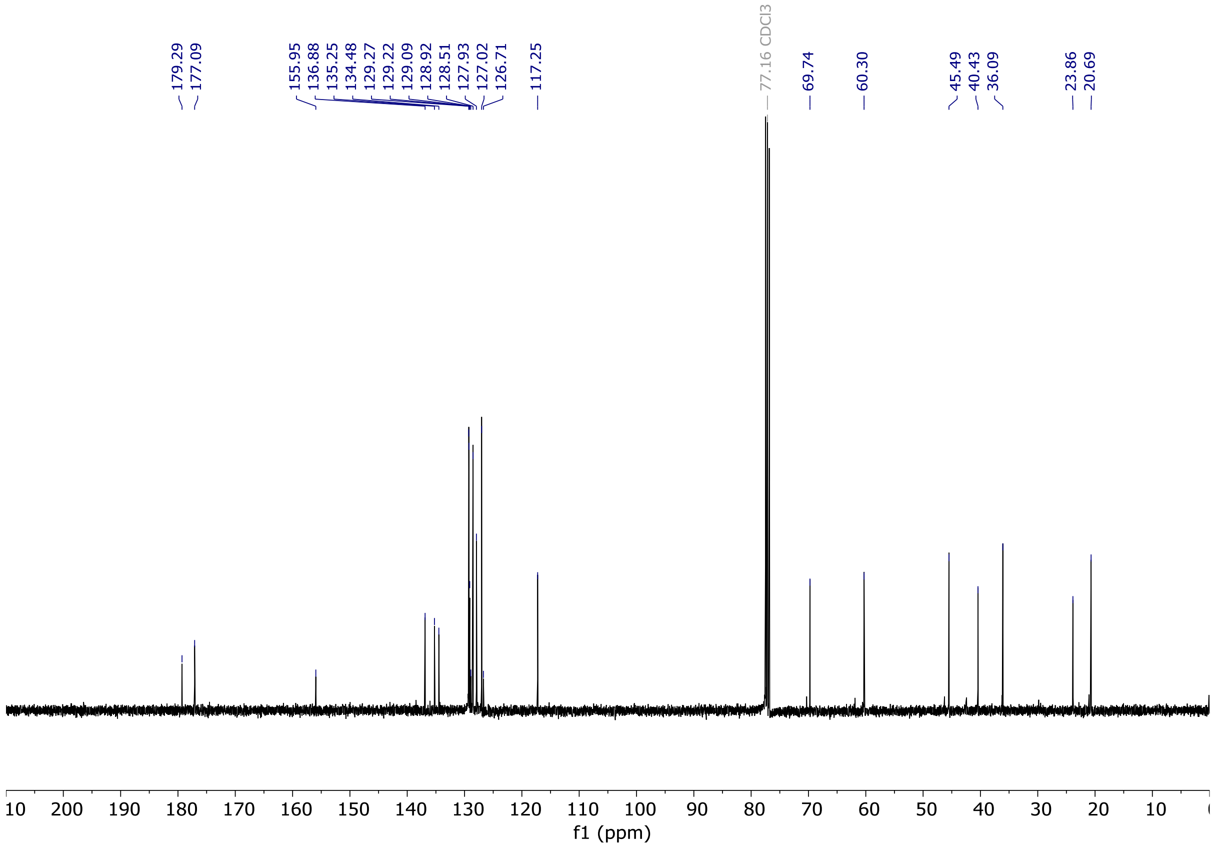

^{1}H NMR (400 MHz, $CDCl_3$)

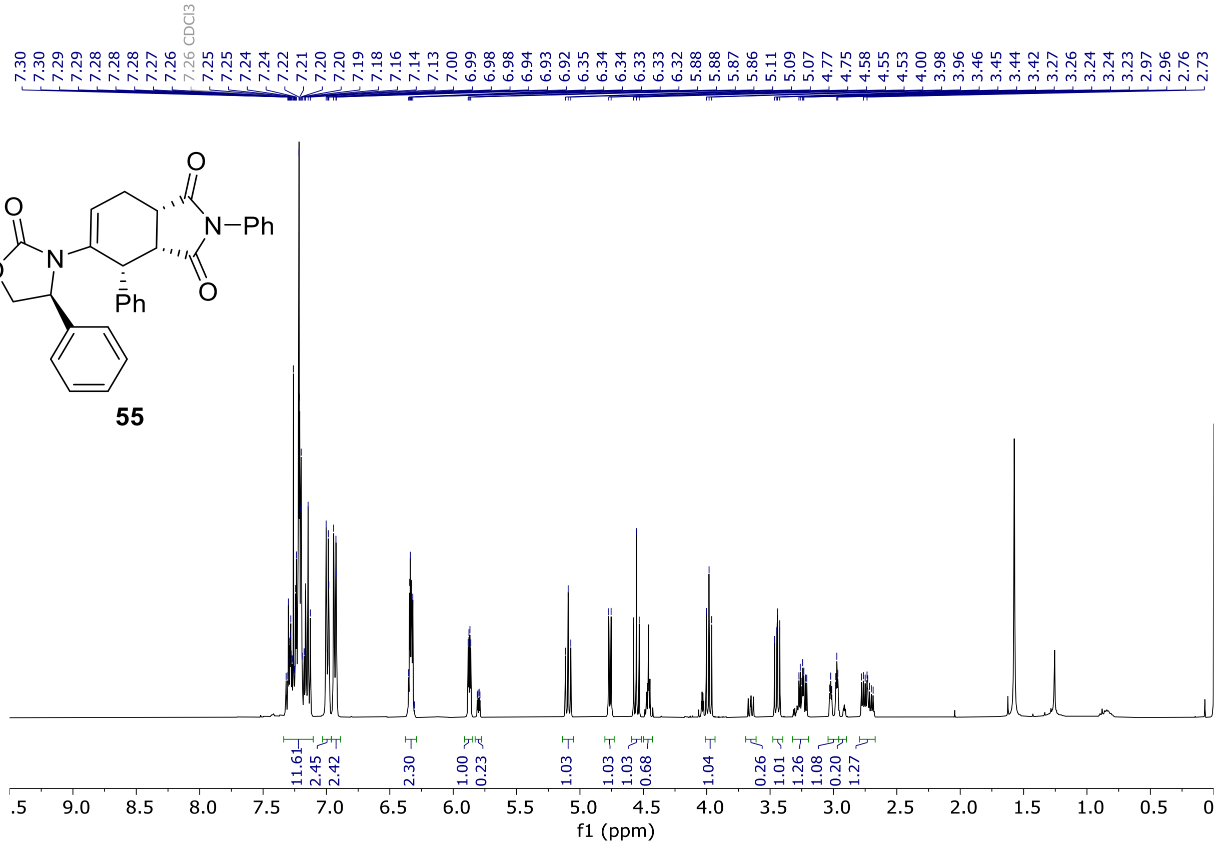

^{13}C {^{1}H} NMR (101 MHz, $CDCl_3$)

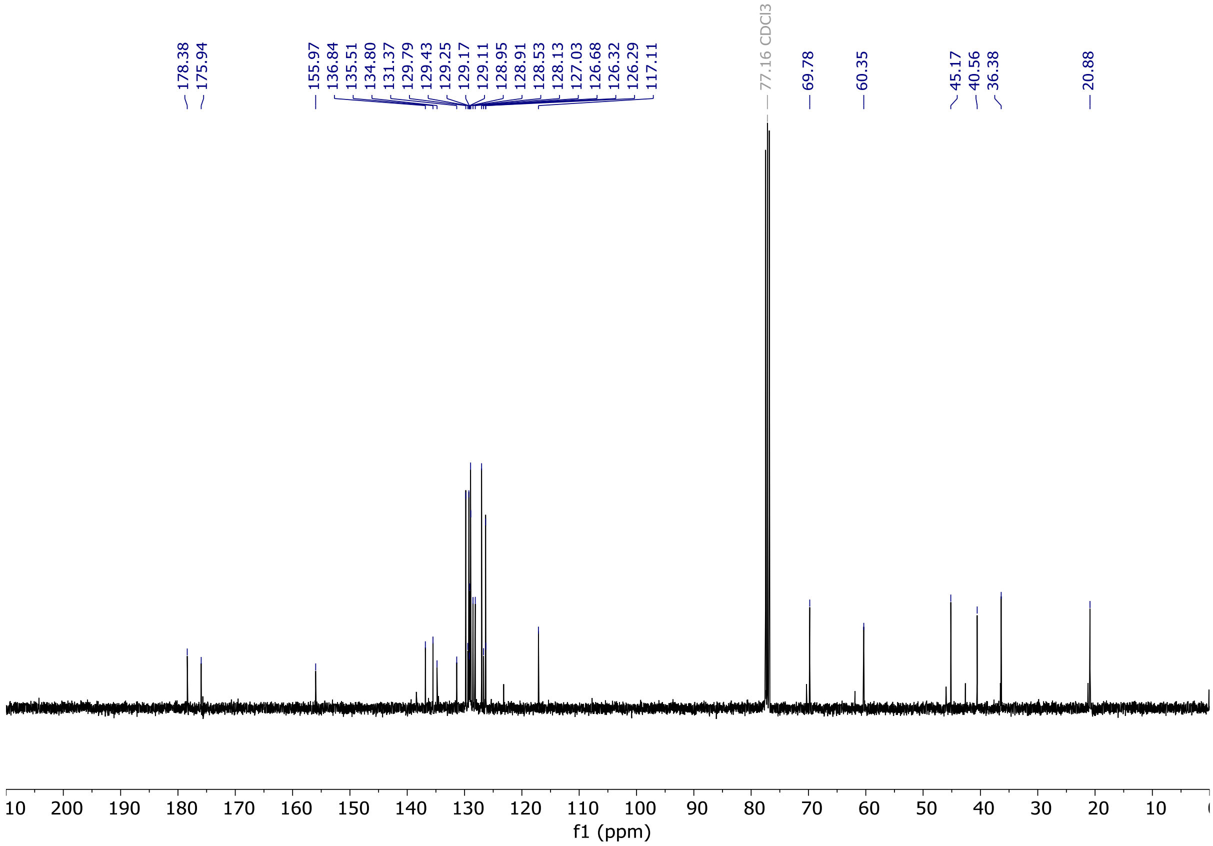

^{1}H NMR (400 MHz, $CDCl_3$)

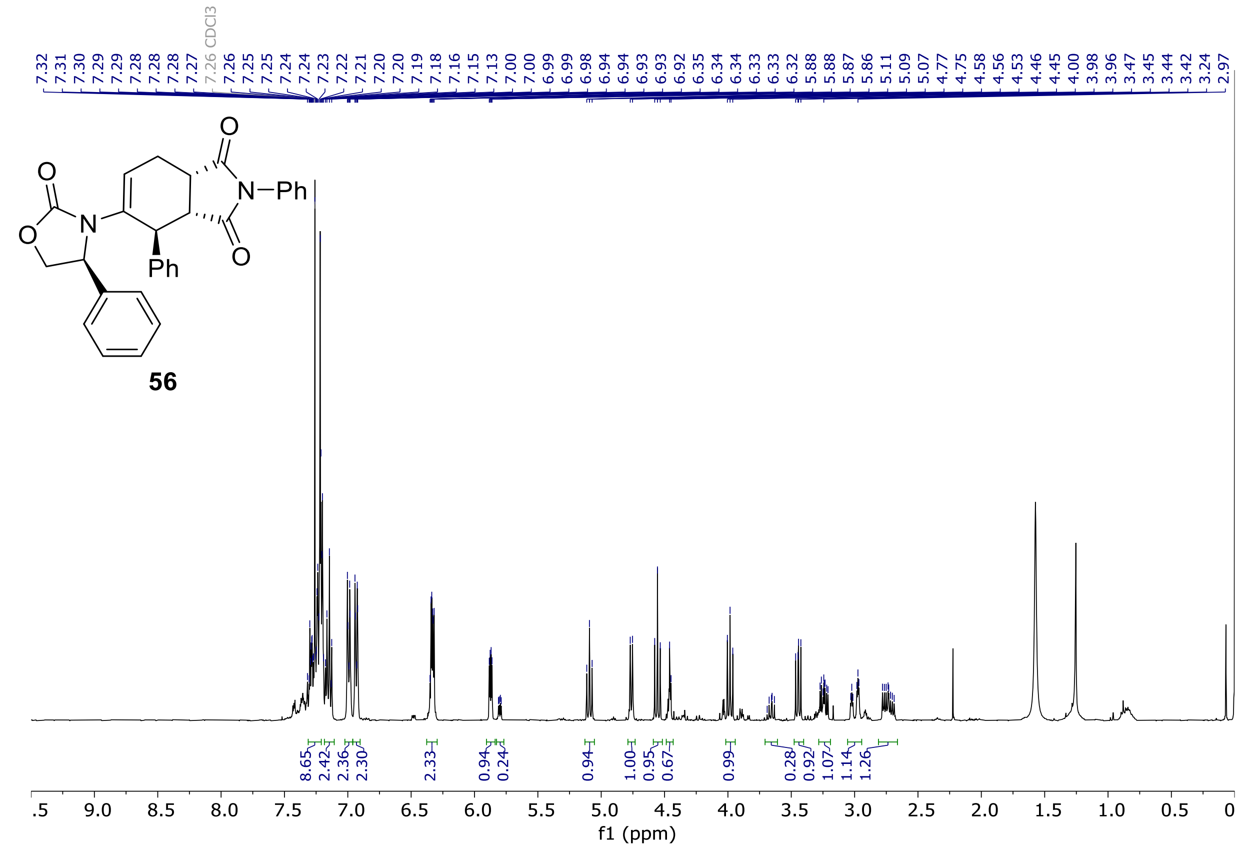

^{13}C {^{1}H} NMR (101 MHz, $CDCl_3$)

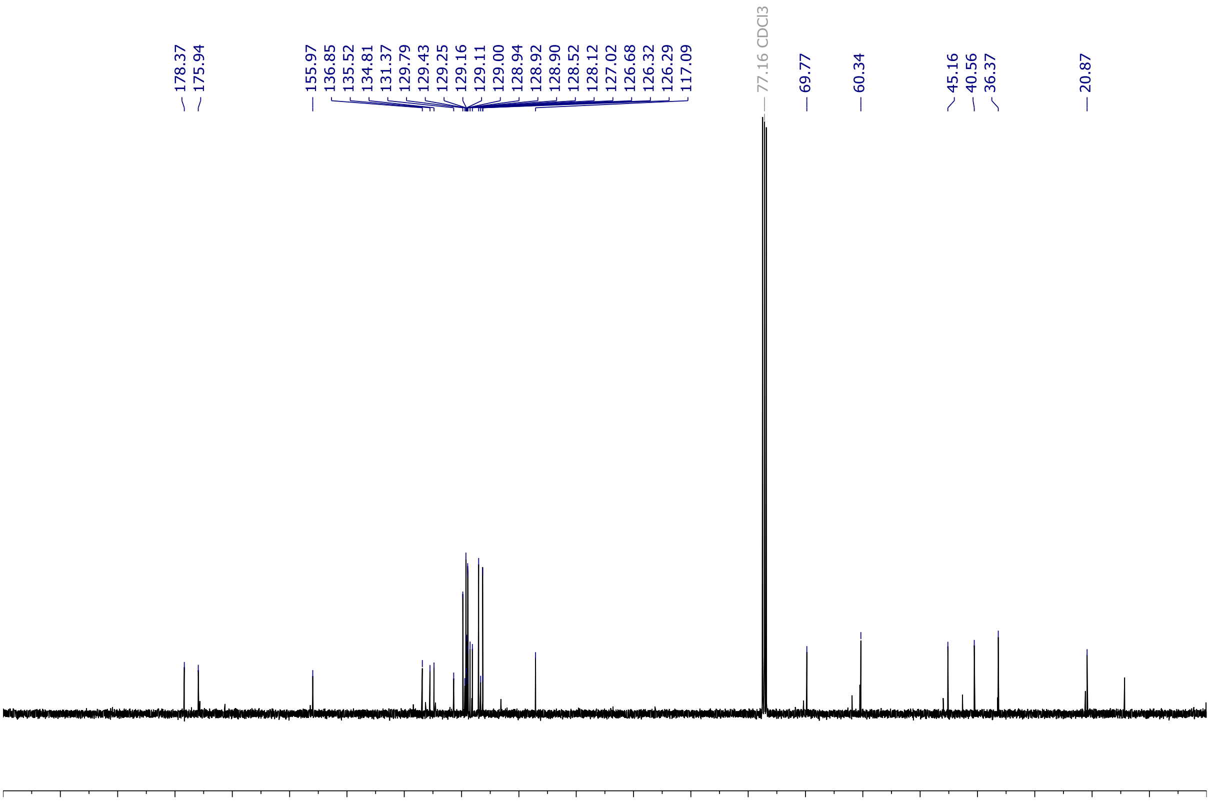

^{1}H NMR (400 MHz, $CDCl_3$)

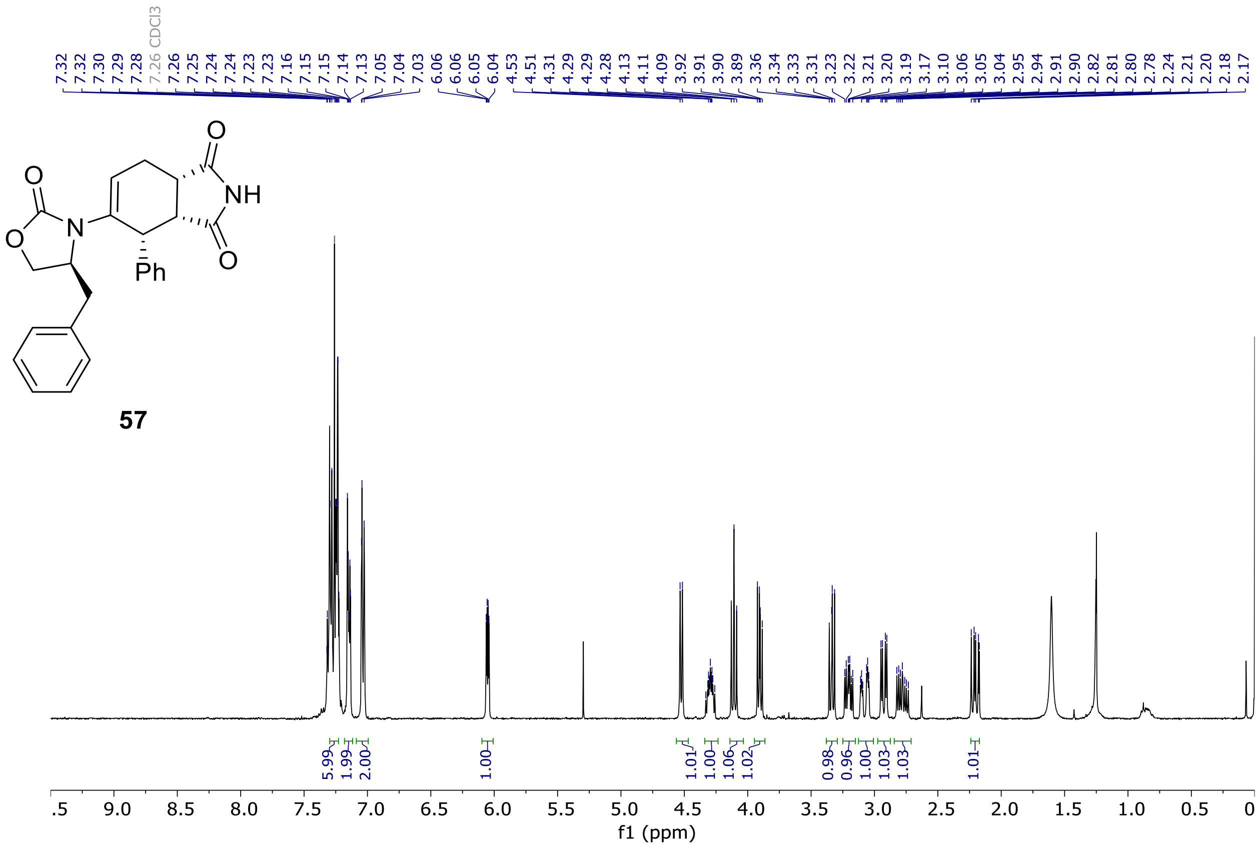

57

^{13}C {^{1}H} NMR (101 MHz, $CDCl_3$)

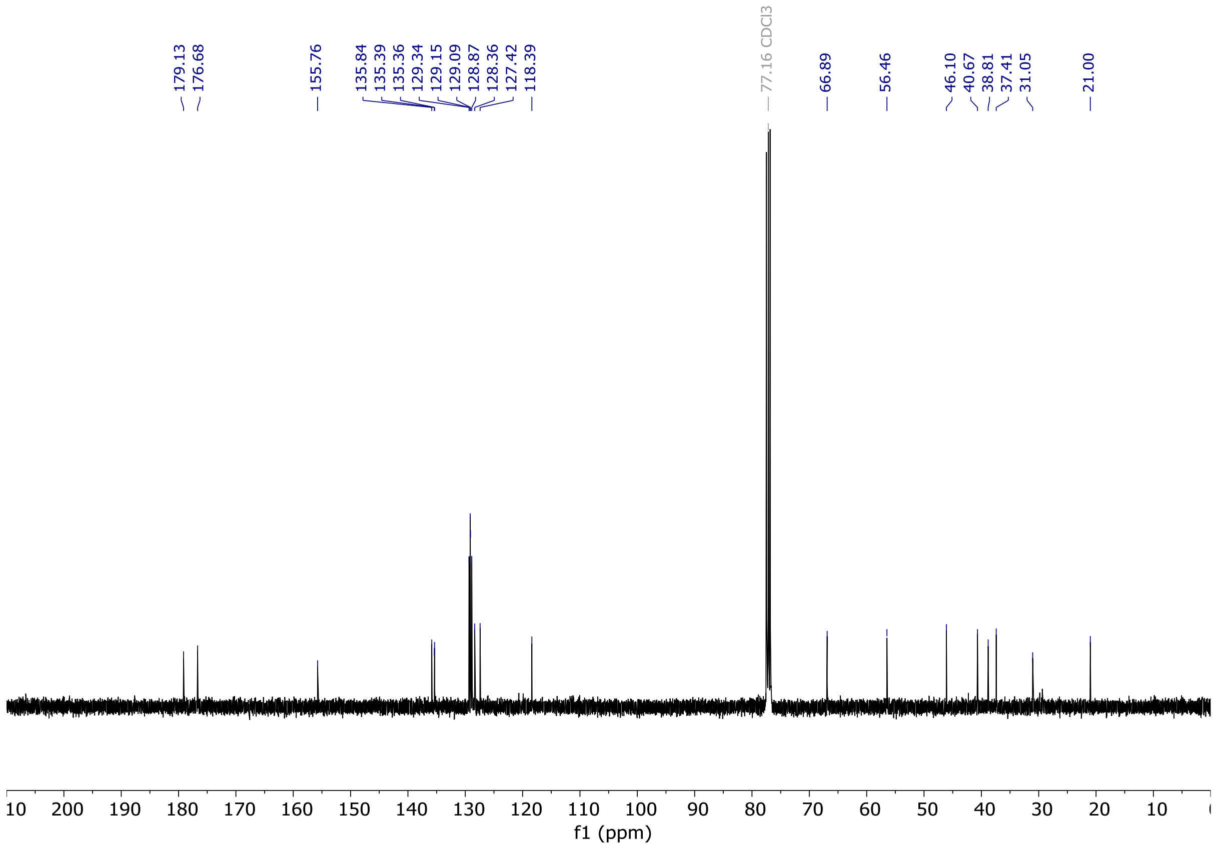

^{1}H NMR (500 MHz, CDCl$_3$)

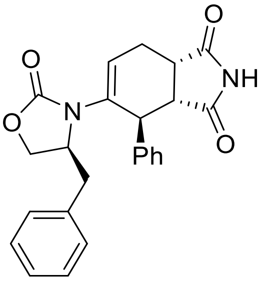

58

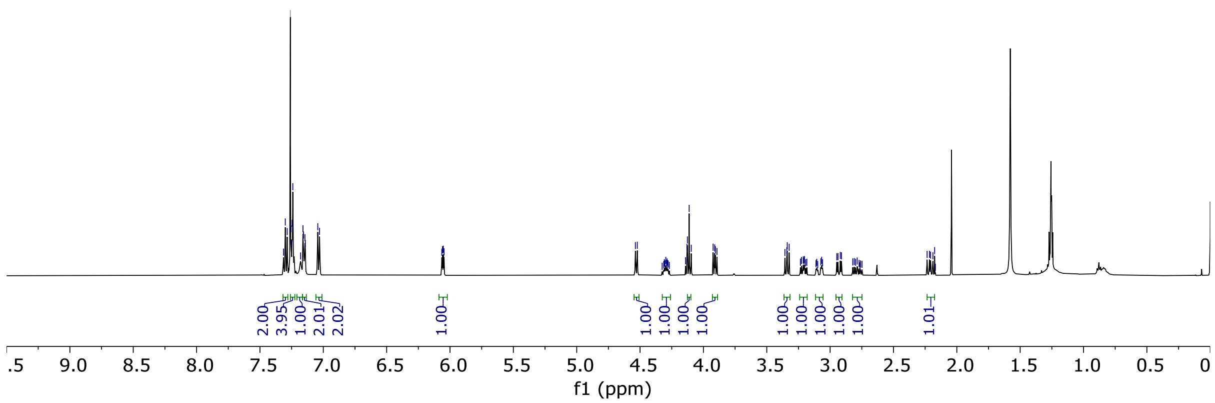

^{13}C {1H} NMR (101 MHz, CDCl$_3$)

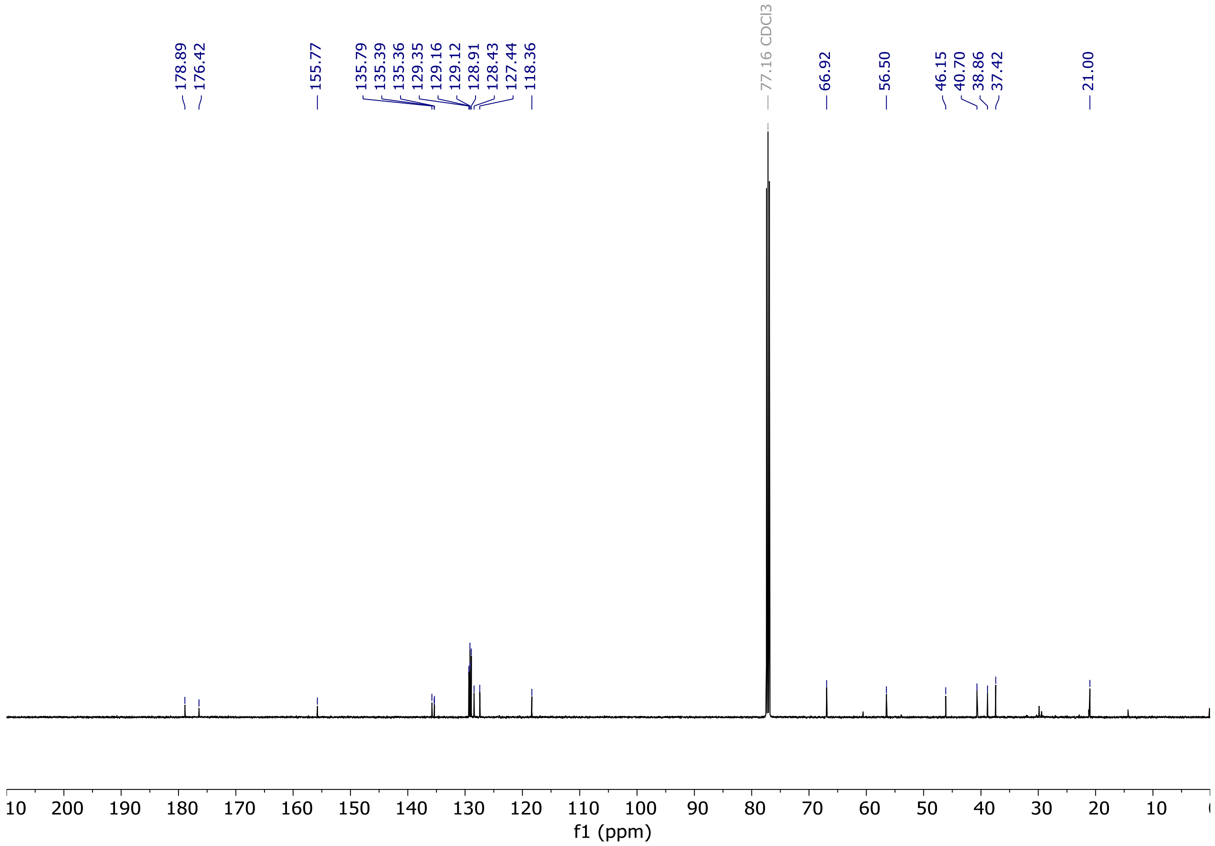

^{1}H NMR (400 MHz, $CDCl_3$)

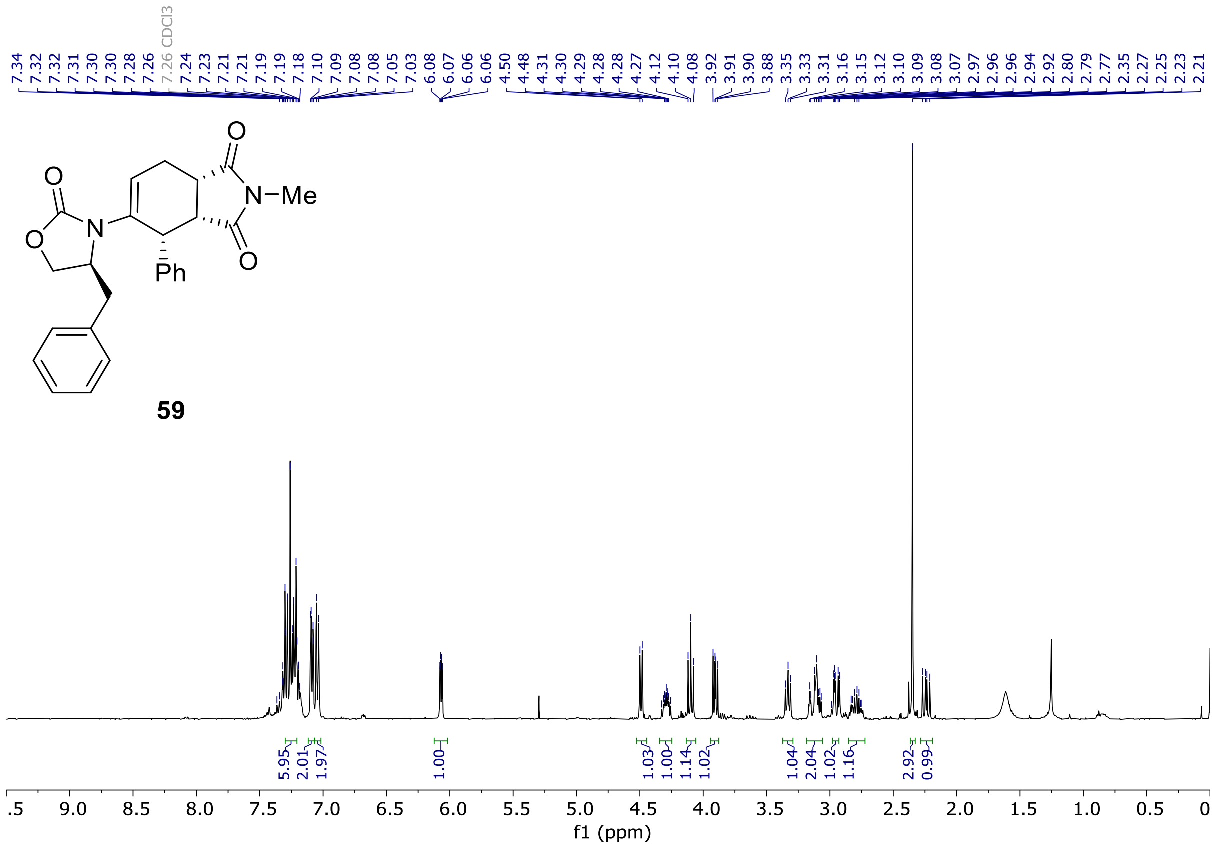

^{13}C {1H} NMR (101 MHz, $CDCl_3$)

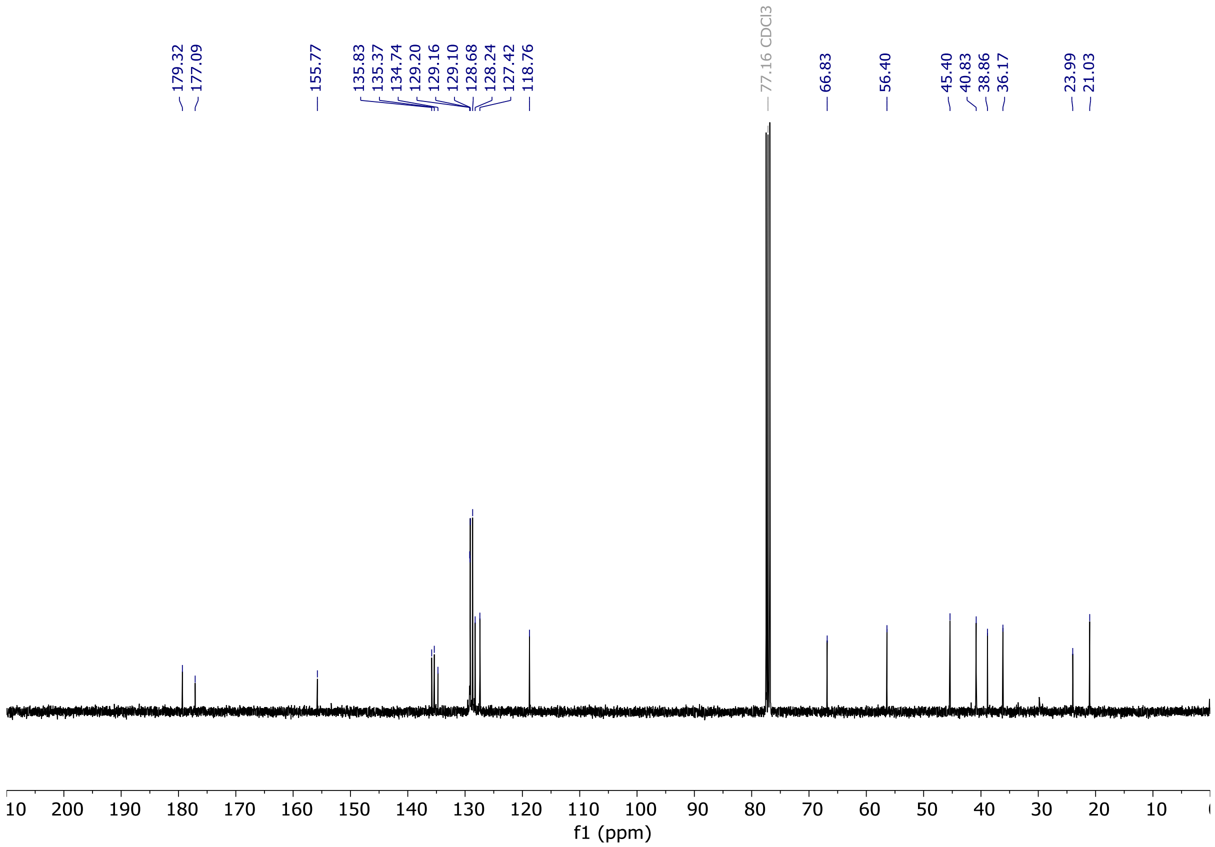

^{1}H NMR (500 MHz, CDCl$_3$)

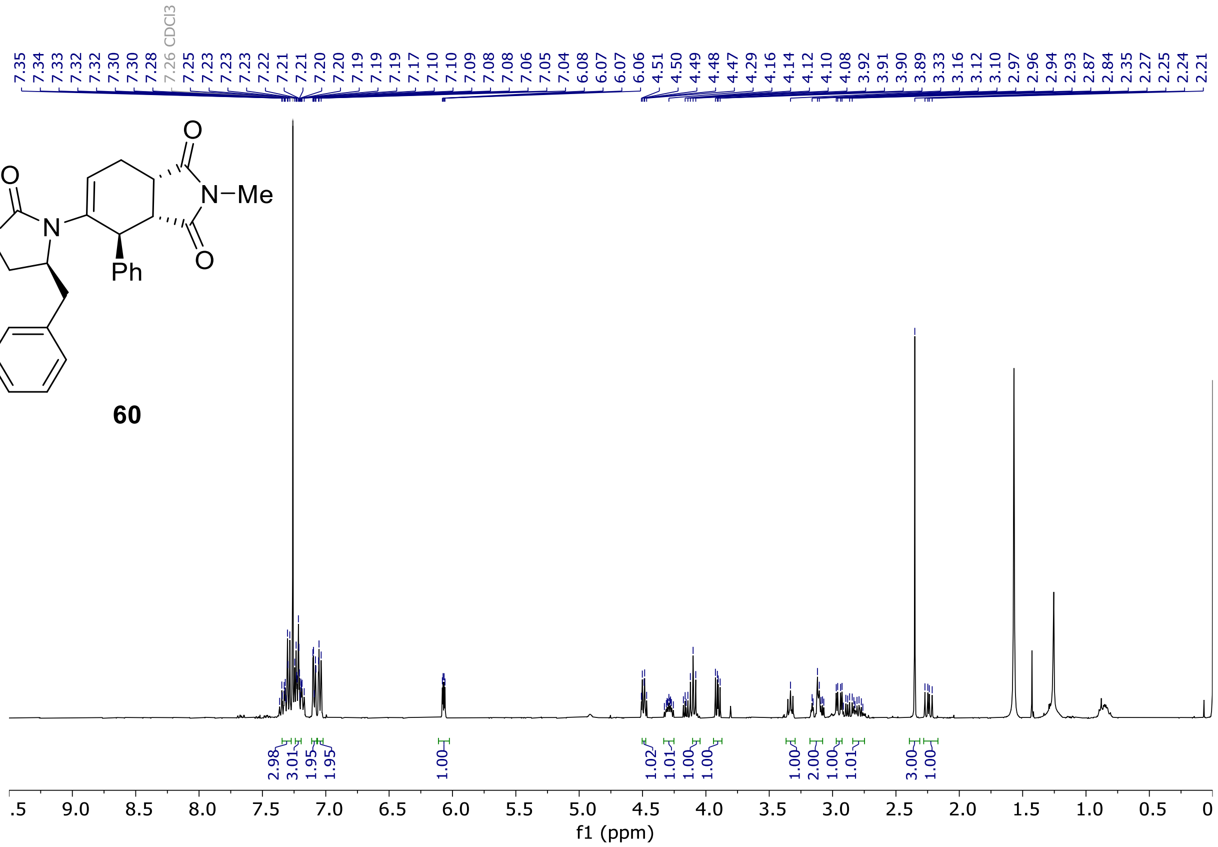

60

^{13}C {1H} NMR (101 MHz, CDCl$_3$)

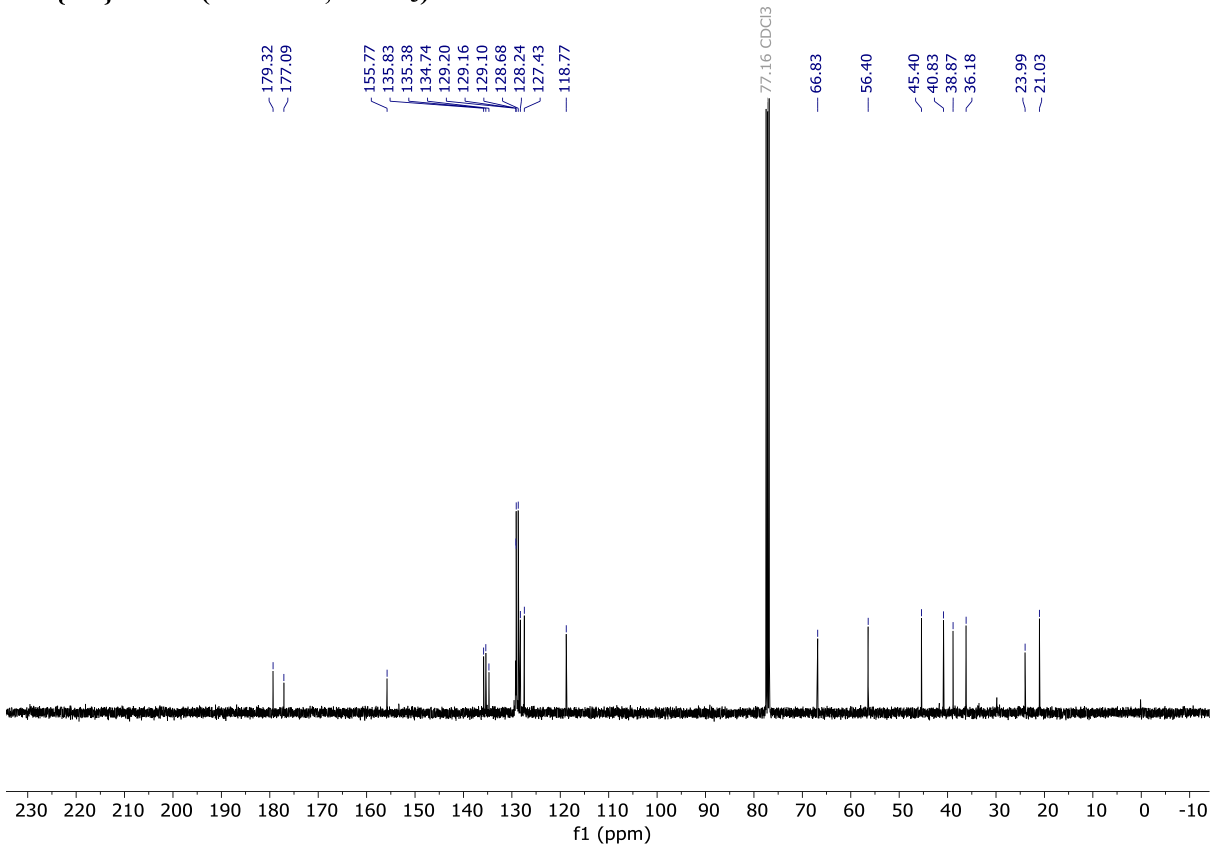

^{1}H NMR (400 MHz, CDCl$_3$)

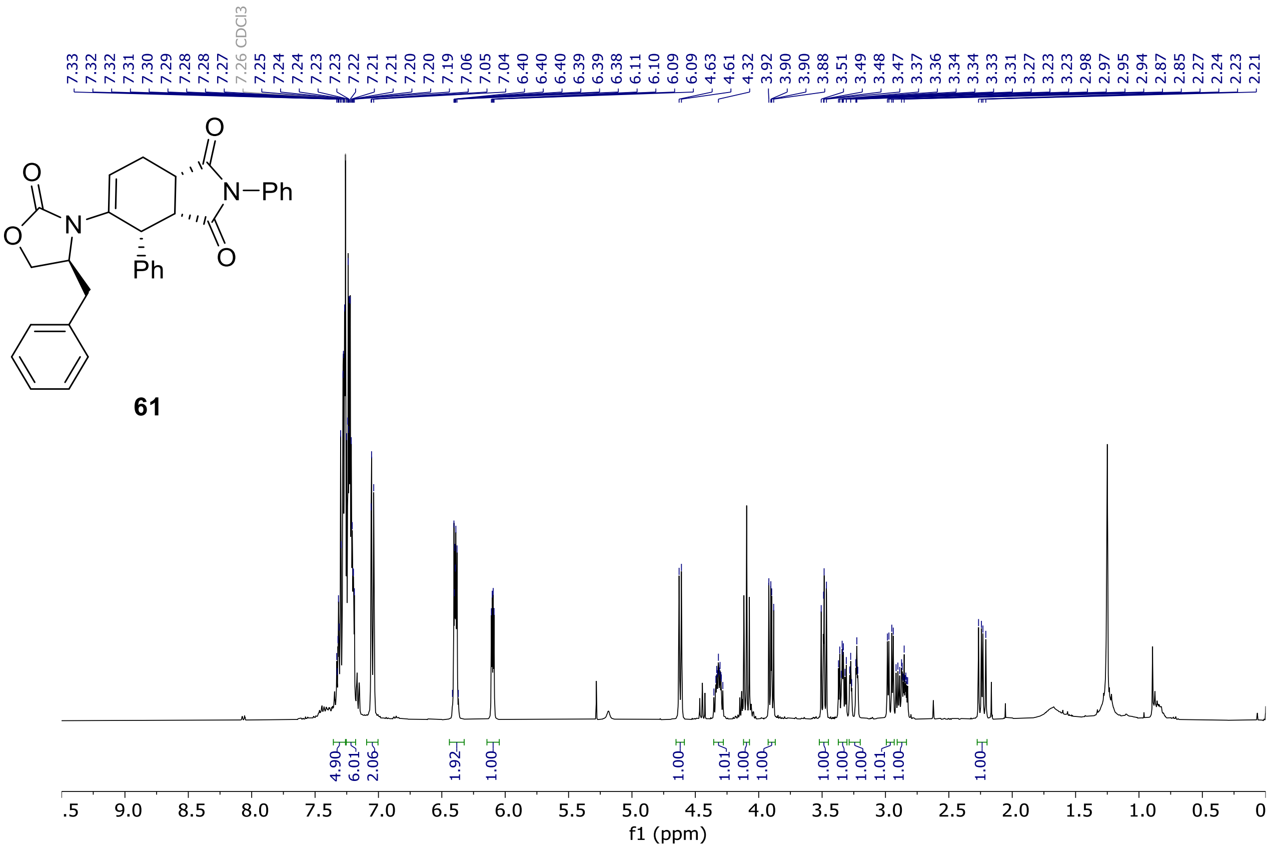

61

^{13}C {1H} NMR (101 MHz, CDCl$_3$)

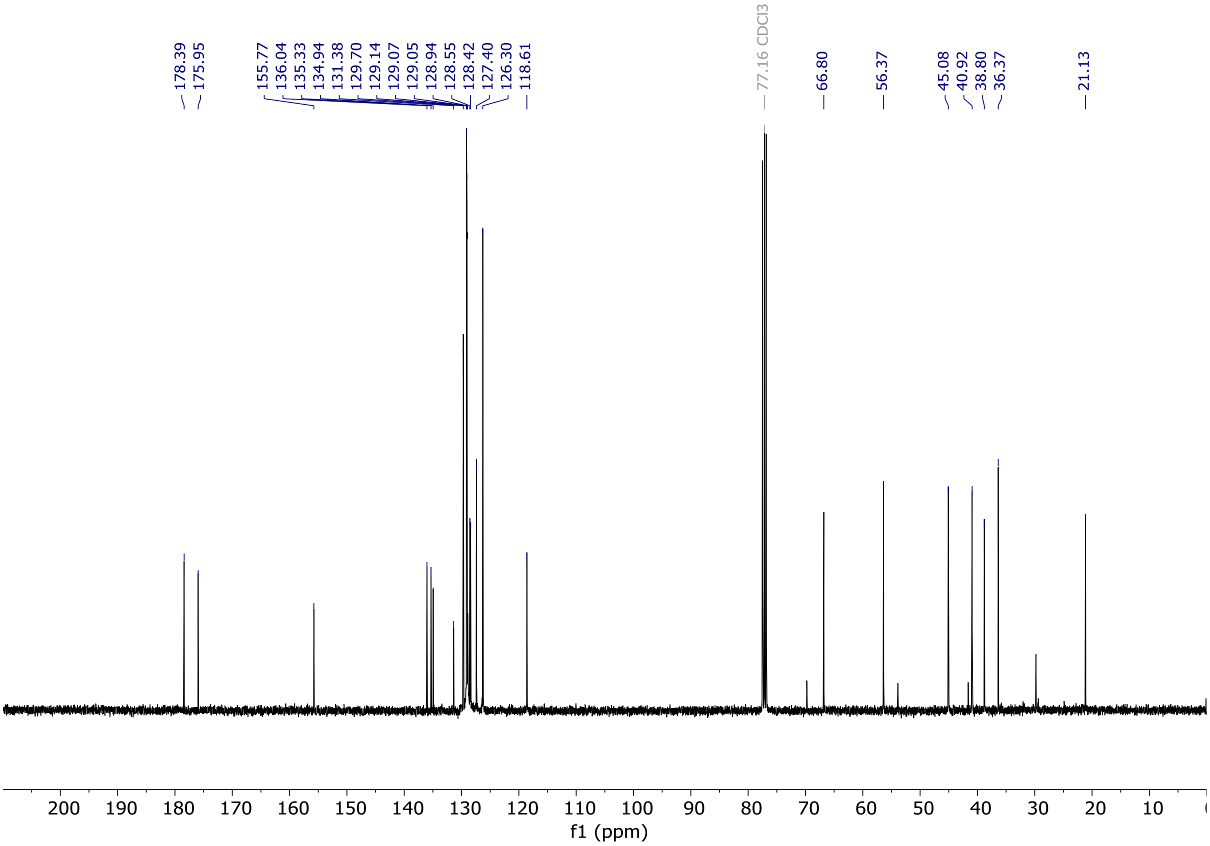

^{1}H NMR (400 MHz, $CDCl_3$)

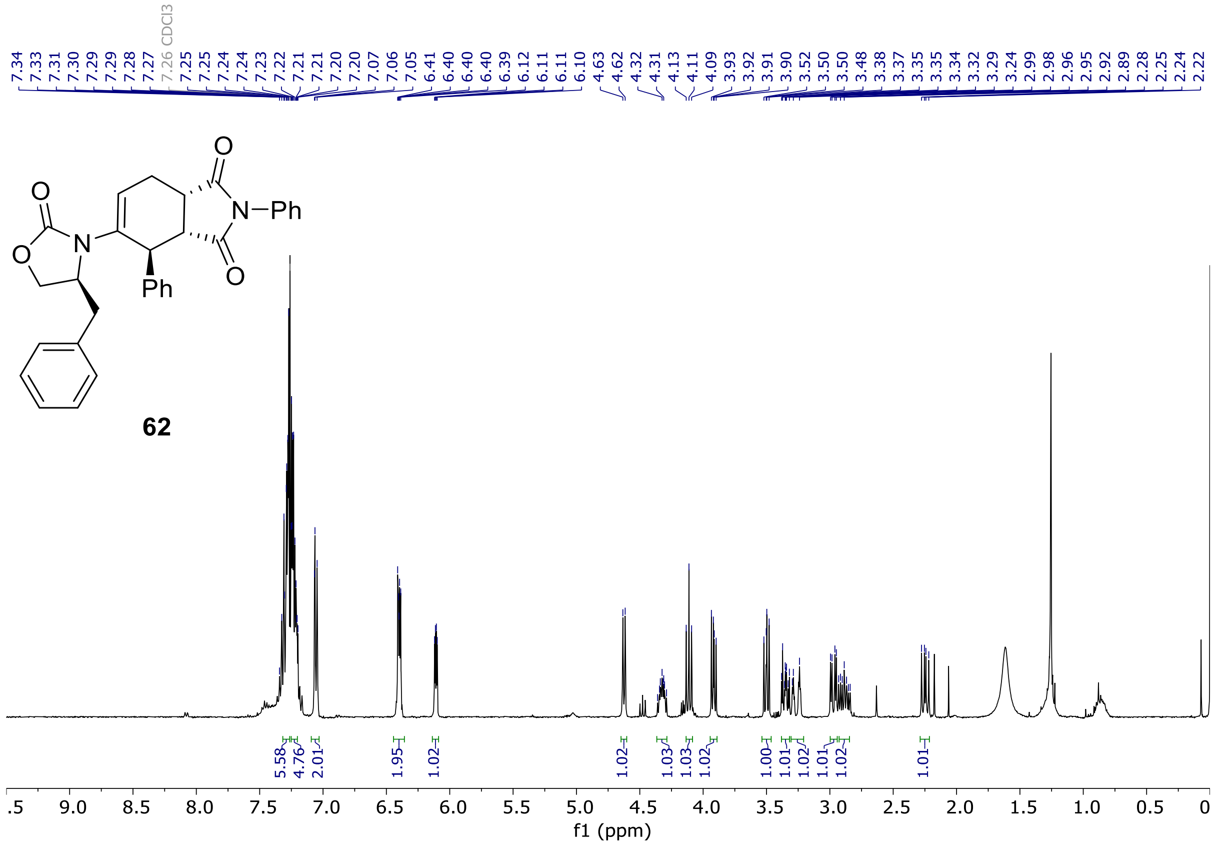

^{13}C {1H} NMR (101 MHz, $CDCl_3$)

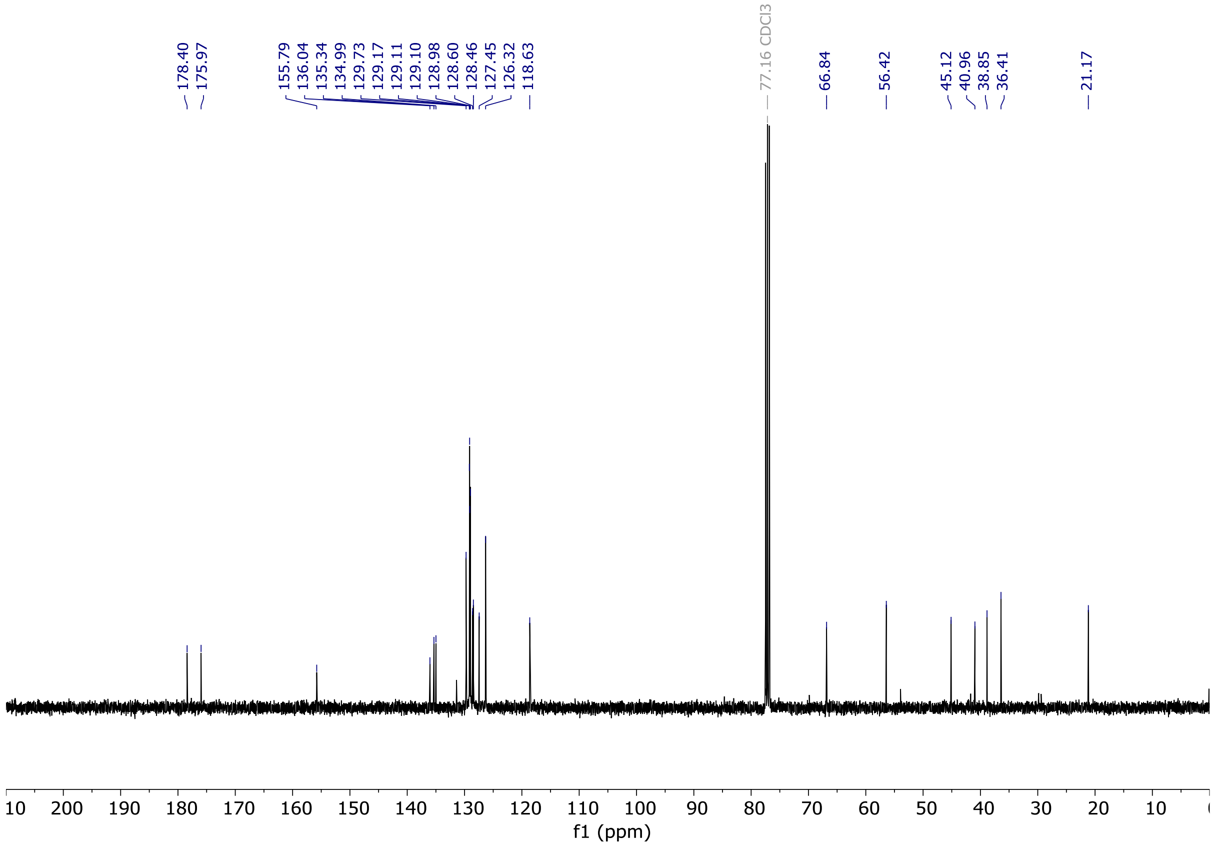

^{1}H NMR (400 MHz, $CDCl_3$)

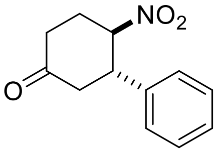

63

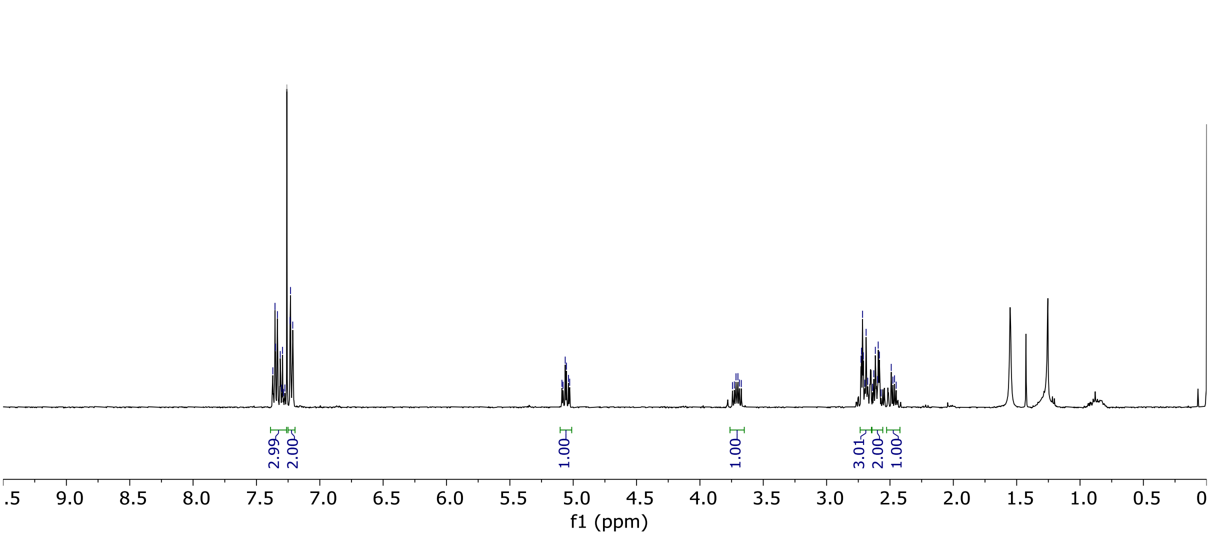

^{13}C {1H} NMR (101 MHz, $CDCl_3$)

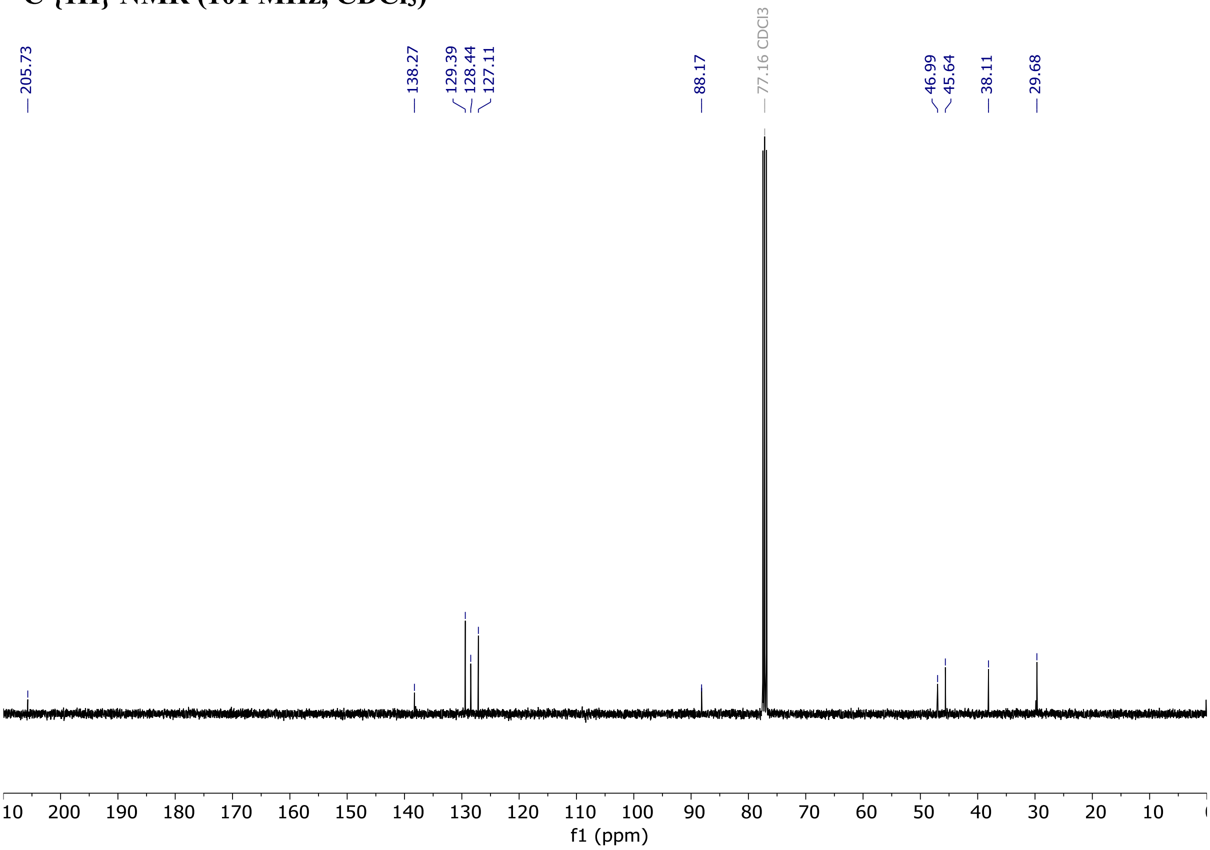

^{1}H NMR (400 MHz, $CDCl_3$)

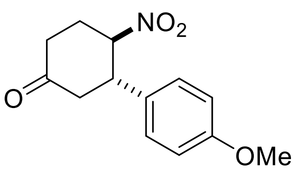

64

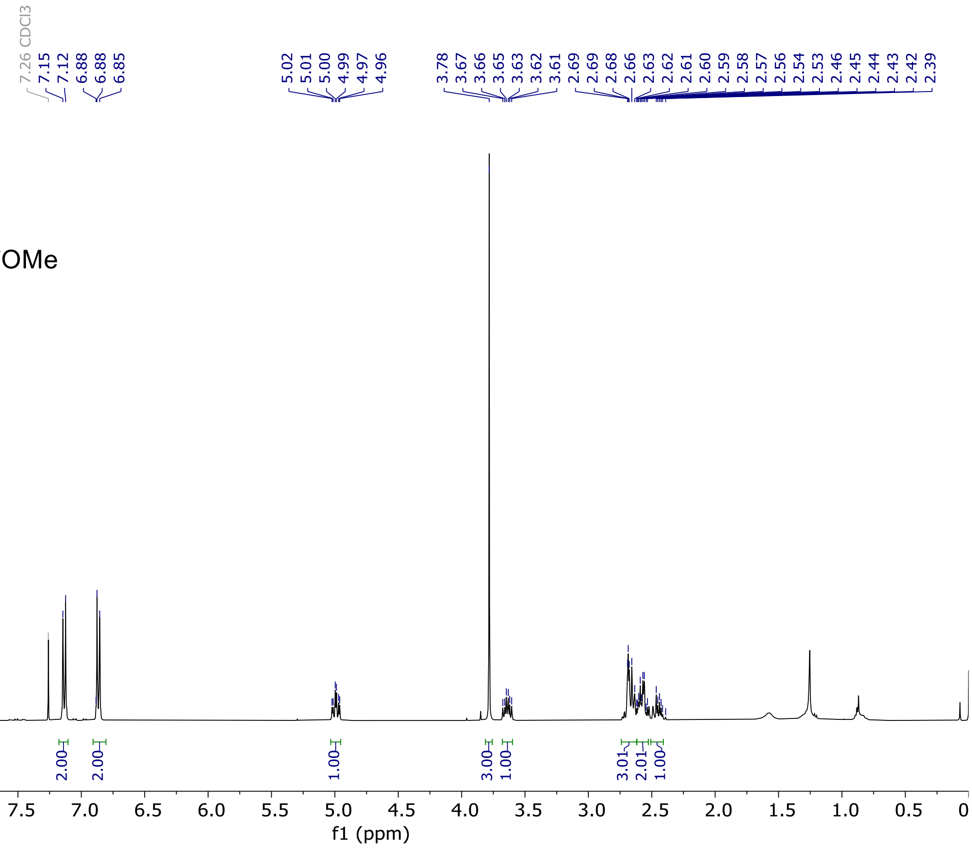

^{13}C {1H} NMR (126 MHz, $CDCl_3$)

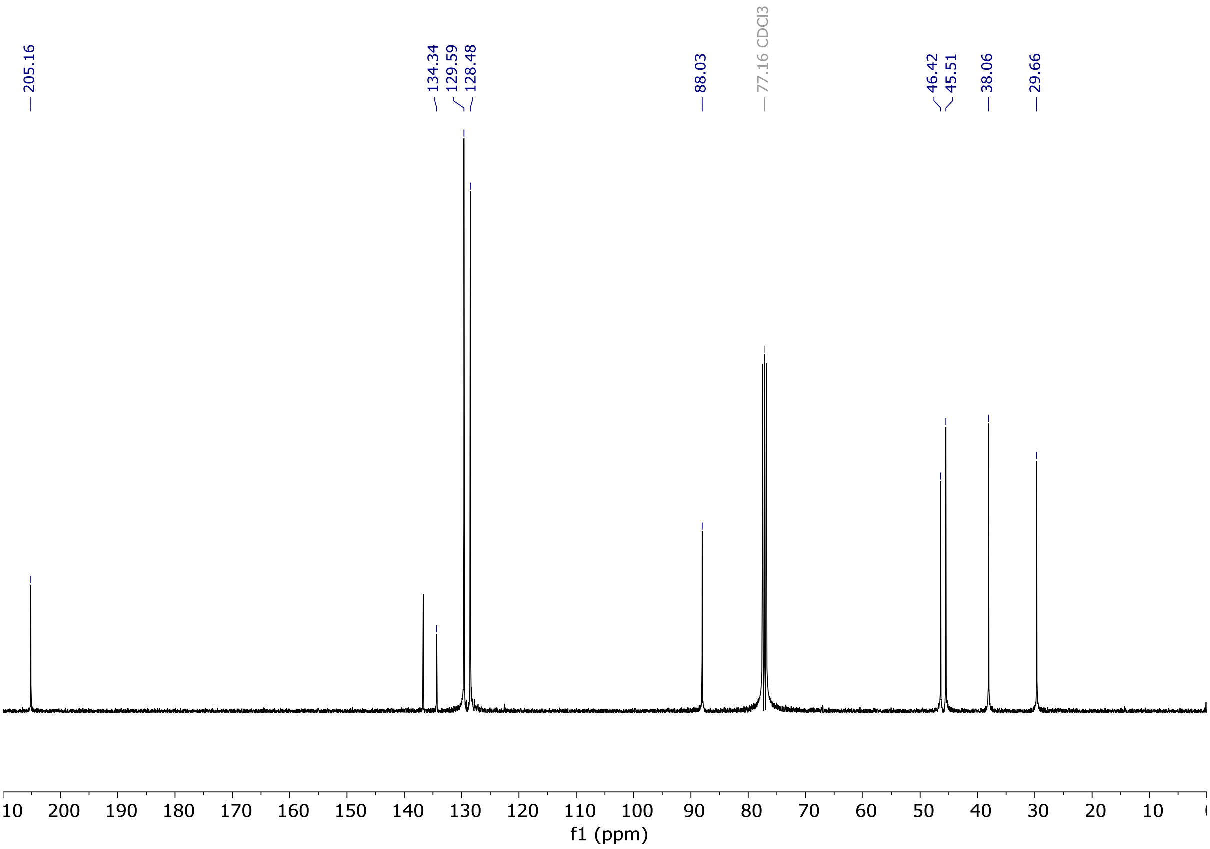

^{1}H NMR (400 MHz, $CDCl_3$)

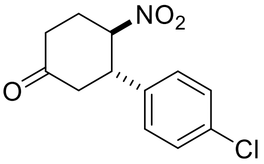

65

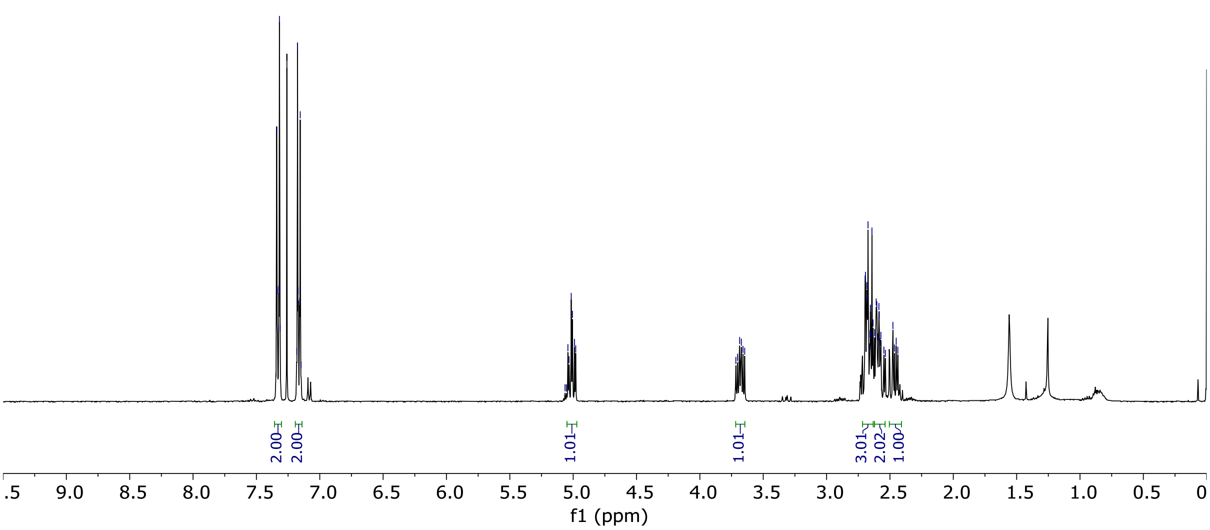

^{13}C {1H} NMR (126 MHz, $CDCl_3$)

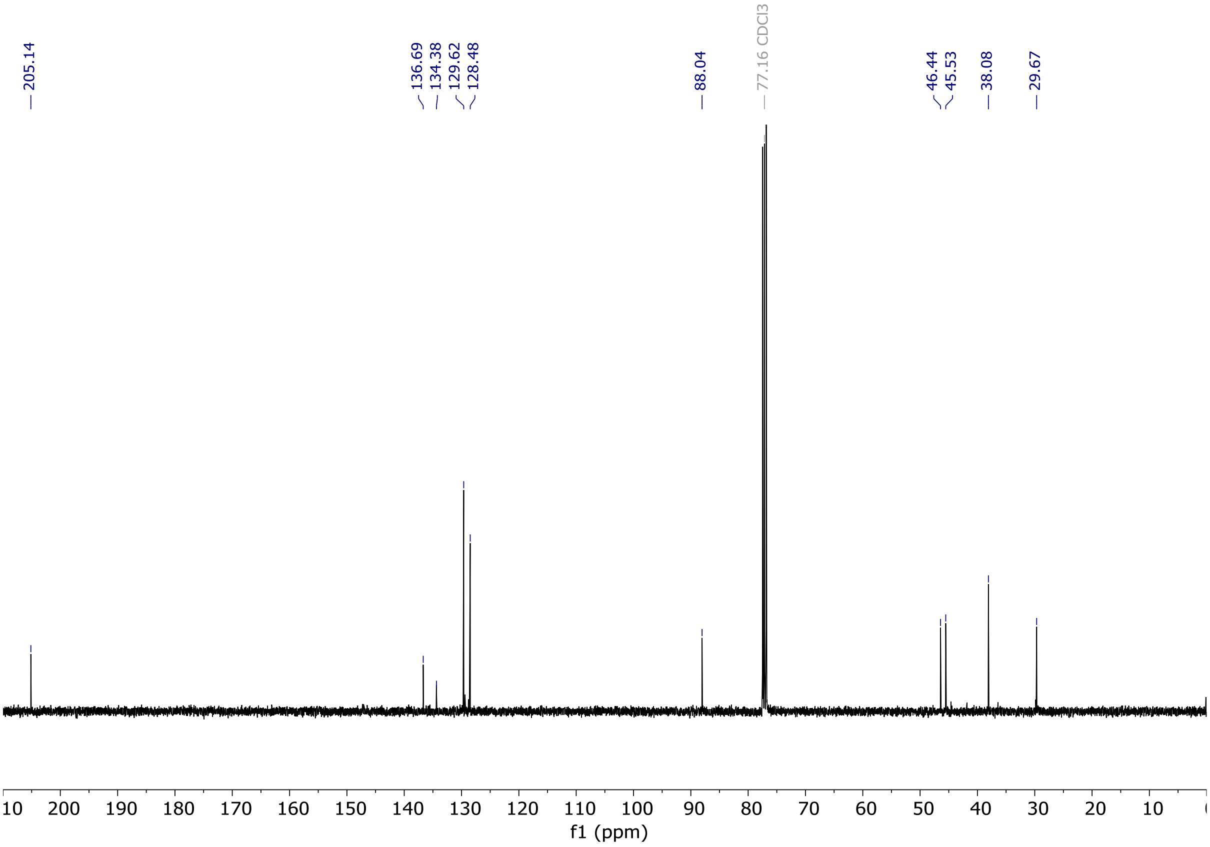

1H NMR (400 MHz, *d*-DMSO)

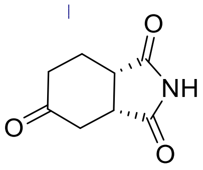

66

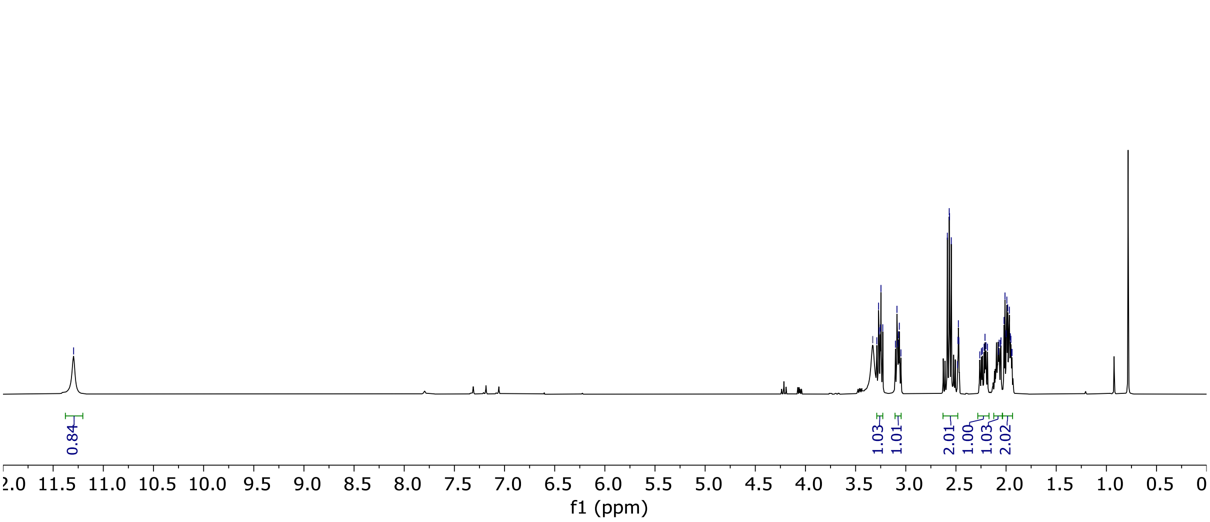

13C {1H} NMR (101 MHz, *d*-DMSO)

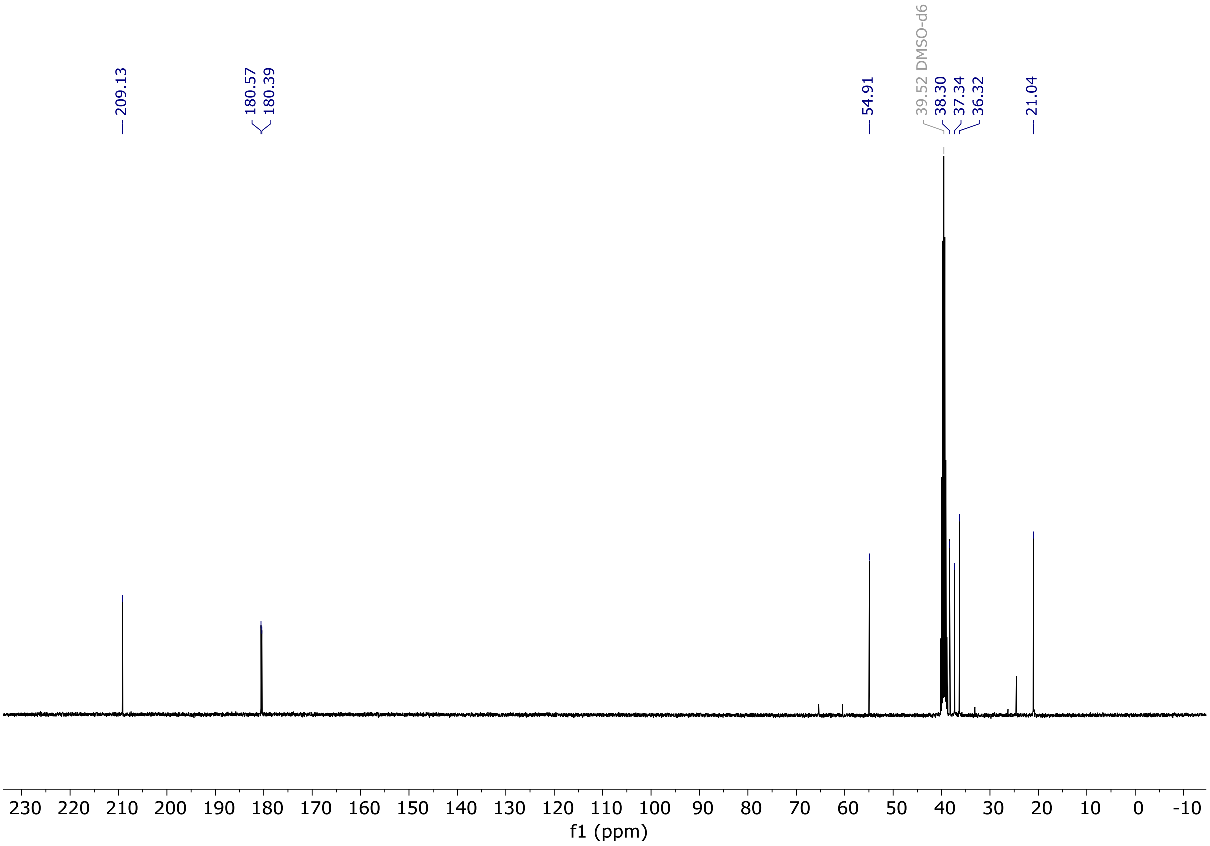

^{1}H NMR (500 MHz, *d*-DMSO)

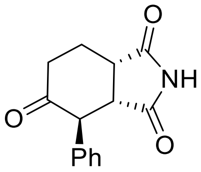

67/68

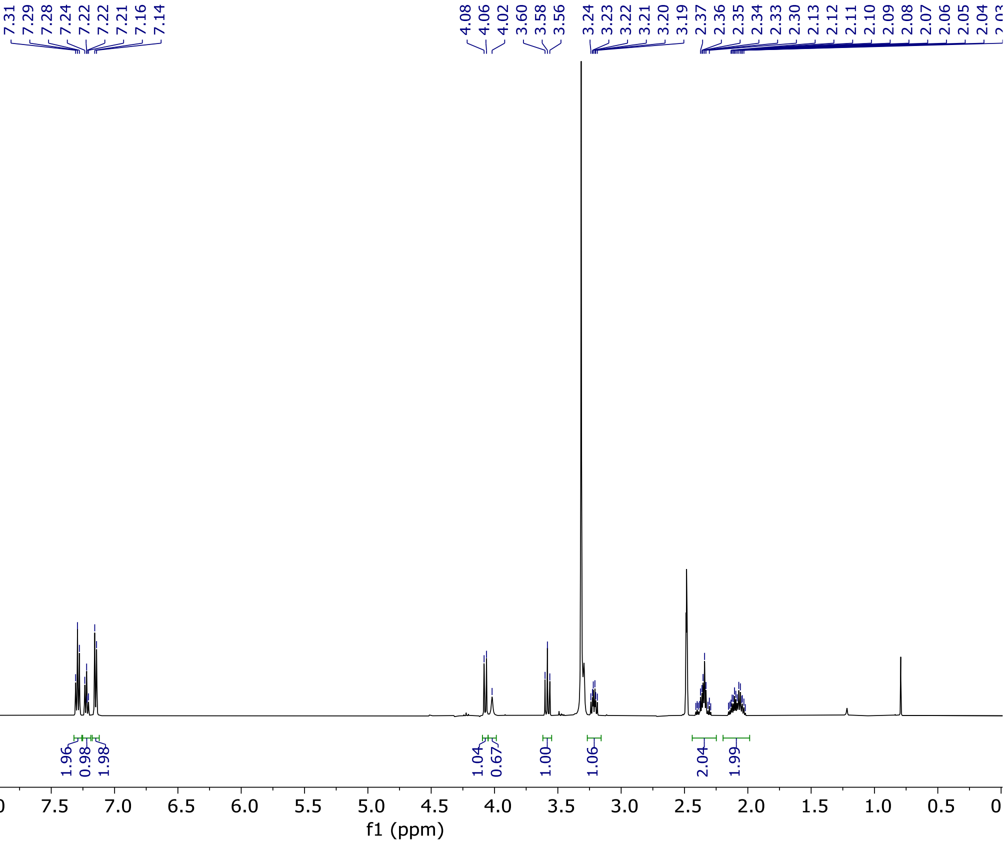

^{13}C {1H} NMR (101 MHz, *d*-DMSO)

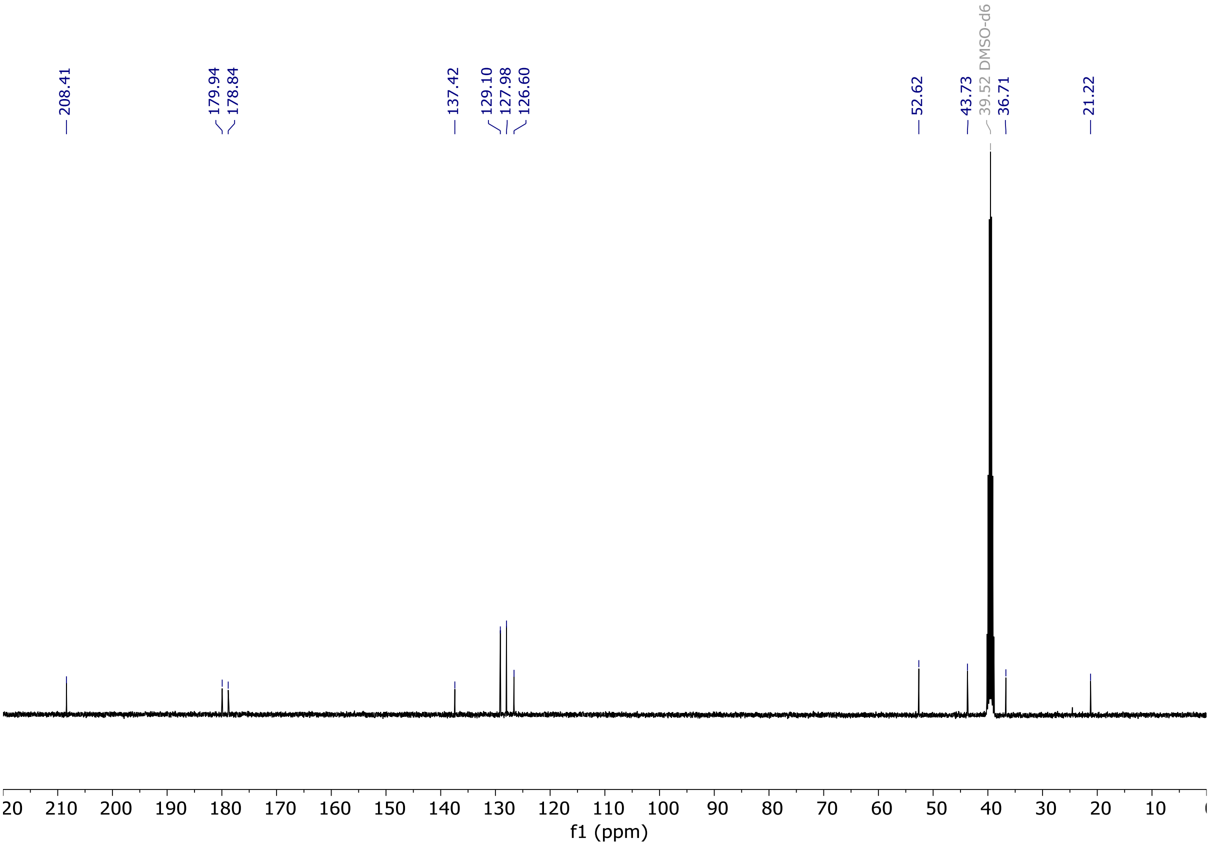

^{1}H NMR (400 MHz, $CDCl_3$)

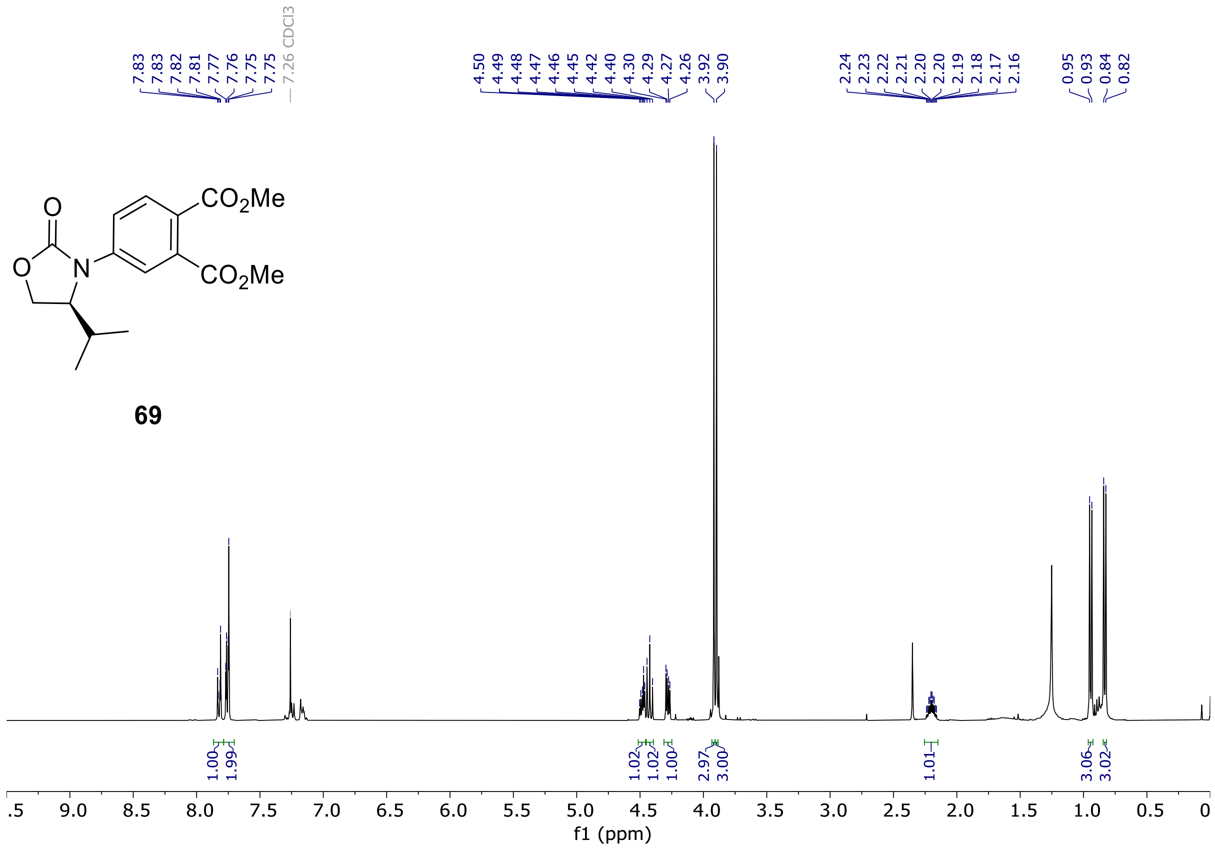

^{13}C {1H} NMR (101 MHz, $CDCl_3$)

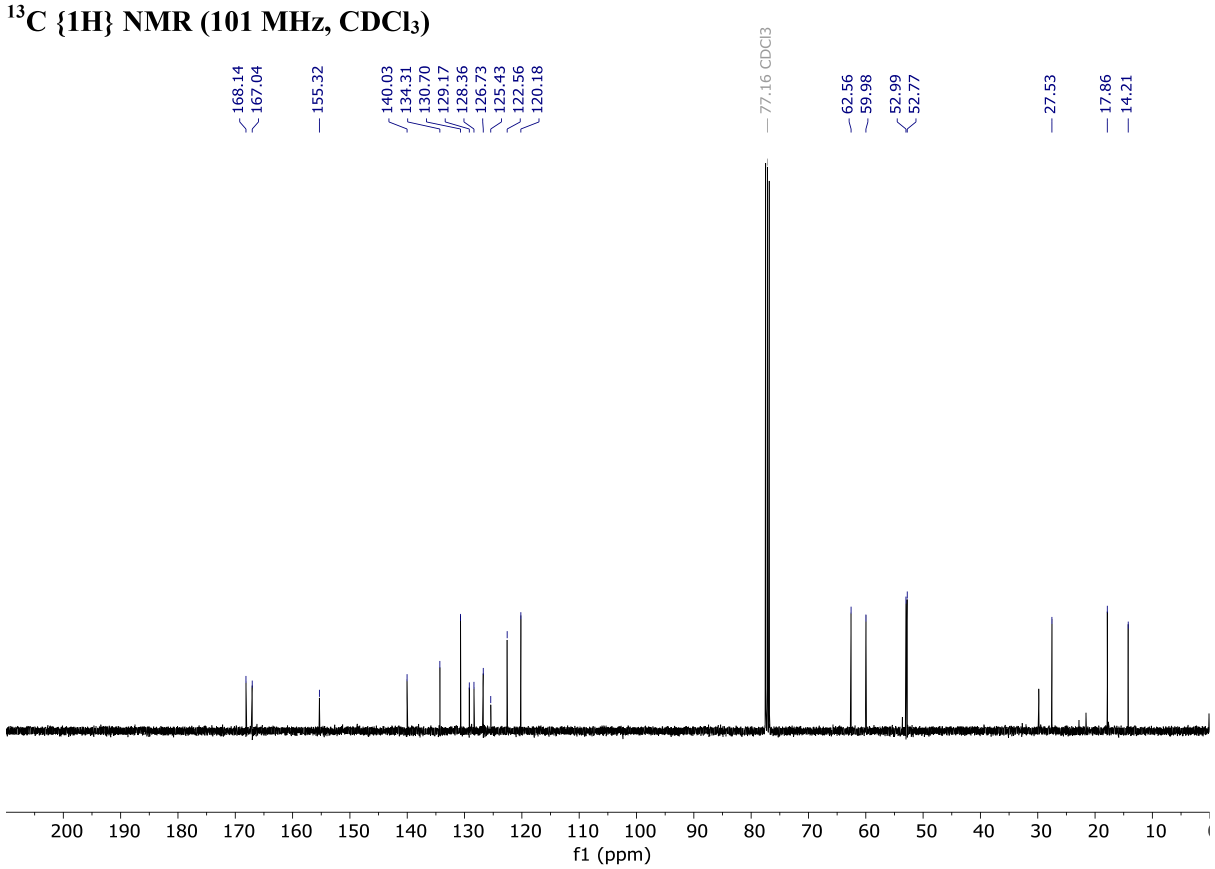

X-Ray Crystal Data

Compound **I (5b)**:

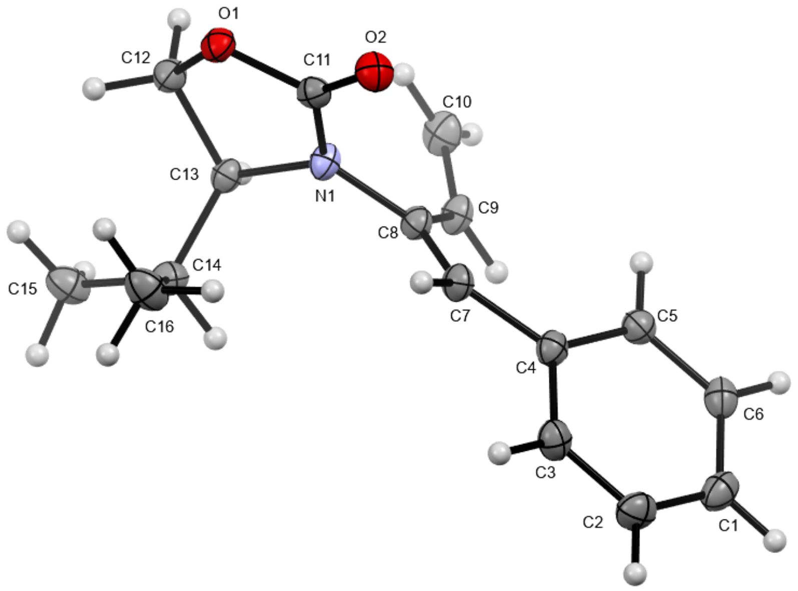

Figure S3. Asymmetric unit, showing the atom-labelling scheme. Displacement ellipsoids are drawn at the 50% probability level.

Table S1. Crystal Data and details of the structure determination.

**Crystal Data**		
Formula		C16 H19 N O2
Formula Weight		257.32
Crystal System		Orthorhombic
Space group		P212121 (No. 19)
a, b, c [Å]		7.6374(1) 10.0695(1) 17.7903(3)
alpha, beta, gamma [°]		90 90 90
V [Å^3]		1368.16(3)
Z		4
D(calc) [g/cm^3]		1.249
Mu(CuKα) [/mm]		0.653
F(000)		552
Crystal Size [mm]		0.05 x 0.13 x 0.15
**Data Collection**		
Temperature (K)		102
Radiation [Angstrom] CuKα		CuKa 1.54184
Theta Min-Max [°]		5.0, 72.5
Dataset		-9: 9 ; -12: 12 ; -22: 19
Tot., Uniq. Data, R(int)		10929, 2677, 0.026
Observed Data [I>0.0 sigma(I)]		2529
**Refinement**		
Nref, Npar		2677, 176
R, wR2, S		0.0266, 0.0661, 1.11
Max. and Av. Shift/Error	0.00, 0.00	
Flack x	0.0(2)	

Min. and Max. Resd. Dens. [e/ Å^3]	-0.12, 0.18

Compound **II (6b)**

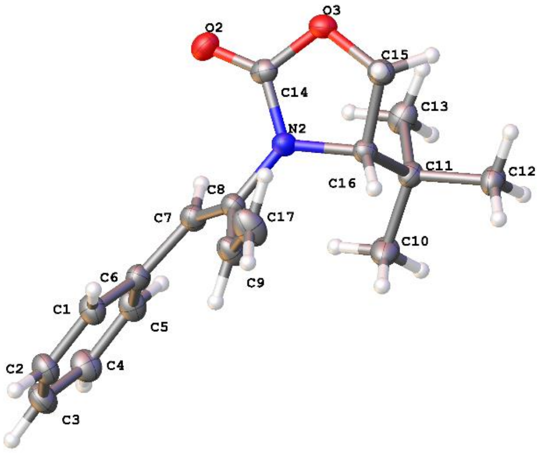

Figure S4: Asymmetric unit, showing the atom-labelling scheme. Displacement ellipsoids are drawn at the 50% probability level.

Table S2. Crystal Data and details of the structure determination.

**Empirical formula**	$C_{17}H_{21}NO_2$
**Formula weight**	271.35
**Temperature [K]**	104.55(10)
**Crystal system**	orthorhombic
**Space group (number)**	P212121 (19)
***a* [Å]**	8.25900(12)
***b* [Å]**	10.47778(13)
***c* [Å]**	17.1960(2)
**α [°]**	90
**β [°]**	90
**γ [°]**	90
**Volume [Å^3]**	1488.07(3)
Z	4
**ρ_{calc} [gcm^{-3}]**	1.211
**μ [mm^{-1}]**	0.624
***F*(000)**	584
**Crystal size [mm^3]**	0.259×0.154×0.125
**Crystal colour**	translucent light colourless
**Crystal shape**	block
**Radiation**	Cu K_α (λ=1.54184 Å)
**2θ range [°]**	9.89 to 150.52 (0.80 Å)
**Index ranges**	$-10 \le h \le 9$<br>$-13 \le k \le 13$<br>$-21 \le l \le 21$
**Reflections collected**	23568
**Independent reflections**	3037<br>R_{int} = 0.0333<br>R_{sigma} = 0.0157
**Completeness to θ = 67.684°**	100.0 %
**Data / Restraints / Parameters**	3037/0/192
**Goodness-of-fit on F^2**	1.088
**Final *R* indexes [$I \ge 2\sigma(I)$]**	R_1 = 0.0279<br>wR_2 = 0.0690

**Final *R* indexes [all data]**	R_1 = 0.0306<br>wR_2 = 0.0718
**Largest peak/hole [eÅ^{-3}]**	0.12/-0.17
**Flack X parameter**	-0.08(7)

Compound **III (7a)**:

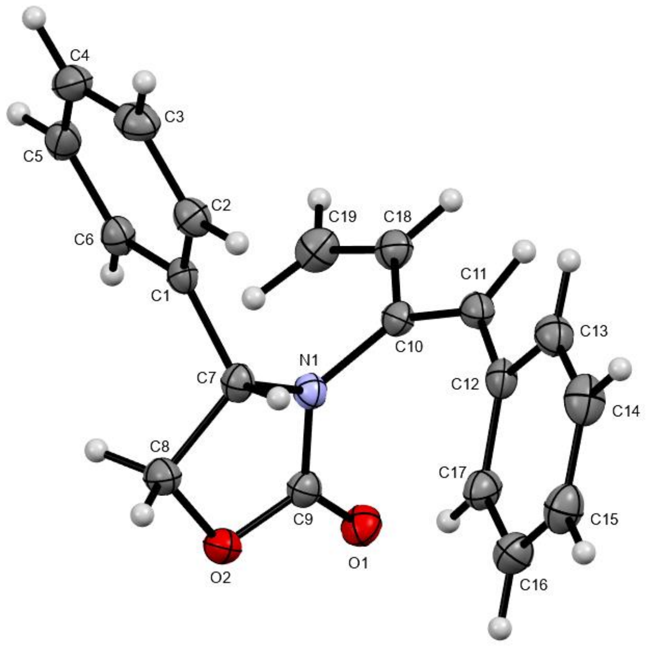

Figure S5. Asymmetric unit, showing the atom-labelling scheme. Displacement ellipsoids are drawn at the 50% probability level.

Table S3. Crystal Data and details of the structure determination.

**Crystal Data**	
Formula	C19 H17 N O2
Formula Weight	291.33
Crystal System	Orthorhombic
Space group	P212121 (No. 19)
a, b, c [Å]	7.8702(1) 11.6561(1) 16.5019(1)
alpha, beta, gamma [°]	90 90 90
V [$Å^3$]	1513.82(2)
Z	4
D(calc) [g/cm^3]	1.278
Mu(CuKα) [/mm]	0.661
F(000)	616
Crystal Size [mm]	0.12 x 0.17 x 0.19
**Data Collection**	
Temperature (K)	101
Radiation [Angstrom] CuKα	CuKα 1.54184
Theta Min-Max [º]	4.6, 74.6
Dataset	-9: 9 ; -14: 14 ; -20: 20
Tot., Uniq. Data, R(int)	48356, 3077, 0.057
Observed Data [I>0.0 sigma(I)]	2986
**Refinement**	
Nref, Npar	3077, 207
R, wR2, S	0.0250, 0.0622, 1.07
Max. and Av. Shift/Error	0.00, 0.00
Flack x	-0.08(6)
Min. and Max. Resd. Dens. [e/ $Å^3$]	-0.14, 0.12

Compound **IV (18)**:

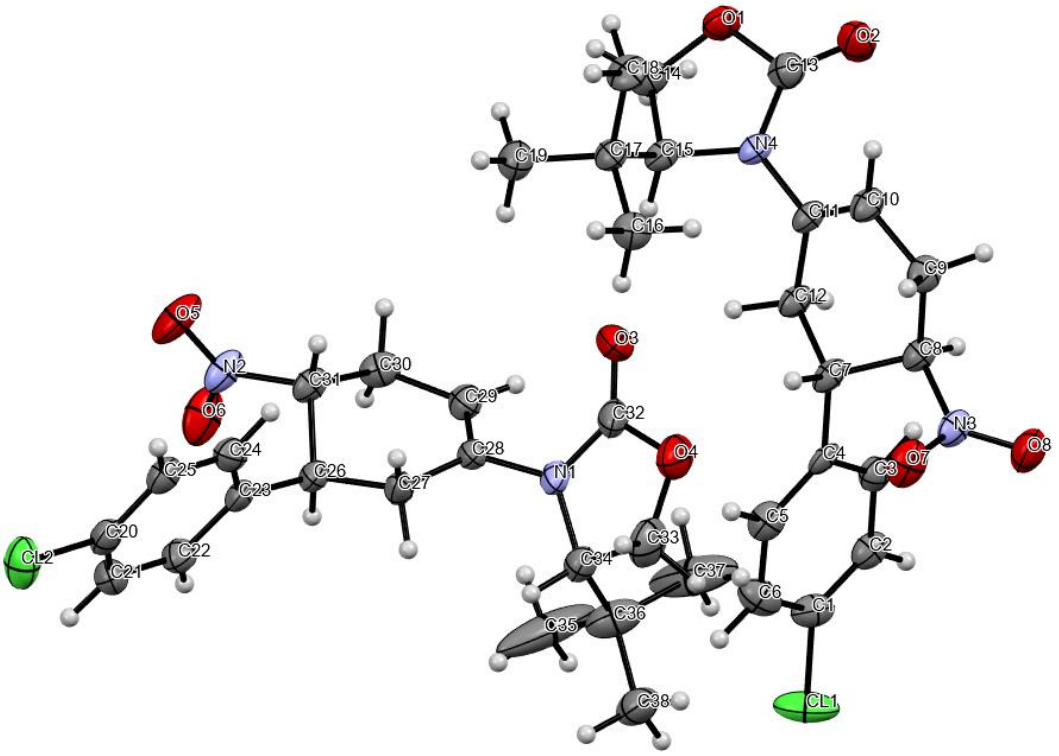

Figure S6. Asymmetric unit, showing the atom-labelling scheme. Displacement ellipsoids are drawn at the 50% probability level.

Table S4. Crystal Data and details of the structure determination.

**Crystal Data**	
Formula	C19 H23 Cl N2 O4
Formula Weight	378.84
Crystal System	monoclinic
Space group	P21 (No. 4)
a, b, c [Å]	11.4251(2) 12.4987(3) 13.6362(3)
alpha, beta, gamma [°]	90 91.2673(19) 90
V [Å^3]	1946.76(7)
Z	4
D(calc) [g/cm^3]	1.293
Mu(CuKα) [/mm]	1.957
F(000)	800
Crystal Size [mm]	0.07 x 0.14 x 0.27
**Data Collection**	
Temperature (K)	103
Radiation [Angstrom] CuKα	CuKa 1.54184
Theta Min-Max [º]	3.2, 76.5
Dataset	-14: 13 ; -15: 15 ; -14: 17
Tot., Uniq. Data, R(int)	21139, 7665, 0.032
Observed Data [I>0.0 sigma(I)]	7078
**Refinement**	

Nref, Npar	7665, 475
R, wR2, S	0.0688, 0.2185, 1.10
Max. and Av. Shift/Error	0.00, 0.00
Flack x	0.012(10)
Min. and Max. Resd. Dens. [e/ Å^3]	-0.44, 0.71

Compound **VII (27)**:

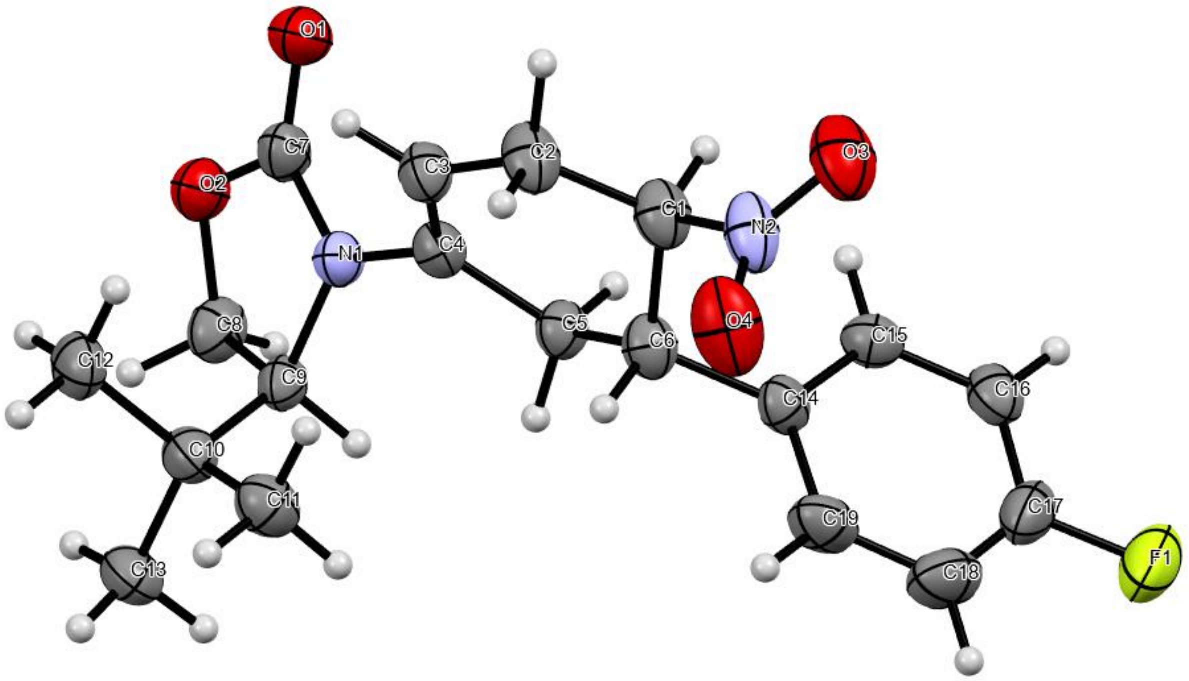

Figure S7. Asymmetric unit, showing the atom-labelling scheme. Displacement ellipsoids are drawn at the 50% probability level.

Table S5. Crystal Data and details of the structure determination.

**Empirical formula**	$C_{19}H_{23}FN_2O_4$
**Formula weight**	362.39
**Temperature [K]**	104.6(4)
**Crystal system**	orthorhombic
**Space group (number)**	P212121 (19)
***a* [Å]**	11.4030(3)
***b* [Å]**	13.0245(4)
***c* [Å]**	12.6975(4)
**α [°]**	90
**β [°]**	90
**γ [°]**	90
**Volume [Å^3]**	1885.81(10)
Z	4
**ρ_{calc} [gcm^{-3}]**	1.276
**μ [mm^{-1}]**	0.802
***F*(000)**	768
**Crystal size [mm^3]**	0.45×0.24×0.17
**Crystal colour**	translucent light colourless
**Crystal shape**	block
**Radiation**	Cu K_α (λ=1.54184 Å)
**2θ range [°]**	9.73 to 145.75 (0.81 Å)
**Index ranges**	$-14 \leq h \leq 14$<br>$-16 \leq k \leq 16$<br>$-15 \leq l \leq 15$
**Reflections collected**	24230

**Independent reflections**	3729<br>R_{int} = 0.0909<br>R_{sigma} = 0.0385
**Completeness to θ = 67.684°**	100.0 %
**Data / Restraints / Parameters**	3729/0/239
**Goodness-of-fit on F^2**	1.070
**Final *R* indexes [I≥2σ(I)]**	R_1 = 0.0758<br>wR_2 = 0.2085
**Final *R* indexes [all data]**	R_1 = 0.0780<br>wR_2 = 0.2116
**Largest peak/hole [eÅ^{-3}]**	0.45/-0.30
**Flack X parameter**	0.14(14)
**Extinction coefficient**	0.0066(16)

Compound **V** **(28)**:

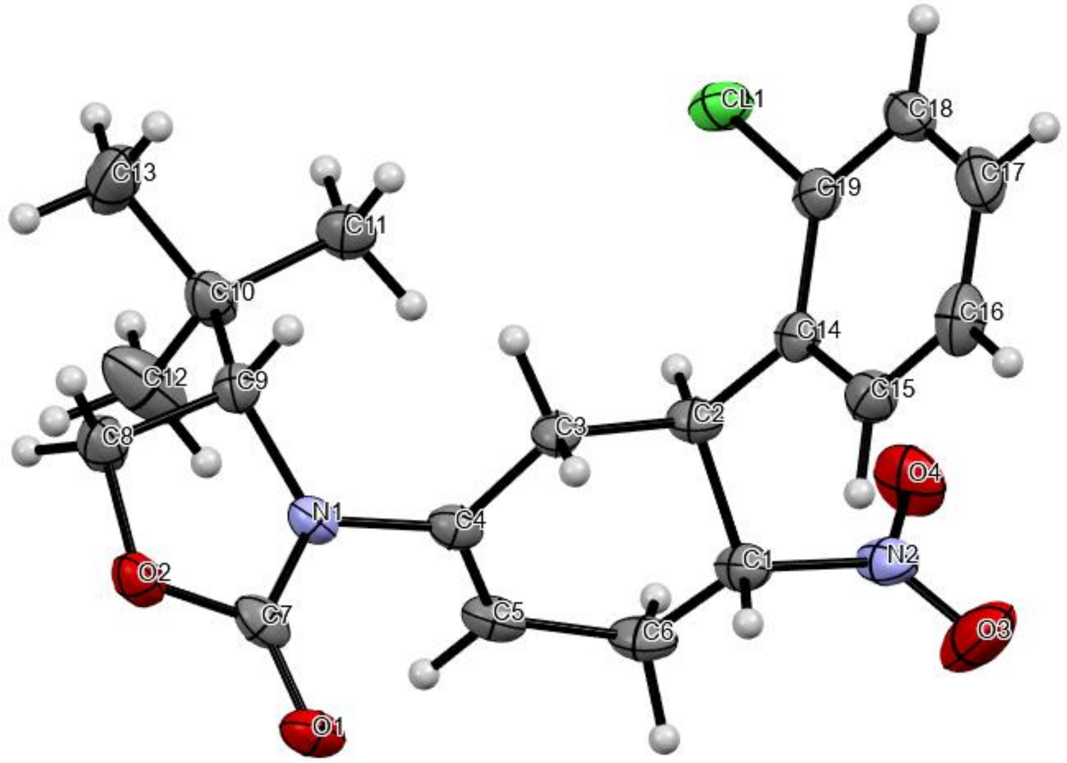

Figure S8. Asymmetric unit, showing the atom-labelling scheme. Displacement ellipsoids are drawn at the 50% probability level.

Table S6. Crystal Data and details of the structure determination.

**Empirical formula**	$C_{19}H_{23}ClN_2O_4$
**Formula weight**	378.84
**Temperature [K]**	104.90(14)
**Crystal system**	orthorhombic
**Space group (number)**	P212121 (19)
a **[Å]**	10.14492(18)
b **[Å]**	12.27747(19)
c **[Å]**	14.9814(2)
**α [°]**	90
**β [°]**	90
**γ [°]**	90
**Volume [Å³]**	1865.99(5)
Z	4
**ρ_{calc} [gcm^{-3}]**	1.349
**μ [mm^{-1}]**	2.042
***F*(000)**	800
**Crystal size [mm³]**	0.285×0.247×0.185
**Crystal colour**	translucent intense colourless
**Crystal shape**	block
**Radiation**	Cu K_α (λ=1.54184 Å)
**2θ range [°]**	9.31 to 145.34 (0.81 Å)

**Index ranges**	$-12 \leq h \leq 12$<br>$-15 \leq k \leq 15$<br>$-18 \leq l \leq 18$
**Reflections collected**	21036
**Independent reflections**	3680<br>R_{int} = 0.0432<br>R_{sigma} = 0.0243
**Completeness to θ = 67.684°**	100.0 %
**Data / Restraints / Parameters**	3680/0/238
**Goodness-of-fit on F^2**	1.018
**Final *R* indexes [*I*≥2σ(*I*)]**	R_1 = 0.0371<br>wR_2 = 0.0928
**Final *R* indexes [all data]**	R_1 = 0.0400<br>wR_2 = 0.0948
**Largest peak/hole [eÅ^{-3}]**	0.69/-0.34
**Flack X parameter**	0.001(8)

Compound **VI (31)**:

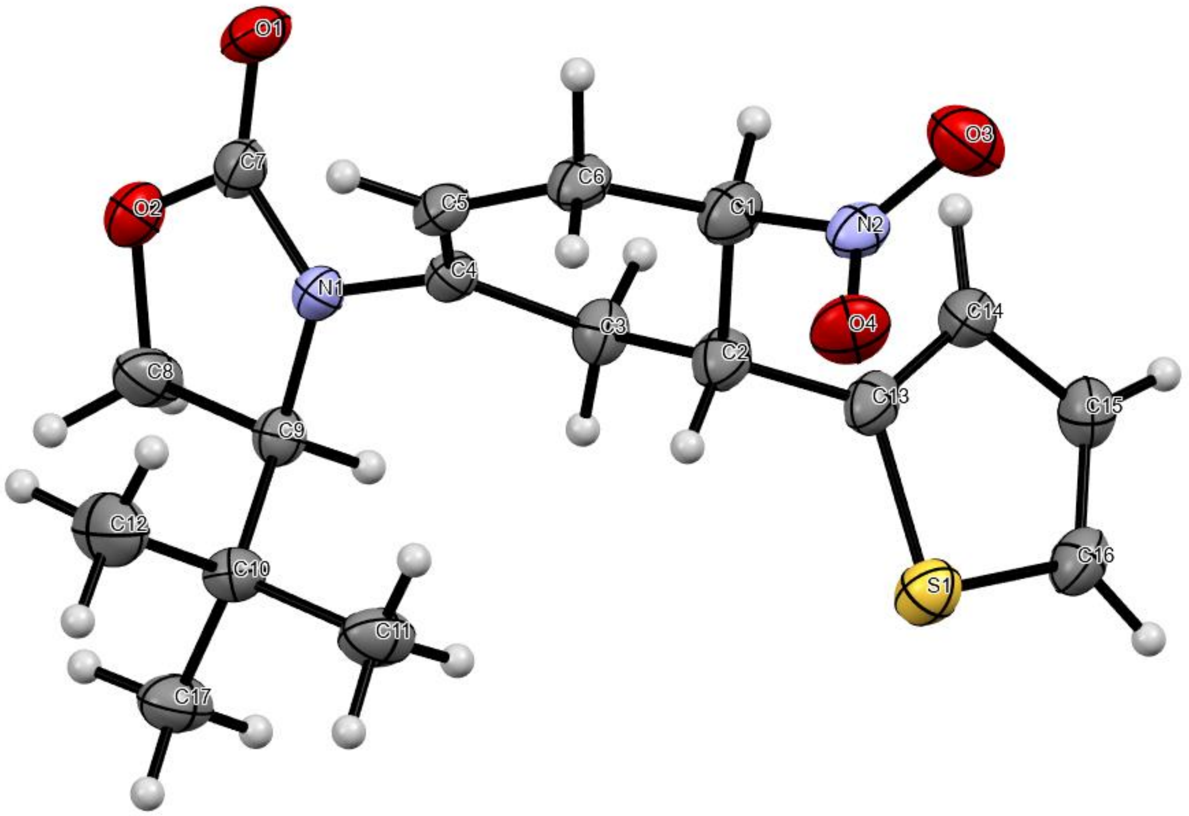

Figure S9. Asymmetric unit, showing the atom-labelling scheme. Displacement ellipsoids are drawn at the 50% probability level.

Table S7. Crystal Data and details of the structure determination.

**Empirical formula**	$C_{17}H_{22}N_2O_4S$
**Formula weight**	350.42
**Temperature [K]**	117(18)
**Crystal system**	orthorhombic
**Space group (number)**	P212121 (19)
***a* [Å]**	10.0719(2)
***b* [Å]**	11.46842(19)
***c* [Å]**	14.7735(3)
**α [°]**	90
**β [°]**	90
**γ [°]**	90
**Volume [Å³]**	1706.47(6)
Z	4
**ρ_{calc} [gcm⁻³]**	1.364
**μ [mm⁻¹]**	1.893
***F*(000)**	744
**Crystal size [mm³]**	0.3×0.21×0.16
**Crystal colour**	clear light colourless
**Crystal shape**	block

**Radiation**	Cu K_α (λ=1.54184 Å)
**2θ range [°]**	9.76 to 145.50 (0.81 Å)
**Index ranges**	−12 ≤ h ≤ 11<br>−14 ≤ k ≤ 14<br>−18 ≤ l ≤ 18
**Reflections collected**	14990
**Independent reflections**	3343<br>R_{int} = 0.0540<br>R_{sigma} = 0.0334
**Completeness to θ = 67.684°**	100.0 %
**Data / Restraints / Parameters**	3343/0/220
**Goodness-of-fit on F^2**	1.034
**Final R indexes [I≥2σ(I)]**	R_1 = 0.0403<br>wR_2 = 0.1044
**Final R indexes [all data]**	R_1 = 0.0437<br>wR_2 = 0.1082
**Largest peak/hole [eÅ^{-3}]**	0.55/-0.40
**Flack X parameter**	0.010(12)

Compound **VIII (34):**

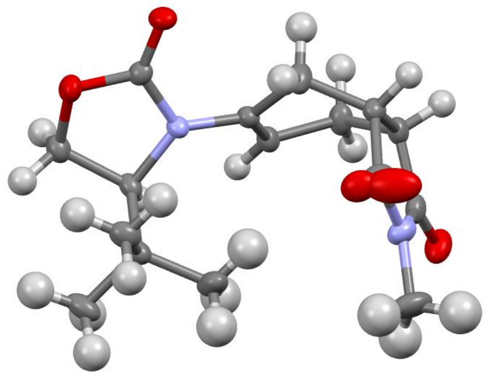

Figure S10. Asymmetric unit, showing the atom-labelling scheme. Displacement ellipsoids are drawn at the 50% probability level.

Table S8. Crystal Data and details of the structure determination.

**Empirical formula**	C16H22N2O4
**Formula weight**	396.43
**Temperature [K]**	110 (7)
**Crystal system**	orthorhombic
**Space group (number)**	(19)
**a [Å]**	6.2659(2)
**b [Å]**	9.8128(3)
**c [Å]**	25.6841(6)
**α [°]**	90
**β [°]**	90
**γ [°]**	90
**Volume [Å3]**	1579.21(8)
**Z**	4
**ρcalc [gcm−3]**	1.289
**μ [mm−1]**	0.764
**F(000)**	656
**Crystal size [mm3]**	0.27×0.07×0.05
**Crystal colour**	translucent light colourless
**Crystal shape**	Block
**Radiation**	CuKα (λ=1.54184 Å)
**2θ range [°]**	6.33 to 152.83 (0.79 Å)
**Index ranges**	−7 ≤ h ≤ 7<br>−12 ≤ k ≤ 12<br>−32 ≤ l ≤ 32
**Reflections collected**	19012
**Independent reflections**	3282<br>Rint = 0.0665<br>Rsigma = 0.0367
**Completeness to θ = 67.684°**	100.0 %
**Data / Restraints / Parameters**	3282/0/214
**Goodness-of-fit on F2**	1.040

**Final R indexes [I≥2σ(I)]**	R1 = 0.0393<br>wR2 = 0.0946
**Final R indexes [all data]**	R1 = 0.0433<br>wR2 = 0.0974
**Largest peak/hole [eÅ−3]**	0.26/-0.20
**Flack X parameter**	-0.20(12)

Compound **IX (38):**

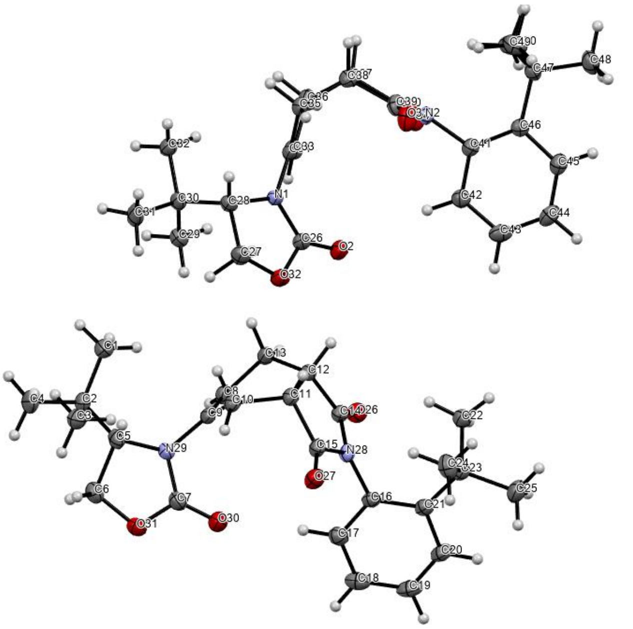

Figure S11. Asymmetric unit, showing the atom-labelling scheme. Displacement ellipsoids are drawn at the 50% probability level.

Table S9. Crystal Data and details of the structure determination.

**Empirical formula**	C25H32N2O4
**Formula weight**	424.52
**Temperature [K]**	104.15
**Crystal system**	monoclinic
**Space group (number)**	P21 (4)
**a [Å]**	8.05790(10)
**b [Å]**	27.9208(5)
**c [Å]**	10.3433(2)
**α [°]**	90
**β [°]**	108.520(2)
**γ [°]**	90
**Volume [Å3]**	2206.55(7)
**Z**	4
**ρcalc [gcm−3]**	1.278
**μ [mm−1]**	0.694
**F(000)**	912
**Crystal size [mm3]**	0.164×0.062×0.03
**Crystal colour**	translucent intense colourless
**Crystal shape**	block
**Radiation**	CuKα (λ=1.54184 Å)
**2θ range [°]**	6.33 to 149.73 (0.80 Å)
**Index ranges**	−10 ≤ h ≤ 10<br>−28 ≤ k ≤ 34<br>−12 ≤ l ≤ 12
**Reflections collected**	35247
**Independent reflections**	7898<br>Rint = 0.0367<br>Rsigma = 0.0267

**Completeness to θ = 67.684°**	100.0 %
**Data / Restraints / Parameters**	7898/1/571
**Goodness-of-fit on F2**	1.045
**Final R indexes [I≥2σ(I)]**	R1 = 0.0387<br>wR2 = 0.1014
**Final R indexes [all data]**	R1 = 0.0403<br>wR2 = 0.1030
**Largest peak/hole [eÅ−3]**	0.43/-0.17
**Flack X parameter**	0.02(7)

Compound **X (45)**:

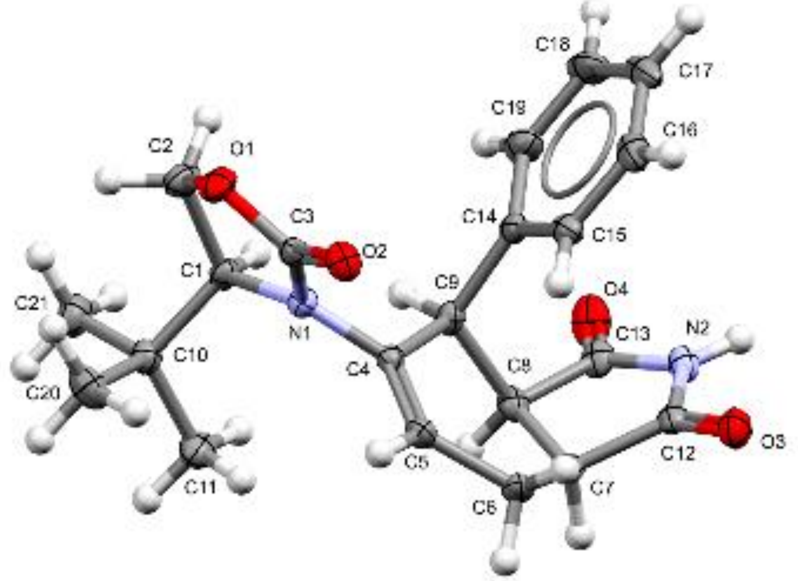

Figure S12. Asymmetric unit, showing the atom-labelling scheme. Displacement ellipsoids are drawn at the 50% probability level. Graphics were obtained using Mercury 3.0. [4]

Table S10. Crystal Data and details of the structure determination.

	**AOC296.cif**
Empirical formula	$C_{21}H_{24}N_2O_4$
Formula weight	368.42
Temperature [K]	105(3)
Crystal system	orthorhombic
Space group (number)	$P2_12_12_1$ (19)
a [Å]	7.34690(10)
b [Å]	15.1203(2)
c [Å]	17.0491(2)
α [°]	90
β [°]	90
γ [°]	90
Volume [Å^3]	1893.94(4)
Z	4
ρ_{calc} [gcm^{-3}]	1.292
μ [mm^{-1}]	0.732
F(000)	784
Crystal size [mm^3]	0.27×0.25×0.18
Crystal colour	translucent light colourless
Crystal shape	block
Radiation	Cu K_α (λ=1.54184 Å)
2Θ range [°]	7.82 to 145.43 (0.81 Å)
Index ranges	$-8 \le h \le 7$<br>$-18 \le k \le 18$<br>$-20 \le l \le 20$
Reflections collected	21298

Independent reflections	3703<br>R_{int} = 0.0427<br>R_{sigma} = 0.0256
Completeness	100.0 %
Data / Restraints / Parameters	3703/0/251
Goodness-of-fit on F^2	1.076
Final R indexes [$I \geq 2\sigma(I)$]	R_1 = 0.0311<br>wR_2 = 0.0711
Final R indexes [all data]	R_1 = 0.0349<br>wR_2 = 0.0737
Largest peak/hole [eÅ^{-3}]	0.18/-0.19
Flack X parameter	-0.11(8)

Compound **XI (46)**:

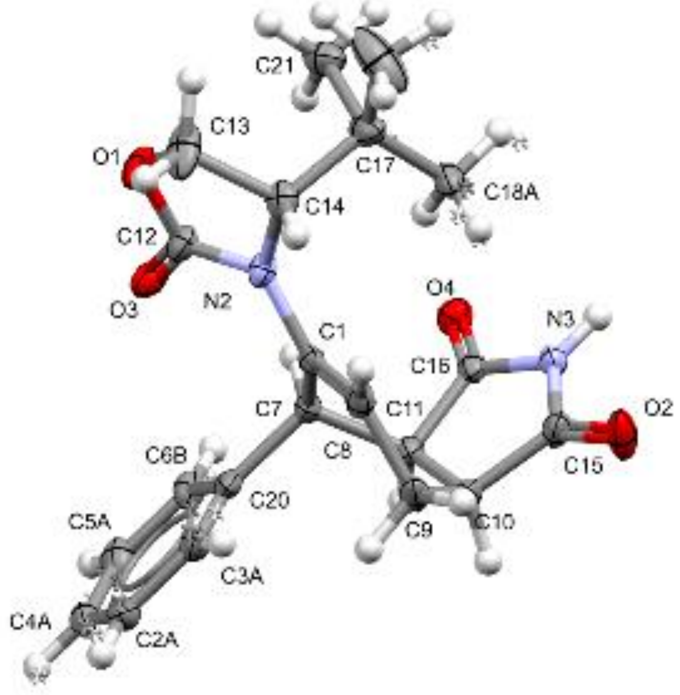

Figure S13. Asymmetric unit, showing the atom-labelling scheme. Displacement ellipsoids are drawn at the 50% probability level. Graphics were obtained using Mercury 3.0. [4]

Table S11. Crystal Data and details of the structure determination.

	**AOC418.cif**
Empirical formula	$C_{21}H_{23.99}N_2O_4$
Formula weight	368.35
Temperature [K]	129(30)
Crystal system	orthorhombic
Space group (number)	$P2_12_12_1$ (19)
a [Å]	7.29565(9)
b [Å]	9.57161(13)
c [Å]	26.1015(3)
α [°]	90
β [°]	90
γ [°]	90
Volume [Å^3]	1822.70(4)
Z	4
ρ_{calc} [gcm^{-3}]	1.342
μ [mm^{-1}]	0.760
F(000)	784
Crystal size [mm^3]	0.289×0.212×0.145
Crystal colour	translucent light colourless
Crystal shape	block
Radiation	Cu K_α (λ=1.54184 Å)
2Θ range [°]	6.77 to 145.45 (0.81 Å)

Index ranges	$-9 \leq h \leq 9$<br>$-8 \leq k \leq 11$<br>$-32 \leq l \leq 32$
Reflections collected	17001
Independent reflections	3564<br>$R_{int} = 0.0327$<br>$R_{sigma} = 0.0207$
Completeness	100.0 %
Data / Restraints / Parameters	3564/21/285
Goodness-of-fit on F^2	1.034
Final *R* indexes [$I \geq 2\sigma(I)$]	$R_1 = 0.0314$<br>$wR_2 = 0.0773$
Final *R* indexes [all data]	$R_1 = 0.0337$<br>$wR_2 = 0.0789$
Largest peak/hole [eÅ^{-3}]	0.23/-0.20
Flack X parameter	-0.03(6)

Compound **XII (63)**:

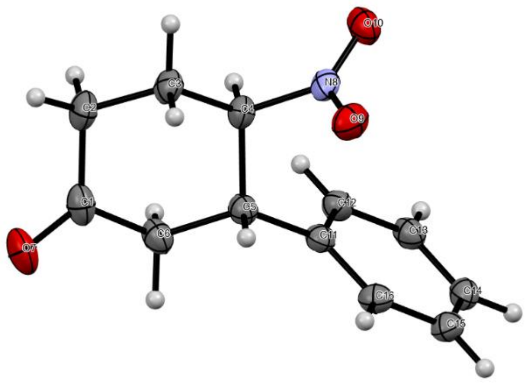

Figure S14. Asymmetric unit, showing the atom-labelling scheme. Displacement ellipsoids are drawn at the 50% probability level.

Table S12. Crystal Data and details of the structure determination.

**Crystal Data**	
Formula	C12 H13 N O3
Formula Weight	219.23
Crystal System	orthorhombic
Space group	P212121 (No. 19)
a, b, c [Å]	5.6677(1) 10.8726(3) 17.5306(5)
V [Å^3]	1080.28(5)
Z	4
D(calc) [g/cm^3]	1.348
Mu(CuKα) [/mm]	0.804
F(000)	464
Crystal Size [mm]	0.04 x 0.04 x 0.15
**Data Collection**	
Temperature (K)	105
Radiation [Angstrom] CuKα	CuKa 1.54184
Theta Min-Max [°]	4.8, 76.4
Dataset	-6: 7 ; -13: 13 ; -21: 22
Tot., Uniq. Data, R(int)	8561, 2240, 0.036
Observed Data [I>0.0 sigma(I)]	2065
**Refinement**	
Nref, Npar	2240, 145
R, wR2, S	0.0360, 0.0909, 1.06
Max. and Av. Shift/Error	0.00, 0.00
Flack x	-0.15(16)
Min. and Max. Resd. Dens. [e/ Å^3]	-0.19, 0.25

Compound **XIII (64)**:

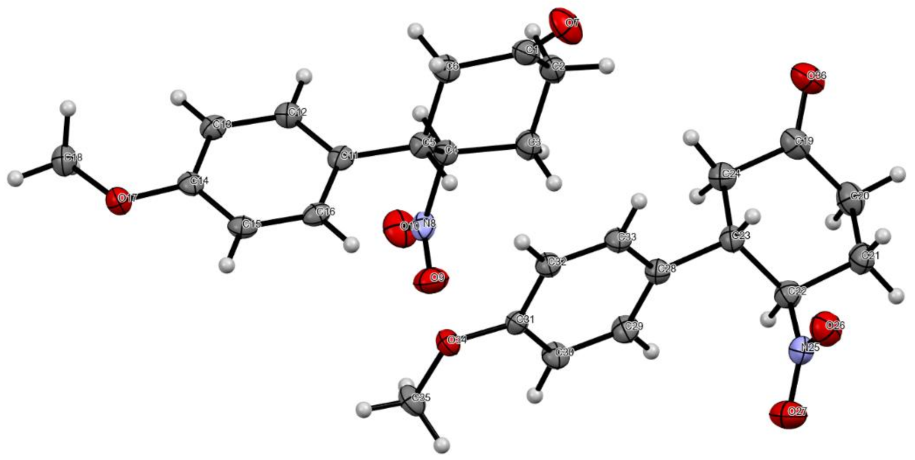

Figure S15. Asymmetric unit, showing the atom-labelling scheme. Displacement ellipsoids are drawn at the 50% probability level.

Table S13. Crystal Data and details of the structure determination.

**Crystal Data**	
Formula	C13 H15 N O4
Formula Weight	249.26
Crystal System	Orthorhombic
Space group	P212121 (No. 19)
a, b, c [Å]	8.33221(4) 9.75542(5) 29.98794(15)
V [$Å^3$]	2437.55(2)
Z	8
D(calc) [g/cm^3]	1.358
Mu(CuKα) [/mm]	0.843
F(000)	1056
Crystal Size [mm]	0.04 x 0.17 x 0.27
**Data Collection**	
Temperature (K)	103
Radiation [Angstrom] CuKα	CuKa 1.54184
Theta Min-Max [º]	2.9, 76.7
Dataset	-10: 10 ; -12: 11 ; -37: 37
Tot., Uniq. Data, R(int)	114950, 5118, 0.055
Observed Data [I>0.0 sigma(I)]	4992
**Refinement**	
Nref, Npar	5118, 328
R, wR2, S	0.0275, 0.0721, 1.02
Max. and Av. Shift/Error	0.00, 0.00
Flack x	-0.05(4)
Min. and Max. Resd. Dens. [e/ $Å^3$]	-0.14, 0.19

Compound **XIV (65)**:

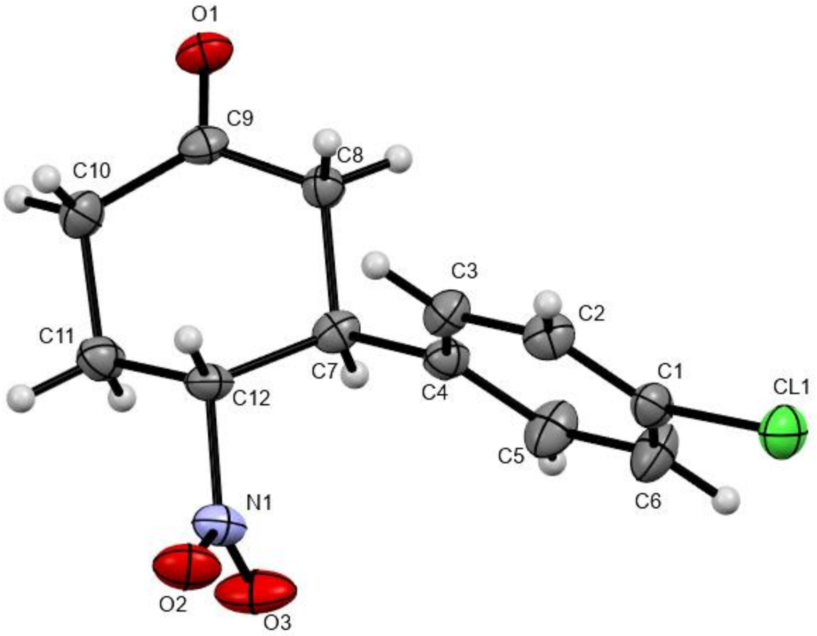

Figure S16. Asymmetric unit, showing the atom-labelling scheme. Displacement ellipsoids are drawn at the 50% probability level.

Table S14. Crystal Data and details of the structure determination.

**Crystal Data**	
Formula	C12 H12 Cl N O3
Formula Weight	253.68
Crystal System	monoclinic
Space group	P21 (No. 4)
a, b, c [Å]	10.0191(2) 5.9316(1) 10.5162(2)
alpha, beta, gamma [°]	90 99.777(2) 90
V [$Å^3$]	615.89(2)
Z	2
D(calc) [g/cm^3]	1.368
Mu(CuKα) [/mm]	2.732
F(000)	264
Crystal Size [mm]	0.08 x 0.08 x 0.20
**Data Collection**	
Temperature (K)	102
Radiation [Angstrom] CuKα	CuKα 1.54184
Theta Min-Max [°]	4.3, 72.4
Dataset	-12: 12 ; -7: 6 ; -13: 13
Tot., Uniq. Data, R(int)	11006, 2293, 0.022
Observed Data [I>0.0 sigma(I)]	2234
**Refinement**	
Nref, Npar	2293, 154
R, wR2, S	0.0230, 0.0571, 1.07
Max. and Av. Shift/Error	0.00, 0.00
Flack x	-0.017(6)
Min. and Max. Resd. Dens. [e/ $Å^3$]	-0.15, 0.17

Compound **XV (66)**:

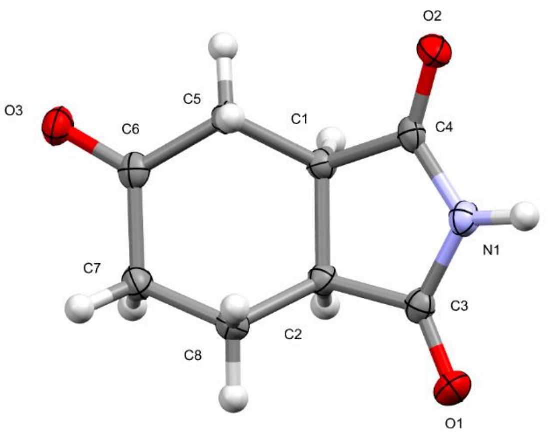

Figure S17. Asymmetric unit, showing the atom-labelling scheme. Displacement ellipsoids are drawn at the 50% probability level.

Table S15. Crystal Data and details of the structure determination.

**Empirical formula**	C8H9NO3
**Formula weight**	167.16
**Temperature [K]**	106.15
**Crystal system**	orthorhombic
**Space group (number)**	P212121 (19)
**a [Å]**	6.9165(2)
**b [Å]**	8.3865(3)
**c [Å]**	12.5734(3)
**α [°]**	90
**β [°]**	90
**γ [°]**	90
**Volume [Å3]**	729.33(4)
**Z**	4
**ρcalc [gcm−3]**	1.522
**μ [mm−1]**	0.993
**F(000)**	352
**Crystal size [mm3]**	0.328×0.125×0.105
**Crystal colour**	Translucent intense colourless
**Crystal shape**	Block
**Radiation**	Cu Kα (λ=1.54184 Å)
**2θ range [°]**	12.69 to 145.20 (0.80 Å)
**Index ranges**	$-8 \le h \le 8$<br>$-10 \le k \le 10$<br>$-15 \le l \le 15$
**Reflections collected**	8076
**Independent reflections**	1439<br>Rint = 0.0709<br>Rsigma = 0.0338
**Completeness to θ = 67.684°**	100.0 %
**Data / Restraints / Parameters**	1439/0/114

**Goodness-of-fit on F2**	1.055
**Final R indexes [I≥2σ(I)]**	R1 = 0.0398<br>wR2 = 0.1021
**Final R indexes [all data]**	R1 = 0.0417<br>wR2 = 0.1051
**Largest peak/hole [eÅ−3]**	0.23/-0.22
**Flack X parameter**	0.0(3)

:

Compound **XVI (67)**:

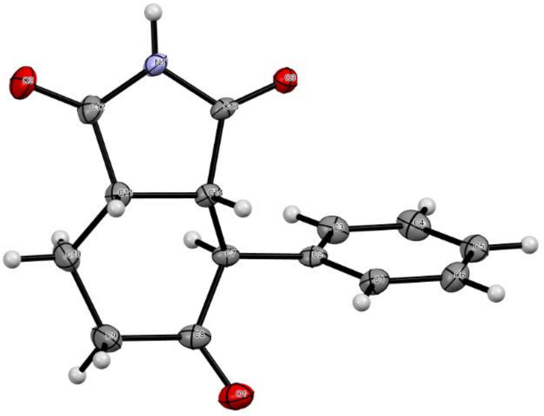

Figure S18. Asymmetric unit, showing the atom-labelling scheme. Displacement ellipsoids are drawn at the 50% probability level.

Table S16. Crystal Data and details of the structure determination.

**Empirical formula**	C14H13NO3
**Formula weight**	243.25
**Temperature [K]**	104.3(2)
**Crystal system**	orthorhombic
**Space group (number)**	P212121 (19)
**a [Å]**	6.04700(10)
**b [Å]**	11.6867(3)
**c [Å]**	16.4032(3)
**α [°]**	90
**β [°]**	90
**γ [°]**	90
**Volume [Å3]**	1159.21(4)
**Z**	4
**ρcalc [gcm−3]**	1.394
**μ [mm−1]**	0.812
**F(000)**	512
**Crystal size [mm3]**	0.414×0.144×0.111
**Crystal colour**	clear light colourless
**Crystal shape**	block
**Radiation**	Cu Kα (λ=1.54184 Å)
**2θ range [°]**	9.29 to 148.86 (0.80 Å)
**Index ranges**	−6 ≤ h ≤ 7<br>−12 ≤ k ≤ 14<br>−19 ≤ l ≤ 20
**Reflections collected**	10404
**Independent reflections**	2305<br>Rint = 0.0786<br>Rsigma = 0.0515
**Completeness to θ = 67.684°**	98.7 %
**Data / Restraints / Parameters**	2305/0/164

**Goodness-of-fit on F2**	1.053
**Final R indexes [I≥2σ(I)]**	R1 = 0.0484<br>wR2 = 0.1279
**Final R indexes [all data]**	R1 = 0.0557<br>wR2 = 0.1370
**Largest peak/hole [eÅ−3]**	0.29/-0.30
**Flack X parameter**	0.1(5)

:

Compound **XVII (68)**:

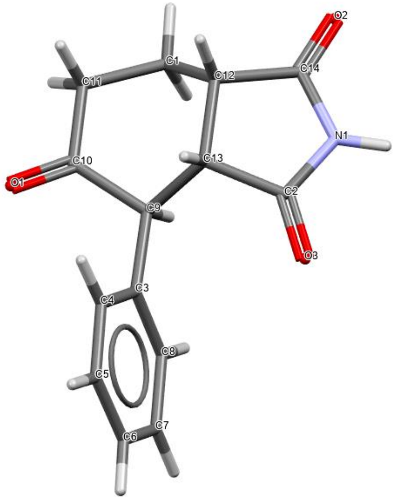

Figure S19. Asymmetric unit, showing the atom-labelling scheme. Displacement ellipsoids are drawn at the 50% probability level. Graphics were obtained using Mercury 3.0. [4]

Table S17. Crystal Data and details of the structure determination.

Empirical formula	C14H13NO3
Formula weight	243.25
Temperature [K]	104.3(2)
Crystal system	orthorhombic
Space group (number)	$P2_12_12_1$ (19)
a [Å]	6.04500(11)
b [Å]	11.69314(17)
c [Å]	16.3921(2)
α [°]	90
β [°]	90
γ [°]	90
Volume [Å3]	1158.67(3)
Z	4
ρcalc [gcm−3]	1.394
μ [mm−1]	0.812
F(000)	512
Crystal size [mm3]	0.236×0.141×0.08
Crystal colour	translucent light colourless

Crystal shape	block
Radiation	Cu Kα (λ=1.54184 Å)
2θ range [°]	9.29 to 145.05 (0.81 Å)
Index ranges	$-7 \leq h \leq 7$<br>$-14 \leq k \leq 14$<br>$-20 \leq l \leq 19$
Reflections collected	10929
Independent reflections	2280<br>Rint = 0.0424<br>Rsigma = 0.0276
Completeness to θ = 67.684°	100.0 %
Data / Restraints / Parameters	2280/0/163
Goodness-of-fit on F2	1.059
Final R indexes [I≥2σ(I)]	R1 = 0.0280<br>wR2 = 0.0676
Final R indexes [all data]	R1 = 0.0308<br>wR2 = 0.0696
Largest peak/hole [eÅ−3]	0.17/-0.14
Flack X parameter	-0.17(10)

SFC Traces:
Figure S20: Racemic Trace (**63)**

Empower 3 SOFTWARE

RA

SAMPLE INFORMATION

Sample Name:	AOC3_219re	Acquired By:	System
Sample Type:	Unknown	Sample Set Name:	AOC3_219re screen
Vial:	1:F,7	Acq. Method Set:	
Injection #:	1	Processing Method	
Injection Volume:	10.00 ul	Channel Name:	210.3nm
Run Time:	7.0 Minutes	Proc. Chnl. Descr.:	PDA 210.3 nm
Date Acquired:	9/16/2022 5:27:29 PM IST		
Date Processed:	2/3/2023 11:44:33 AM GMT		

Auto-Scaled Chromatogram

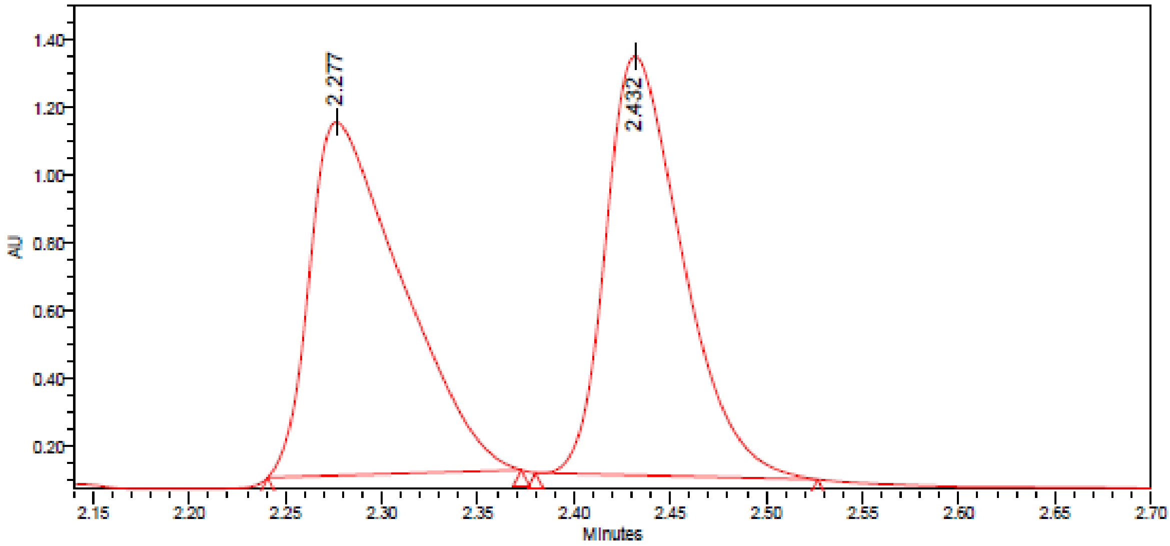

Peak Results

	Name	RT	Area	Height	% Area
1		2.277	3493736	1044398	50.41
2		2.432	3436711	1238451	49.59

Instrument Method: C4_99_1to70_30_5min_B1_3ml_min

Stored: 9/15/2022 5:49:27 PM IST

Method Information

Method Comments

Method Modified User System

Reported by User: System
Report Method: RA
Report Method ID 3105
Page: 1 of 5

Project Name: Aoibheann IVAOC
Date Printed:
2/3/2023
11:52:55 AM Europe/Dublin

SFC Traces:
Figure S21: Asymmetric Trace (**63**)

Empower 3 SOFTWARE   AOC383old

SAMPLE INFORMATION			
Sample Name:	aoc3_173	Acquired By:	System
Sample Type:	Unknown	Sample Set Name:	AOC3_173NO2PhtBu
Vial:	1:F,7	Acq. Method Set:	
Injection #:	1	Processing Method	trialtbunsphhydrolysed
Injection Volume:	10.00 ul	Channel Name:	210.3nm
Run Time:	7.0 Minutes	Proc. Chnl. Descr.:	PDA 210.3 nm
Date Acquired:	10/12/2022 3:23:07 PM IST		
Date Processed:	2/3/2023 12:45:50 PM GMT		

Auto-Scaled Chromatogram

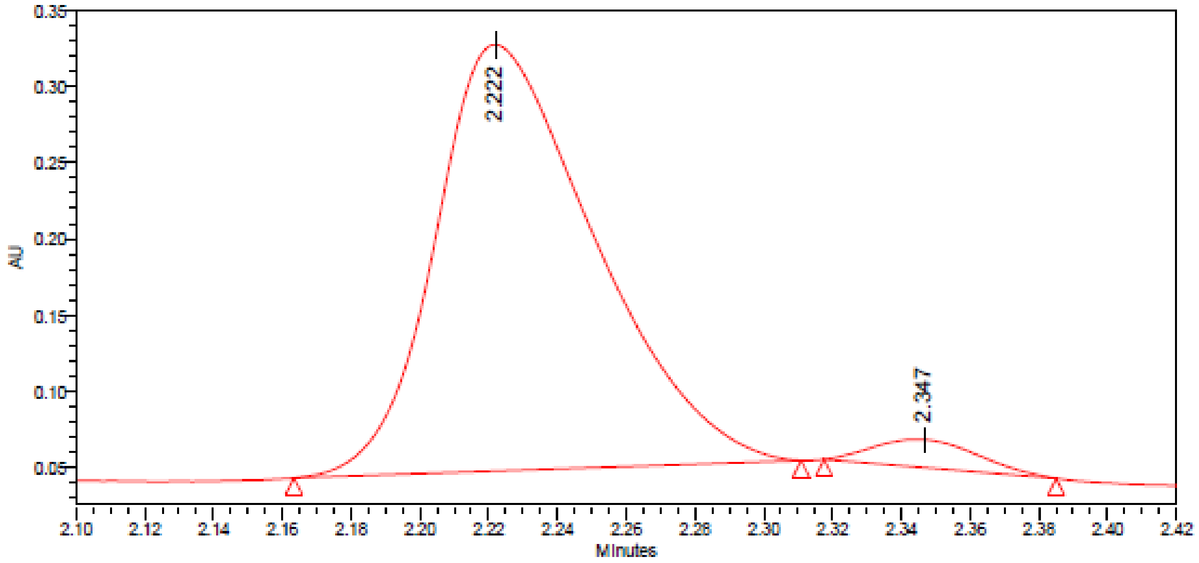

Peak Results

	Name	RT	Area	Height	% Area
1		2.222	904211	279795	95.89
2		2.347	38770	18443	4.11

Instrument Method: C4_99_1to70_30_5min_B1_3ml_min

Stored: 9/15/2022 5:49:27 PM IST

Method Information
Method Comments
Method Modified User System

Reported by User: System
Report Method: AOC383old
Report Method ID 3120
Page: 1 of 5

Project Name: Aoibheann I\AOC
Date Printed:
2/3/2023
12:47:48 PM Europe/Dublin

SFC Traces:
Figure S22: Racemic Trace (**64**)

Empower 3 SOFTWARE

SFC Rac Hydrolysed NSPhOMe 1

S A M P L E   I N F O R M A T I O N

Sample Name:	AOC_OMe_Rac_ACN	Acquired By:	System
Sample Type:	Unknown	Sample Set Name:	24Jan24_547wRac_ACN
Vial:	1:F,2	Acq. Method Set:	
Injection #:	1	Processing Method:	
Injection Volume:	10.00 ul	Channel Name:	219.7nm
Run Time:	8.0 Minutes	Proc. Chnl. Descr.:	PDA Spectrum PDA 219.7 nm (PDA
Date Acquired:	1/24/2024 4:24:56 PM GMT		
Date Processed:	1/24/2024 6:19:14 PM GMT		

Auto-Scaled Chromatogram

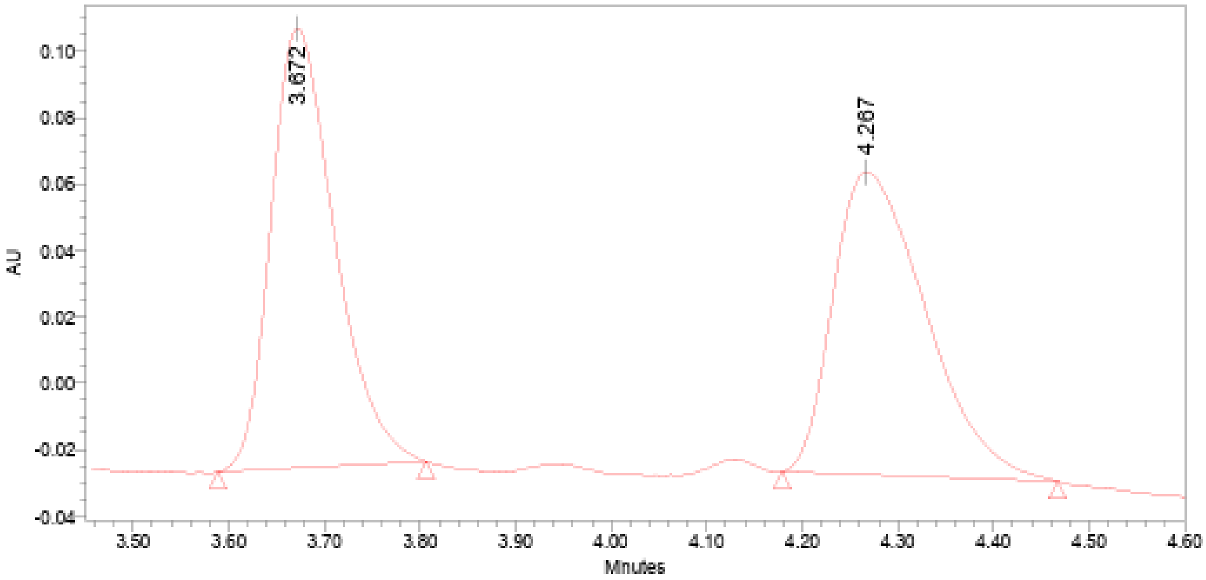

Peak Results

	Name	RT	Area	Height	Amount	Units	% Area
1		3.672	613737	132319			50.03
2		4.267	613029	91064			49.97

PDA Result Table

	Name	RT	Purity1 Angle	Purity1 Threshold	Match1 Spect. Name	Match1 Angle	Match1 Threshold
1		3.672					
2		4.267					

SFC Traces:

Figure S23: Asymmetric Trace (**64**)

Empower 3 SOFTWARE

SFC Asy Hydrolysed NSPhOMe 1

S A M P L E   I N F O R M A T I O N

Sample Name:	AOC_547_ACN	Acquired By:	System
Sample Type:	Unknown	Sample Set Name:	24Jan24_547wRac_ACN
Vial:	1:F,7	Acq. Method Set:	
Injection #:	1	Processing Method:	SFC tBu Hydrolysed NSPhOMe
Injection Volume:	10.00 ul	Channel Name:	219.7nm
Run Time:	8.0 Minutes	Proc. Chnl. Descr.:	PDA Spectrum PDA 219.7 nm (PDA
Date Acquired:	1/24/2024 4:44:39 PM GMT		
Date Processed:	1/24/2024 6:22:00 PM GMT		

Auto-Scaled Chromatogram

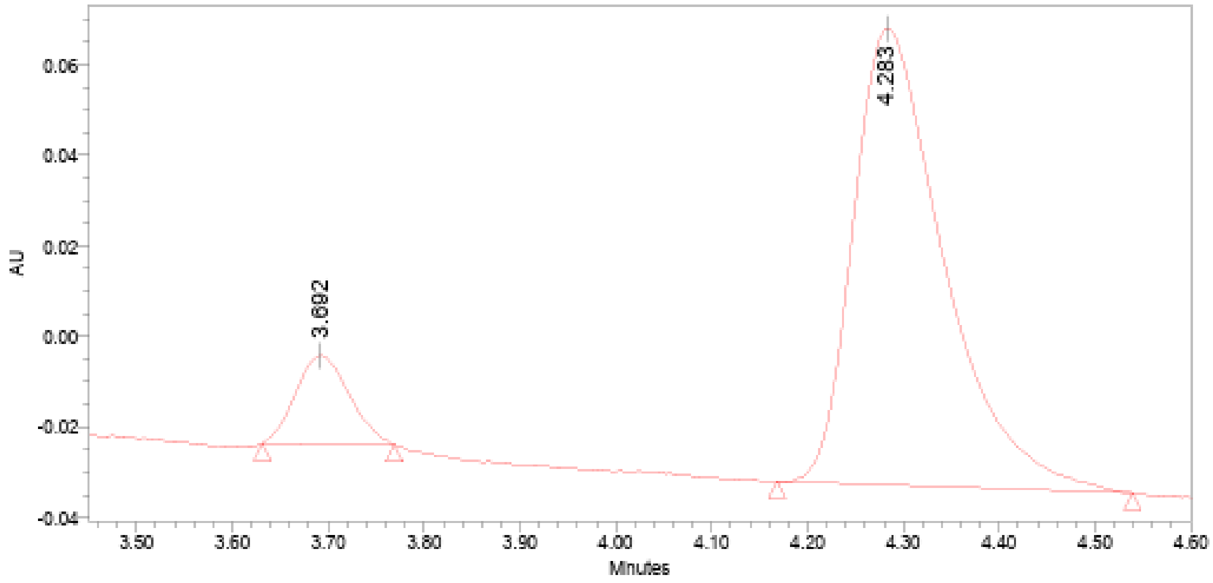

Peak Results

	Name	RT	Area	Height	Amount	Units	% Area
1		3.692	75433	19535			10.24
2		4.283	660898	100701			89.76

PDA Result Table

	Name	RT	Purity1 Angle	Purity1 Threshold	Match1 Spect. Name	Match1 Angle	Match1 Threshold
1		3.692					
2		4.283					

SFC Traces:

Figure S24: Racemic Trace (**65**)

Empower 3 SOFTWARE

Racemic Hydrolysed NSPhCl 3

SAMPLE INFORMATION

Sample Name:	Rac_Hydrolysed_Cl	Acquired By:	System
Sample Type:	Unknown	Sample Set Name:	AOC_ReRun_8Jan24
Vial:	1:F,4	Acq. Method Set:	
Injection #:	1	Processing Method:	tBu Hydrolysed Rac 3
Injection Volume:	10.00 ul	Channel Name:	216.0nm
Run Time:	5.0 Minutes	Proc. Chnl. Descr.:	PDA Spectrum PDA 216.0 nm (PDA
Date Acquired:	1/8/2024 6:16:21 PM GMT		
Date Processed:	1/8/2024 6:22:20 PM GMT		

Auto-Scaled Chromatogram

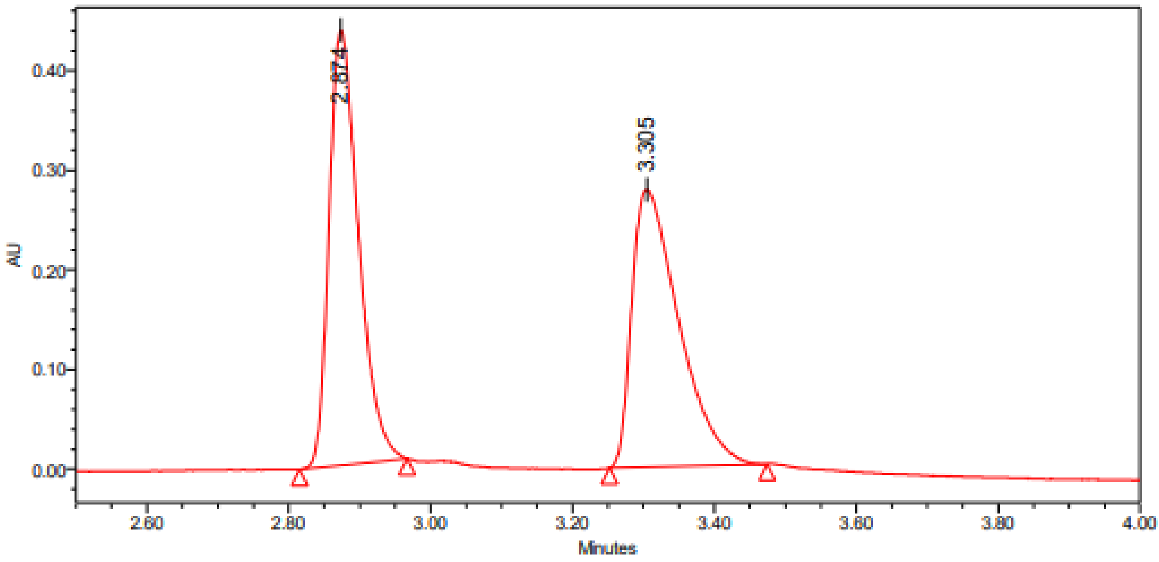

Peak Results

	Name	RT	Area	Height	Amount	Units	% Area
1		2.874	1260816	437118			49.74
2		3.305	1274007	278212			50.26

PDA Result Table

	Name	RT	Purity1 Angle	Purity1 Threshold	Match1 Spect. Name	Match1 Angle	Match1 Threshold
1		2.874					
2		3.305					

Reported by User: System
Report Method: Racemic Hydrolysed NSPhC
Report Method ID: 1444
Page: 1 of 1

Project Name: GuiryGroup\Aoibheann
Date Printed:
1/8/2024
6:46:18 PM Europe/London

SFC Traces:

Figure S25: Asymmetric Trace (**65**)

Empower 3 SOFTWARE

tBu Hydrolysed NSPhCl 3

SAMPLE INFORMATION

Sample Name:	AOC3_257As8020iPrOH	Acquired By:	System
Sample Type:	Unknown	Sample Set Name:	AOC3_257As8020acn
Vial:	1:F,3	Acq. Method Set:	
Injection #:	1	Processing Method:	tBu Hydrolysed Cl FINAL
Injection Volume:	5.00 ul	Channel Name:	218.4nm
Run Time:	5.0 Minutes	Proc. Chnl. Descr.:	PDA Spectrum PDA 218.4 nm (PDA
Date Acquired:	1/8/2024 12:01:39 PM GMT		
Date Processed:	1/8/2024 6:43:03 PM GMT		

Auto-Scaled Chromatogram

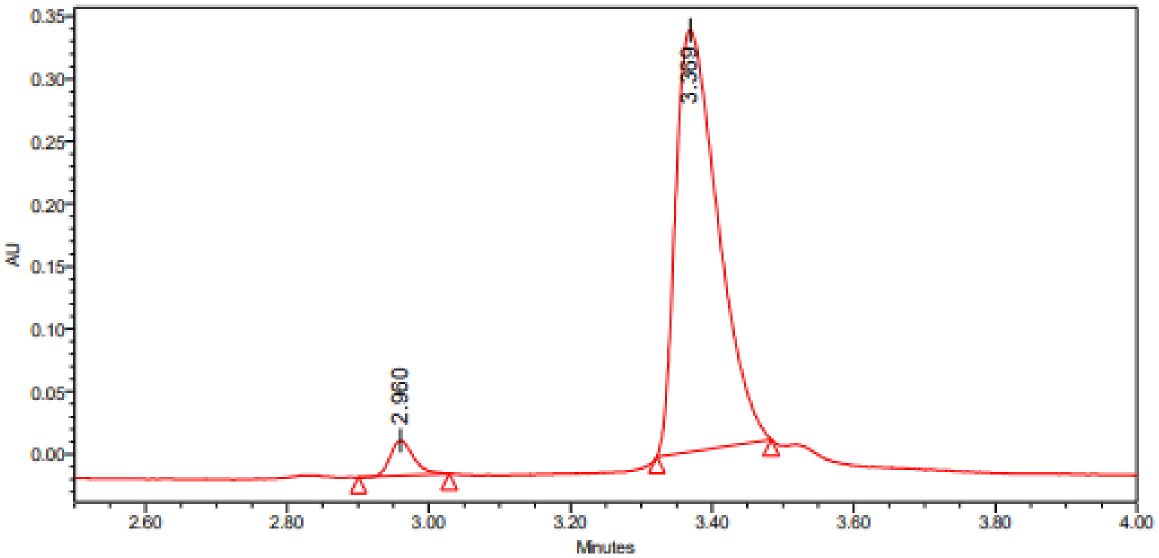

Peak Results

	Name	RT	Area	Height	Amount	Units	% Area
1		2.960	65294	28248			4.47
2		3.369	1395886	337182			95.53

PDA Result Table

	Name	RT	Purity1 Angle	Purity1 Threshold	Match1 Spect. Name	Match1 Angle	Match1 Threshold
1		2.960					
2		3.369					
